# Nickel-catalyzed enantioselective vinylation of aryl 2-azaallyl anions†‡

**DOI:** 10.1039/d1sc00972a

**Published:** 2021-03-26

**Authors:** Shengzu Duan, Guogang Deng, Yujin Zi, Xiaomei Wu, Xun Tian, Zhengfen Liu, Minyan Li, Hongbin Zhang, Xiaodong Yang, Patrick J. Walsh

**Affiliations:** Key Laboratory of Medicinal Chemistry for Natural Resource, Ministry of Education, Yunnan Provincial Center for Research & Development of Natural Products, School of Chemical Science and Technology, Yunnan University Kunming 650091 P. R. China xdyang@ynu.edu.cn zhanghb@ynu.edu.cn; Roy and Diana Vagelos Laboratories, Penn/Merck Laboratory for High-Throughput Experimentation, Department of Chemistry, University of Pennsylvania 231 South 34th Street Philadelphia PA USA pwalsh@sas.upenn.edu liminyan@sas.upenn.edu

## Abstract

A unique enantioselective nickel-catalyzed vinylation of 2-azaallyl anions is advanced for the first time. This method affords diverse vinyl aryl methyl amines with high enantioselectivities, which are frequently occurring scaffolds in natural products and medications. This C–H functionalization method can also be extended to the synthesis of enantioenriched 1,3-diamine derivatives by employing suitably elaborated vinyl bromides. Key to the success of this process is the identification of a Ni/chiraphos catalyst system and a less reducing 2-azaallyl anion, all of which favor an anionic vinylation route over a background radical reaction. A telescoped gram scale synthesis and a product derivatization study confirmed the scalability and synthetic potential of this method.

## Abstract 2

A unique enantioselective Ni-catalyzed vinylation of 2-azaallyl anions is advanced. This method affords vinyl aryl methyl amine or 1,3-diamine derivatives with high enantioselectivities, which are frequently occurring scaffolds in medications.

## Graphical abstract

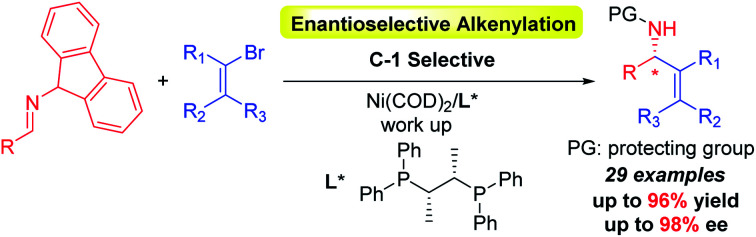

## Introduction

**Fig. 1:**
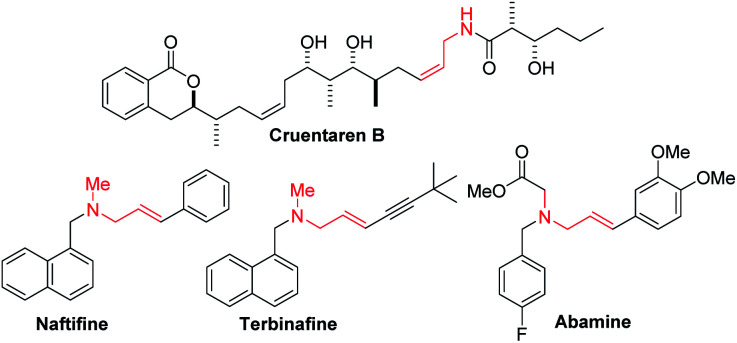
Examples of allylic amine-containing pharmaceuticals.

Enantioenriched amines are among the most important structural motifs in the pharmaceutical industry.^1^ It has been estimated that chiral amines are substructures in 40% of current pharmaceuticals.^2,3^ Among amine-containing molecules, allylic amines are an important sub-class in the pharmaceutical industry (cruentaren B, naftifine, terbinafine and abamine, Fig. 1). Moreover, allylic amines are fundamental building blocks in synthesis.^4,5^ Enantioenriched allylic amines, however, are often difficult to synthesize using asymmetric catalysis.^6,7^

**Scheme 1:**
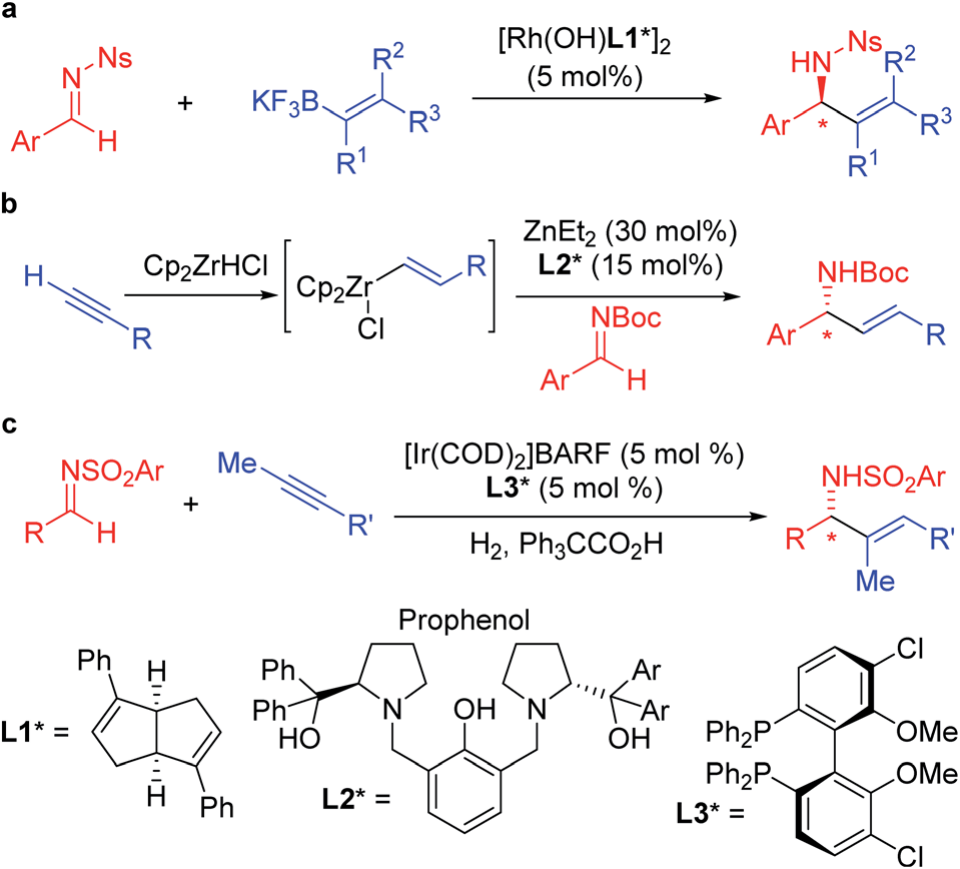
Enantioenriched allylic amine synthesis *via* asymmetric additions to imines.

In recent years, practical synthetic routes toward enantioenriched amines have been advanced by Ellman,^8–10^ Carreira^11–15^ and others.^16–21^ Enantioenriched allylic amines are desirable targets because of their utility, and several methods have been reported that involve the asymmetric addition of organometallic reagents to activated imines in the presence of enantioenriched catalysts. Early work on the rhodium catalyzed asymmetric arylation of imines by Hayashi's group^22^ inspired the use of vinyl trifluoroborates, as exemplified by the work of Lin and Wu (Scheme 1a).^23,24^ Other approaches based on inexpensive metals, such as Trost's alkyne hydrozirconation followed by zinc-Pro-phenol-based catalyzed asymmetric addition to *N*-Boc activated aldimines have received attention (Scheme 1b).^25^ An impressive asymmetric imine vinylation reaction was reported by Krische starting with imine and alkyne in the presence of hydrogen and a chiral iridium-based catalyst (Scheme 1c).^26^ The intermediate vinyl iridium species is diverted from hydrogenation to the asymmetric vinylation process. Other interesting approaches have also been documented.^27^

With an interest in the synthesis of amines, several groups have focused on the Umpolung reactivity of *N*-benzyl ketimine derivatives. Upon deprotonation under mild conditions, *N*-benzyl ketimines form 2-azaallyl anions that can be functionalized in transition metal catalyzed processes, or under transition metal-free conditions, to provide various amines.^28,29^ This strategy of deprotonation of *N*-benzyl ketimines to generate intermediate 2-azaallyl anions as reactive nucleophiles benefits from its avoidance of preformed organometallics that are common reagents in C–C bond forming reactions through cross-coupling processes.

**Scheme 2:**
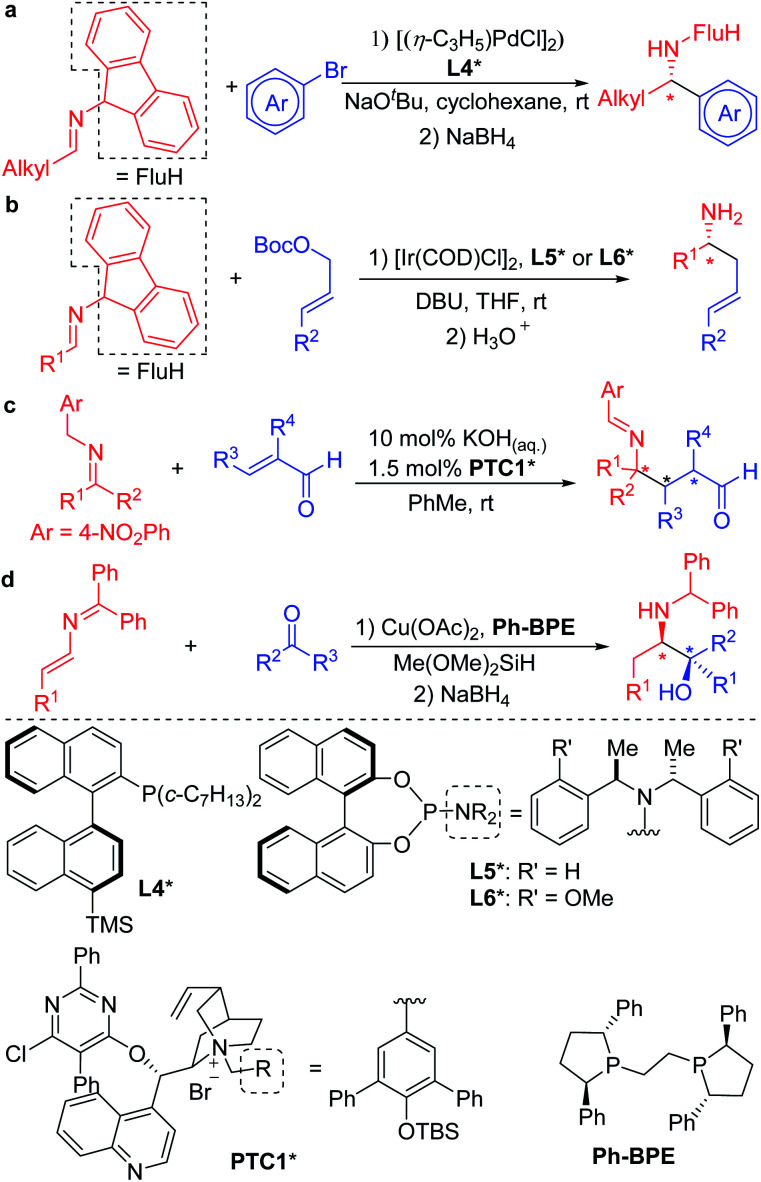
Chiral amine synthesis from enantioselective functionalization of 2-azaallyl anions.

The mild nature of semi-stabilized 2-azaallyl anions has made them targets for use in enantioselective functionalization reactions. Successful examples include Buchwald and Zhu's pioneering enantioselective Pd catalyzed arylation of alkyl 2-azaallyl anions with tailored chiral phosphine L4* (Scheme 2a).^30^ Enantioselective allylic substitution with 2-azaallyl anion nucleophiles has attracted the attention of groups including Niu,^31–34^ Chruma,^35,36^ You,^37–41^ and Han.^42^ Among these, the iridium catalyzed asymmetric allylic substitution with ligands L5* and L6* stand out as highly enantioselective (Scheme 2b).^29^ Deng and coworkers^43–46^ reported an impressive functionalization of trifluoromethyl amines using asymmetric conjugate additions (Scheme 2c).^43^ Here, the 4-nitro group proved essential to stabilize the 2-azaallyl anion, enabling the deprotonation with KOH in the presence of phase-transfer catalyst PTC1*. The nitrobenzyl moiety is also likely responsible for the regioselectivity of the C-3 functionalization, which results in the formation of quaternary stereocenters. A novel strategy was employed by the Malcolmson's group^47,48^ who started with 2-azadienes and an enantioenriched copper catalyst. Hydrocupration generates an enantioenriched copper complex with the bound 2-azaallyl anion that adds in an enantioselective fashion to the carbonyl group (Scheme 2d).^47^

**Scheme 3:**
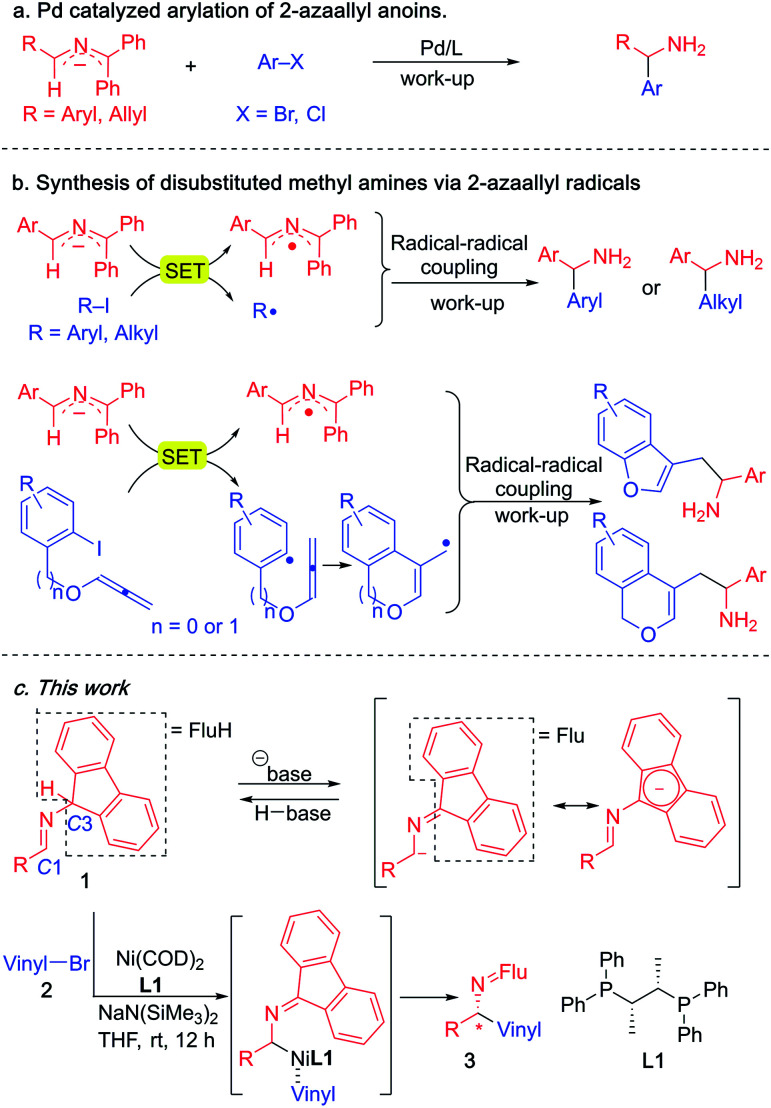
Reaction of 2-azaallyl anions from our team. (a) Pd catalyzed racemic arylation of 2-azaallyl anions. (b) Transition metal-free radical coupling reactions. (c) This work, the enantioselective vinylation of 2-azaallyl anions.

Since 2014, our group has accessed a wide variety of diarylmethylamines through the functionalization of 2-azaallyl anions (Scheme 3a).^49,50^ We also discovered the unique reducing feature of 2-azaallyl anions and developed a series of methods for the efficient transition metal-free synthesis of aryl-, alkyl- and allyl-methylamines from 2-azaallyl radicals (Scheme 3b).^51–54^ Herein, we continue our journey in 2-azaallyl chemistry by developing the first enantioselective nickel-catalyzed vinylation of 2-azaallyl anions (Scheme 3c). Successful identification of Ni(COD)_2_/chiraphos is key for the enantioselectivity. A wide range of imines and vinyl bromides are tolerated under the mild reaction conditions with no C-3 vinylation^51^ or base promoted product isomerization observed. We also conducted a telescoped gram scale synthesis and product derivatization study to demonstrate the scalability and synthetic potential of the current method. It is noteworthy that the methods developed by Buchwald's and Niu's groups (Scheme 2) involve expensive precious metals and/or ligands and are not suitable for the synthesis of the enantioenriched allylic amines reported herein.

## Results and discussion

### Reaction development and optimization

Prompted by our previous experience with benzophenone *N*-benzyl imines, which readily participate in single electron processes upon deprotonation (Scheme 3b), the Pd catalyzed 2-azaallyl anion arylation^49,50,55,56^ (Scheme 3a) and Ni catalyzed cross-coupling^57–61^ chemistry, we selected Ni(COD)_2_ as the nickel source and the mild base LiO^*t*^Bu to deprotonate the imine **1a**. We wanted to avoid complication by SET steps and radical intermediates, as proposed by Ohshima's team in their recent copper-catalyzed coupling reactions synthesis of hindered amino acids using 2-azaallyl anions.^62^ To lower the reducing tendencies of the 2-azaallyl anion intermediates, we opted to use fluorenyl amine derivatives.

We began to explore this reaction by examining 26 chiral ligands (see ESI, Table S1‡ for details) under the conditions listed in Table 1. The top hits, as judged by product enantiomeric excess, were observed with Fryzuk and Bosnich's (*S*,*S*)-chiraphos^63^ (**L1**, 78% ee, 65% yield, entry 1) and Ph-BPE (**L2**, 47% ee, 52% yield, entry 2). We found that a phosphine–oxazoline ligand **L3** afforded the target product **3aa** in 55% ee and 47% yield. When BOX ligands **L4** [2,2′-(propane-2,2-diyl)bis(4-phenyl-4,5-dihydrooxazole)] and **L5** [2,2′-(propane-2,2-diyl)bis(4-benzyl-4,5-dihydrooxazole)] were used we observed formation of **3aa** in 66% and 75% yield, respectively, but were surprised to find that both gave racemic product. In addition to bidentate ligands, mono-dentate phosphine ligand **L6** afforded 45% ee, albeit in 34% yield. Based on these results, we continued to use chiraphos (**L1**), which afforded the highest product ee and yield in the initial screen.

**Table 1:** Optimization of vinylation of imine **1a**^a^^,^^b^

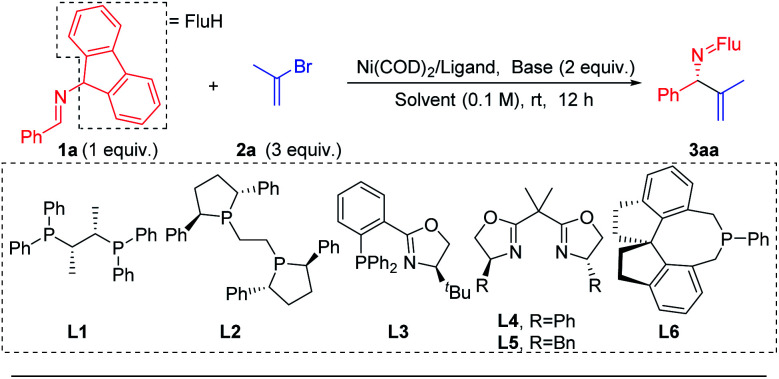						
Entry	**L**	Ni/**L** (mol%)	Base	Solvent	**3aa** (%)	ee (%)
1	**L1**	5/10	LiO^*t*^Bu	THF	65	78
2	**L2**	5/10	LiO^*t*^Bu	THF	52	47
3	**L3**	5/10	LiO^*t*^Bu	THF	47	55
4	**L4**	5/10	LiO^*t*^Bu	THF	66	0
5	**L5**	5/10	LiO^*t*^Bu	THF	75	0
6	**L6**	5/10	LiO^*t*^Bu	THF	34	45
7	**L1**	5/10	NaO^*t*^Bu	THF	23	23
8	**L1**	5/10	KO^*t*^Bu	THF	6	—
9	**L1**	5/10	LiN(SiMe_3_)_2_	THF	63	84
10	**L1**	5/10	NaN(SiMe_3_)_2_	THF	94	92
11	**L1**	5/10	KN(SiMe_3_)_2_	THF	42	82
12	**L1**	5/10	NaN(SiMe_3_)_2_	CPME	62	48
13	**L1**	5/10	NaN(SiMe_3_)_2_	MTBE	28	20
14	**L1**	5/10	NaN(SiMe_3_)_2_	Et_2_O	32	14
15^c^	**L1**	5/10	NaN(SiMe_3_)_2_	THF	95	93
16	**L1**	2.5/5	NaN(SiMe_3_)_2_	THF	62	92
17^d^	**L1**	5/10	NaN(SiMe_3_)_2_	THF	4	—

a Reactions conducted on a 0.2 mmol scale with 2 equiv. base.

b Isolated yield of **3aa** after chromatographic purification; ee (enantiomeric excess) of **3aa** was determined by chiral phase HPLC.

c NaN(SiMe_3_)_2_ (1.5 equiv.).

d Pd(OAc)_2_ instead of Ni(COD)_2_.

The next variable examined in the optimization was the base. At the outset of this work, we were concerned that a base that could deprotonate the aldimine substrate, might also deprotonate the product, as was observed by Ohshima,^62^ resulting in product racemization and possibly partial isomerization. We were also cognizant that transmetallation would likely be the enantiodetermining step and, if true, the nature of the main group metal associated with the 2-azaallyl anion would be important. We tested 5 different bases that could deprotonate the aldimine [NaO^*t*^Bu, KO^*t*^Bu, LiN(SiMe_3_)_2_, NaN(SiMe_3_)_2_, KN(SiMe_3_)_2_, entries 7–11]. We were delighted to discover that NaN(SiMe_3_)_2_ provided the desired product in 94% yield with 92% ee (Table 1, entry 10). We then turned our attention to probing the impact of the solvent. Three solvents were evaluated [(CPME (cyclopentyl methyl ether), MTBE (methyl *tert*-butyl ether), and diethyl ether (entries 12–14)], however none of these rivaled the results with THF in entry 10. Dropping the equivalents of base from 2 to 1.5 led to a slight increase in the yield to 95% and the product ee to 93% (entry 15). An attempt to lower the catalyst loading to 2.5 mol% afforded a synthetically acceptable yield of 62% and high enantioselectivity (92% ee, entry 16). Notably, under otherwise identical conditions to entry 15, switching from Ni(COD)_2_ to Pd(OAc)_2_ significantly decreased the yield (4%, entry 17, see ESI, Table S5‡ for details).

It is interesting to note that various groups,^35,36^ including ours,^49–51^ observed regioselectivity issues with 2-azaallyl anions, wherein partial substitution took place at the more hindered C-3 position of the azaallyl group. Regioselectivity issues in the functionalization of 2-azaallyl anions can be problematic in the application of the methods, because the C-1 and C-3 isomers are usually difficult to separate. We were pleased to find that the regioselectivity in our nickel catalyzed process was very high and C-3 products were not observed.

### Scope of imines

With the optimized conditions in hand, a range of aldimines were subjected to the nickel catalyzed enantioselective vinylation. As shown in Table 2, isolated yields of the corresponding allylic amine derivatives were generated in >63% and most enantioselectivities are >85%. A range of *para*-substituted aldimines underwent the vinylation with 2-bromopropene regardless of the electronic nature of the substituent. For example, electron rich imine **1b** (4-NMe_2_) afforded **3ba** in 96% yield and 91% ee. Despite the high aptitude of nickel complexes to undergo oxidative additions of aryl halides and other C–X bonds,^63^ imines bearing halogens (**1c**, 4-F; **1d**, 4-Cl; and **1e**, 4-Br) furnished the allylic imine products in 91%, 63% and 82% yields with 90%, 86% and 85% ee, respectively. Thus, the catalyst displays a high degree of chemoselectivity in the oxidative addition of C(sp^2^)–Br bonds.

**Table 2:** Scope of aldimines^a^^,^^b^^,^^c^

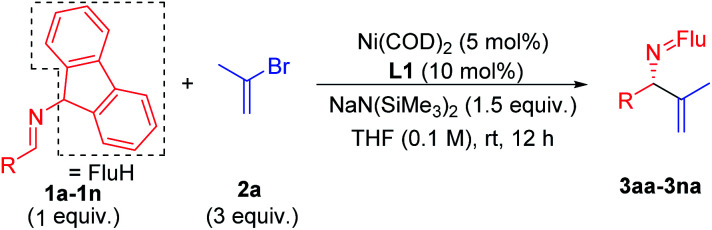
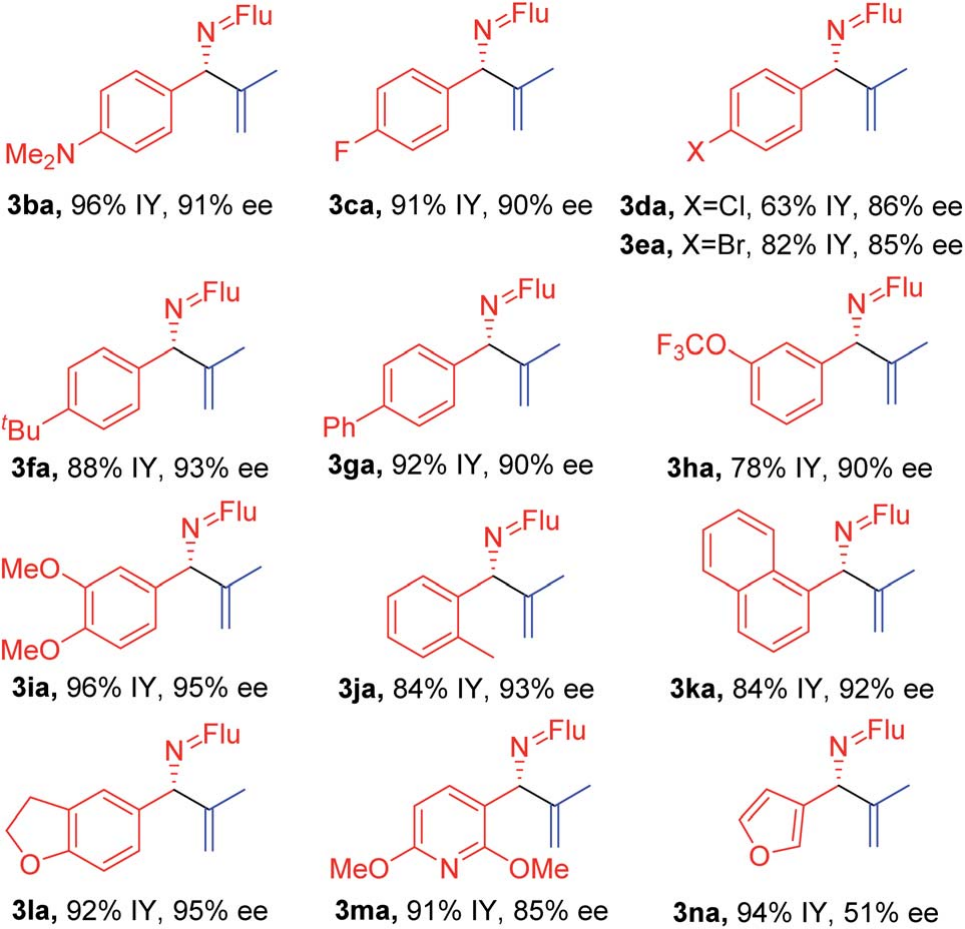

a Reactions conducted on a 0.4 mmol scale using 1 equiv. **1** and 3 equiv. **2a** at 0.1 M.

b Isolated yields after chromatographic purification. Flu = 9-fluorenyl.

c ee's of imine products were determined by chiral phase HPLC analysis.

Aldimines possessing electronically neutral substituents, including 4-^*t*^Bu and 4-Ph, performed well, providing the desired products (**3fa** and **3ga**) in 88% and 92% yields with 93% and 90% ee, respectively. Substrates bearing *meta*-substituents, like 3-OCF_3_, resulted in 78% yield of **3ha** with 90% ee. A 3,4-dimethoxy substituted imine (**2i**) led to target product **3ia** in 96% yield with 95% ee. The sterically hindered 2-tolyl imine and 1-natphthyl imine did not impact the catalyst enantioselectivity, giving **3ja** and **3ka** both in 84% yield with 92–93% ee.

Heterocycle-containing structures are of great value to the pharmaceutical industry.^64,65^ With this in mind, selected heterocycles were incorporated into the imine substrates. The dihydrobenzofuran derived imine was converted to the corresponding product **3la** in 92% yield with 95% ee. Pyridines are among the most prevalent heterocycles in medicinal chemistry^66^. To our delight, the pyridyl-based substrate **1m** underwent the vinylation in 91% yield with 85% ee. An imine bearing a 3-furyl group provided the product **3na** in 94% yield, but ee dropped to 51%. We were worried that the product **3na** might have undergone racemization *via* deprotonation by base followed by reprotonation. As such, we monitored the product ee as a function of time by analyzing samples from the reaction at 3.0, 6.0 and 9.0 h. The ee of **3na**, however, remain 51% over the time course of the reaction (see ESI, Table S6‡ for details).

### Scope of the vinyl bromide coupling partners

Substituted vinyl bromides possessing aliphatic groups, heterocycles, and extended ring systems were next explored. As shown in Table 3, diverse vinyl bromides were amenable to the asymmetric additions. Use of the parent 1-bromo ethylene (**2b**) enabled the isolation of the vinylation product **3ab** with 87% ee and 60% yield. Replacing the methyl group of 2-bromo propene with an ethyl group (**2c**) did not impact the yield (90%) or the product ee (93%) compared to the model reaction. In contrast, the isomeric *trans*-2-bromo-2-butene (**2d**), containing a trisubstituted alkene, was more challenging and furnished the product in 62% yield with 71% ee.

**Table 3:** Scope of vinyl bromides^a^^,^^b^^,^^c^

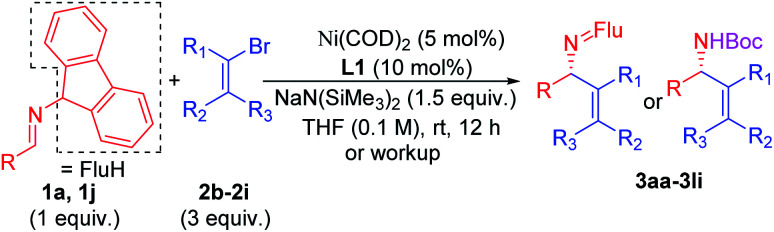
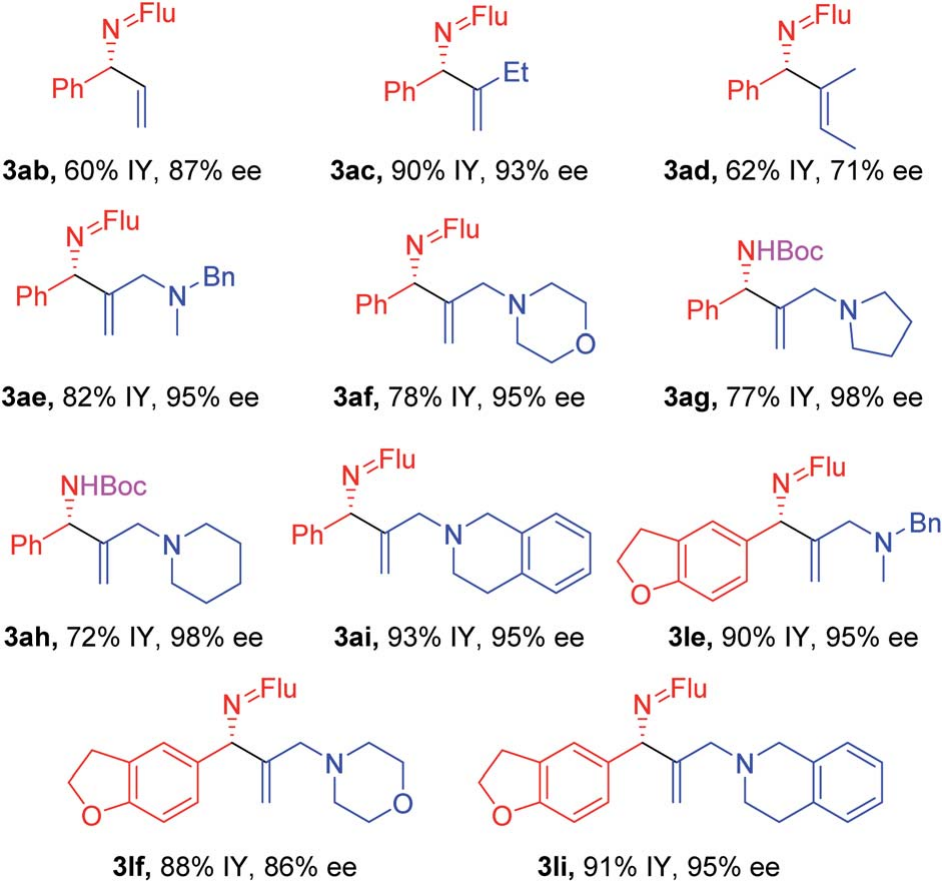

a Reactions conducted on a 0.4 mmol scale using 1 equiv. **1a**, **1j** and 3 equiv. **2** at 0.1 M.

b Isolated yields after chromatographic purification. Flu = 9-fluorenyl.

c ee's of imine products were determined by HPLC analysis.

We next examined vinyl bromide substrates bearing amino groups to prepare diamine derivatives. Thus, coupling of **1a** with *N*-benzyl-2-bromo-*N*-methylprop-2-en-1-amine (**2e**) delivered the diamine derivative **3ae** in 82% yield and 95% ee. Cyclic analogs 4-(2-bromoallyl)morpholine (**2f**), 1-(2-bromoallyl)pyrrolidine (**2g**) and 1-(2-bromoallyl)piperidine (**2h**) were next subjected to the optimized reaction conditions, affording the products in 72–78% yields and 95–98% ee. The efficiency of the reaction was maintained when a vinyl bromide bearing extended ring system on the methylene carbon (**2i**) was employed, furnishing heterocyclic diamine derivative **3ai** (93% yield, 95% ee).

Imine **1l**, with a heteroaromatic scaffold, was selected for coupling with three vinyl bromides (**2e**, **2f** and **2i**), producing **3le**, **3lf** and **3li** in excellent yields (88–91%) and enantioselectivities (86–95% ee). It is noteworthy that these enantioenriched diamine derivatives would be difficult to prepare by other methods.

### Gram scale synthesis and product derivatization

**Scheme 4:**
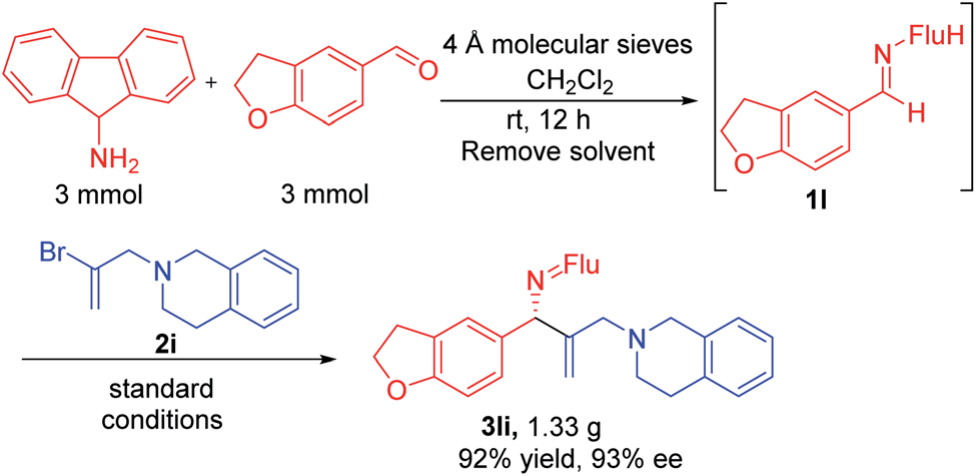
Gram-scale sequential one-pot imine synthesis/vinylation.

**Scheme 5:**
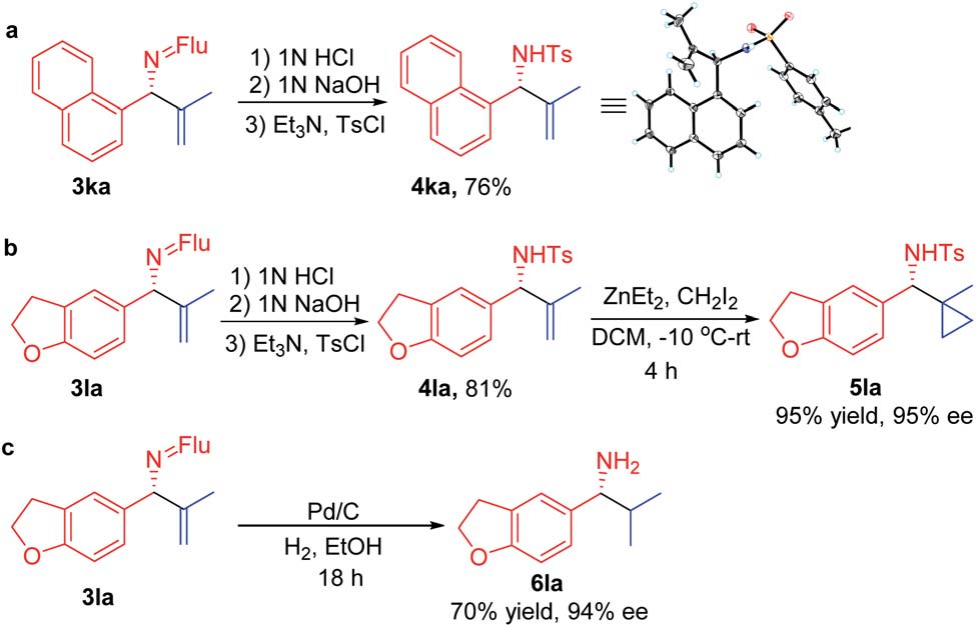
Transformation of the products.

In order for a method to be useful, it must be scalable. To test the scalability of our enantioselective vinylation, we explored the telescoped imine formation/asymmetric vinylation procedure on gram scale (Scheme 4). To our delight, product **3li** was successfully prepared in overall 92% yield (1.33 g) with 93% ee, demonstrating the potential application on larger scales. In order to determine the facial selectivity of the reaction and the absolute configuration of the asymmetric vinylation, we hydrolyzed **3ka** to the parent amine, then re-protected with TsCl to increase the crystallinity. The configuration of product **4ka** was determined to be (*R*) by single crystal X-ray analysis (Scheme 5a, CCDC 2058299‡).

To demonstrate the synthetic utility of the allylic amine products, we explored derivatization. Cyclopropyl amines are common building blocks in the pharmaceutical industry.^67^ Thus, conversion of **3la** to the corresponding sulfonamide **4la** was readily accomplished in 81% yield. Subjecting the resulting sulfonamide to diethylzinc and diiodomethane led to cyclopropyl derivative **5la** in 95% yield (Scheme 5b). Importantly, the ee of **4la** and **5la** were preserved through these transformations. Hydrogenation of the allylic double bond was also conducted using **3la** with Pd/C. The hydrogenated and deprotected amine was isolated in 70% yield. This result bodes well for the synthesis of enantioenriched amines with aliphatic substituents that are otherwise difficult to access but are of great value in pharmaceutical industry (Scheme 5c).

## Conclusions

In summary, we describe the first development of a highly enantioselective, convenient and practical vinylation of 2-azaallyl anions. The current method enables the synthesis of a wide variety of enantiomerically enriched allylic amines, including highly functionalized 1,3-diamine derivatives. A telescoped procedure has been introduced that is applicable to the gram scale preparation of a highly enantioenriched allylic 1,3-diamine derivative. The catalyst is based on a commercially available diphosphine, chiraphos, and a widely used nickel source. Overall, this method constitutes a straightforward and practical contribution to the asymmetric functionalization of 2-azaallyl anions.

## Author contributions

S. D. & G. D. contributed equally to this work. X. Y. conceived of the project. M. L., H. Z. and P. J. W. designed the experiments. S. D., G. D., Y. Z., X. W., X. T. and Z. L. performed the research. M. L., X. Y. and P. J. W. wrote the manuscript.

## Conflicts of interest

There are no conflicts to declare.

## Supplementary Material

SC-012-D1SC00972A-s001

SC-012-D1SC00972A-s002
