# Supplementary material for: Nickel-catalyzed enantioselective vinylation of aryl 2-azaallyl anions
Source: Chem Sci. 2021 Mar 26;12(18):6406–12. doi: 10.1039/d1sc00972a (PMC8115067; doi:10.1039/d1sc00972a)
Supplement: SC-012-D1SC00972A-s001 [file SC-012-D1SC00972A-s001.pdf]

Electronic Supplementary Material (ESI) for Chemical Science.
This journal is © The Royal Society of Chemistry 2021

Electronic Supplementary Information (ESI)

Nickel-Catalyzed Enantioselective Vinylation of Aryl 2-Azaallyl Anions

Shengzu Duan,†[a] Guogang Deng,†[a] Yujin Zi,[a] Xiaomei Wu,[a] Xun Tian,[a] Zhengfen Liu,[a] Minyan Li,*[b] Hongbin Zhang,*[a] Xiaodong Yang*[a] & Patrick J. Walsh*[b]

[a] Key Laboratory of Medicinal Chemistry for Natural Resource, Ministry of Education; Yunnan Provincial Center for Research & Development of Natural Products; School of Chemical Science and Technology, Yunnan University, Kunming, 650091, P. R. China. *e-mail:xdyang@ynu.edu.cn; zhanghb@ynu. edu.cn

[b] Roy and Diana Vagelos Laboratories, Penn/Merck Laboratory for High-Throughput Experimentation, Department of Chemistry, University of Pennsylvania, 231 South 34th Street, Philadelphia, Pennsylvania, 19104, USA. *e-mail: pwalsh@sas.upenn.edu; liminyan@sas.upenn.edu

†S. D. & G. D. contributed equally.

TABLE OF CONTENT

1. General methods……………………………………………………………………...…....………..S2

2. Preparation of imines…………………………………………………………………...….……..S3

3. Preparation of alkenyl bromides…………………………………………………………………S5

4. Ni-catalyzed asymmetric alkenylation of imines (Table 1): *Lab scale reaction optimization of Chiral ligands, solvents, bases, ratio of Ni : ligand and switching from Ni to Pd as metal source*………………..S7

5. General procedure and characterization of Ni-catalyzed asymmetric alkenylation of imines with alkenyl bromides (Tables 2 and 3) …………………………………………………………….………..S11

6. Gram-scale sequential one-pot asymmetric imine synthesis/vinylation procedure……………….………S22

7. Transformation of the products…………………………………………………………...….……S23

8. Reaction time course study of coupling between **1n** and **2a** (Table S6).…………………….….……...S25

9. X-ray crystal structure of compound **4ka**……………………………………………………...………..S26

10. Supplementary references………………………………………….………………......……...S28

11. NMR spectra and HPLC chromatography of the products……….…...……………………………S29

1. General methods.

All air- and moisture-sensitive solutions and chemicals were handled under a nitrogen atmosphere of a glovebox and solutions were transferred via "Titan" brand pipettor. Anhydrous solvents, including DME (dimethoxyethane), CPME (cyclopentyl methyl ether), MTBE (methyl *tert*-butyl ether), toluene, tetrahydrofuran (THF), cyclohexane, Et_2O (diethyl ether) and 1,4-dioxane were purchased from Sigma-Aldrich and used without purification. Unless otherwise stated, all reagents were commercially available and used as received without purification. $Ni(COD)_2$ was purchased from Sigma-Aldrich and used as received. Chiral ligands were purchased from TCI and J&K. Other chemicals were obtained from Sigma-Aldrich, Acros, TCI and Alfa-Aesar. TLC was performed with Merck TLC Silica gel60 F_{254} plates with detection under UV light at 254 nm. Silica gel (200-300 mesh, Qingdao) was used for flash chromatography. Deactivated silica gel was prepared by addition of 15 mL Et_3N to 1 L of silica gel. The products were purified with XDB-C_{18} (9.4 × 250 mm, 5 μm) column on an Agilent HPLC 1260 system. ^1H and $^{13}C\{^1H\}$ NMR spectra were obtained using a Brüker DRX 400 spectrometer at 400 MHz and 100 MHz, respectively. Chemical shifts were reported in units of parts per million (ppm) downfield from tetramethylsilane (TMS), and all coupling constants were reported in hertz. The infrared (IR) spectra were measured on a Nicolet iS10 FTIR spectrometer with 4 cm^{-1} resolution and 32 scans between wavenumber of 4000 cm^{-1} and 400 cm^{-1}. High Resolution Mass spectra were taken on AB QSTAR Pulsar mass spectrometer. Melting points were obtained on a XT-4 melting-point apparatus and were uncorrected. The enantiomeric excess was determined by chiral phase HPLC with *n*-hexane and *i*-propanol as eluents. Optical rotations were measured on a JASCO DIP-370 polarimeter at the indicated temperature (20 ºC) with a sodium lamp (D line, 589 nm).

2. Preparation of imines

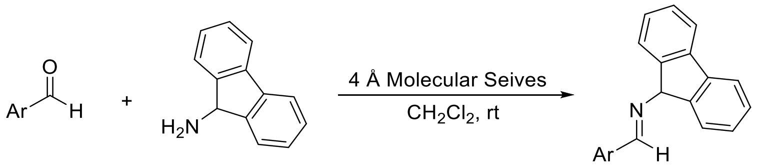

N-fluorenyl imines (**1a, 1b, 1d, 1e** and **1n**) were prepared following reported procedures.[1,2]

To a solution of an aldehyde and 9*H*-fluoren-9-amine (1:1 mixture) in CH_2Cl_2 (0.2 M) at room temperature was added 4 Å molecular sieves (0.3 g/mmol). The mixture was stirred at room temperature until completion (ca. 12 h), as indicated by ^{1}H NMR, and filtered. The filtrate was concentrated and triturated with hexanes to give the corresponding *N*-fluorenyl imines as a solid that is sufficiently pure for further reactions.

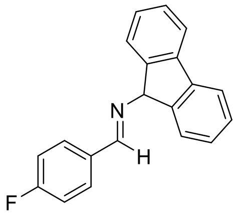

(*E*)-*N*-(9*H*-Fluoren-9-yl)-1-(4-fluorophenyl)methanimine (1c). ^{1}H NMR (400 MHz, Chloroform-*d*) δ 8.64 (s, 1H), 7.73 – 7.66 (m, 4H), 7.34 – 7.29 (m, 4H), 7.21 (td, *J* = 7.2, 1.2 Hz, 2H), 7.00 (t, *J* = 8.4 Hz, 2H), 5.32 (s, 1H) ppm; **^{13}C{^{1}H} NMR** (100 MHz, Chloroform-*d*) δ 164.6 (d, $^1J_{C\text{-}F}$ = 249.7 Hz), 162.0, 144.8, 141.2, 132.5 (d, $^4J_{C\text{-}F}$ = 3.1 Hz), 130.6 (d, $^3J_{C\text{-}F}$ = 8.7 Hz), 128.6, 127.6, 125.4, 120.3, 115.8 (d, $^2J_{C\text{-}F}$ = 22.0 Hz), 74.7 ppm; **IR** (thin film): 3357, 3057, 2878, 1628, 1448, 1227, 1040, 816, 727 cm^{-1}; **^{19}F NMR** (376 MHz, Chloroform-*d*) δ -108.9 (s, 1F). **HRMS** calc'd for $C_{20}H_{15}FN^+$ 288.1183, found 288.1176 $[M+H]^+$. **Mp**: 138 – 140 °C.

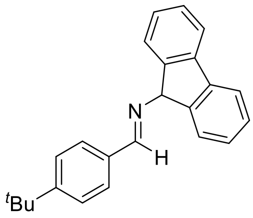

(*E*)-1-(4-(*tert*-Butyl)phenyl)-*N*-(9*H*-fluoren-9-yl)methanimine (1f). ^{1}H NMR (400 MHz, Chloroform-*d*) δ 8.68 (s, 1H), 7.70 – 7.66 (m, 4H), 7.37 – 7.28 (m, 6H), 7.22 – 7.19 (m, 2H), 5.31 (d, *J* = 2.4 Hz 1H), 1.25 (s, 9H) ppm; **^{13}C{^{1}H} NMR** (100 MHz, Chloroform-*d*) δ 163.5, 154.6, 145.1, 141.2, 133.5, 128.5, 127.5, 125.7, 125.4, 120.2, 75.0, 35.1, 31.3 ppm, one resonance was not observed due to overlapping peaks; **IR** (thin film): 3390, 3064, 2850, 1633, 1450, 1233, 1044, 802, 743 cm^{-1}; **HRMS** calc'd for $C_{24}H_{24}N^+$ 326.1903, found 326.1901 $[M+H]^+$. **Mp**: 124 – 126 °C.

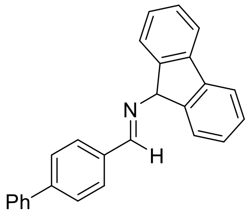

(*E*)-1-([1,1'-Biphenyl]-4-yl)-*N*-(9*H*-fluoren-9-yl)methanimine (1g). ^{1}H NMR (400 MHz, Chloroform-*d*) δ 8.72 (s, 1H), 7.81 – 7.78 (m, 2H), 7.69 – 7.66 (m, 2H), 7.57 – 7.50 (m, 4H), 7.37 – 7.19 (m, 9H), 5.35 (s, 1H) ppm; **^{13}C{^{1}H} NMR** (100 MHz, Chloroform-*d*) δ 163.2, 144.9, 143.8, 141.2, 140.5, 135.1, 129.1, 129.0, 128.6, 127.9, 127.6, 127.4, 127.3, 125.4, 120.3, 74.9 ppm; **IR** (thin film): 3363, 3057, 2920, 1627, 1448, 1226, 1040, 833, 740 cm^{-1}; **HRMS** calc'd for $C_{26}H_{20}N^+$ 346.1590, found 346.1590 $[M+H]^+$. **Mp**: 171 – 173 °C.

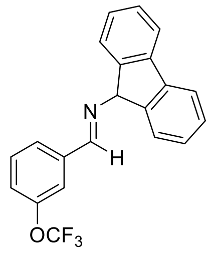

(*E*)-*N*-(9*H*-Fluoren-9-yl)-1-(3-(trifluoromethoxy)phenyl)methanimine (1h). ^{1}H NMR (400 MHz, Chloroform-*d*) δ 8.56 (s, 1H), 7.63 (d, *J* = 7.6 Hz, 2H), 7.56 (d, *J* = 8.0 Hz, 2H), 7.30 – 7.23 (m, 5H), 7.19 – 7.12 (m, 3H), 5.29 (s, 1H) ppm; **^{13}C{^{1}H} NMR** (100 MHz, Chloroform-*d*) δ 161.7, 149.7, 144.5, 141.1, 138.2, 130.1, 128.7, 127.6, 127.0, 125.4, 123.3, 120.6 (q, $J_{C\text{-}F}$ = 256.1 Hz), 120.7, 120.3, 74.5 ppm; **^{19}F NMR** (376 MHz, Chloroform-*d*) δ -57.6 (s, 3F). **IR** (thin film): 3068,

2987, 2853, 1639, 1450, 1166, 1002, 932, 841, 730, 684 cm^{-1}; **HRMS** calc'd for $C_{21}H_{15}F_3NO^+$ 354.1100, found 354.1100 $[M+H]^+$. **Mp**: 73 – 75 ^oC.

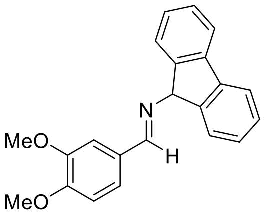

(*E*)-1-(3,4-Dimethoxyphenyl)-*N*-(9*H*-fluoren-9-yl)methanimine (1i). **^{1}H NMR** (400 MHz, Chloroform-*d*) δ 8.58 (s, 1H), 7.67 (s, 1H), 7.66 – 7.65 (m, 1H), 7.38 (s, 1H), 7.33 – 7.29 (m, 4H), 7.23 – 7.15 (m, 3H), 6.80 (t, *J* = 8.0 Hz, 1H), 5.30 (s, 1H), 3.82 (s, 3H) , 3.77 (s, 3H) ppm; **^{13}C{^{1}H} NMR** (100 MHz, Chloroform-*d*) δ 163.1, 151.7, 149.5, 145.1, 141.1, 129.5, 128.5, 127.5, 125.4, 123.6, 120.2, 110.5, 109.4, 74.8, 56.14, 56.07 ppm; **IR** (thin film): 3395, 3065, 2837, 1682, 1420, 1239, 1025, 877, 739 cm^{-1}; **HRMS** calc'd for $C_{22}H_{20}NO_2^+$ 330.1489, found 330.1488 $[M+H]^+$. **Mp**: 154 – 156 ^oC.

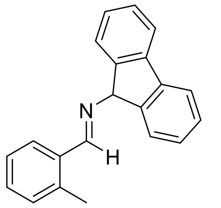

(*E*)-*N*-(9*H*-Fluoren-9-yl)-1-(*o*-tolyl)methanimine (1j). **^{1}H NMR** (400 MHz, Chloroform-*d*) δ 8.97 (s, 1H), 7.81 (dd, *J* = 7.6, 4.0 Hz, 1H), 7.63 (dd, *J* = 8.4, 2.8 Hz, 2H), 7.30 – 7.26 (m, 4H), 7.20 – 7.15 (m, 3H), 7.08 (d, *J* = 7.6 Hz, 2H), 5.28 (s, 1H), 2.46 (s, 3H) ppm; **^{13}C{^{1}H} NMR** (100 MHz, Chloroform-*d*) δ 162.1, 145.0, 141.1, 137.8, 134.2, 130.9, 130.6, 128.5, 128.1, 127.5, 126.3, 125.3, 120.2, 75.3, 19.5 ppm; **IR** (thin film): 3065, 2872, 1630, 1450, 1226, 1157, 1006, 740 cm^{-1}; **HRMS** calc'd for $C_{21}H_{18}N^+$ 284.1434, found 284.1433 $[M+H]^+$. **Mp**: 127 – 129 ^oC.

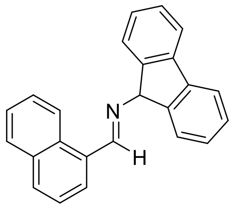

(*E*)-*N*-(9*H*-Fluoren-9-yl)-1-(naphthalen-1-yl)methanimine (1k). **^{1}H NMR** (400 MHz, Chloroform-*d*) δ 9.35 (s, 1H), 8.81 (d, *J* = 7.6 Hz, 1H), 7.90 (dd, *J* = 7.2, 1.2 Hz, 1H), 7.83 (d, *J* = 8.4 Hz, 1H), 7.79 (dd, *J* = 8.4, 1.2 Hz, 1H), 7.69 (d, *J* = 7.6 Hz, 2H), 7.47 – 7.31 (m, 7H), 7.22 (td, *J* = 7.6, 1.2 Hz, 2H), 5.42 (s, 1H) ppm; **^{13}C{^{1}H} NMR** (100 MHz, Chloroform-*d*) δ 163.1, 145.1, 141.2, 133.9, 131.6, 131.5, 129.3, 128.8, 128.6, 127.6, 127.4, 126.2, 125.4, 125.4, 124.5, 120.3, 75.7 ppm, one resonance was not observed due to overlapping peaks; **IR** (thin film): 3383, 3063, 2865, 1682, 1421, 1166, 1018, 742 cm^{-1}; **HRMS** calc'd for $C_{24}H_{18}N^+$ 320.1434, found 320.1433 $[M+H]^+$. **Mp**: 146 – 148 ^oC.

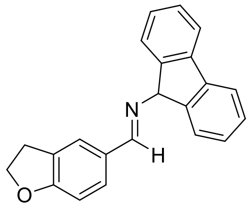

(*E*)-1-(2,3-Dihydrobenzofuran-5-yl)-*N*-(9*H*-fluoren-9-yl)methanimine (1l). **^{1}H NMR** (400 MHz, Chloroform-*d*) δ 8.59 (s, 1H), 7.67 (t, *J* = 8.4 Hz, 3H), 7.41 (dd, *J* = 8.0, 1.6 Hz, 1H), 7.32 – 7.29 (m, 4H), 7.20 (td, *J* = 7.6, 1.2 Hz, 2H), 6.72 (d, *J* = 8.4 Hz, 1H), 5.27 (s, 1H), 4.49 (t, *J* = 8.4 H z, 2H), 3.06 (t, *J* = 8.4 Hz, 2H) ppm; **^{13}C{^{1}H} NMR** (100 MHz, Chloroform-*d*) δ 163.2, 162.9, 145.2, 141.1, 130.6, 129.2, 128.5, 128.1, 127.5, 125.3, 124.6, 120.2, 109.2, 74.7, 72.0, 29.2 ppm; **IR** (thin film): 3359, 3057, 2919, 1627, 1447, 1003, 833, 741, 659 cm^{-1}; **HRMS** calc'd for $C_{22}H_{18}NO^+$ 312.1383, found 312.1387 $[M+H]^+$. **Mp**: 125 – 127 ^oC.

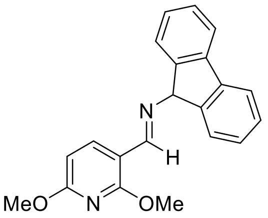

(*E*)-1-(2,6-Dimethoxypyridin-3-yl)-*N*-(9*H*-fluoren-9-yl)methanimine (1 m). **^{1}H NMR** (400 MHz, Chloroform-*d*) δ 8.93 (s, 1H), 8.07 (dd, *J* = 8.0, 1.2 Hz, 1H), 7.61 (dd, *J* = 7.2, 1.2 Hz, 2H), 7.25 (t, *J* = 7.2 Hz, 4H), 7.16 (td, *J* = 7.2, 1.2 Hz, 2H), 6.18 (d, *J* = 8.4 Hz, 1H), 5.23 (s, 1H), 3.91 (s, 3H), 3.81 (s, 3H) ppm; **^{13}C{^{1}H} NMR** (100 MHz, Chloroform-*d*) δ 164.9, 161.8, 158.3,

145.2, 141.0, 139.4, 128.4, 127.4, 125.3, 120.1, 110.8, 102.5, 75.2, 53.8, 53.6 ppm; **IR** (thin film): 3354, 3016, 2866, 1678, 1484, 1155, 1018, 765 cm^{-1}; **HRMS** calc'd for $C_{21}H_{19}N_2O_2^+$ 331.1441, found 331.1442 $[M+H]^+$. **Mp**: 119 – 121 °C.

3. Preparation of alkenyl bromides

Compounds **2a-2d** were purchased from Sigma-Aldrich and directly used. Alkenyl bromides **2e-2h** were prepared according to literature procedures.[3]

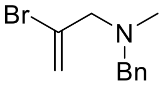

***N*-Benzyl-2-bromo-*N*-methylprop-2-en-1-amine (2e).** The reaction was performed following the literature procedures.[3] Under an air atmosphere, to 2,3-dibromoprop-1-ene (500 mg, 2.5 mmol, 1 equiv) in THF (8.3 mL, 0.3 M) was added *N*-methyl-1-phenylmethanamine (646 µL, 5 mmol, 2 equiv) via syringe at room temperature. The reaction mixture was heated to 35 °C and stirred for 4 h. Once the reaction was complete, H_2O (25 mL) was added and the organics were extracted with CH_2Cl_2 (25 mL). The combined organics were dried over Na_2SO_4, filtered, and concentrated under vacuum. The crude residue was purified by column chromatography (silica gel, 20% to 50% EtOAc in petroleum ether) to afford the title compound as a clear oil (479 mg, 80%). The ^1H and $^{13}C\{^1H\}$ data for this compound match the literature data.[3]

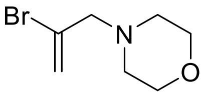

4-(2-Bromoallyl)morpholine (2f). The reaction was performed following the literature procedures.[3] Under an air atmosphere, to 2,3-dibromoprop-1-ene (500 mg, 2.5 mmol, 1 equiv) in THF (8.3 mL, 0.3 M) was added morpholine (432 µL, 5 mmol, 2 equiv) via syringe at room temperature. The reaction mixture was stirred at room temperature for 24 h. Once the reaction was complete, H_2O (25 mL) was added and the organics were extracted with CH_2Cl_2 (25 mL). The combined organics were dried over Na_2SO_4, filtered, and concentrated under vacuum. The crude residue was purified by column chromatography (silica gel, 20-80% EtOAc in petroleum ether) to afford the title compound as a clear oil (368 mg, 71%). The ^1H and $^{13}C\{^1H\}$ data for this compound match the literature data.[3]

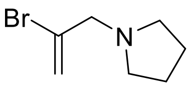

1-(2-Bromoallyl)pyrrolidine (2g). The reaction was performed following the literature procedures.[3] Under an air atmosphere, to 2,3-dibromoprop-1-ene (500 mg, 2.5 mmol, 1 equiv) in THF (8.3 mL, 0.3 M) was added pyrrolidine (411 µL, 5 mmol, 2 equiv) via syringe at room temperature. The reaction mixture was heated to 40 °C and stirred for 24 h. Once the reaction was complete, it was cooled to room temperature and H_2O (25 mL) was added and the organics were extracted with CH_2Cl_2 (25 mL). The combined organics were dried over Na_2SO_4, filtered, and concentrated under vacuum. The crude residue was purified by column chromatography (silica gel, 20-50% EtOAc in petroleum ether) to afford the title compound as a clear oil (404 mg, 85%). **^{1}H NMR** (400 MHz, Chloroform-*d*) δ 5.76 (dd, *J* = 4.4, 1.2 Hz, 1H), 5.45 (d, *J* = 5.6 Hz, 1H), 3.23 (d, *J* = 6.4 Hz, 2H), 2.50 – 2.46 (m, 4H), 1.77 – 1.70 (m, 4H) ppm; **$^{13}C\{^1H\}$ NMR** (100 MHz, Chloroform-*d*) δ 31.6, 117.6, 64.5, 53.7, 23.6 ppm; **IR** (thin film): 2966, 2790, 1631, 1431, 1125, 891, 583 cm^{-1}; **HRMS** calc'd for $C_7H_{13}BrN^+$ 190.0226, found 190.0229 $[M+H]^+$.

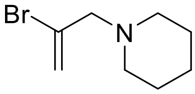

1-(2-Bromoallyl)piperidine (2h). The reaction was performed following the literature procedures.[3] Under an air atmosphere, to 2,3-dibromoprop-1-ene (500 mg, 2.5 mmol, 1 equiv) in THF (8.3 mL, 0.3 M) was added piperidine (501 µL, 5 mmol, 2 equiv) via syringe at room temperature. The reaction mixture was heated to 40 °C for 24 h with

stirring. Once the reaction was complete, it was cooled to room temperature and H_2O (25 mL) was added and the organics were extracted with CH_2Cl_2 (25 mL). The combined organics were dried over Na_2SO_4, filtered, and concentrated under vacuum. The crude residue was purified by column chromatography (silica gel, 20-50% EtOAc in petroleum ether) to afford the title compound as a clear oil (367 mg, 72%). **^{1}H NMR** (400 MHz, Chloroform-*d*) δ 5.77 (d, *J* = 1.6 Hz, 1H), 5.48 (s, 1H), 3.07 (s, 2H), 2.34 (t, *J* = 5.2 Hz, 4H), 1.55 – 1.49 (m, 4H), 1.39 – 1.34 (m, 2H) ppm; **^{13}C{^{1}H} NMR** (100 MHz, Chloroform-*d*) δ 131.2, 118.0, 67.2, 54.2, 26.0, 24.3 ppm; **IR** (thin film): 2935, 2854, 2790, 1630, 1441, 1110, 891, 789, 569 cm^{-1}; **HRMS** calc'd for $C_8H_{15}BrN^+$ 204.0382, found 204.0387 $[M+H]^+$.

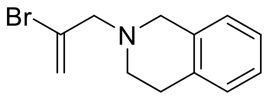

2-(2-Bromoallyl)-1,2,3,4-tetrahydroisoquinoline (2i). Under an air atmosphere, the 1,2,3,4-tetrahydroisoquinoline (313 µL, 2.5 mmol, 1 equiv) and K_2CO_3 (1.04 g, 7.5 mmol, 5 equiv) were added to MeCN (22.5 mL, 0.1 M). Next, 2,3-dibromoprop-1-ene (999 mg, 5 mmol, 2 equiv) was added via syringe at room temperature. The reaction mixture was heated to 40 ^{o}C for 12 h. Once the reaction was complete, it was cooled to room temperature. H_2O (25 mL) was added and the organics were extracted with CH_2Cl_2 (25 mL). The combined organics were dried over Na_2SO_4, filtered, and concentrated under vacuum. The crude residue was purified by column chromatography (silica gel, 2-10% EtOAc in petroleum ether) to afford the title compound as a yellow oil (548 mg, 87%). **^{1}H NMR** (400 MHz, Chloroform-*d*) δ 7.14 – 7.10 (m, 3H), 7.02 – 7.00 (m, 1H), 5.93 (d, *J* = 1.2 Hz, 1H), 5.63 (s, 1H), 3.69 (s, 2H), 3.37 (s, 2H), 2.92 (t, *J* = 6.0 Hz, 2H), 2.80 (t, *J* = 6.0 Hz, 2H) ppm; **^{13}C{^{1}H} NMR** (100 MHz, Chloroform-*d*) δ 134.6, 134.4, 130.8, 128.9, 126.7, 126.3, 125.8, 118.8, 66.1, 55.5, 50.3, 29.1 ppm; **IR** (thin film): 2921, 2803, 1631, 1450, 1093, 896, 741 cm^{-1}; **HRMS** calc'd for $C_{12}H_{15}BrN^+$ 252.0382, found 252.0384 $[M+H]^+$.

4. Ni-catalyzed asymmetric alkenylation of imines (Table 1): ***Lab scale reaction optimization of chiral ligands, solvents, bases, ratio of Ni : ligand and switching from Ni to Pd as metal source***

Table S1. Screening of chiral ligands for Ni-catalyzed asymmetric alkenylation[a]

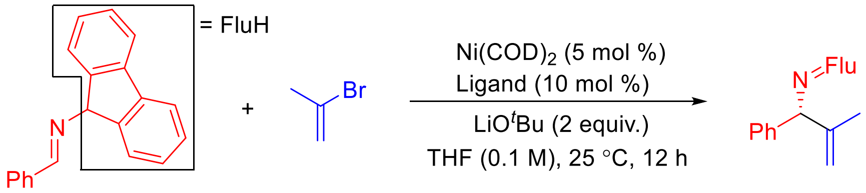

entry	ligand	CAS number	yield of **3aa** (%)[b]	ee of **3aa** (%)[c]
1	**L1**	64896-28-2	65	78
2	**L2**	528565-79-9	52	47
3	**L3**	164858-79-1	47	55
4	**L4**	131457-46-0	66	0
5	**L5**	176706-98-2	75	0
6	**L6**	885701-78-0	34	45
7	**L7**	37002-48-5	70	0
8	**L8**	503538-69-0	28	0
9	**L9**	76189-55-4	77	0
10	**L10**	99646-28-3	82	0
11	**L11**	636559-55-2	33	0
12	**L12**	636559-56-3	55	0
13	**L13**	138517-61-0	5	-
14	**L14**	1439556-82-7	91	0
15	**L15**	244261-66-3	90	0
16	**L16**	138517-62-1	7	-
17	**L17**	301847-89-2	85	0
18	**L18**	136705-65-2	79	0
19	**L19**	934705-43-8	29	0
20	**L20**	712352-08-4	88	0
21	**L21**	157488-65-8	52	0
22	**L22**	349103-24-8	53	0
23	**L23**	1221902-06-2	61	0
24	**L24**	917377-74-3	66	0
25	**L25**	528521-87-1	71	0
26	**L26**	917377-75-4	59	0

[a]Reactions conducted on a 0.1 mmol scale using 1 equiv. of **1a**, and 3 equiv. of **2a**, $Ni(COD)_2$ (5 mol %), ligands (10 mol %) and LiO^{t}Bu (2.0 equiv.) in 1 mL of THF at 25 °C for 12 h. [b]Isolated yield of **3aa** after chromatographic purification. [c]The ee (enantiomeric excess) of **3aa** was determined by chiral phase HPLC.

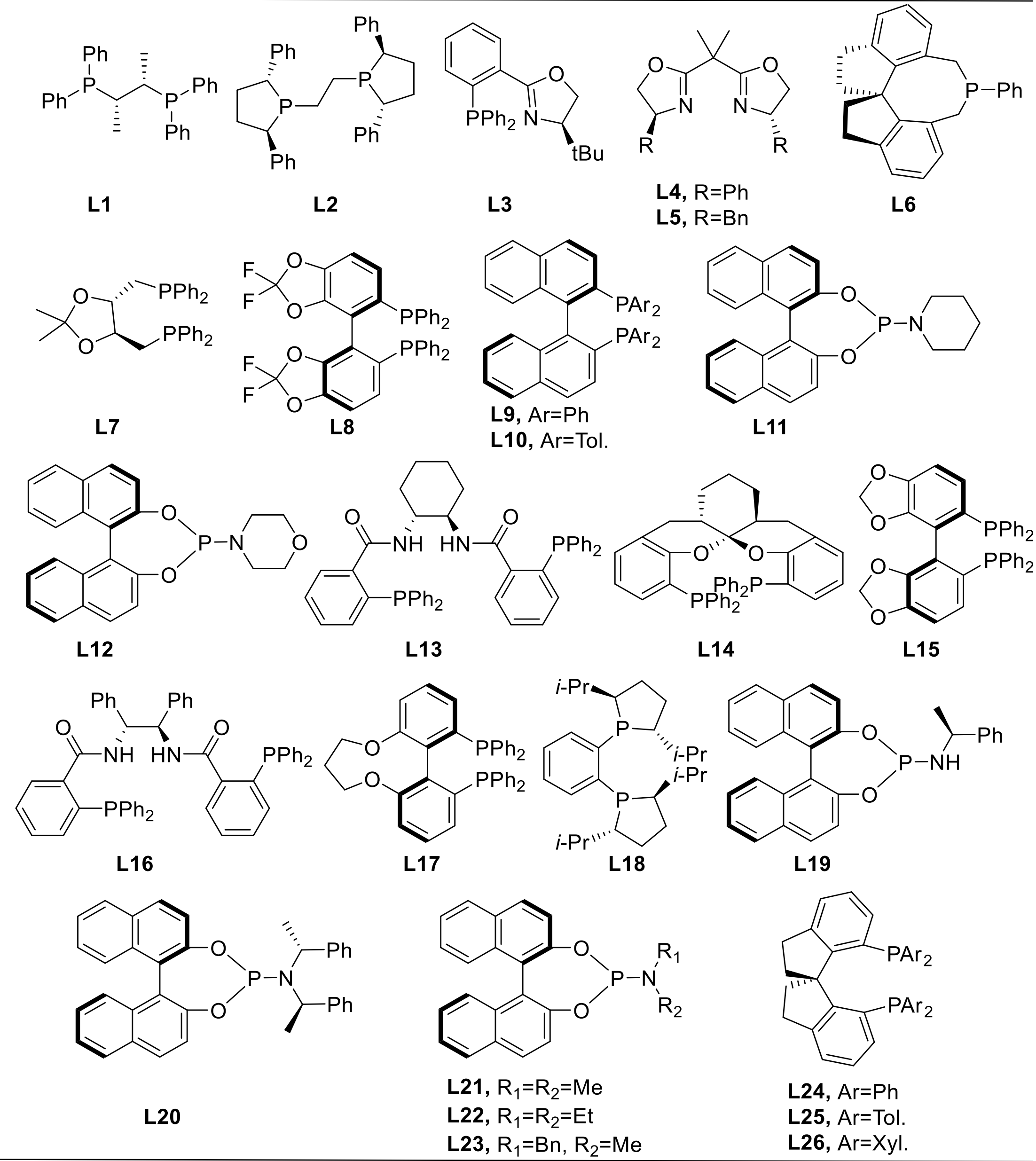

Table S2. Screening of bases for Ni-catalyzed asymmetric alkenylation[a]

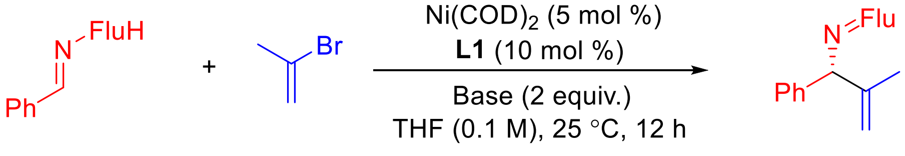

1a **2a** **3aa**

entry	base	yield of **3aa** (%)[b]	ee of **3aa** (%)[c]
1	LiO^{t}Bu	65	78
2	NaO^{t}Bu	23	23
3	KO^{t}Bu	6	-
4	$LiN(SiMe_3)_2$	63	84
5	$NaN(SiMe_3)_2$	94	92
6	$KN(SiMe_3)_2$	42	82

7	Et_3N	0	-
8	DBU	0	-
9	TMG	0	-
10	DABCO	0	-

[a]Reactions conducted on a 0.1 mmol scale using 1 equiv. of **1a**, and 3 equiv. of **2a**, $Ni(COD)_2$ (5 mol %), ligands (10 mol %) and base (2.0 equiv.) in 1 mL of THF at 25 °C for 12 h. [b]Isolated yield of **3aa** after chromatographic purification. [c]The ee (enantiomeric excess) of **3aa** was determined by chiral phase HPLC.

Table S3. Screening of solvents for Ni-catalyzed asymmetric alkenylation[a]

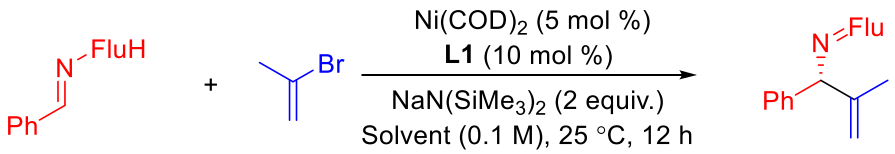

1a **2a** **3aa**

entry	Solvent	yield of **3aa** (%)[b]	ee of **3aa** (%)[c]
1	THF	94	92
2	DME	4	-
3	CPME	62	48
4	MTBE	32	20
5	Cyclohexane	5	-
6	Toluene	0	-
7	Et_2O	32	14
8	1,4-Dioxane	4	-

[a]Reactions conducted on a 0.1 mmol scale using 1 equiv. of **1a**, and 3 equiv. of **2a**, $Ni(COD)_2$ (5 mol %), ligands (10 mol %) and $NaN(SiMe_3)_2$ (2.0 equiv.) in 1 mL of solvent at 25 °C for 12 h. [b]Isolated yield of **3aa** after chromatographic purification. [c]The ee (enantiomeric excess) of **3aa** was determined by chiral phase HPLC.

Table S4. Further optimization for Ni-catalyzed asymmetric alkenylation[a]

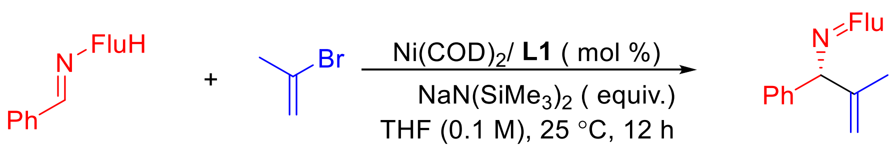

1a **2a** **3aa**

entry	$Ni(COD)_2$/ **L1** (mol %)	$NaN(SiMe_3)_2$ (equiv.)	yield of **3aa** (%)[b]	ee of **3aa** (%)[c]
1	5/10	2	94	92
2	2.5/5	2	62	92
3	5/10	1.5	95	93

[a]Reactions conducted on a 0.1 mmol scale using 1 equiv. of **1a**, and 3 equiv. of **2a**, $Ni(COD)_2$, **L1** and $NaN(SiMe_3)_2$ in 1 mL of THF at 25 °C for 12 h. [b]Isolated yield of **3aa** after chromatographic purification. [c]The ee (enantiomeric excess) of **3aa** was determined by chiral phase HPLC.

Table S5. Switching Ni to Pd as metal sources[a]

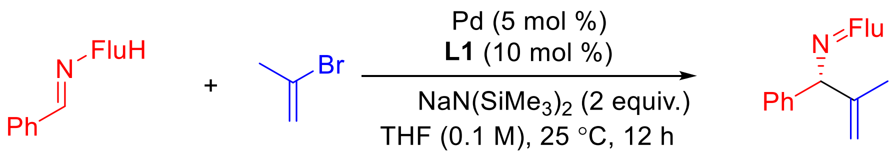

1a **2a** **3aa**

entry	Pd	yield of **3aa** (%)[b]	ee of **3aa** (%)
1	$Pd(OAc)_2$	4	-
2	$[PdCl(allyl)]_2$	0	-
3	$Pd(dba)_2$	0	-
4	$Pd(COD)Cl_2$	0	-

[a]Reactions conducted on a 0.1 mmol scale using 1 equiv. of **1a**, and 3 equiv. of **2a**, Pd (5 mol %), **L1** (10 mol %) and $NaN(SiMe_3)_2$ (1.5 equiv.) in 1 mL of THF at 25 °C for 12 h. [b]Isolated yield of **3aa** after chromatographic purification.

5. General procedure and characterization of Ni-catalyzed asymmetric alkenylation of imines with alkenyl bromides (Tables 2 and 3)

General procedure for the Ni-catalyzed asymmetric alkenylation of imines with alkenyl bromides:
An oven-dried 8 mL reaction vial equipped with a stir bar was charged with imines (**1**, 0.4 mmol, 1.0 equiv) and vinyl halides (**2**, 1.2 mmol, 3.0 equiv) in a glove box under a nitrogen atmosphere at room temperature. A stock solution containing $Ni(COD)_2$ (13.8 mg, 0.02 mmol, 5 mol %) and **L1** (17.0 mg, 0.04 mmol, 10 mol %) in 2 mL of dry THF was taken up by syringe and added to the reaction vial under nitrogen. Then, $NaN(SiMe_3)_2$ (110.0 mg, 0.6 mmol, 1.5 equiv) in 2 mL of dry THF was added to the reaction mixture. The vial was capped, removed from the glove box, and stirred for 12 h at 25 °C until TLC showed complete consumption of the imine. The reaction mixture was quenched with three drops of H_2O, diluted with 3 mL of ethyl acetate, and filtered over a pad of $MgSO_4$ and silica. The pad was rinsed with ethyl acetate (3X2 mL) and the combined solutions were concentrated in *vacuo*. The crude material was loaded onto a silica gel column and purified by flash chromatography.

General workup procedure for the Ni-catalyzed asymmetric alkenylation of imines with alkenyl bromides (for 3ag, 3ah, 4ka, 4la):
After the reaction was complete, 1 M aq. HCl (0.5 mL) was added to the reaction mixture. The resulting mixture was left at room temperature until the imine products were fully consumed, and then diluted with H_2O (2 mL). The mixture was concentrated to remove THF and extracted with Et_2O (5 mL) to remove fluorenone. The aqueous layer was basified with NaOH (1 M) to pH > 9 and extracted with CH_2Cl_2. To the combined organic layers was added Et_3N (100 μL) and Boc_2O (100 μL) or 4-methylbenzenesulfonyl chloride (95 mg). The mixture was left at 25 °C until full consumption of the primary amine was indicated by TLC analysis. The mixture was concentrated and subjected to flash chromatography to give the corresponding carbamates or sulfonamides.

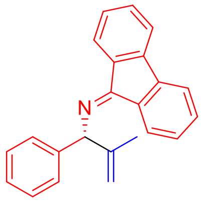

(*R*)-*N*-(2-Methyl-1-phenylallyl)-9*H*-fluoren-9-imine (3aa): The reaction was performed following the general procedure with (*E*)-*N*-(9*H*-fluoren-9-yl)-1-phenylmethanimine **1a** (107.7 mg, 0.4 mmol) and 2-bromoprop-1-ene **2a** (145.2 mg, 1.2 mmol). The crude product was separated by flash chromatography on deactivated silica gel (hexanes to hexanes:ethyl acetate = 100:1). Further purification was performed on an Agilent HPLC 1260 system using acetonitrile:H_2O (90:10 vol./vol.) as mobile phase and flow rate of 4 mL/min with monitoring at 254 nm to give the product **3aa** (117.6 mg, 95% yield, 93% *ee*) as a yellow oil. $\mathbf{R}_f$ = 0.50 (hexanes:ethyl acetate = 10:1). **^{1}H NMR** (400 MHz, Chloroform-*d*) δ 7.85 (d, *J* = 7.2 Hz, 1H), 7.64 (d, *J* = 7.6 Hz, 1H), 7.51 (d, *J* = 7.2 Hz, 1H), 7.45 – 7.40 (m, 3H), 7.30 – 7.19 (m, 5H), 7.16 – 7.13 (m, 1H), 7.09 (dd, *J* = 7.6, 1.2 Hz, 1H), 5.98 (s, 1H), 5.06 (s, 1H), 4.84 (s, 1H), 1.74 (s, 3H) ppm; **^{13}C{^{1}H} NMR** (100 MHz, Chloroform-*d*) δ 162.5, 147.6, 144.1, 142.6, 141.1, 139.0, 131.7, 131.3, 131.0, 128.5, 128.4, 128.0, 127.7, 127.5, 127.1, 123.1, 120.4, 119.3, 112.0, 71.0, 19.3 ppm; **IR** (thin film): 3059, 2962, 1643, 1599, 1491, 1318, 1028, 792, 729 cm^{-1}; **HRMS** calc'd for $C_{23}H_{20}N^+$ 310.1590, found 310.1589 $[M+H]^+$; **HPLC analysis:** Daicel CHIRALPAK OJ-H, *n*-hexane/*i*-PrOH = 75/25, flow rate = 0.3 mL/min, λ = 250 nm, retention time: t_{major} = 19.72 min, t_{minor} = 24.71 min; $[\alpha]_D^{20}$ = -16.17 (c 1.0, $CHCl_3$).

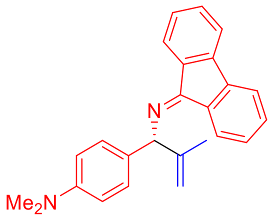

(*R*)-4-(1-((9*H*-Fluoren-9-ylidene)amino)-2-methylallyl)-*N*,*N*-dimethylaniline (3ba): The reaction was performed following the general procedure with (*E*)-4-(((9*H*-fluoren-9-yl)imino)methyl)-*N*,*N*-dimethylaniline **1b** (125.0 mg, 0.4 mmol) and 2-bromoprop-1-ene **2a** (145.2 mg, 1.2 mmol). The crude product was separated by flash chromatography on deactivated silica gel (hexanes to hexanes:ethyl acetate = 30:1). Further purification was performed on an Agilent HPLC 1260 system using acetonitrile:H_2O (90:10 vol./vol.) as mobile phase and flow rate of 4 mL/min with monitoring at 254 nm to give the product **3ba** (135.4 mg, 96% yield, 91% *ee*) as a yellow oil. **R*f*** = 0.36 (hexanes:ethyl acetate = 10:1). **^{1}H NMR** (400 MHz, Chloroform-*d*) δ 7.85 (d, *J* = 7.6 Hz, 1H), 7.69 (d, *J* = 7.6 Hz, 1H), 7.52 (d, *J* = 7.6 Hz, 1H), 7.45 (d, *J* = 7.2 Hz, 1H), 7.29 (dd, *J* = 7.6, 1.2 Hz, 1H), 7.26 – 7.23 (m, 3H), 7.20 (td, *J* = 7.6, 1.2 Hz, 1H), 7.11 (td, *J* = 7.6, 1.2 Hz, 1H), 6.62 (d, *J* = 4.4 Hz, 2H), 5.91 (s, 1H), 5.03 (s, 1H), 4.82 (s, 1H), 2.81 (s, 6H), 1.77 (s, 3H) ppm; **^{13}C{^{1}H} NMR** (100 MHz, Chloroform-*d*) δ 161.9, 149.8, 148.1, 144.0, 141.0, 139.2, 131.8, 131.1, 130.8, 130.5, 128.3, 128.2, 128.0, 127.9, 123.1, 120.3, 119.3, 112.8, 111.3, 70.5, 40.8, 19.4 ppm; **IR** (thin film): 3058, 2967, 1644, 1458, 1275, 1110, 1033, 899, 764, 673 cm^{-1}; **HRMS** calc'd for $C_{25}H_{25}N_2^+$ 353.2012, found 353.2018 [M+H]$^+$; **HPLC analysis:** Daicel CHIRALPAK AD-H, *n*-hexane/*i*-PrOH = 95/5, flow rate = 0.5 mL/min, λ = 250 nm, retention time: t_{major} = 10.59 min, t_{minor} = 11.46 min; $[\alpha]_D^{20}$ = +44.81 (c 1.0, $CHCl_3$).

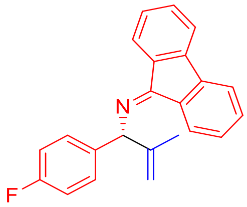

(*R*)-*N*-(1-(4-Fluorophenyl)-2-methylallyl)-9*H*-fluoren-9-imine (3ca): The reaction was performed following the general procedure with (*E*)-*N*-(9*H*-fluoren-9-yl)-1-(4-fluorophenyl)methanimine **1c** (114.9 mg, 0.4 mmol) and 2-bromoprop-1-ene **2a** (145.2 mg, 1.2 mmol). The crude product was separated by flash chromatography on deactivated silica gel (hexanes to hexanes:ethyl acetate = 100:1). Further purification was performed on an Agilent HPLC 1260 system using acetonitrile:H_2O (90:10 vol./vol.) as mobile phase and flow rate of 4 mL/min with monitoring at 254 nm to give the product **3ca** (119.2 mg, 91% yield, 90% *ee*) as a yellow oil. **R*f*** = 0.49 (hexanes:ethyl acetate = 10:1). **^{1}H NMR** (400 MHz, Chloroform-*d*) δ 7.85 (d, *J* = 7.6 Hz, 1H), 7.63 (d, *J* = 8.0 Hz, 1H), 7.55 (d, *J* = 7.6 Hz, 1H), 7.47 (d, *J* = 7.6 Hz, 1H), 7.42 – 7.37 (m, 2H), 7.33 – 7.28 (m, 2H), 7.22 (td, *J* = 7.2, 1.2 Hz, 1H), 7.13 (td, *J* = 7.6, 1.2 Hz, 1H), 6.97 – 6.91 (m, 2H), 5.95 (s, 1H), 5.04 (s, 1H), 4.85 (s, 1H), 1.73 (s, 3H) ppm; **^{13}C{^{1}H} NMR** (100 MHz, Chloroform-*d*) δ 162.7, 162.0 (d, $^1J_{C-F}$ = 243.4 Hz), 147.2, 144.2, 141.1, 138.9, 138.3 (d, $^4J_{C-F}$ = 1.4 Hz), 131.6, 131.5, 131.2, 129.1 (d, $^3J_{C-F}$ = 7.8 Hz), 128.5, 128.1, 127.7, 123.1, 120.5, 119.4, 115.3 (d, $^2J_{C-F}$ = 21.0 Hz), 112.2, 70.2, 19.3 ppm; **^{19}F NMR** (376 MHz, Chloroform-*d*) δ -116.0 (s, 1F). **IR** (thin film): 3054, 2987, 1644, 1505, 1449, 1275, 1095, 751, 654 cm^{-1}; **HRMS** calc'd for $C_{23}H_{19}FN^+$ 328.1496, found 328.1495 [M+H]$^+$; **HPLC analysis:** Daicel CHIRALPAK AD-H, *n*-hexane/*i*-PrOH = 85/15, flow rate = 0.3 mL/min, λ = 250 nm, retention time: t_{major} = 13.69 min, t_{minor} = 15.22 min; $[\alpha]_D^{20}$ = -14.85 (c 1.0, $CHCl_3$).

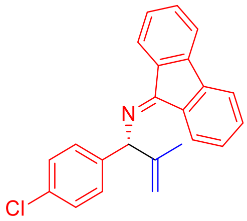

(*R*)-*N*-(1-(4-Chlorophenyl)-2-methylallyl)-9*H*-fluoren-9-imine (3da): The reaction was performed following the general procedure with (*E*)-1-(4-chlorophenyl)-*N*-(9*H*-fluoren-9-yl)methanimine **1d** (121.5 mg, 0.4 mmol) and 2-bromoprop-1-ene **2a** (145.2 mg, 1.2 mmol). The crude product was separated by flash chromatography on deactivated silica gel (hexanes to hexanes:ethyl acetate = 100:1). Further purification was performed on an Agilent HPLC 1260

system using acetonitrile:H_2O (90:10 vol./vol.) as mobile phase and flow rate of 4 mL/min with monitoring at 254 nm to give the product **3da** (86.7 mg, 63% yield, 85% *ee*) as a yellow oil. **R*f*** = 0.47 (hexanes:ethyl acetate = 10:1). **^{1}H NMR** (400 MHz, Chloroform-*d*) δ 7.82 (d, *J* = 7.6 Hz, 1H), 7.59 (d, *J* = 7.6 Hz, 1H), 7.51 (d, *J* = 7.2 Hz, 1H), 7.43 (d, *J* = 7.6 Hz, 1H), 7.36 – 7.34 (m, 2H), 7.29 (dd, *J* = 7.2, 1.2 Hz, 1H), 7.25 (dd, *J* = 7.6, 1.2 Hz, 1H), 7.22 – 7.17 (m, 3H), 7.10 (td, *J* = 7.6, 1.2 Hz, 1H), 5.92 (s, 1H), 5.03 (s, 1H), 4.83 (s, 1H), 1.71 (s, 3H) ppm; **^{13}C{^{1}H} NMR** (100 MHz, Chloroform-*d*) δ 163.1, 147.4, 144.5, 141.5, 141.4, 139.2, 133.1, 131.9, 131.8, 131.5, 129.2, 128.9, 128.8, 128.4, 127.9, 123.4, 120.8, 119.7, 112.7, 70.6, 19.5 ppm; **IR** (thin film): 3394, 3061, 2873, 1644, 1275, 1090, 913, 764, 654 cm^{-1}; **HRMS** calc'd for $C_{23}H_{19}ClN^+$ 344.1201, found 344.1203 $[M+H]^+$; **HPLC analysis:** Daicel CHIRALPAK OJ-H, *n*-hexane/*i*-PrOH = 75/25, flow rate = 0.3 mL/min, λ = 250 nm, retention time: t_{major} = 16.09 min, t_{minor} = 17.69 min; $[\alpha]_D^{20}$ = -9.28 (c 1.0, $CHCl_3$).

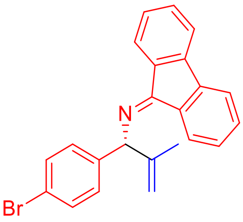

(*R*)-*N*-(1-(4-Bromophenyl)-2-methylallyl)-9*H*-fluoren-9-imine (3ea): The reaction was performed following the general procedure with (*E*)-1-(4-bromophenyl)-*N*-(9*H*-fluoren-9-yl)methanimine **1e** (139.3 mg, 0.4 mmol) and 2-bromoprop-1-ene **2a** (145.2 mg, 1.2 mmol). The crude product was separated by flash chromatography on deactivated silica gel (hexanes to hexanes:ethyl acetate = 100:1). Further purification was performed on an Agilent HPLC 1260 system using acetonitrile:H_2O (85:15 vol./vol.) as mobile phase and flow rate of 4 mL/min with monitoring at 254 nm to give the product **3ea** (127.4 mg, 82% yield, 85% *ee*) as a yellow oil. **R*f*** = 0.41 (hexanes:ethyl acetate = 10:1). **^{1}H NMR** (400 MHz, Chloroform-*d*) δ 7.83 (d, *J* = 7.6 Hz, 1H), 7.60 (d, *J* = 7.6 Hz, 1H), 7.53 (d, *J* = 7.6 Hz, 1H), 7.46 (d, *J* = 7.6 Hz, 1H), 7.38 – 7.35 (m, 2H), 7.32 – 7.27 (m, 4H), 7.21 (td, *J* = 7.6, 1.2 Hz, 1H), 7.12 (td, *J* = 7.6, 1.2 Hz, 1H), 5.92 (s, 1H), 5.04 (s, 1H), 4.85 (s, 1H), 1.72 (s, 3H) ppm; **^{13}C{^{1}H} NMR** (100 MHz, Chloroform-*d*) δ 162.8, 145.0, 144.2, 141.7, 141.1, 138.9, 131.6, 131.5, 131.2, 129.3, 128.5, 128.1, 127.6, 123.1, 121.0, 120.5, 119.4, 112.4, 70.3, 19.2 ppm, one resonance was not observed due to overlapping peaks; **IR** (thin film): 3061, 2916, 2359, 1644, 1449, 1101, 913, 764, 633 cm^{-1}; **HRMS** calc'd for $C_{23}H_{19}BrN^+$ 388.0695, found 388.0692 $[M+H]^+$; **HPLC analysis:** Daicel CHIRALPAK AD-H, *n*-hexane/*i*-PrOH = 85/15, flow rate = 0.3 mL/min, λ = 250 nm, retention time: t_{major} = 14.37 min, t_{minor} = 16.59 min; $[\alpha]_D^{20}$ = -45.77 (c 1.0, $CHCl_3$).

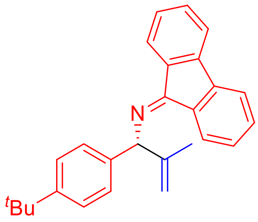

(*R*)-*N*-(1-(4-(*tert*-Butyl)phenyl)-2-methylallyl)-9*H*-fluoren-9-imine (3fa): The reaction was performed following the general procedure with (*E*)-1-(4-(*tert*-butyl)phenyl)-*N*-(9*H*-fluoren-9-yl)methanimine **1f** (130.2 mg, 0.4 mmol) and 2-bromoprop-1-ene **2a** (145.2 mg, 1.2 mmol). The crude product was separated by flash chromatography on deactivated silica gel (hexanes to hexanes:ethyl acetate = 100:1). Further purification was performed on an Agilent HPLC 1260 system using acetonitrile:H_2O (90:10 vol./vol.) as mobile phase and flow rate of 4 mL/min with monitoring at 254 nm to give the product **3fa** (128.7 mg, 88% yield, 92% *ee*) as a yellow oil. **R*f*** = 0.50 (hexanes:ethyl acetate = 10:1). **^{1}H NMR** (400 MHz, Chloroform-*d*) δ 7.86 (d, *J* = 7.2 Hz, 1H), 7.71 (d, *J* = 7.6 Hz, 1H), 7.55 (d, *J* = 7.2 Hz, 1H), 7.47 (d, *J* = 7.2 Hz, 1H), 7.36 – 7.26 (m, 6H), 7.21 (td, *J* = 7.2, 0.8 Hz, 1H), 7.14 (td, *J* = 7.2, 0.8 Hz, 1H), 5.98 (s, 1H), 5.06 (s, 1H), 4.84 (s, 1H), 1.76 (s, 3H), 1.22 (s, 9H) ppm; **^{13}C{^{1}H} NMR** (100 MHz, Chloroform-*d*) δ 162.2, 149.9, 147.6, 144.2, 141.1, 131.8, 131.3, 131.0, 128.4, 128.0, 127.8, 127.2, 125.4, 123.1, 120.4, 119.3, 111.8, 70.6, 34.6, 31.5, 19.5 ppm, two resonances were not observed due to overlapping peaks; **IR** (thin film): 3058 2963, 1644, 1449, 1364,

1269, 1102, 898, 792, 653 cm^{-1}; **HRMS** calc'd for $C_{27}H_{28}N^+$ 366.2216, found 366.2213 $[M+H]^+$; **HPLC analysis:** Daicel CHIRALPAK OD-H, *n*-hexane/*i*-PrOH = 95/5, flow rate = 0.5 mL/min, λ = 250 nm, retention time: t_{major} = 11.68 min, t_{minor} = 14.95 min; $[\alpha]_D^{20}$ = +17.38 (c 1.0, $CHCl_3$).

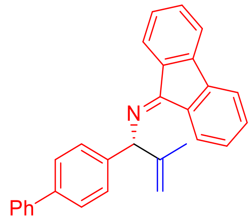

(*R*)-*N*-(1-([1,1'-Biphenyl]-4-yl)-2-methylallyl)-9*H*-fluoren-9-imine (3ga): The reaction was performed following the general procedure with (*E*)-1-([1,1'-biphenyl]-4-yl)-*N*-(9*H*-fluoren-9-yl)methanimine **1g** (138.2 mg, 0.4 mmol) and 2-bromoprop-1-ene **2a** (145.2 mg, 1.2 mmol). The crude product was separated by flash chromatography on deactivated silica gel (hexanes to hexanes:ethyl acetate = 100:1). Further purification was performed on an Agilent HPLC 1260 system using acetonitrile:H_2O (90:10 vol./vol.) as mobile phase and flow rate of 4 mL/min with monitoring at 254 nm to give the product **3ga** (141.9 mg, 92% yield, 90% *ee*) as a yellow oil. $\mathbf{R_f}$ = 0.47 (hexanes:ethyl acetate = 10:1). **^{1}H NMR** (400 MHz, Chloroform-*d*) δ 7.88 (d, *J* = 7.2 Hz, 1H), 7.69 (d, *J* = 7.6 Hz, 1H), 7.54 (d, *J* = 7.6 Hz, 1H), 7.50 – 7.46 (m, 7H), 7.34 – 7.27 (m, 4H), 7.22 (td, *J* = 7.2, 1.2 Hz, 2H), 7.13 (td, *J* = 7.6, 1.2 Hz, 1H), 6.03 (s, 1H), 5.10 (s, 1H), 4.88 (s, 1H), 1.79 (s, 3H) ppm; **^{13}C{^{1}H} NMR** (100 MHz, Chloroform-*d*) δ 162.6, 147.5, 144.2, 141.7, 141.1, 140.0, 139.0, 131.7, 131.4, 131.1, 128.8, 128.4, 128.1, 128.0, 127.8, 127.3, 127.2, 123.1, 120.5, 119.4, 112.1, 70.7, 19.4 ppm, two resonances were not observed due to overlapping peaks; **IR** (thin film): 3059, 2970, 1648, 1449, 1219, 1014, 849, 772, 654 cm^{-1}; **HRMS** calc'd for $C_{29}H_{24}N^+$ 386.1903, found 386.1903 $[M+H]^+$; **HPLC analysis:** Daicel CHIRALPAK OJ-H, *n*-hexane/*i*-PrOH = 70/30, flow rate = 0.25 mL/min, λ = 250 nm, retention time: t_{major} = 32.10 min, t_{minor} = 28.92 min; $[\alpha]_D^{20}$ = -11.18 (c 1.0, $CHCl_3$).

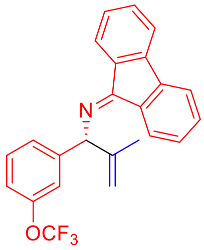

(*R*)-*N*-(2-Methyl-1-(3-(trifluoromethoxy)phenyl)allyl)-9*H*-fluoren-9-imine (3ha): The reaction was performed following the general procedure with (*E*)-*N*-(9*H*-fluoren-9-yl)-1-(3-(trifluoromethoxy)phenyl)methanimine **1h** (141.3 mg, 0.4 mmol) and 2-bromoprop-1-ene **2a** (145.2 mg, 1.2 mmol). The crude product was separated by flash chromatography on deactivated silica gel (hexanes to hexanes:ethyl acetate = 100:1). Further purification was performed on an Agilent HPLC 1260 system using acetonitrile:H_2O (90:10 vol./vol.) as mobile phase and flow rate of 4 mL/min with monitoring at 254 nm to give the product **3ha** (122.7 mg, 78% yield, 90% *ee*) as a yellow oil. $\mathbf{R_f}$ = 0.47 (hexanes:ethyl acetate = 10:1). **^{1}H NMR** (400 MHz, Chloroform-*d*) δ 7.85 (d, *J* = 7.6 Hz, 1H), 7.65 (d, *J* = 7.6 Hz, 1H), 7.56 (d, *J* = 7.2 Hz, 1H), 7.48 (d, *J* = 7.6 Hz, 1H), 7.38 – 7.23 (m, 6H), 7.17 – 7.13 (m, 1H), 7.03 (d, *J* = 8.0 Hz, 1H), 5.98 (s, 1H), 5.07 (s, 1H), 4.87 (s, 1H), 1.73 (s, 3H) ppm; **^{13}C{^{1}H} NMR** (100 MHz, Chloroform-*d*) δ 163.0, 149.6, 146.7, 145.3, 144.3, 141.1, 138.9, 131.64, 131.58, 131.2, 129.7, 128.5, 128.1, 127.7, 126.0, 123.1, 120.6, 120.7 (q, J_{C-F} = 255.2 Hz), 120.3, 119.4, 112.7, 70.3, 19.2 ppm, one resonance was not observed due to overlapping peaks; **^{19}F NMR** (376 MHz, Chloroform-*d*) δ -57.6 (s, 3F). **IR** (thin film): 3056, 2923, 1651, 1450, 1213, 1102, 913, 742, 654 cm^{-1}; **HRMS** calc'd for $C_{24}H_{19}F_3NO^+$ 394.1413, found 394.1413 $[M+H]^+$; **HPLC analysis:** Daicel CHIRALPAK OJ-H, *n*-hexane/*i*-PrOH = 98/2, flow rate = 0.5 mL/min, λ = 260 nm, retention time: t_{major} = 10.87 min, t_{minor} = 13.14 min; $[\alpha]_D^{20}$ = -17.08 (c 1.0, $CHCl_3$).

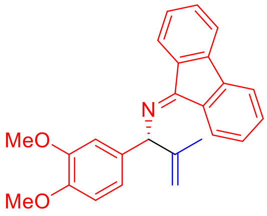

(*R*)-*N*-(1-(3,4-Dimethoxyphenyl)-2-methylallyl)-9*H*-fluoren-9-imine (3ia): The reaction was performed following the general procedure with (*E*)-1-(3,4-dimethoxyphenyl)-*N*-(9*H*-fluoren-9-yl)methanimine **1i** (131.8 mg, 0.4 mmol) and 2-bromoprop-1-ene **2a** (145.2 mg, 1.2 mmol). The crude product was separated by flash chromatography on deactivated silica gel (hexanes to hexanes:ethyl acetate = 15:1). Further purification was performed on an Agilent HPLC 1260 system using acetonitrile:H_2O (80:20 vol./vol.) as mobile phase and flow rate of 4 mL/min with monitoring at 254 nm to give the product **3ia** (141.9 mg, 96% yield, 95% *ee*) as a yellow oil. **R_f** = 0.30 (hexanes:ethyl acetate = 10:1). **^{1}H NMR** (400 MHz, Chloroform-*d*) δ 7.85 (d, *J* = 7.6 Hz, 1H), 7.67 (d, *J* = 8.0 Hz, 1H), 7.54 (d, *J* = 7.6 Hz, 1H), 7.47 (d, *J* = 7.2 Hz, 1H), 7.33 – 7.28 (m, 2H), 7.21 (t, *J* = 7.2 Hz, 1H), 7.13 (t, *J* = 7.6 Hz, 1H), 7.00 (s, 1H), 6.92 (d, *J* = 8.4 Hz, 1H), 6.73 (dd, *J* = 8.4, 2.0 Hz, 1H), 5.91 (s, 1H), 5.03 (s, 1H), 4.85 (s, 1H), 3.80 (s, 3H), 3.76 (s, 3H), 1.76 (s, 3H) ppm; **^{13}C{^{1}H} NMR** (100 MHz, Chloroform-*d*) δ 162.5, 149.1, 148.2, 147.5, 144.1, 141.1, 139.0, 135.2, 131.7, 131.3, 131.0, 128.4, 128.0, 127.8, 123.0, 120.4, 119.7, 119.3, 111.8, 111.0, 110.8, 70.6, 56.02, 55.98, 19.4 ppm; **IR** (thin film): 3053, 2976, 2253, 1643, 1449, 1376, 1269, 1048, 880, 760, 657 cm^{-1}; **HRMS** calc'd for $C_{25}H_{24}NO_2^+$ 370.1802, found 370.1802 $[M+H]^+$; **HPLC analysis:** Daicel CHIRALPAK AD-H, *n*-hexane/*i*-PrOH = 98/2, flow rate = 1.0 mL/min, λ = 254 nm, retention time: t_{major} = 17.23 min, t_{minor} = 18.93 min; $[\alpha]_D^{20}$ = +3.99 (c 1.0, $CHCl_3$).

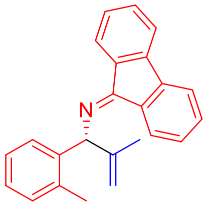

(*R*)-*N*-(2-Methyl-1-(*o*-tolyl)allyl)-9*H*-fluoren-9-imine (3ja): The reaction was performed following the general procedure with (*E*)-*N*-(9*H*-fluoren-9-yl)- 1-(*o*-tolyl)methanimine **1j** (113.4 mg, 0.4 mmol) and 2-bromoprop-1-ene **2a** (145.2 mg, 1.2 mmol). The crude product was separated by flash chromatography on deactivated silica gel (hexanes to hexanes:ethyl acetate = 100:1). Further purification was performed on an Agilent HPLC 1260 system using acetonitrile:H_2O (75:25 vol./vol.) as mobile phase and flow rate of 4 mL/min with monitoring at 254 nm to give the product **3ja** (108.7 mg, 84% yield, 92% *ee*) as a yellow oil. **R_f** = 0.47 (hexanes:ethyl acetate = 10:1). **^{1}H NMR** (400 MHz, Cmhloroform-*d*) δ 7.85 (d, *J* = 7.6 Hz, 1H), 7.57 (d, *J* = 5.6 Hz, 1H), 7.55 (d, *J* = 4.4 Hz, 1H), 7.50 (d, *J* = 3.6 Hz, 1H), 7.48 (d, *J* = 3.2 Hz, 1H), 7.33 – 7.28 (m, 2H), 7.22 (d, *J* = 7.2, 1.2 Hz, 1H), 7.15 (dd, *J* = 7.6, 1.2 Hz, 1H), 7.12 – 7.07 (m, 3H), 6.08 (s, 1H), 4.89 – 4.88 (m, 2H), 2.37 (s, 3H), 1.76 (s, 3H) ppm; **^{13}C{^{1}H} NMR** (100 MHz, Chloroform-*d*) δ 162.5, 145.9, 144.2, 141.1, 140.7, 139.0, 136.0, 131.6, 131.3, 131.0, 130.7, 128.4, 128.1, 128.0, 127.2, 127.0, 126.3, 123.2, 120.5, 119.3, 112.6, 68.0, 20.2, 19.8 ppm; **IR** (thin film): 3055, 2928, 1633, 1488, 1221, 1138, 1008, 945, 772, 656 cm^{-1}; **HRMS** calc'd for $C_{24}H_{22}N^+$ 324.1747, found 324.1748 $[M+H]^+$; **HPLC analysis:** Daicel CHIRALPAK AD-H, *n*-hexane/*i*-PrOH = 85/15, flow rate = 0.3 mL/min, λ = 250 nm, retention time: t_{major} = 14.67 min, t_{minor} = 17.00 min; $[\alpha]_D^{20}$ = -60.02 (c 1.0, $CHCl_3$).

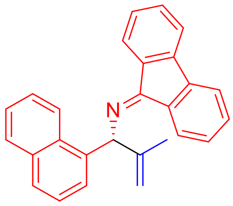

(*R*)-*N*-(2-Methyl-1-(naphthalen-1-yl)allyl)-9*H*-fluoren-9-imine (3ka): The reaction was performed following the general procedure with (*E*)-*N*-(9*H*-fluoren-9-yl)-1-(naphthalen-2-yl)methanimine **1k** (127.8 mg, 0.4 mmol) and 2-bromoprop-1-ene **2a** (145.2 mg, 1.2 mmol). The crude product was separated by flash chromatography on deactivated silica gel (hexanes to hexanes:ethyl acetate = 100:1). Further purification was performed on an Agilent HPLC 1260 system using acetonitrile:H_2O (70:30 vol./vol.) as mobile phase and flow rate of 2.5 mL/min with monitoring at 254 nm to give the

product **3ka** (120.8 mg, 84% yield, 92% *ee*) as a yellow solid. **Mp**: 167 – 169 °C. **R*f*** = 0.48 (hexanes:ethyl acetate = 10:1). **^{1}H NMR** (400 MHz, Chloroform-*d*) δ 7.91 (d, *J* = 7.6 Hz, 1H), 7.91 (d, *J* = 6.4 Hz, 1H), 7.75 – 7.70 (m, 3H), 7.67 (d, *J* = 7.6 Hz, 1H), 7.61 (dd, *J* = 8.4, 2.0 Hz, 1H), 7.54 (d, *J* = 7.6 Hz, 1H), 7.48 (d, *J* = 7.2 Hz, 1H), 7.37 – 7.22 (m, 5H), 7.14 – 7.08 (m, 1H), 6.13 (s, 1H), 5.12 (s, 1H), 4.90 (s, 1H), 1.78 (s, 3H) ppm; **^{13}C{^{1}H} NMR** (100 MHz, Chloroform-*d*) δ 162.8, 147.4, 144.2, 141.2, 140.0, 139.0, 133.5, 132.9, 131.7, 131.4, 131.1, 128.4, 128.3, 128.13, 128.08, 127.8, 127.8, 126.1, 125.9, 125.8, 123.2, 120.5, 119.4, 112.3, 71.3, 19.4 ppm, one resonance was not observed due to overlapping peaks; **IR** (thin film): 3054, 2988, 1621, 1448, 1319, 1199, 1110, 1005, 879, 781, 655 cm^{-1}; **HRMS** calc'd for $C_{27}H_{22}N^+$ 360.1747, found 360.1747 [M+H]$^+$; **HPLC analysis:** Daicel CHIRALPAK OD-H, *n*-hexane/*i*-PrOH = 95/5, flow rate = 0.8 mL/min, λ = 260 nm, retention time: t_{major} = 15.77 min, t_{minor} = 10.85 min; $[\alpha]_D^{20}$ = +11.25 (c 1.0, $CHCl_3$).

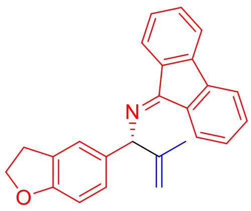

(*R*)-*N*-(1-(2,3-Dihydrobenzofuran-5-yl)-2-methylallyl)-9*H*-fluoren-9-imine (3la): The reaction was performed following the general procedure with (*E*)-1-(2,3-dihydrobenzofuran-5-yl)-*N*-(9*H*-fluoren-9-yl)methanimine **1l** (124.6 mg, 0.4 mmol) and 2-bromoprop-1-ene **2a** (145.2 mg, 1.2 mmol). The crude product was separated by flash chromatography on deactivated silica gel (hexanes to hexanes:ethyl acetate = 20:1). Further purification was performed on an Agilent HPLC 1260 system using acetonitrile:H_2O (90:10 vol./vol.) as mobile phase and flow rate of 4 mL/min with monitoring at 254 nm to give the product **3la** (129.3 mg, 92% yield, 95% *ee*) as a yellow oil. **R*f*** = 0.34 (hexanes:ethyl acetate = 10:1). **^{1}H NMR** (400 MHz, Chloroform-*d*) δ 7.86 (d, *J* = 7.2 Hz, 1H), 7.69 (d, *J* = 7.6 Hz, 1H), 7.57 (d, *J* = 7.6 Hz, 1H), 7.49 (d, *J* = 7.2 Hz, 1H), 7.33 (td, *J* = 4.4, 1.2 Hz, 1H), 7.30 (td, *J* = 4.4, 1.2 Hz, 1H), 7.26 (s, 1H), 7.23 (td, *J* = 7.6, 1.2 Hz, 1H), 7.17 – 7.12 (m, 2H), 6.66 (d, *J* = 8.0 Hz, 1H), 5.92 (s, 1H), 5.04 (s, 1H), 4.84 (s, 1H), 4.47 (t, *J* = 8.8 Hz, 2H), 3.11 (t, *J* = 8.4 Hz, 2H), 1.77 (s, 3H) ppm; **^{13}C{^{1}H} NMR** (100 MHz, Chloroform-*d*) δ 162.2, 159.3, 147.9, 144.1, 141.1, 139.1, 134.7, 131.8, 131.3, 131.0, 128.4, 128.0, 127.8, 127.31, 127.25, 124.0, 123.1, 120.4, 119.3, 111.6, 109.0, 71.4, 70.6, 30.0, 19.4 ppm; **IR** (thin film): 3056, 2988, 1610, 1543, 1449, 1275, 1100, 913, 764, 655 cm^{-1}; **HRMS** calc'd for $C_{25}H_{22}NO^+$ 352.1696, found 352.1694 [M+H]$^+$; **HPLC analysis:** Daicel CHIRALPAK OJ-H, *n*-hexane/*i*-PrOH = 85/15, flow rate = 0.5 mL/min, λ = 260 nm, retention time: t_{major} = 15.97 min, t_{minor} = 18.71 min; $[\alpha]_D^{20}$ = +31.71 (c 1.0, $CHCl_3$).

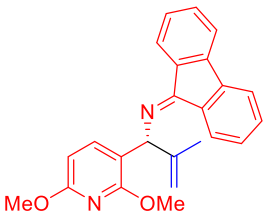

(*R*)-*N*-(1-(2,6-Dimethoxypyridin-3-yl)-2-methylallyl)-9*H*-fluoren-9-imine (3ma): The reaction was performed following the general procedure with (*E*)-1-(2,6-dimethoxypyridin-3-yl)-*N*-(9*H*-fluoren-9-yl)methanimine **1m** (132.2 mg, 0.4 mmol) and 2-bromoprop-1-ene **2a** (145.2 mg, 1.2 mmb The crude product was separated by flash chromatography on deactivated silica gel (hexanes to hexanes:ethyl acetate = 100:1). Further purification was performed on an Agilent HPLC 1260 system using acetonitrile:H_2O (90:10 vol./vol.) as mobile phase and flow rate of 4 mL/min with monitoring at 254 nm to give the product **3ma** (134.9 mg, 91% yield, 85% *ee*) as a yellow oil. **R*f*** = 0.41 (hexanes:ethyl acetate = 10:1). **^{1}H NMR** (400 MHz, Chloroform-*d*) δ 7.82 – 7.79 (m, 2H), 7.73 (d, *J* = 8.0 Hz, 1H), 7.49 (d, *J* = 7.2 Hz, 1H), 7.43 (d, *J* = 7.2 Hz, 1H), 7.26 (t, *J* = 7.6 Hz, 2H), 7.20 – 7.12 (m, 2H), 6.22 – 6.20 (m, 2H), 5.01 (s, 1H), 4.80 (s, 1H), 3.91 (s, 3H), 3.79 (s, 3H), 1.76 (s, 3H) ppm; **^{13}C{^{1}H} NMR** (100 MHz, Chloroform-*d*) δ 162.4, 161.9, 159.5, 146.8, 144.0, 141.1, 140.2, 139.1, 131.7, 131.2, 130.9, 128.3, 128.1, 127.6, 122.9, 120.3, 119.3, 116.6, 111.5, 101.2,

62.5, 53.6, 53.5, 19.8 ppm; **IR** (thin film): 3058, 2944, 1641, 1450, 1386, 1245, 1095, 954, 764, 653 cm^{-1}; **HRMS** calc'd for $C_{24}H_{23}N_2O_2^+$ 371.1754, found 371.1753 $[M+H]^+$; **HPLC analysis:** Daicel CHIRALPAK OD-H, *n*-hexane/*i*-PrOH = 98/2, flow rate = 0.8 mL/min, λ = 260 nm, retention time: t_{major} = 8.23 min, t_{minor} = 6.63 min; $[\alpha]_D^{20}$ = -46.97 (c 1.0, $CHCl_3$).

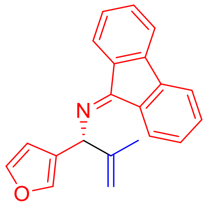

(*R*)-*N*-(1-(Furan-3-yl)-2-methylallyl)-9*H*-fluoren-9-imine (3na): The reaction was performed following the general procedure with (*E*)-*N*-(9*H*-fluoren-9-yl)-1-(furan-3-yl)methanimine **1n** (103.7 mg, 0.4 mmol) and 2-bromoprop-1-ene **2a** (145.2 mg, 1.2 mmol). The crude product was separated by flash chromatography on deactivated silica gel (hexanes to hexanes:ethyl acetate = 100:1). Further purification was performed on an Agilent HPLC 1260 system using acetonitrile:H_2O (90:10 vol./vol.) as mobile phase and flow rate of 5 mL/min with monitoring at 254 nm to give the product **3na** (112.6 mg, 94% yield, 51% *ee*) as a yellow oil. $\mathbf{R}_f$ = 0.46 (hexanes:ethyl acetate = 10:1). **^{1}H NMR** (400 MHz, Chloroform-*d*) δ 7.83 (d, *J* = 7.2 Hz, 1H), 7.67 (d, *J* = 7.6 Hz, 1H), 7.56 (d, *J* = 7.6 Hz, 1H), 7.49 (d, *J* = 7.6 Hz, 1H), 7.34 – 7.30 (m, 3H), 7.26 – 7.20 (m, 2H), 7.14 (td, *J* = 7.6, 0.8 Hz, 1H), 6.37 (s, 1H), 5.94 (s, 1H), 5.06 (s, 1H), 4.88 (s, 1H), 1.83 (s, 3H) ppm; **^{13}C{^{1}H} NMR** (100 MHz, Chloroform-*d*) δ 162.7, 146.9, 144.1, 143.2, 141.1, 140.0, 138.9, 131.6, 131.4, 131.1, 128.4, 128.1, 127.9, 126.5, 123.1, 120.5, 119.4, 112.2, 110.1, 63.9, 18.9 ppm; **IR** (thin film): 3052, 2920, 1644, 1449, 1324, 1158, 1019, 913, 874, 730, 654 cm^{-1}; **HRMS** calc'd for $C_{21}H_{18}NO^+$ 300.1383, found 300.1383 $[M+H]^+$; **HPLC analysis:** Daicel CHIRALPAK OD-H, *n*-hexane/*i*-PrOH = 98/2, flow rate = 1.0 mL/min, λ = 250 nm, retention time: t_{major} = 8.03 min, t_{minor} = 9.33 min; $[\alpha]_D^{20}$ = -7.37 (c 1.0, $CHCl_3$).

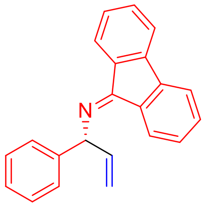

(*R*)-*N*-(1-Phenylallyl)-9*H*-fluoren-9-imin*e* (3ab): The reaction was performed following the general procedure with (*E*)-*N*-(9*H*-fluoren-9-yl)-1-phenylmethanimine **1a** (107.7 mg, 0.4 mmol) and bromoethene **2b** (1.2 ml, 1.0 M in THF, 1.2 mmol). The crude product was separated by flash chromatography on deactivated silica gel (hexanes to hexanes:ethyl acetate = 100:1). Further purification was performed on an Agilent HPLC 1260 system using acetonitrile:H_2O (80:20 vol./vol.) as mobile phase and flow rate of 4 mL/min with monitoring at 254 nm to give the product **3ab** (70.9 mg, 60% yield, 87% *ee*) as a yellow oil. $\mathbf{R}_f$ = 0.34 (hexanes:ethyl acetate = 10:1). **^{1}H NMR** (400 MHz, Chloroform-*d*) δ 7.85 (d, *J* = 7.2 Hz, 1H), 7.75 (d, *J* = 7.6 Hz, 1H), 7.52 (d, *J* = 7.2 Hz, 1H), 7.45 (t, *J* = 7.6 Hz, 3H), 7.30 – 7.25 (m, 4H), 7.22 – 7.10 (m, 3H), 6.24 – 6.16 (m, 1H), 6.07 (d, *J* = 7.6 Hz, 1H), 5.19 (d, *J* = 17.2 Hz, 1H), 5.10 (d, *J* = 10.4 Hz, 1H) ppm; **^{13}C{^{1}H} NMR** (100 MHz, Chloroform-*d*) δ 162.7, 144.1, 143.0, 141.1, 140.1, 131.7, 131.4, 131.1, 128.7, 128.4, 128.1, 127.8, 127.4, 127.2, 123.1, 120.5, 119.4, 115.1, 67.4 ppm, one resonance was not observed due to overlapping peaks; **IR** (thin film): 3005, 2988, 1644, 1599, 1449, 1275, 1066, 913, 749, 700 cm^{-1}; **HRMS** calc'd for $C_{22}H_{18}N^+$ 296.1434, found 296.1432 $[M+H]^+$; **HPLC analysis:** Daicel CHIRALPAK OD-H, *n*-hexane/*i*-PrOH = 95/5, flow rate = 0.5 mL/min, λ = 260 nm, retention time: t_{major} = 10.57 min, t_{minor} = 12.20 min; $[\alpha]_D^{20}$ = +48.76 (c 1.0, $CHCl_3$).

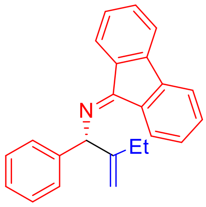

(R)-*N*-(2-Methylene-1-phenylbutyl)-9*H*-fluoren-9-imine (3ac): The reaction was performed following the general procedure with (*E*)-*N*-(9*H*-fluoren-9-yl)-1-phenylmethanimine **1a** (107.7 mg, 0.4 mmol) and 2-bromobut-1-ene **2c** (162.0 mg, 1.2 mmol). The crude product was separated by flash chromatography on deactivated silica gel (hexanes to hexanes:ethyl acetate = 100:1). Further purification was performed on an Agilent HPLC 1260 system using acetonitrile:H_2O (90:10 vol./vol.) as mobile phase and flow rate of 4 mL/min with monitoring at 254 nm to give the product **3ac** (116.4 mg, 90% yield, 93% *ee*) as a yellow oil. $\mathbf{R_f}$ = 0.32 (hexanes:ethyl acetate = 10:1). **^{1}H NMR** (400 MHz, Chloroform-*d*) δ 7.86 (d, *J* = 7.2 Hz, 1H), 7.69 (d, *J* = 8.0 Hz, 1H), 7.55 (d, *J* = 7.6 Hz, 1H), 7.48 (d, *J* = 7.6 Hz, 1H), 7.42 (d, *J* = 7.2 Hz, 2H), 7.33 – 7.22 (m, 5H), 7.17 – 7.11 (m, 2H), 6.05 (s, 1H), 5.12 (s, 1H), 4.88 (s, 1H), 2.27 – 2.17 (m, 1H), 2.06 – 1.96 (m, 1H), 0.98 (t, *J* = 7.2 Hz, 3H) ppm; **^{13}C{^{1}H} NMR** (100 MHz, Chloroform-*d*) δ 162.3, 153.1, 144.2, 142.9, 141.1, 139.1, 131.7, 131.3, 131.0, 128.5, 128.4, 128.2, 128.0, 127.7, 127.1, 123.1, 120.4, 119.3, 109.8, 70.8, 25.1, 12.3 ppm; **IR** (thin film): 3051, 2955, 1638, 1448, 1252, 1111, 999, 906, 731, 702 cm^{-1}; **HRMS** calc'd for $C_{24}H_{22}N^+$ 324.1747, found 324.1749 $[M+H]^+$; **HPLC analysis:** Daicel CHIRALPAK OD-H, *n*-hexane/*i*-PrOH = 90/10, flow rate = 0.5 mL/min, λ = 260 nm, retention time: t_{major} = 6.65 min, t_{minor} = 8.75 min; $[\alpha]_D^{20}$ = +12.53 (c 1.0, $CHCl_3$).

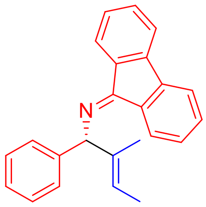

(*R*, *E*)-*N*-(2-Methyl-1-phenylbut-2-en-1-yl)-9*H*-fluoren-9-imine (3ad): The reaction was performed following the general procedure with (*E*)-*N*-(9*H*-fluoren-9-yl)-1-phenylmethanimine **1a** (107.7 mg, 0.4 mmol) and (*E*)-2-bromobut-2-ene **2d** (162.0 mg, 1.2 mmol). The crude product was separated by flash chromatography on deactivated silica gel (hexanes to hexanes:ethyl acetate = 100:1). Further purification was performed on an Agilent HPLC 1260 system using acetonitrile:H_2O (90:10 vol./vol.) as mobile phase and flow rate of 4 mL/min with monitoring at 254 nm to give the product **3ad** (80.2 mg, 62% yield, 70% *ee*) as a yellow oil. $\mathbf{R_f}$ = 0.57 (hexanes:ethyl acetate = 10:1). **^{1}H NMR** (400 MHz, Chloroform-*d*) δ 7.86 (d, *J* = 7.2 Hz, 1H), 7.63 (d, *J* = 7.6 Hz, 1H), 7.53 (d, *J* = 7.2 Hz, 1H), 7.47 (d, *J* = 7.6 Hz, 1H), 7.36 (d, *J* = 7.2 Hz, 2H), 7.32 – 7.20 (m, 5H), 7.15 – 7.07 (m, 2H), 5.96 (s, 1H), 5.60 (q, *J* = 6.4 Hz, 1H), 1.65 (s, 3H), 1.57 (d, *J* = 6.8 Hz, 3H) ppm; **^{13}C{^{1}H} NMR** (100 MHz, Chloroform-*d*) δ 162.2, 144.1, 142.9, 141.1, 139.1, 138.5, 131.8, 131.2, 130.9, 128.38, 128.36, 128.0, 127.8, 127.4, 126.9, 123.1, 121.0, 120.4, 119.3, 72.5, 13.5, 12.9 ppm; **IR** (thin film): 3051, 2967, 1652, 1444, 1334, 1153, 1032, 913, 748, 667 cm^{-1}; **HRMS** calc'd for $C_{24}H_{22}N^+$ 324.1747, found 324.1749 $[M+H]^+$; **HPLC analysis:** Daicel CHIRALPAK OJ-H, *n*-hexane/*i*-PrOH = 95/5, flow rate = 0.3 mL/min, λ = 260 nm, retention time: t_{major} = 21.74 min, t_{minor} = 16.67 min; $[\alpha]_D^{20}$ = +0.20 (c 1.0, $CHCl_3$).

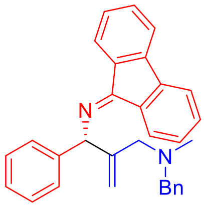

(*S*)-2-(((9*H*-Fluoren-9-ylidene)amino)(phenyl)methyl)-*N*-benzyl-*N*-methylprop-2-en-1-amine (3ae): The reaction was performed following the general procedure with (*E*)-*N*-(9*H*-fluoren-9-yl)-1-phenylmethanimine **1a** (107.7 mg, 0.4 mmol) and *N*-benzyl-2-bromo-*N*-methylprop-2-en-1-amine **2e** (288.2 mg, 1.2 mmol). The crude product was separated by flash chromatography on deactivated silica gel (hexanes to hexanes:ethyl acetate = 15:1). Further purification was performed on an Agilent HPLC 1260 system using acetonitrile:H_2O (95:5 vol./vol.) as mobile phase and flow rate of 4 mL/min with monitoring at 254 nm to give the product **3ae** (140.6 mg, 82% yield, 93% *ee*) as a yellow oil. $\mathbf{R_f}$ = 0.40 (hexanes:ethyl acetate = 10:1). **^{1}H NMR** (400 MHz, Chloroform-*d*) δ 8.07 (d, *J* = 7.6 Hz, 1H), 7.82 (d, *J* = 7.6 Hz, 1H), 7.53 (d, *J* = 7.6 Hz, 1H), 7.46 (d, *J* = 7.6 Hz, 1H), 7.38 – 7.35

(m, 2H), 7.31 – 7.25 (m, 4H), 7.23 – 7.16 (m, 6H), 7.15 – 7.10 (m, 1H), 7.02 (td, *J* = 7.6, 1.2 Hz, 1H), 6.29 (s, 1H), 5.26 (s, 1H), 5.08 (s, 1H), 3.50 (d, *J* = 13.2 Hz, 1H), 3.36 (d, *J* = 13.2 Hz, 1H), 3.02 (d, *J* = 13.2 Hz, 1H), 2.80 (d, *J* = 13.2 Hz, 1H), 2.06 (s, 3H) ppm; **^{13}C{^{1}H} NMR** (100 MHz, Chloroform-*d*) δ 161.9, 149.3, 144.0, 143.2, 141.1, 139.7, 139.1, 131.9, 131.2, 130.9, 129.1, 128.4, 128.3, 128.13, 128.07, 128.0, 127.1, 123.0, 120.3, 119.3, 113.8, 67.2, 62.6, 61.5, 42.4 ppm, two resonances were not observed due to overlapping peaks; **IR** (thin film): 3392, 2928, 1644, 1449, 1270, 1102, 1024, 913, 742, 698, 654 cm^{-1}; **HRMS** calc'd for $C_{31}H_{29}N_2^+$ 429.2325, found 429.2330 [M+H]$^+$; **HPLC analysis:** Daicel CHIRALPAK IA, *n*-hexane/*i*-PrOH = 98/2, flow rate = 0.8 mL/min, λ = 260 nm, retention time: t_{major} = 5.09 min, t_{minor} = 5.51 min; $[\alpha]_D^{20}$ = +151.54 (c 1.0, CHCl$_3$).

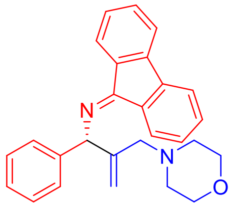

(*S*)-*N*-(2-(Morpholinomethyl)-1-phenylallyl)-9*H*-fluoren-9-imine (3af): The reaction was performed following the general procedure with (*E*)-*N*-(9*H*-fluoren-9-yl)-1-phenylmethanimine **1a** (107.7 mg, 0.4 mmol) and 4-(2-bromoallyl)morpholine **2f** (247.3 mg, 1.2 mmol). The crude product was separated by flash chromatography on deactivated silica gel (hexanes to hexanes:ethyl acetate = 15:1). Further purification was performed on an Agilent HPLC 1260 system using acetonitrile:H_2O (90:10 vol./vol.) as mobile phase and flow rate of 5 mL/min with monitoring at 254 nm to give the product **3af** (123.1 mg, 78% yield, 95% *ee*) as a yellow oil. **R*f*** = 0.38 (hexanes:ethyl acetate = 10:1). **^{1}H NMR** (400 MHz, Chloroform-*d*) δ 8.00 (d, *J* = 8.0 Hz, 1H), 7.82 (d, *J* = 7.2 Hz, 1H), 7.50 (d, *J* = 7.6 Hz, 1H), 7.46 (d, *J* = 6.8 Hz, 2H), 7.42 (d, *J* = 7.2 Hz, 1H), 7.28 – 7.21 (m, 4H), 7.17 – 7.09 (m, 3H), 6.30 (s, 1H), 5.22 (s, 1H), 5.02 (s, 1H), 3.64 – 3.55 (m, 4H), 2.88 (d, *J* = 13.2 Hz, 1H), 2.77 (d, *J* = 13.2 Hz, 1H), 2.37 – 2.32 (m, 2H), 2.27 – 2.21 (m, 2H) ppm; **^{13}C{^{1}H} NMR** (100 MHz, Chloroform-*d*) δ 161.7, 147.5, 144.1, 143.0, 141.0, 139.0, 131.9, 131.2, 130.9, 128.4, 128.3, 128.0, 127.9, 127.7, 127.2, 123.0, 120.4, 119.3, 114.2, 67.3, 62.3, 53.9 ppm, one resonance was not observed due to overlapping peaks; **IR** (thin film): 2960, 2853, 1643, 1450, 1347, 1261, 1116, 913, 793, 700, 653 cm^{-1}; **HRMS** calc'd for $C_{27}H_{27}N_2O^+$ 395.2118, found 395.2118 [M+H]$^+$; **HPLC analysis:** Daicel CHIRALPAK OD-H, *n*-hexane/*i*-PrOH = 98/2, flow rate = 0.8 mL/min, λ = 260 nm, retention time: t_{major} = 11.37 min, t_{minor} = 17.14 min; $[\alpha]_D^{20}$ = +95.34 (c 1.0, CHCl$_3$).

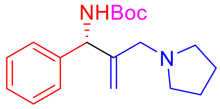

tert*-Butyl (*S*)-(1-Phenyl-2-(pyrrolidin-1-ylmethyl)allyl)carbamate (3ag):** The reaction was performed following the general procedure with (*E*)-*N*-(9*H*-fluoren-9-yl)-1-phenylmethanimine **1a** (107.7 mg, 0.4 mmol) and 1-(2-bromoallyl)pyrrolidine **2g** (228.1 mg, 1.2 mmol). The reaction was worked up following **General Workup Procedure**. The crude product was separated by flash chromatography on deactivated silica gel (hexanes to hexanes:ethyl acetate = 5:1) to give the product **3ag** (97.5 mg, 77% yield, 98% *ee*) as a colorless oil. **R*f = 0.39 (hexanes:ethyl acetate = 3:1). **^{1}H NMR** (400 MHz, Chloroform-*d*) δ 7.25 – 7.23 (m, 4H), 7.18 – 7.13 (m, 1H), 5.33 (d, *J* = 8.0 Hz, 1H), 5.11 (s, 1H), 4.96 (s, 1H), 3.08 (d, *J* = 12.8 Hz, 1H), 2.60 (d, *J* = 12.8 Hz, 1H), 2.43 – 2.38 (m, 2H), 2.36 – 2.31 (m, 2H), 1.73 – 1.66 (m, 4H), 1.36 (s, 9H) ppm; **^{13}C{^{1}H} NMR** (100 MHz, Chloroform-*d*) δ 155.7, 145.5, 141.5, 128.4, 127.0, 126.5, 115.7, 78.9, 60.2, 59.0, 53.9, 28.6, 23.6 ppm; **IR** (thin film): 2931, 1717, 1437, 1356, 1219, 1107, 977, 772 cm^{-1}; **HRMS** calc'd for $C_{19}H_{29}N_2O_2^+$ 317.2224, found 317.2224 [M+H]$^+$; **HPLC analysis** of the *N*-fluorenyl imine product: Daicel CHIRALPAK OJ-H, *n*-hexane/*i*-PrOH = 90/10, flow rate = 0.5 mL/min, λ = 260 nm, retention time: t_{major} = 8.33 min, t_{minor} = 11.79 min; $[\alpha]_D^{20}$ = -38.42 (c 1.0, CHCl$_3$).

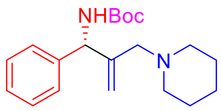

tert-Butyl (*S*)-(1-Phenyl-2-(piperidin-1-ylmethyl)allyl)carbamate (3ah): The reaction was performed following the general procedure with (*E*)-*N*-(9*H*-fluoren-9-yl)-1-phenylmethanimine **1a** (107.7 mg, 0.4 mmol) and 1-(2-bromoallyl)piperidine **2h** (244.9 mg, 1.2 mmol). The reaction was worked up following **General Workup Procedure**. The crude product was separated by flash chromatography on deactivated silica gel (hexanes to hexanes:ethyl acetate = 5:1) to give the product **3ah** (95.2 mg, 72% yield, 98% *ee*) as a colorless oil. $\mathbf{R}_f$ = 0.43 (hexanes:ethyl acetate = 3:1). **^{1}H NMR** (400 MHz, Chloroform-*d*) δ 7.70 (s, 1H), 7.33 – 7.28 (m, 4H), 7.25 – 7.19 (m, 1H), 5.40 (d, *J* = 7.6 Hz, 1H), 5.25 (s, 1H), 4.95 (s, 1H), 2.90 (d, *J* = 12.8 Hz, 1H), 2.55 (d, *J* = 12.8 Hz, 1H), 2.52 – 2.37 (m, 2H), 2.29 – 2.11 (m, 2H), 1.64 – 1.57 (m, 4H), 1.44 (s, 9H), 1.33 – 1.29 (m, 2H) ppm; **^{13}C{^{1}H} NMR** (100 MHz, Chloroform-*d*) δ 155.8, 144.3, 141.7, 128.4, 127.0, 126.4, 117.2, 78.8, 62.4, 60.6, 54.3, 28.6, 26.3, 24.5 ppm; **IR** (thin film): 2988, 1802, 1449, 1380, 1219, 1126, 898, 771 cm^{-1}; **HRMS** calc'd for $C_{20}H_{31}N_2O_2^+$ 331.2380, found 331.2376 $[M+H]^+$; **HPLC analysis** of the *N*-fluorenyl imine product: Daicel CHIRALPAK OJ-H, *n*-hexane/*i*-PrOH = 80/20, flow rate = 0.1 mL/min, λ = 260 nm, retention time: t_{major} = 37.91 min, t_{minor} = 41.37 min; $[\alpha]_D^{20}$ = -53.94 (c 1.0, $CHCl_3$).

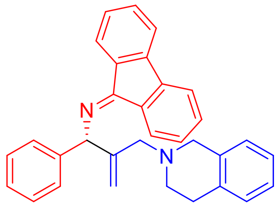

(*S*)-*N*-(2-((3,4-Dihydroisoquinolin-2(1*H*)-yl)methyl)-1-phenylallyl)-9*H*-fluoren-9-imine (3ai): The reaction was performed following the general procedure with (*E*)-*N*-(9*H*-fluoren-9-yl)-1-phenylmethanimine **1a** (107.7 mg, 0.4 mmol) and 2-(2-bromoallyl)-1,2,3,4-tetrahydroisoquinoline **2i** (302.6 mg, 1.2 mmol). The crude product was separated by flash chromatography on deactivated silica gel (hexanes to hexanes:ethyl acetate = 15:1). Further purification was performed on an Agilent HPLC 1260 system using acetonitrile:H_2O (95:5 vol./vol.) as mobile phase and flow rate of 5 mL/min with monitoring at 254 nm to give the product **3ai** (163.9 mg, 93% yield, 95% *ee*) as a yellow oil. $\mathbf{R}_f$ = 0.35 (hexanes:ethyl acetate = 10:1). **^{1}H NMR** (400 MHz, Chloroform-*d*) δ 7.98 (d, *J* = 7.6 Hz, 1H), 7.84 (d, *J* = 7.6 Hz, 1H), 7.52 (d, *J* = 6.8 Hz, 2H), 7.44 (d, *J* = 7.6 Hz, 1H), 7.40 (d, *J* = 7.6 Hz, 1H), 7.28 – 7.23 (m, 3H), 7.19 – 7.12 (m, 3H), 7.09 – 6.99 (m, 3H), 6.85 (d, *J* = 7.2 Hz, 1H), 6.66 (t, *J* = 7.2 Hz, 1H), 6.38 (s, 1H), 5.28 (s, 1H), 5.08 (s, 1H), 3.57 (d, *J* = 15.2 Hz, 1H), 3.42 (d, *J* = 14.8 Hz, 1H), 3.07 (d, *J* = 13.2 Hz, 1H), 2.94 – 2.86 (m, 2H), 2.83 – 2.76 (m, 1H), 2.71 – 2.65 (m, 1H), 2.60 – 2.54 (m, 1H) ppm; **^{13}C{^{1}H} NMR** (100 MHz, Chloroform-*d*) δ 161.8, 148.2, 143.8, 143.3, 141.1, 139.0, 135.3, 134.7, 131.8, 131.1, 130.9, 128.8, 128.4, 128.22, 128.16, 128.0, 127.2, 126.8, 126.2, 125.6, 123.0, 120.2, 119.2, 113.7, 67.3, 62.1, 56.3, 51.3, 29.6 ppm, one resonance was not observed due to overlapping peaks; **IR** (thin film): 2989, 1637, 1450, 1252, 1110, 934, 889, 703, 653 cm^{-1}; **HRMS** calc'd for $C_{32}H_{29}N_2^+$ 441.2325, found 441.2325 $[M+H]^+$; **HPLC analysis:** Daicel CHIRALPAK IA, *n*-hexane/*i*-PrOH = 98/2, flow rate = 0.8 mL/min, λ = 260 nm, retention time: t_{major} = 5.64 min, t_{minor} = 6.90 min; $[\alpha]_D^{20}$ = +66.02 (c 1.0, $CHCl_3$).

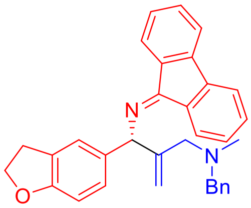

(*S*)-2-(((9*H*-Fluoren-9-ylidene)amino)(2,3-dihydrobenzofuran-5-yl)-methyl)-*N*-benzyl-*N*-methylprop-2-en-1-amine (3le): The reaction was performed following the general procedure with (*E*)-1-(2,3-dihydrobenzofuran-5-yl)-*N*-(9*H*-fluoren-9-yl)methanimine **1l** (124.6 mg, 0.4 mmol) and *N*-benzyl-2-bromo-*N*-methylprop-2-en-1-amine **2e** (288.2 mg, 1.2 mmol). The crude product was separated by flash chromatography on deactivated silica gel (hexanes to hexanes:ethyl acetate = 15:1). Further purification was performed on

an Agilent HPLC 1260 system using acetonitrile:H_2O (95:5 vol./vol.) as mobile phase and flow rate of 5 mL/min with monitoring at 254 nm to give the product **3la** (169.4 mg, 90% yield, 95% *ee*) as a yellow oil. **R*f*** = 0.31 (hexanes:ethyl acetate = 10:1). **^{1}H NMR** (400 MHz, Chloroform-*d*) δ 8.10 (d, *J* = 7.6 Hz, 1H), 7.79 (d, *J* = 7.2 Hz, 1H), 7.44 (d, *J* = 7.2 Hz, 1H), 7.37 (d, *J* = 7.2 Hz, 1H), 7.25 – 7.09 (m, 9H), 7.01 – 6.97 (m, 2H), 6.56 (d, *J* = 8.0 Hz, 1H), 6.19 (s, 1H), 5.23 (s, 1H), 4.99 (s, 1H), 4.34 (t, *J* = 8.4 Hz, 2H), 3.47 (d, *J* = 13.2 Hz, 1H), 3.29 (d, *J* = 13.2 Hz, 1H), 3.03 – 2.94 (m, 3H), 2.70 (d, *J* = 12.8 Hz, 1H), 2.03 (s, 3H) ppm; **^{13}C{^{1}H} NMR** (100 MHz, Chloroform-*d*) δ 161.4, 159.2, 149.6, 143.9, 141.0, 139.7, 139.0, 135.3, 131.8, 131.1, 130.8, 129.1, 128.3, 128.2, 128.1, 128.0, 127.8, 127.04, 126.96, 124.5, 122.9, 120.2, 119.2, 113.3, 108.8, 71.3, 66.7, 62.6, 61.4, 42.5, 29.9 ppm; **IR** (thin film): 3319, 2925, 2787, 1641, 1488, 1449, 1239, 1022, 942, 730, 698 cm^{-1}; **HRMS** calc'd for $C_{33}H_{31}N_2O^+$ 471.2431, found 471.2436 $[M+H]^+$; **HPLC analysis:** Daicel CHIRALPAK IE, *n*-hexane/*i*-PrOH = 85/15, flow rate = 0.3 mL/min, λ = 260 nm, retention time: t_{major} = 5.09 min, t_{minor} = 5.51 min; $[\alpha]_D^{20}$ = +202.91 (c 1.0, $CHCl_3$).

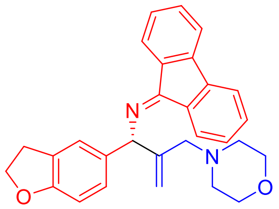

(*S*)-*N*-(1-(2,3-Dihydrobenzofuran-5-yl)-2-(morpholinomethyl)allyl)-9*H*-fluoren-9-imine (3lf): The reaction was performed following the general procedure with (*E*)-1-(2,3-dihydrobenzofuran-5-yl)-*N*-(9*H*-fluoren-9-yl)methanimine **1l** (124.6 mg, 0.4 mmol) and 4-(2-bromoallyl) morpholine **2f** (247.3 mg, 1.2 mmol). The crude product was separated by flash chromatography on deactivated silica gel (hexanes to hexanes:ethyl acetate = 15:1). Further purification was performed on an Agilent HPLC 1260 system using acetonitrile:H_2O (70:30 vol./vol.) as mobile phase and flow rate of 4 mL/min with monitoring at 254 nm to give the product **3lf** (153.7 mg, 88% yield, 86% *ee*) as a yellow oil. **R*f*** = 0.35 (hexanes:ethyl acetate = 10:1). **^{1}H NMR** (400 MHz, Chloroform-*d*) δ 8.03 (d, *J* = 7.6 Hz, 1H), 7.82 (d, *J* = 7.6 Hz, 1H), 7.51 (d, *J* = 7.2 Hz, 1H), 7.43 (d, *J* = 7.2 Hz, 1H), 7.32 – 7.24 (m, 3H), 7.19 – 7.11 (m, 3H), 6.64 (d, *J* = 8.4 Hz, 1H), 6.25 (s, 1H), 5.22 (s, 1H), 4.99 (s, 1H), 4.42 (t, *J* = 8.8 Hz, 2H), 3.66 – 3.57 (m, 4H), 3.06 (t, *J* = 8.8 Hz, 2H), 2.91 (d, *J* = 13.2 Hz, 1H), 2.76 (d, *J* = 13.2 Hz, 1H), 2.40 – 2.34 (m, 2H), 2.27 – 2.23 (m, 2H) ppm; **^{13}C{^{1}H} NMR** (100 MHz, Chloroform-*d*) δ 161.3, 159.3, 147.9, 144.0, 140.9, 139.0, 135.0, 131.9, 131.2, 130.8, 128.2, 127.9, 127.8, 127.7, 127.2, 124.4, 122.9, 120.3, 119.2, 113.7, 108.8, 71.3, 67.3, 66.8, 62.4, 53.9, 29.9 ppm; **IR** (thin film): 2961, 1642, 1488, 1260, 1116, 913, 867, 764, 654 cm^{-1}; **HRMS** calc'd for $C_{29}H_{29}N_2O_2^+$ 437.2224, found 437.2226 $[M+H]^+$; **HPLC analysis:** Daicel CHIRALPAK IE, *n*-hexane/*i*-PrOH = 99/1, flow rate = 1.2 mL/min, λ = 260 nm, retention time: t_{major} = 22.99 min, t_{minor} = 25.19 min; $[\alpha]_D^{20}$ = +122.52 (c 1.0, $CHCl_3$).

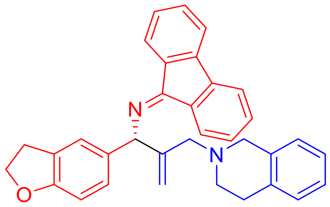

(*S*)-*N*-(1-(2,3-Dihydrobenzofuran-5-yl)-2-((3,4-dihydroisoquinolin-2(1*H*)-yl)methyl)allyl)-9*H*-fluoren-9-imine (3li): The reaction was performed following the general procedure with (*E*)-1-(2,3-dihydrobenzofuran-5-yl)-*N*-(9*H*-fluoren-9-yl)methanimine **1l** (124.6 mg, 0.4 mmol) and 2-(2-bromoallyl)-1,2,3,4-tetrahydroisoquinoline **2i** (302.6 mg, 1.2 mmol). The crude product was separated by flash chromatography on deactivated silica gel (hexanes to hexanes:ethyl acetate = 15:1). Further purification was performed on an Agilent HPLC 1260 system using acetonitrile:H_2O (95:5 vol./vol.) as mobile phase and flow rate of 4 mL/min with monitoring at 254 nm to give the product **3li** (175.7 mg, 91% yield, 95% *ee*) as a yellow oil. **R*f*** = 0.44 (hexanes:ethyl acetate = 10:1). **^{1}H NMR** (400 MHz, Chloroform-*d*) δ 7.99 (d, *J* = 8.0 Hz, 1H), 7.84 (d, *J* = 7.2 Hz, 1H), 7.45 (d, *J* = 7.2 Hz, 1H), 7.41 (d, *J* = 7.2 Hz, 1H), 7.37 (s,

1H), 7.26 – 7.13 (m, 4H), 7.09 – 6.99 (m, 3H), 6.85 (d, J = 7.2 Hz, 1H), 6.68 – 6.64 (m, 2H), 6.32 (s, 1H), 5.27 (s, 1H), 5.06 (s, 1H), 4.41 (t, J = 8.8 Hz, 2H), 3.58 (d, J = 14.8 Hz, 1H), 3.42 (d, J = 15.2 Hz, 1H), 3.13 – 3.03 (m, 3H), 2.93 – 2.86 (m, 2H), 2.84 – 2.77 (m, 1H), 2.73 – 2.67 (m, 1H), 2.61 – 2.55 (m, 1H) ppm; **$^{13}C\{^1H\}$ NMR** (100 MHz, Chloroform-*d*) δ 161.4, 159.3, 148.6, 143.8, 141.0, 139.0, 135.3, 134.6, 131.8, 131.0, 130.8, 128.7, 128.1, 128.01, 127.98, 127.9, 127.2, 126.8, 126.1, 125.6, 124.6, 122.9, 120.1, 119.2, 113.3, 108.9, 71.3, 66.9, 62.1, 56.3, 51.3, 29.9, 29.6 ppm, one resonance was not observed due to overlapping peaks; **IR** (thin film): 2974, 1639, 1487, 1382, 1269, 1129, 1090, 879, 732, 654 cm^{-1}; **HRMS** calc'd for $C_{34}H_{31}N_2O^+$ 483.2431, found 483.2432 $[M+H]^+$; **HPLC analysis:** Daicel CHIRALPAK IA, *n*-hexane/*i*-PrOH = 98/2, flow rate = 0.5 mL/min, λ = 260 nm, retention time: t_{major} = 5.64 min, t_{minor} = 6.90 min; $[\alpha]_D^{20}$ = +36.34 (c 1.0, $CHCl_3$).

6. Gram-scale sequential one-pot asymmetric imine synthesis/vinylation procedure

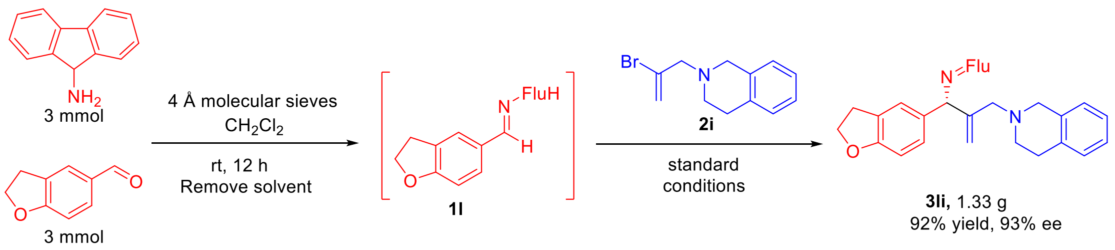

Into an oven-dried reaction Schlenk tube equipped with a magnetic stirring bar was added 9*H*-fluoren-9-amine (543.7 mg, 3 mmol, 1 equiv.) and 4 Å molecular sieves (1 g, powder, <50 µM). The flask was sealed with a rubber stopper and connected to a Schlenk line through a needle. The flask was evacuated, and then refilled with nitrogen. This process was repeated twice, and the reaction flask was kept under a nitrogen atmosphere during the course of the reaction. CH_2Cl_2 (15 mL) was added under nitrogen via syringe through the rubber septum. The resulting mixture was stirred at room temperature for 10 min before the 2,3-dihydrobenzofuran-5-carbaldehyde (444.5 mg, 3 mmol, 1 equiv.) was added under nitrogen via syringe through the rubber septum. The reaction was stirred at room temperature for 12 h, the solvent was completely removed in *vacuo* and the Schlenk tube was filled with nitrogen. A solution (prepared in the glove box) of $Ni(COD)_2$ (41.3 mg, 5 mol%) and **L1** (127.9 mg, 10 mol%) in 10 mL anhydrous THF was added to the Schlenk tube via syringe through the rubber septum. Then, a solution of vinyl bromide **2i** (2.27 g, 9 mmol, 3 equiv.) in 10 mL anhydrous THF was added by syringe through the rubber septum. Next, a solution of $NaN(SiMe_3)_2$ (825.2 mg, 4.5 mmol, 1.5 equiv.) in 10 mL anhydrous THF was added by syringe through the rubber septum. The reaction mixture was stirred for 12 h in total at 25 °C, opened to air, and quenched with 5 mL of H_2O. The layers were separated and the aqueous layer was extracted with CH_2Cl_2 (3X5 mL). The combined organic solution was washed by brine and dried over Na_2SO_4. The combined organic layers were concentrated in *vacuo*. The crude material was loaded onto a deactivated silica gel column via pipette and purified by flash chromatography on deactivated silica gel (eluted with hexanes to hexanes:ethyl acetate = 20:1) to give the product (1.33 g, 92% yield, 93% *ee*) as a yellow oil.

7. Transformation of the products

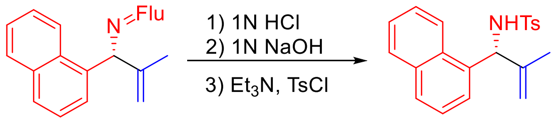

3ka **4ka**, 76%

(*R*)-4-Methyl-*N*-(2-methyl-1-(naphthalen-1-yl)allyl)benzenesulfonamide (4ka): Compound **3ka** (143.7 mg, 0.4 mmol) was worked up following **General workup procedure for the Ni-catalyzed asymmetric alkenylation of imines with alkenyl bromides** to yield the corresponding product **4ka** (106.8 mg, 76%), which was recrystallized in EtOAc/hexanes to give crystals that were suitable for X-ray analysis.

Mp: 137 – 139 °C. **R*f*** = 0.40 (hexanes:ethyl acetate = 4:1). **^{1}H NMR** (400 MHz, Chloroform-*d*) δ 7.81 (d, *J* = 8.0 Hz, 1H), 7.73 – 7.69 (m, 1H), 7.63 – 7.59 (m, 1H), 7.45 (dd, *J* = 6.8, 2.0 Hz, 2H), 7.39 – 7.32 (m, 2H), 7.21 – 7.16 (m, 2H), 6.95 (d, *J* = 8.0 Hz, 2H), 5.49 (d, *J* = 7.6 Hz, 1H), 5.15 (d, *J* = 8.0 Hz, 1H), 4.92 – 4.91 (m, 2H), 2.24 (s, 3H), 1.57 (s, 3H) ppm; **^{13}C{^{1}H} NMR** (100 MHz, Chloroform-*d*) δ 142.5, 142.0, 136.3, 133.5, 132.8, 129.8, 128.1, 127.7, 127.4, 126.0, 125.3, 124.7, 124.2, 124.1, 122.0, 112.8, 58.3, 20.4, 19.6 ppm; **IR** (thin film): 2963, 1649, 1511, 1446, 1261, 1159, 1019, 907, 798, 670 cm^{-1}; **HRMS** calc'd for $C_{21}H_{21}NNaO_2S^+$ 374.1185, found 374.1188 [M+Na]$^+$; **$[\alpha]_D^{20}$** = +8.25 (c 1.0, $CHCl_3$).

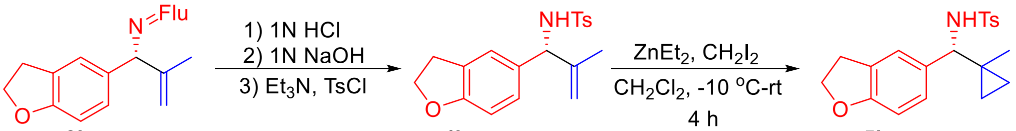

3la **4la**, 81% **5la**, 95%, 95% ee

(*R*)-*N*-(1-(2,3-Dihydrobenzofuran-5-yl)-2-methylallyl)-4-methylbenzenesulfonamide (4la): Compound **3la** (140.5 mg, 0.4 mmol) was worked up following **General workup procedure for the Ni-catalyzed asymmetric alkenylation of imines with alkenyl bromides** to yield the corresponding product **4la** (111.3 mg, 81%) as a colorless oil. **R*f*** = 0.33 (hexanes:ethyl acetate = 4:1). **^{1}H NMR** (400 MHz, Chloroform-*d*) δ 7.54 (d, *J* = 8.4 Hz, 2H), 7.11 (d, *J* = 8.4 Hz, 2H), 6.78 (s, 1H), 6.71 (dd, *J* = 8.4, 2.0 Hz, 1H), 6.50 (d, *J* = 8.0 Hz, 1H), 5.12 (d, *J* = 7.2 Hz, 1H), 4.91 (d, *J* = 1.2 Hz, 1H), 4.80 (dd, *J* = 3.2, 1.2 Hz, 1H), 4.66 (d, *J* = 7.2 Hz, 1H), 4.42 (td, *J* = 8.8, 1.2 Hz, 2H), 3.03 – 2.88 (m, 2H), 2.32 (s, 3H), 1.47 (s, 3H) ppm; **^{13}C{^{1}H} NMR** (100 MHz, Chloroform-*d*) δ 159.6, 143.8, 143.0, 137.7, 131.1, 129.3, 127.3, 127.3, 127.1, 123.7, 112.8, 109.0, 71.4, 62.5, 29.5, 21.5, 19.7 ppm; **IR** (thin film): 2921, 1598, 1491, 1326, 1160, 1094, 982, 770, 670 cm^{-1}; **HRMS** calc'd for $C_{19}H_{21}NNaO_3S^+$ 366.1134, found 366.1131 [M+Na]$^+$; **$[\alpha]_D^{20}$** = +85.39 (c 1.0, $CHCl_3$).

(*R*)-*N*-((2,3-Dihydrobenzofuran-5-yl)(1-methylcyclopropyl)methyl)-4-methylbenzenesulfon-amide (5la): The procedure reported by Charette was followed.[4] To a solution of compound **4la** (68.7 mg, 0.2 mmol) under a nitrogen atmosphere in CH_2Cl_2 (3.0 mL) at -10 °C, Et_2Zn (1 mL, 1.0 M in hexane, 1 mmol, 5.0 equiv.) and CH_2I_2 (267.6 mg, 1 mmol, 5.0 equiv.) were added sequentially. The resulting mixture was stirred for 3 h then the temperature was allowed to rise to room temperature. The mixture was stirred for 1 h at room temperature. The mixture was then diluted with Et_2O (50 mL) and HCl (10% aq., 5 mL) was added. The layers were separated and the organic layer was washed with saturated $NaHCO_3$ (10 mL), brine (10 mL), dried over Na_2SO_4 and filtered. The volatile materials were removed under vacuum and the resulting residue was purified by flash column chromatography (hexanes:EtOAc

= 6:1) to afford **5la** (67.9 mg, 95% yield, 95% *ee*) as a colorless oil.

R*f* = 0.33 (hexanes:ethyl acetate = 4:1). **^{1}H NMR** (400 MHz, Chloroform-*d*) δ 7.51 (d, *J* = 8.0 Hz, 2H), 7.07 (d, *J* = 8.4 Hz, 2H), 6.76 (s, 1H), 6.73 (dd, *J* = 8.4, 2.0 Hz, 1H), 6.48 (d, *J* = 8.0 Hz, 1H), 5.37 (d, *J* = 6.4 Hz, 1H), 4.46 – 4.37 (m, 2H), 3.75 (d, *J* = 6.8 Hz, 1H), 3.03 – 2.92 (m, 2H), 2.30 (s, 3H), 0.84 (s, 3H), 0.53 – 0.48 (m, 1H), 0.27 – 0.15 (m, 3H) ppm; **^{13}C{^{1}H} NMR** (100 MHz, Chloroform-*d*) δ 158.1, 141.8, 136.6, 130.3, 128.1, 126.2, 126.1, 125.6, 122.7, 107.5, 70.2, 63.1, 28.6, 20.4, 19.7, 18.8, 11.0, 10.5 ppm; **IR** (thin film): 2963, 1599, 1493, 1323, 1244, 1159, 1094, 983, 813, 757, 665 cm^{-1}; **HRMS** calc'd for $C_{20}H_{23}NNaO_3S^+$ 380.1291, found 380.1287 [M+Na]$^+$; **HPLC analysis:** Daicel CHIRALPAK AD-H, *n*-hexane/*i*-PrOH = 95/5, flow rate = 1 mL/min, λ = 254 nm, retention time: t_{major} = 34.87 min, t_{minor} = 39.37 min; **$[\alpha]_D^{20}$** = +54.58 (c 1.0, CHCl$_3$).

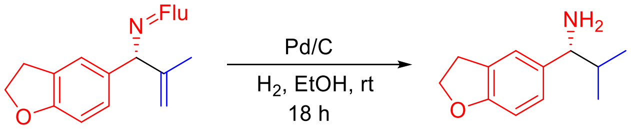

3la **6la,** 70%, 94% ee

(*R*)-1-(2,3-Dihydrobenzofuran-5-yl)-2-methylpropan-1-amine (6la): Following a procedure reported by Charette,[5] a 25 mL round-bottom flask was charged with **3la** (70.4 mg, 0.20 mmol, 1.0 equiv.) and EtOH (3.5 mL), followed by 10% Pd/C (0.020 mmol, 10 mol%). After purging the flask with hydrogen, the solution was stirred for 18 hours at ambient temperature. After consumption of starting material was confirmed by TLC analysis, the reaction mixture was filtered. Next, the combined organic layers were concentrated in *vacuo* and purified by flash column chromatography (EtOAc:Et_3N = 100:1) to afford **6la** (26.8 mg, 70% yield, 94% *ee*) as a colorless oil.

R*f* = 0.38 (EtOAc:MeOH = 10:1). **^{1}H NMR** (400 MHz, Chloroform-*d*) δ 7.13 (s, 1H), 6.98 (d, *J* = 8.0 Hz, 1H), 6.70 (d, *J* = 8.0 Hz, 1H), 4.53 (t, *J* = 8.4 Hz, 2H), 3.51 (d, *J* = 7.6 Hz, 1H), 3.17 (t, *J* = 8.4 Hz, 2H), 1.83 – 1.74 (m, 1H), 1.56 (s, 2H), 0.96 (d, *J* = 6.4 Hz, 3H), 074 (d, *J* = 6.8 Hz, 3H) ppm; **^{13}C{^{1}H} NMR** (100 MHz, Chloroform-*d*) δ 159.0, 137.6, 126.8, 126.8, 123.4, 108.6, 71.2, 62.2, 35.6, 29.8, 19.9, 19.2 ppm; **IR** (thin film): 2957, 1612, 1491, 1363, 1237, 1097, 984, 812 cm^{-1}; **HRMS** calc'd for $C_{12}H_{18}NO^+$ 192.1383, found 192.1384 [M+H]$^+$; **HPLC analysis:** Daicel CHIRALPAK IE, *n*-hexane/*i*-PrOH = 75/25, flow rate = 0.5 mL/min, λ = 260 nm, retention time: t_{major} = 36.23 min, t_{minor} = 33.59 min; **$[\alpha]_D^{20}$** = +12.00 (c 1.0, CHCl$_3$).

8. Reaction time course study of coupling between 1n and 2a (Table S6)

Experiments were set up inside a glovebox under a nitrogen atmosphere. Imine (**1n** 0.1 mmol/reaction) and vinyl bromide (**2a**, 0.3 mmol/reaction) were dosed together into 2 mL crimp top glass vials. A stock solution containing Ni(COD)$_2$ (0.005 mmol/reaction) and **L1** (0.01 mmol/reaction) in 0.5 mL of dry THF was taken up by syringe and added to the reaction vial under nitrogen. Then, NaN(SiMe$_3$)$_2$ (0.15 mmol/reaction) in 0.5 mL of dry THF was added to the reaction mixture. Total volume of the reactions is 1 mL, 0.1 M. The vials were sealed with crimp caps, removed from the glovebox and stirred at 25 ºC. Vials were sequentially quenched with 1 drop of water via syringe through the rubber septum at every 3 h until 12 h. The crude product was separated by flash chromatography on deactivated silica gel (hexanes to hexanes:ethyl acetate = 100:1). Further purification was performed on an Agilent HPLC 1260 system using acetonitrile:H_2O (90:10 vol./vol.) as mobile phase and flow rate of 5 mL/min with monitoring at 254 nm to give the yield of product **3na**. The ee of product **3na** was obtained by chiral phase HPLC.

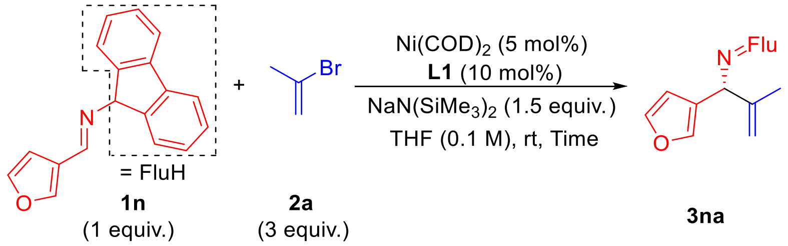

entry	Time	yield of **3na** (%)	ee of **3na** (%)
1	3 h	57	51
2	6 h	74	51
3	9 h	87	51
4	12 h	94	51

9. X-ray crystal structure of compound 4ka

Crystal data for **4ka**: $C_{21}H_{21}NO_2S$, *M* = 351.45, *a* = 6.5212(3) Å, *b* = 19.5200(8) Å, *c* = 27.5641(12) Å, *α* = 90°, *β* = 90°, *γ* = 90°, *V* = 3508.7(3) Å^3, *T* = 100.(2) K, space group *P*212121, *Z* = 8, *μ*(Cu Kα) = 1.745 mm^{-1}, 35118 reflections measured, 6901 independent reflections (R_{int} = 0.1480). The final R_1 values were 0.0497 ($I > 2\sigma(I)$). The final $wR(F^2)$ values were 0.1227 ($I > 2\sigma(I)$). The final R_1 values were 0.0713 (all data). The final $wR(F^2)$ values were 0.1379 (all data). The goodness of fit on F^2 was 1.066. Flack parameter = 0.067(12).

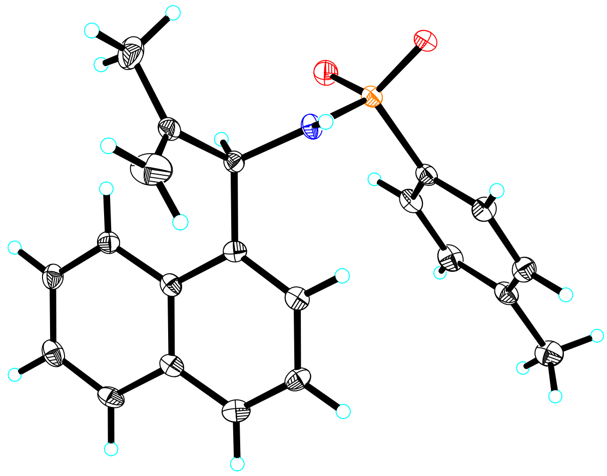

Figure S1. Crystal structure of **4ka** (**CCDC** 2058299)

Crystal data and structure refinement for 4ka.

Identification code	**4ka**	
Empirical formula	$C_{21}H_{21}NO_2S$	
Formula weight	351.45	
Temperature	100(2) K	
Wavelength	1.54178 Å	
Crystal system	Orthorhombic	
Space group	$P2_12_12_1$	
Unit cell dimensions	a = 6.5212(3) Å	α= 90°.
	b = 19.5200(8) Å	β= 90°.
	c = 27.5641(12) Å	γ = 90°.
Volume	3508.7(3) Å^3	
Z	8	
Density (calculated)	1.331 Mg/m^3	
Absorption coefficient	1.745 mm^{-1}	
F(000)	1488	
Crystal size	0.290 x 0.070 x 0.060 mm^3	
Theta range for data collection	2.77 to 72.55°.	
Index ranges	-8<=h<=8, -24<=k<=24, -34<=l<=24	
Reflections collected	35118	
Independent reflections	6901 [R(int) = 0.1480]	

Completeness to theta = 72.55 °	99.5 %
Absorption correction	Semi-empirical from equivalents
Max. and min. transmission	0.90 and 0.45
Refinement method	Full-matrix least-squares on F^2
Data / restraints / parameters	6901 / 0 / 455
Goodness-of-fit on F^2	1.066
Final R indices [I>2sigma(I)]	R1 = 0.0497, wR2 = 0.1227
R indices (all data)	R1 = 0.0713, wR2 = 0.1379
Absolute structure parameter	0.067(12)
Largest diff. peak and hole	0.328 and -0.693 e.$Å^{-3}$

10. Supplementary references

[1] Y. Zhu, S. L. Buchwald, *J. Am. Chem. Soc.* **2014**, 136, 4500−4503.

[2] J. Liu, C. G. Cao, H. B. Sun, X. Zhang, D. Niu, *J. Am. Chem. Soc.* **2016**, 138, 13103−13106.

[3] J. J. Molloy, C. P. Seath, M. J. West, C. Mclaughlin, N. J. Fazakerley, A. R. Kennedy, D. J. Nelson, A. J. B. Watson, *J. Am. Chem. Soc.* **2018**, 140, 126−130.

[4] Sergio Mata, Luis A. López, Rubén Vicente, *Angew. Chem. Int. Ed.* **2018**, 57, 11422–11426.

[5] Y. Matsumoto, J. Sawamura, Y. Murata, T. Nishikata, R. Yazaki, *J. Am. Chem. Soc.* **2020**, 142, 8498−8505.

11. NMR Spectra and HPLC chromatography of the products

Figure S1. ^{1}H NMR spectra (400 MHz, Chloroform-*d*) of (*E*)-*N*-(9*H*-Fluoren-9-yl)-1-(4-fluorophenyl)methanimine (1c).

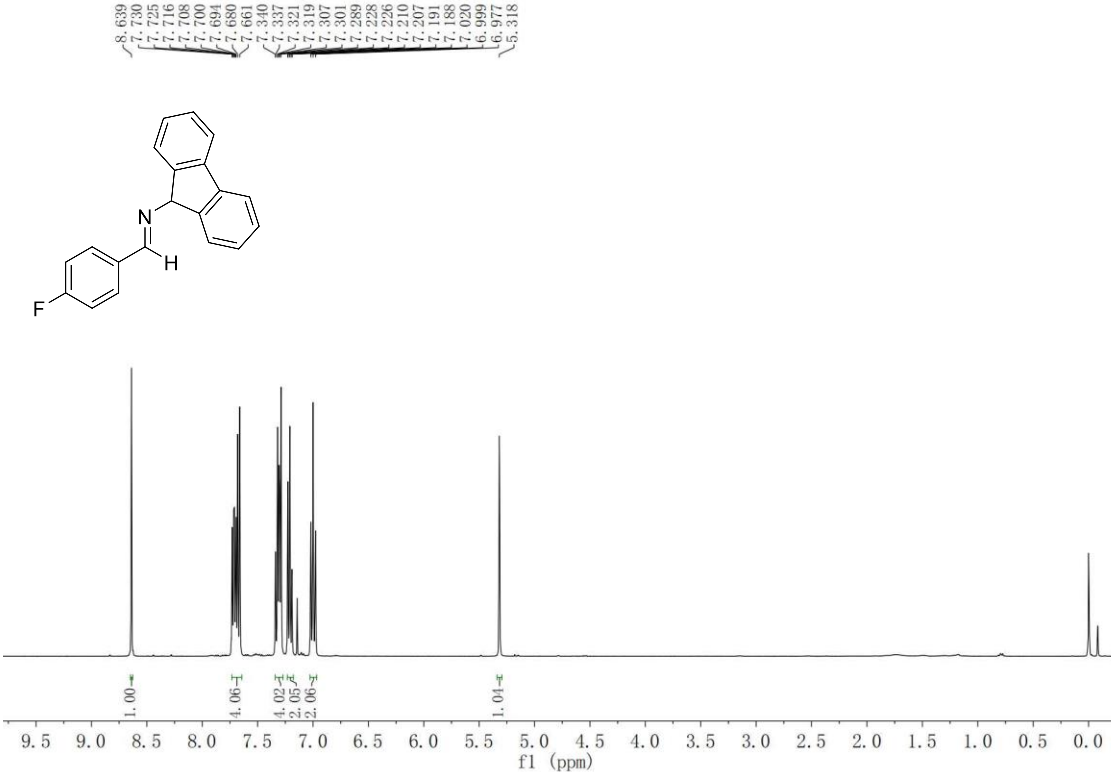

Figure S2. $^{13}C\{^1H\}$ NMR spectra (100 MHz, Chloroform-*d*) of (*E*)-*N*-(9*H*-Fluoren-9-yl)-1-(4-fluorophenyl)methanimine (1c).

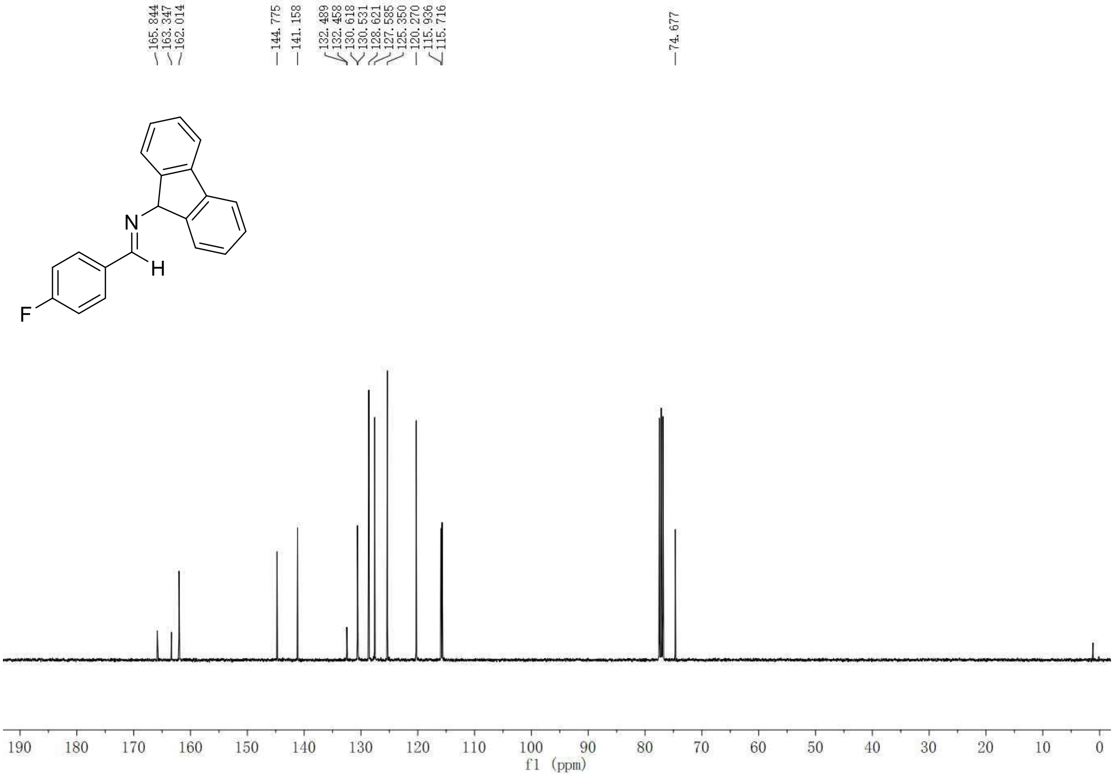

Figure S3. **^{19}F NMR spectra (376 MHz, Chloroform-*d*) of (*E*)-*N*-(9*H*-Fluoren-9-yl)-1-(4-fluorophenyl)methanimine (1c).**

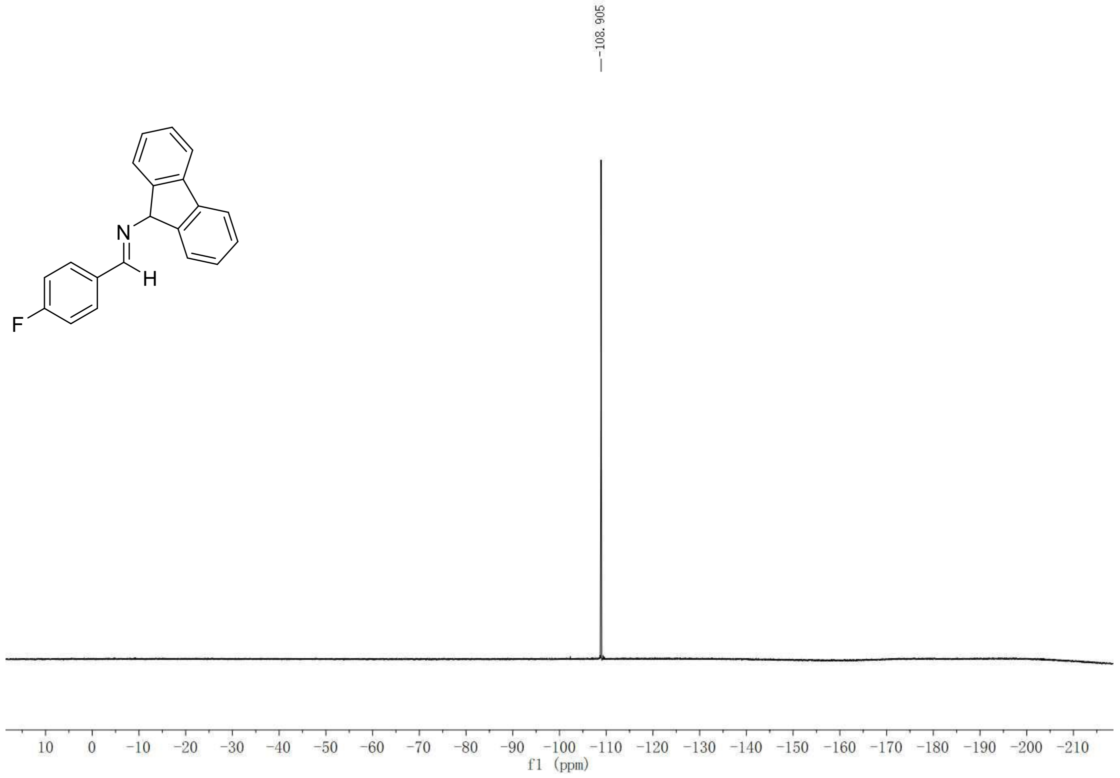

Figure S4. ^{1}**H NMR spectra (400 MHz, Chloroform-*d*) of (*E*)-1-(4-(*tert*-Butyl)phenyl)-*N*-(9*H*-fluoren-9-yl)methanimine (1f).**

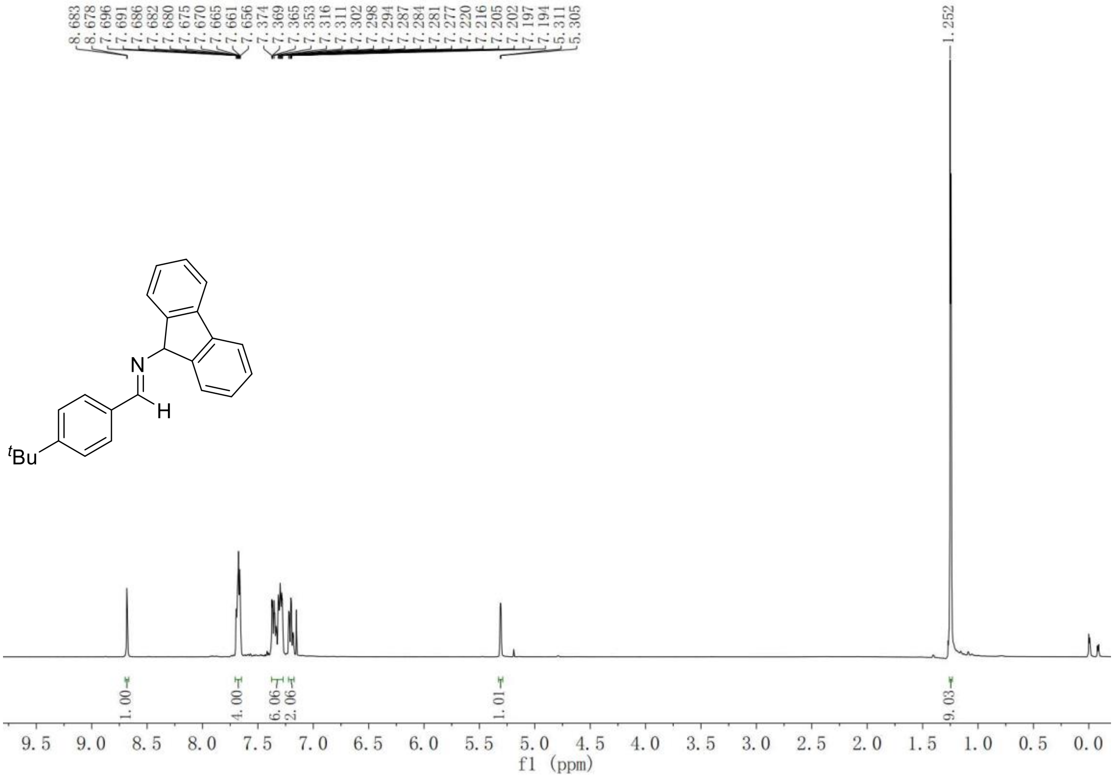

Figure S5. ^{13}**C{**^{1}**H} NMR spectra (100 MHz, Chloroform-*d*) of (*E*)-1-(4-(*tert*-Butyl)phenyl)-*N*-(9*H*-fluoren-9-yl)methanimine (1f).**

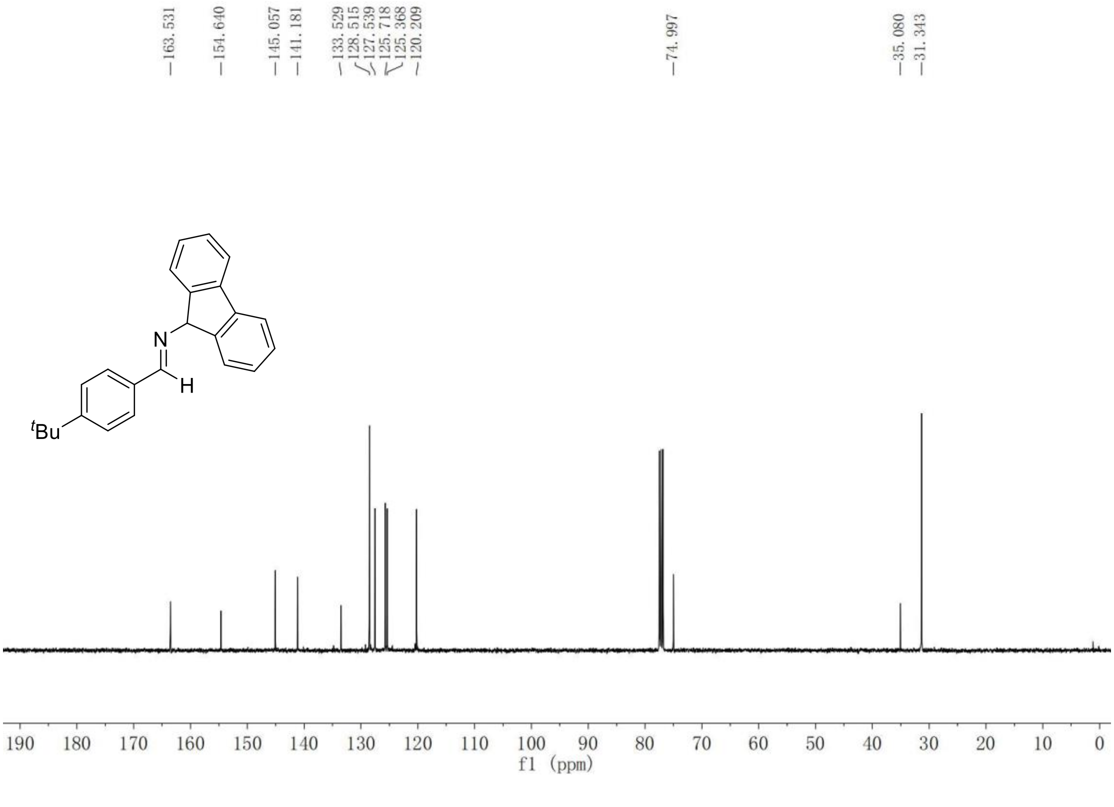

Figure S6. ¹H NMR spectra (400 MHz, Chloroform-*d*) of (*E*)-1-([1,1'-Biphenyl]-4-yl)-*N*-(9*H*-fluoren-9-yl)methanimine (1g).

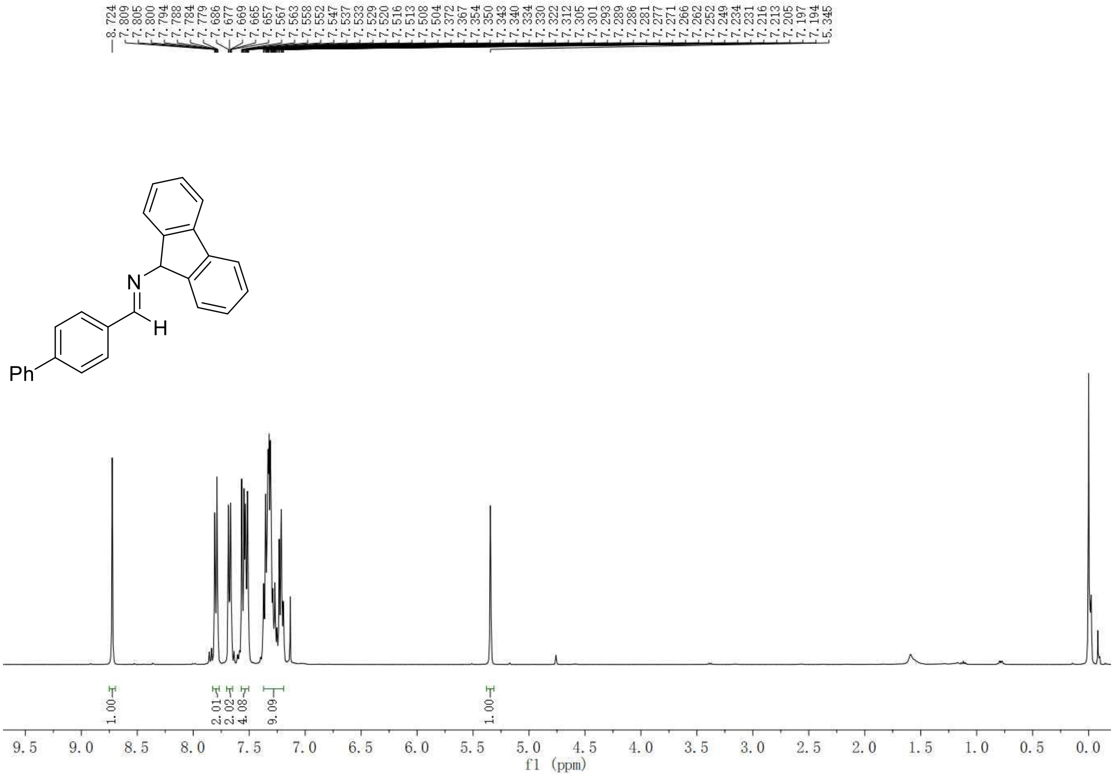

Figure S7. ¹³C{¹H} NMR spectra (100 MHz, Chloroform-*d*) of (*E*)-1-([1,1'-Biphenyl]-4-yl)-*N*-(9*H*-fluoren-9-yl)methanimine (1g).

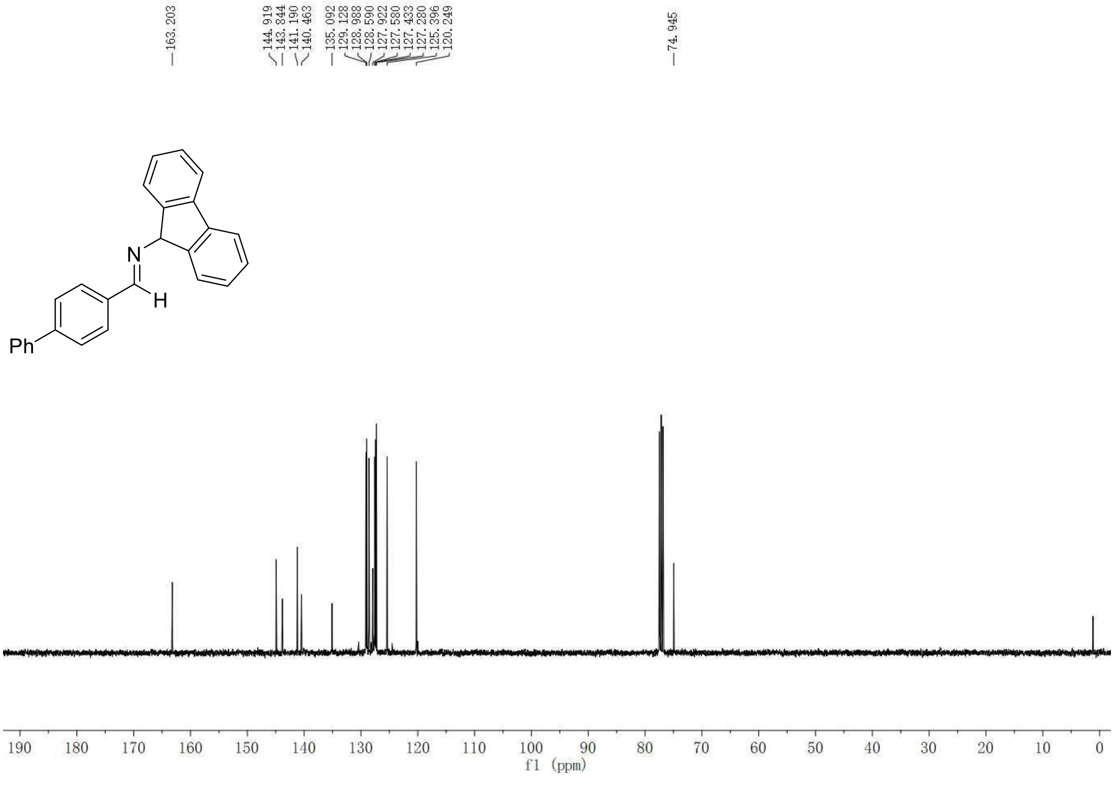

Figure S8. **^{1}H NMR spectra (400 MHz, Chloroform-*d*) of (*E*)-*N*-(9*H*-Fluoren-9-yl)-1-(3-(trifluoromethoxy)phenyl)methanimine (1h).**

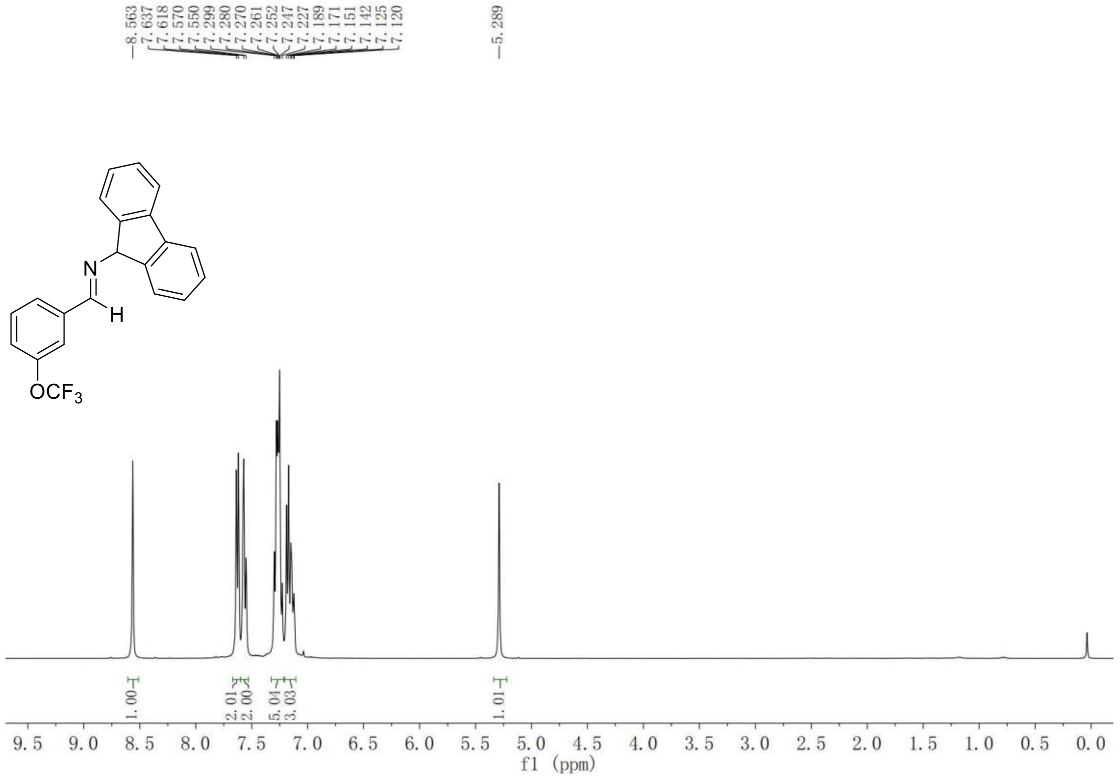

Figure S9. **^{13}C{^{1}H} NMR spectra (100 MHz, Chloroform-*d*) of (*E*)-*N*-(9*H*-Fluoren-9-yl)-1-(3-(trifluoromethoxy)phenyl)methanimine (1h).**

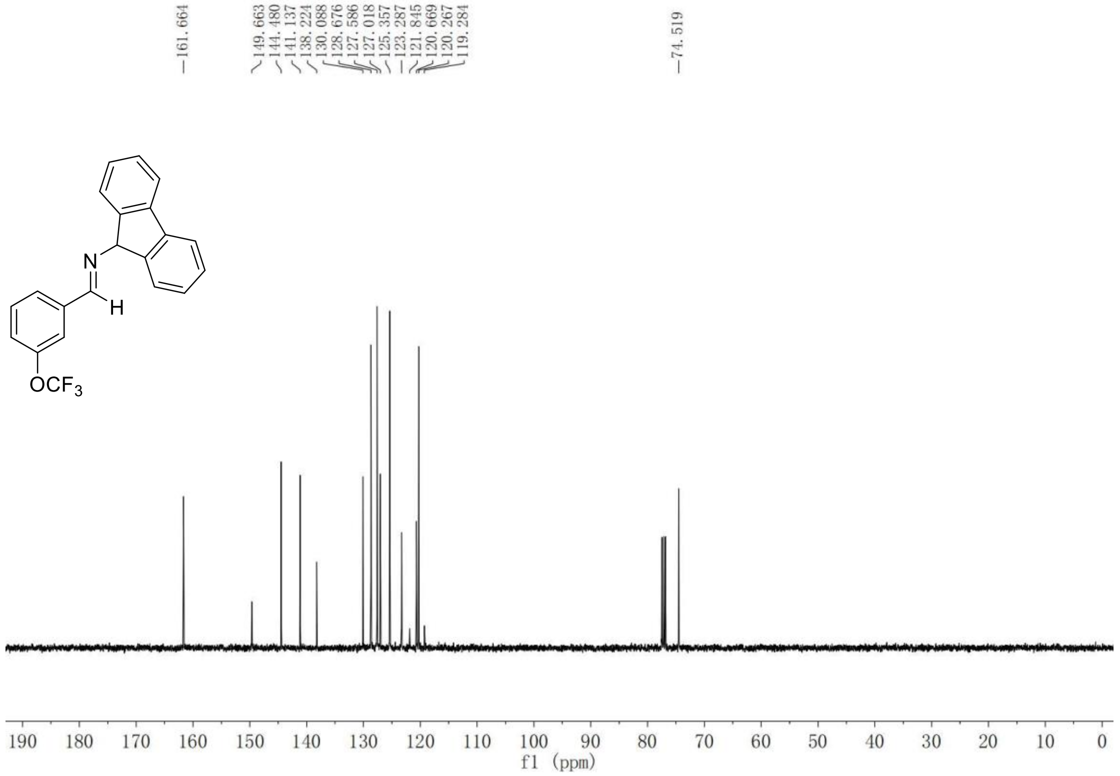

Figure S10. ^{19}F NMR spectra (376 MHz, Chloroform-*d*) of (*E*)-*N*-(9*H*-Fluoren-9-yl)-1-(3-(trifluoromethoxy)phenyl)methanimine (1h).

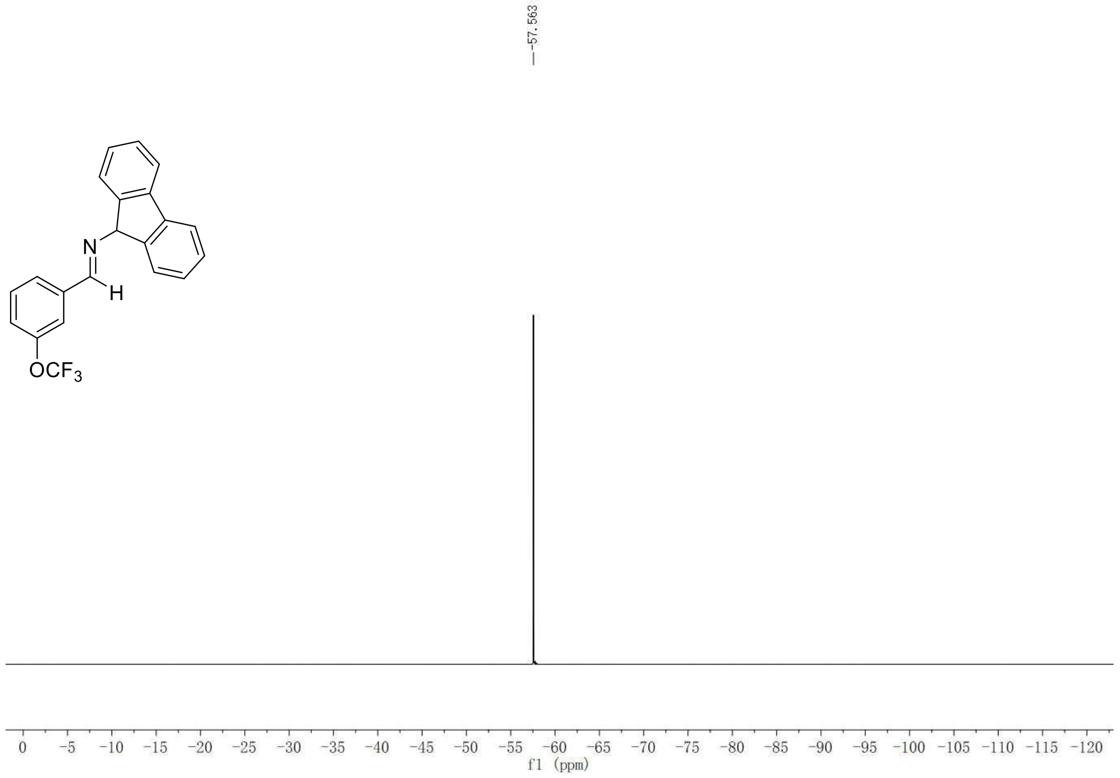

Figure S11. **^{1}H NMR spectra (400 MHz, Chloroform-*d*) of (*E*)-1-(3,4-Dimethoxyphenyl)-*N*-(9*H*-fluoren-9-yl)methanimine (1i).**

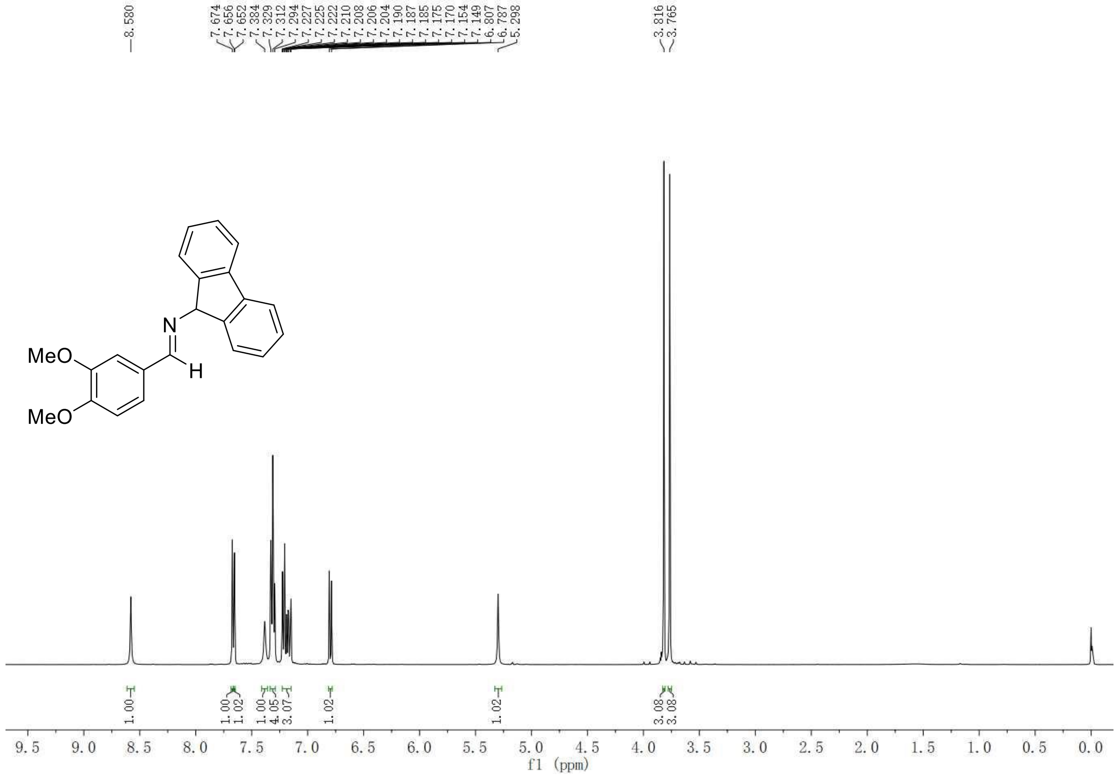

Figure S12. **^{13}C{^{1}H} NMR spectra (100 MHz, Chloroform-*d*) of (*E*)-1-(3,4-Dimethoxyphenyl)-*N*-(9*H*- fluoren-9-yl)methanimine (1i).**

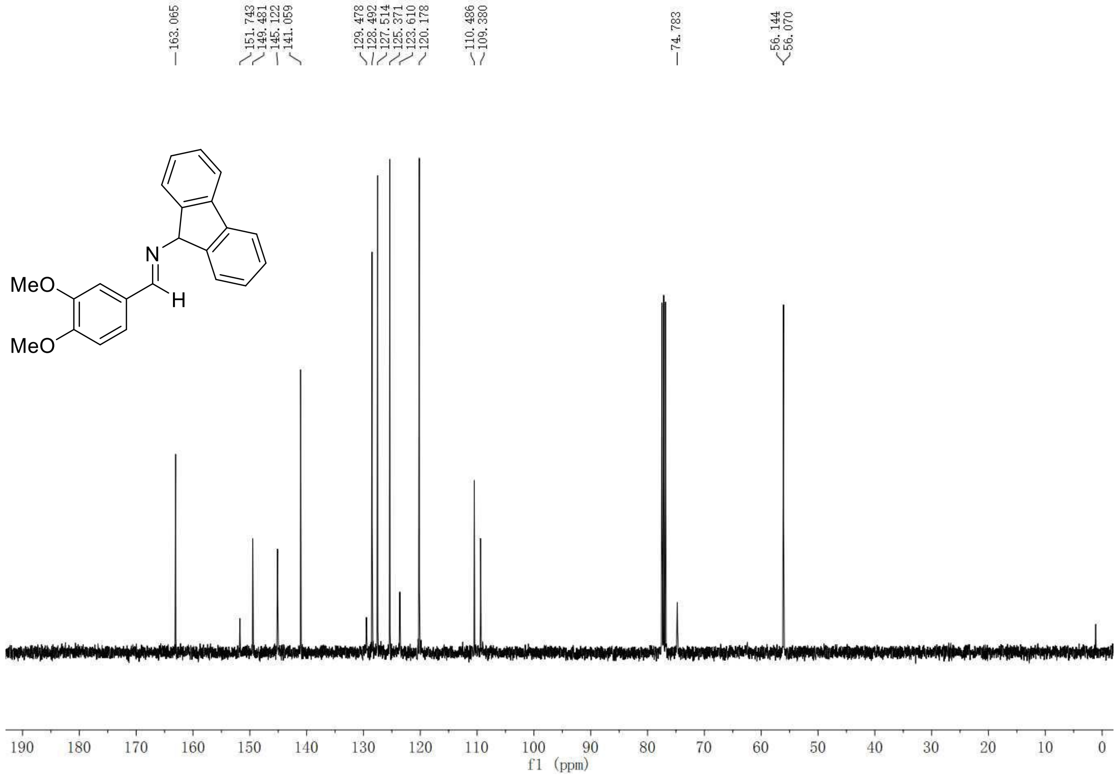

Figure S13. **^{1}H NMR spectra (400 MHz, Chloroform-*d*) of (*E*)-*N*-(9*H*-Fluoren-9-yl)-1-(*o*-tolyl) methanimine (1j).**

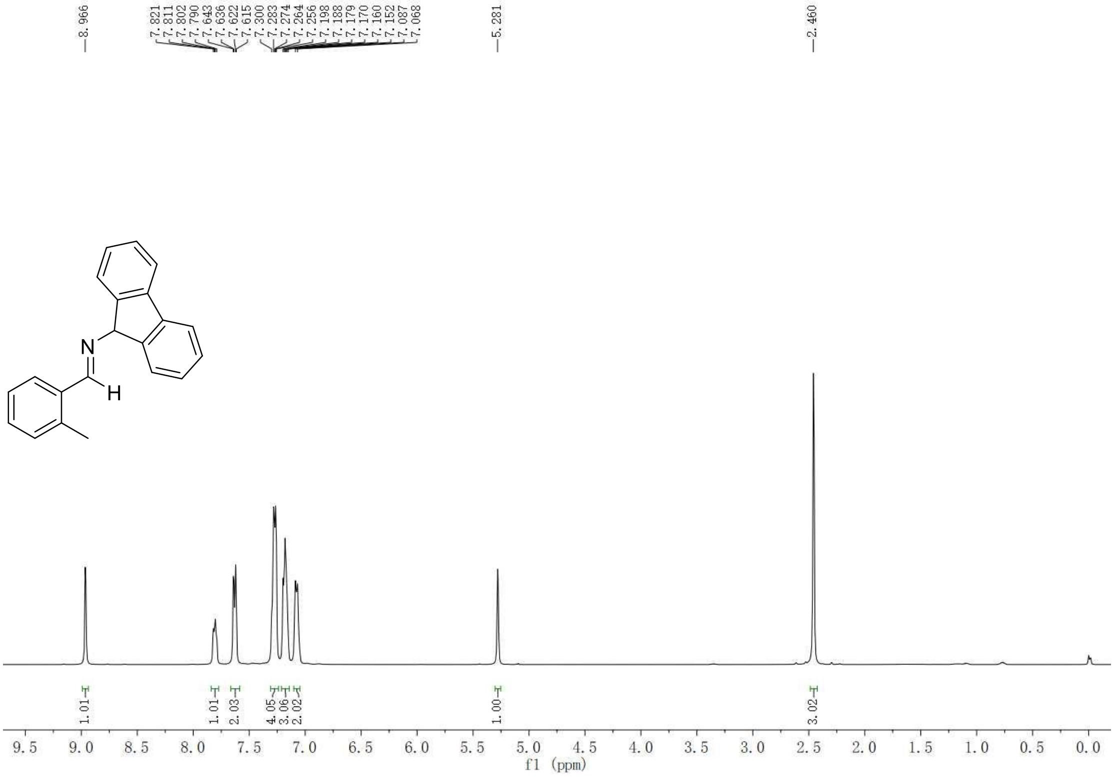

Figure S14. **^{13}C{^{1}H} NMR spectra (100 MHz, Chloroform-*d*) of (*E*)-*N*-(9*H*-Fluoren-9-yl)-1-(*o*-tolyl) methanimine (1j).**

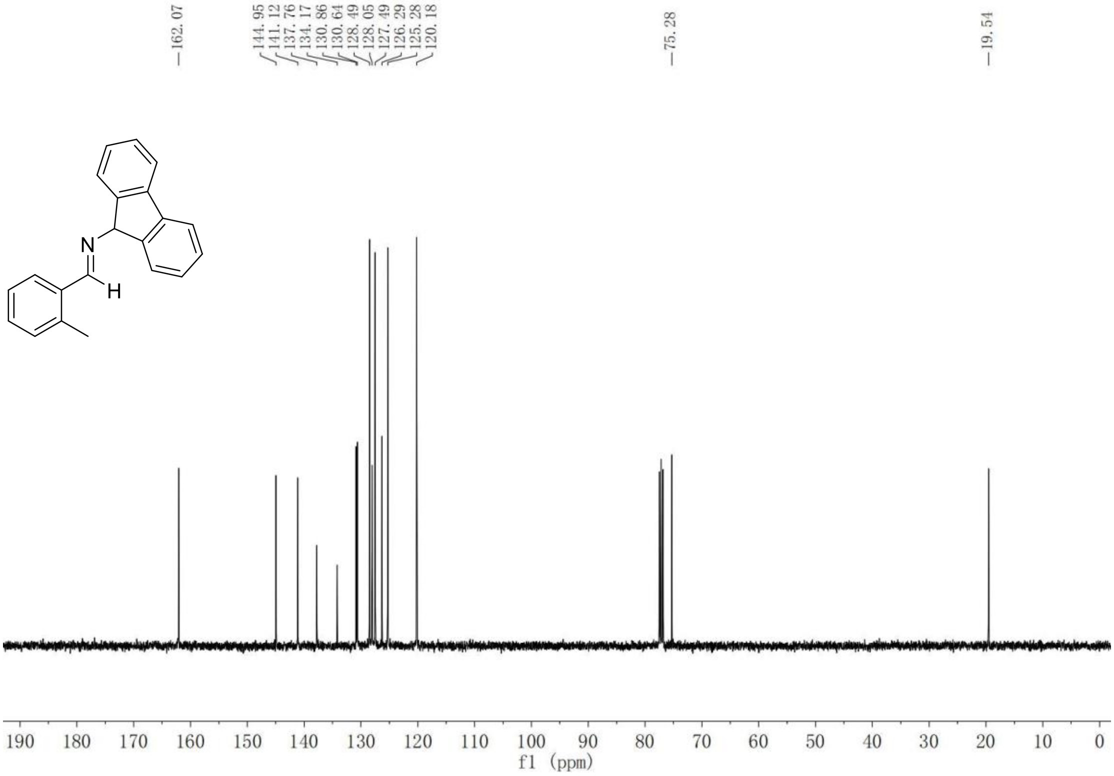

Figure S15. **^{1}H NMR spectra (400 MHz, Chloroform-*d*) of (*E*)-*N*-(9*H*-Fluoren-9-yl)-1-(naphthalen-1-yl)methanimine (1k).**

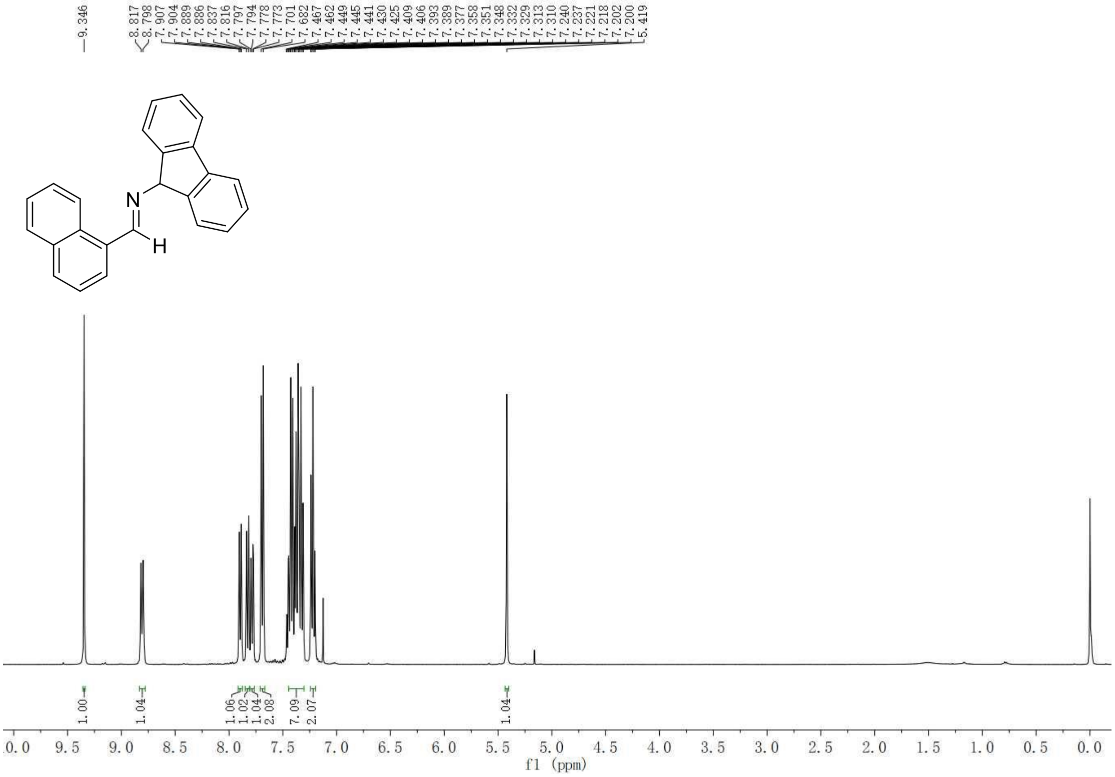

Figure S16. **^{13}C{^{1}H} NMR spectra (100 MHz, Chloroform-*d*) of (*E*)-*N*-(9*H*-Fluoren-9-yl)-1-(naphthalen- 1-yl)methanimine (1k).**

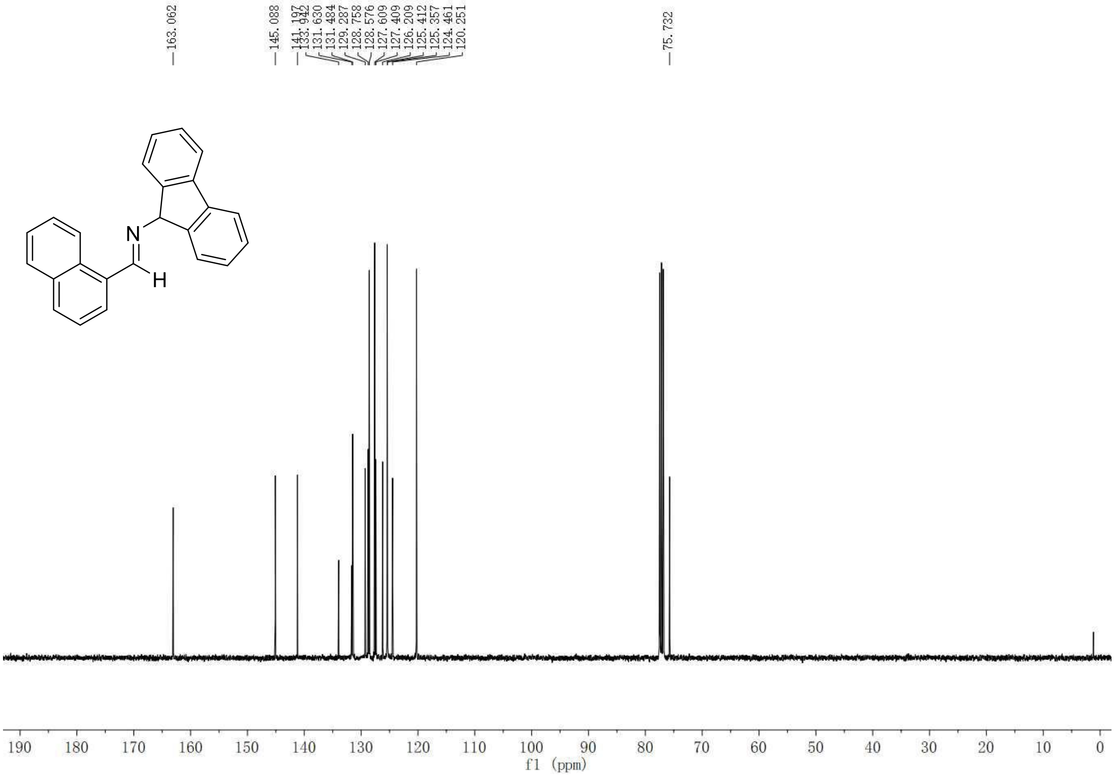

Figure S17. **^{1}H NMR spectra (400 MHz, Chloroform-*d*) of (*E*)-1-(2,3-Dihydrobenzofuran-5-yl)-*N*-(9*H*-fluoren-9-yl)methanimine (1l).**

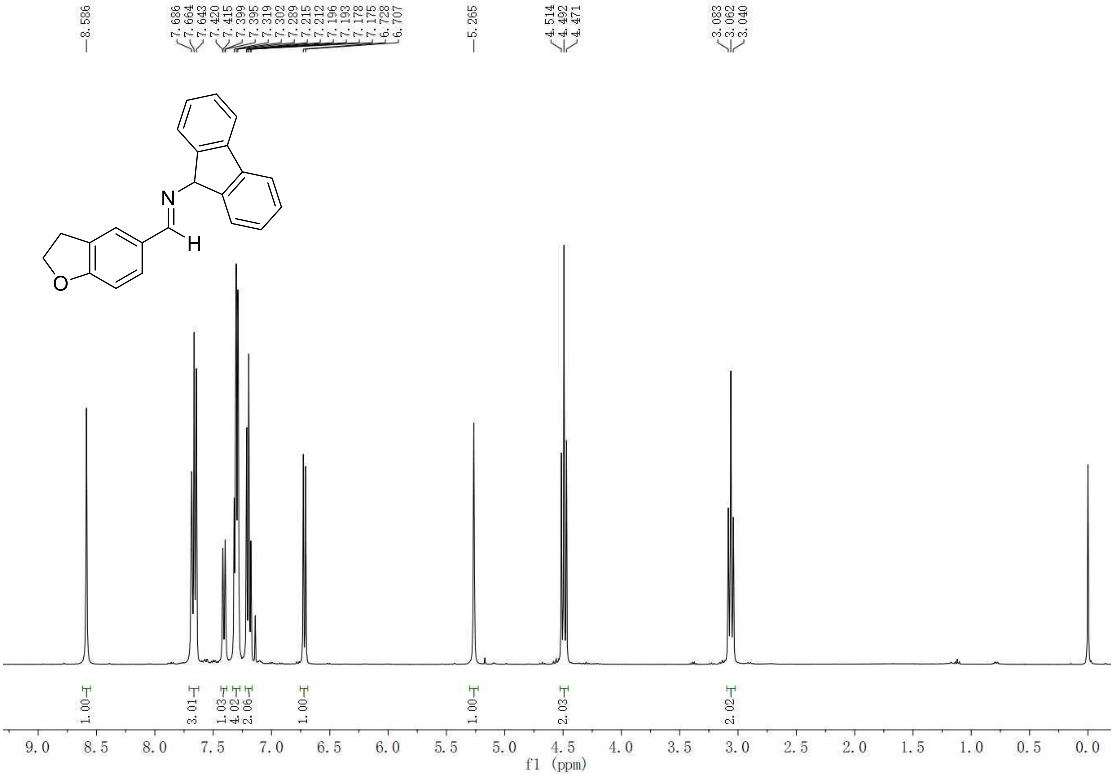

Figure S18. **^{13}C{^{1}H} NMR spectra (100 MHz, Chloroform-*d*) of (*E*)-1-(2,3-Dihydrobenzofuran-5-yl)-*N*-(9*H*-fluoren-9-yl)methanimine (1l).**

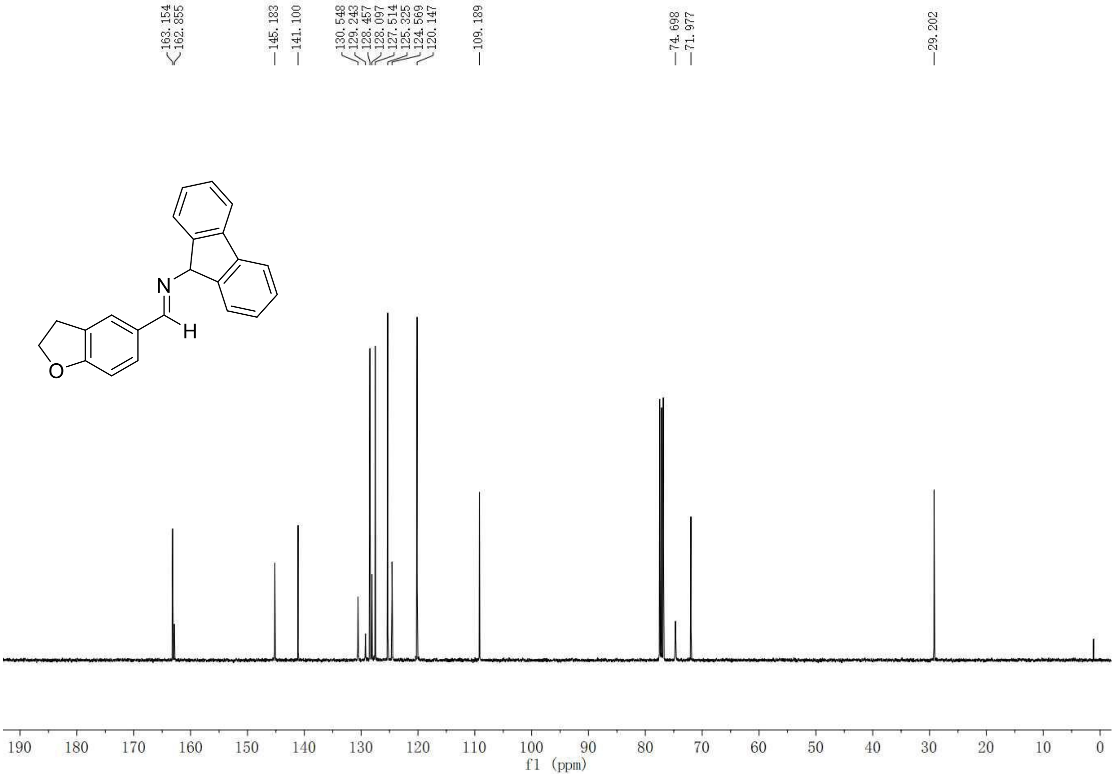

Figure S19. ^{1}H **NMR spectra (400 MHz, Chloroform-*d*) of (*E*)-1-(2,6-Dimethoxypyridin-3-yl)-*N*-(9*H*-fluoren-9-yl)methanimine (1m).**

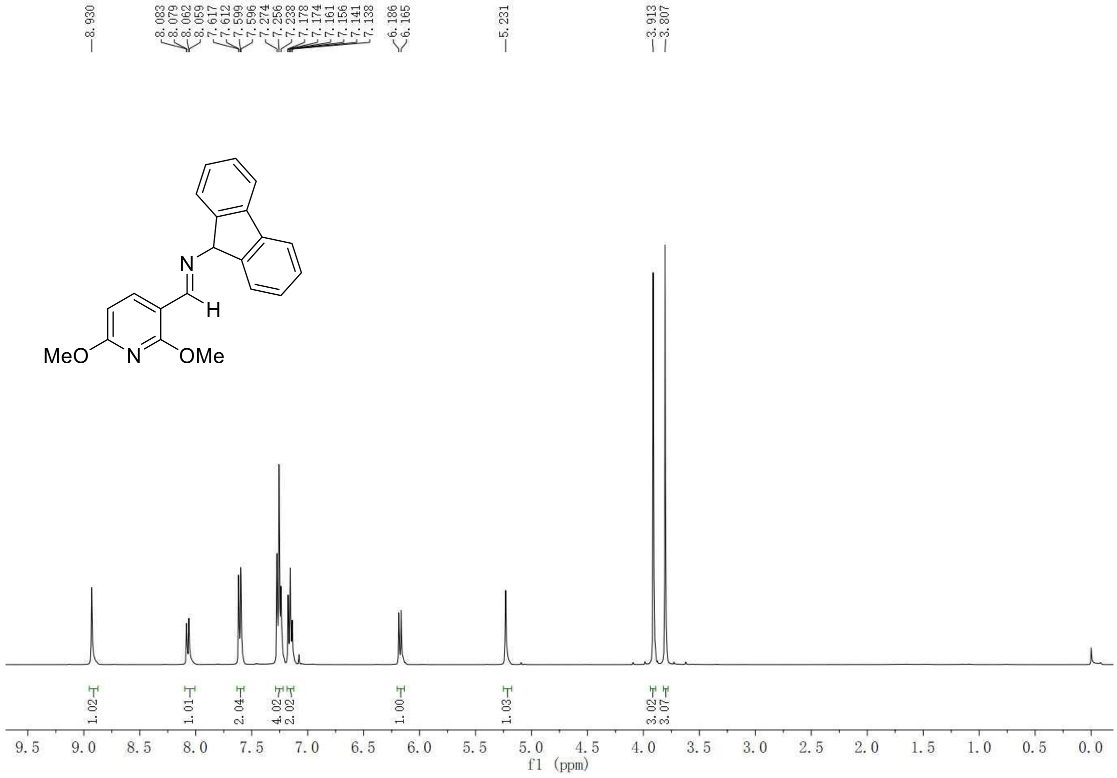

Figure S20. $^{13}C\{^{1}H\}$ **NMR spectra (100 MHz, Chloroform-*d*) of (*E*)-1-(2,6-Dimethoxypyridin-3-yl)-*N*-(9*H*-fluoren-9-yl)methanimine (1m).**

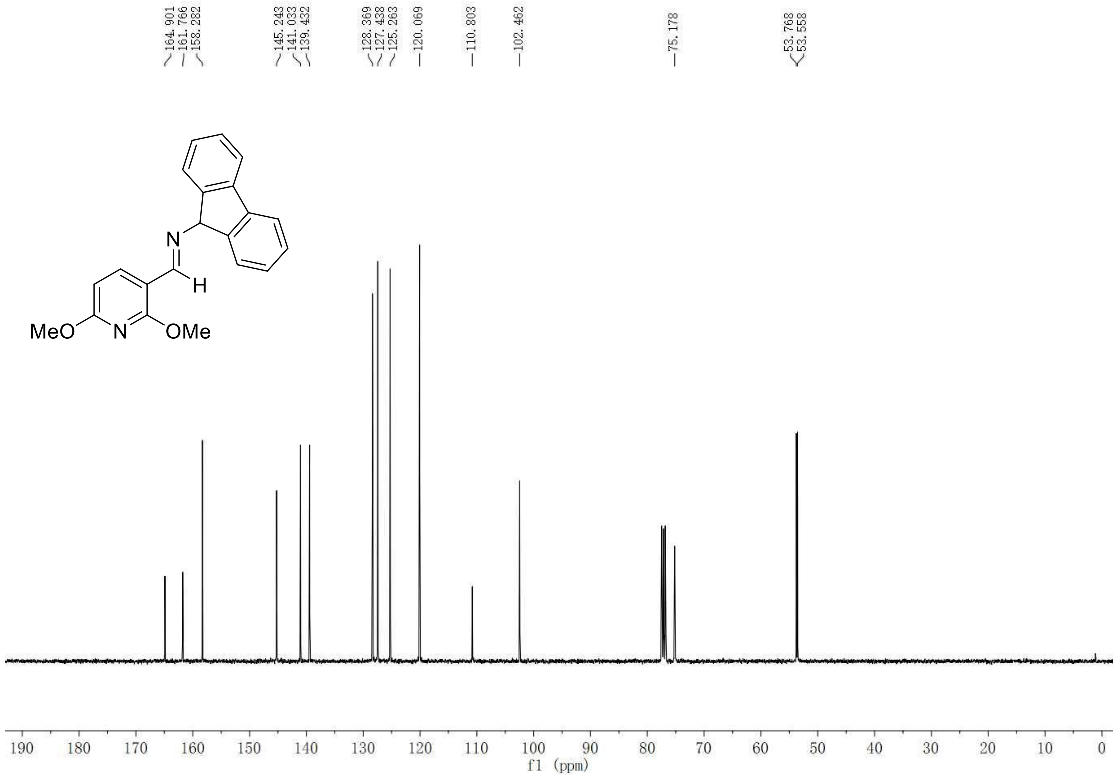

Figure S21. ¹H NMR spectra (400 MHz, Chloroform-*d*) of 1-(2-Bromoallyl)pyrrolidine (2g).

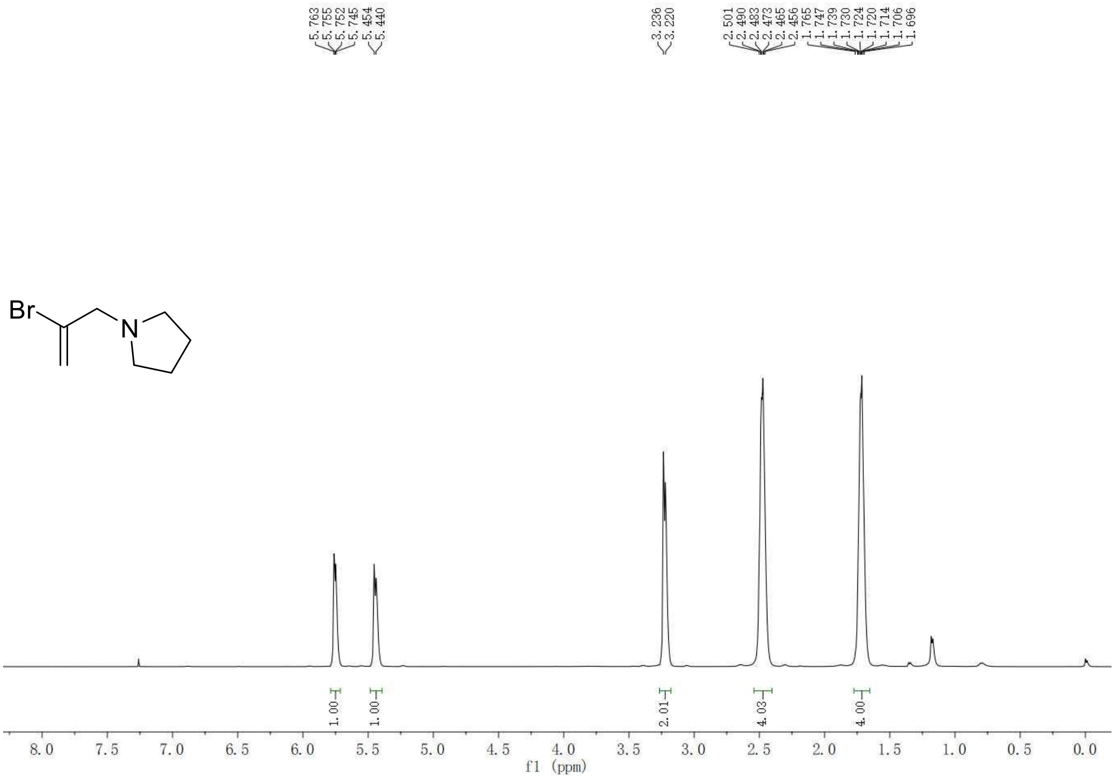

Figure S22. ¹³C{¹H} NMR spectra (100 MHz, Chloroform-*d*) of 1-(2-Bromoallyl)pyrrolidine (2g).

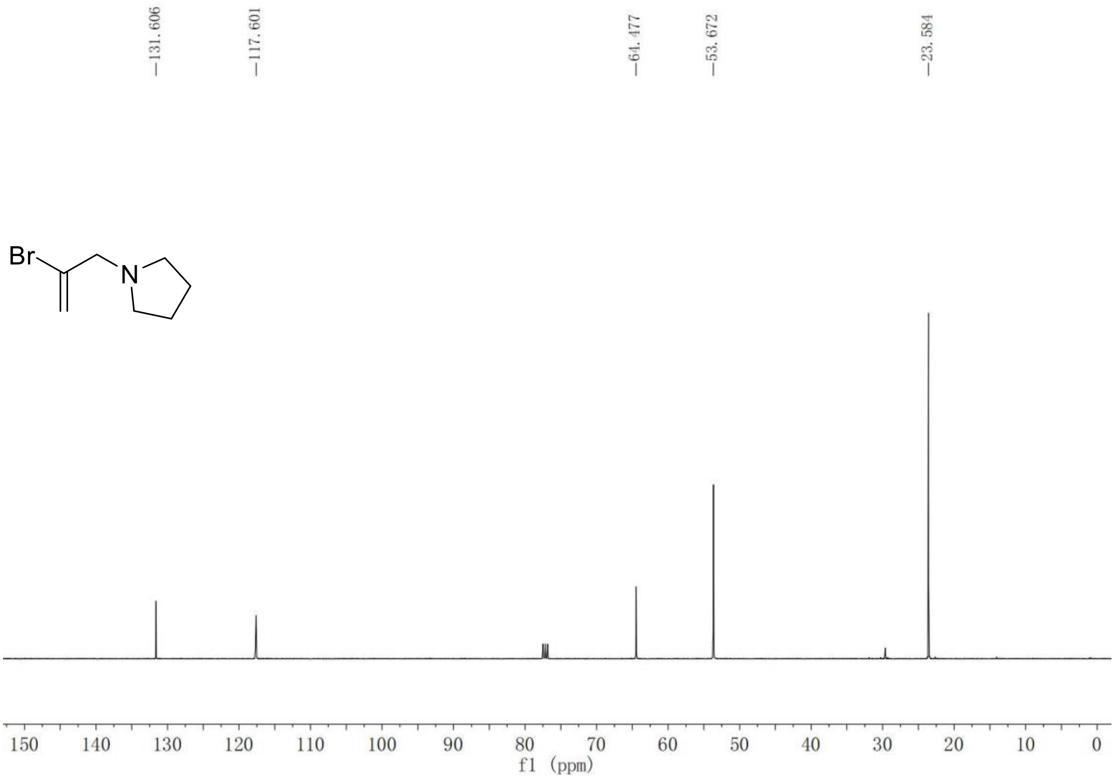

Figure S23. ^{1}H NMR spectra (400 MHz, Chloroform-*d*) of 1-(2-Bromoallyl)piperidine (2h).

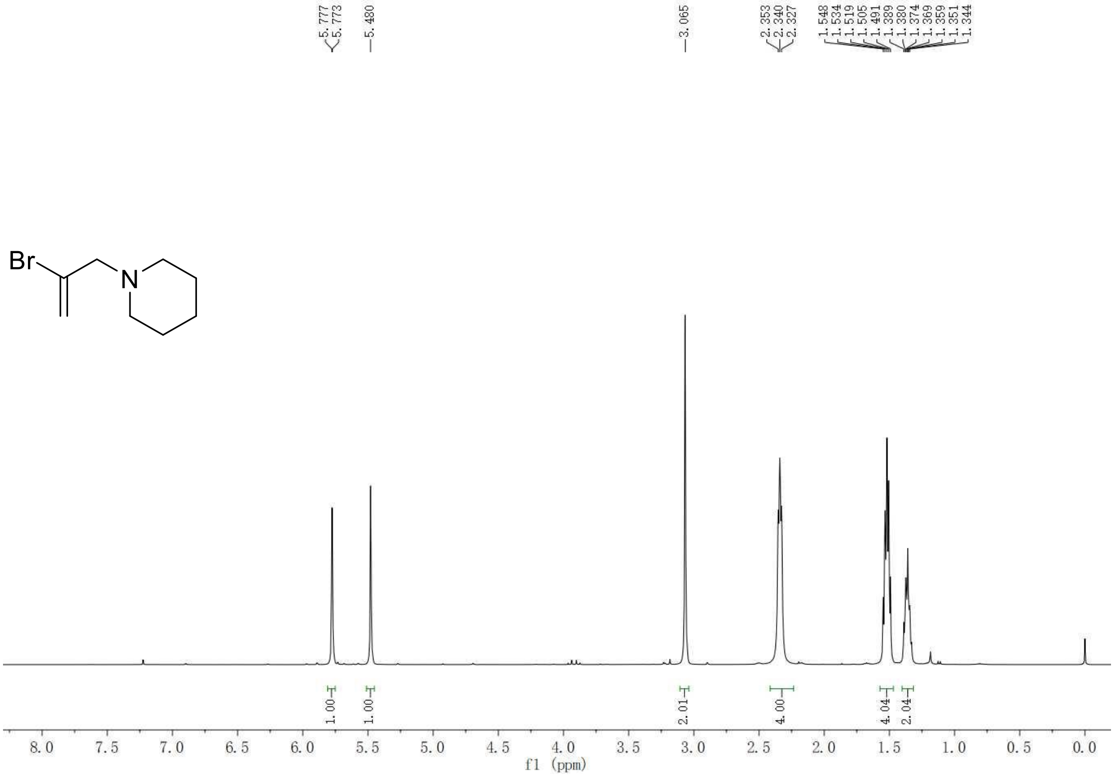

Figure S24. $^{13}C\{^1H\}$ NMR spectra (100 MHz, Chloroform-*d*) of 1-(2-Bromoallyl)piperidine (2h).

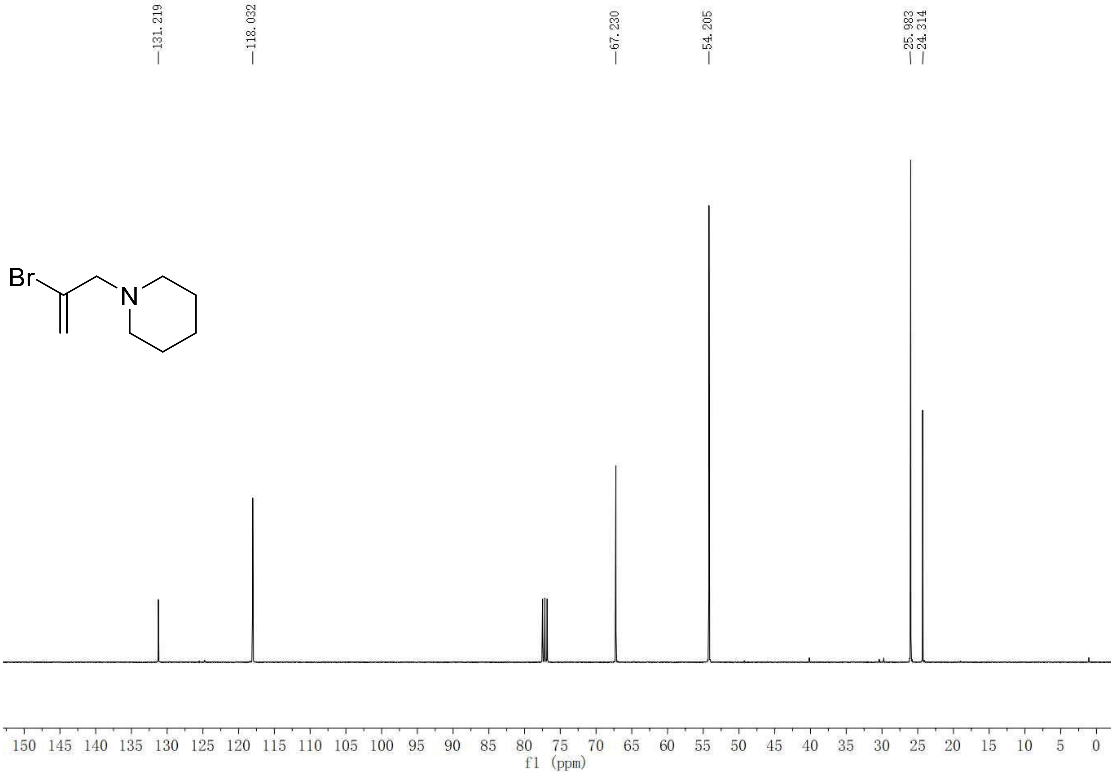

Figure S25. ^{1}H NMR spectra (400 MHz, Chloroform-*d*) of 2-(2-Bromoallyl)-1,2,3,4-tetrahydroisoquinoline (2i).

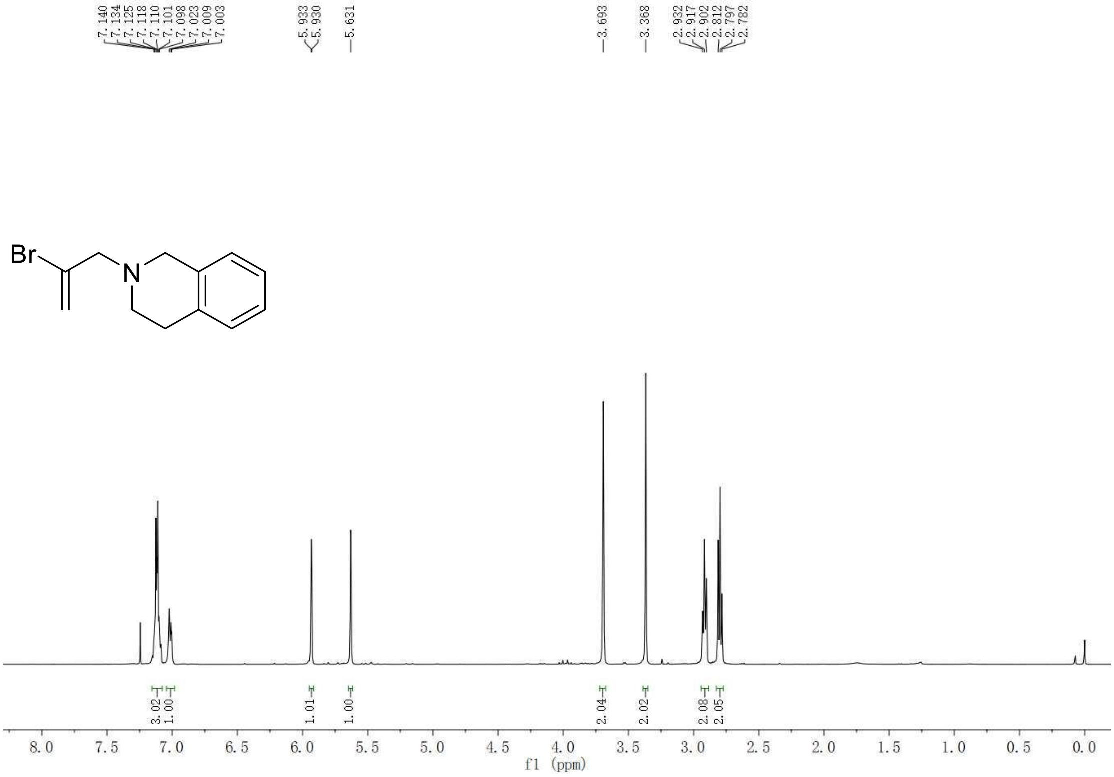

Figure S26. ^{13}C{^{1}H} NMR spectra (100 MHz, Chloroform-*d*) of 2-(2-Bromoallyl)-1,2,3,4-tetrahydroisoquinoline (2i).

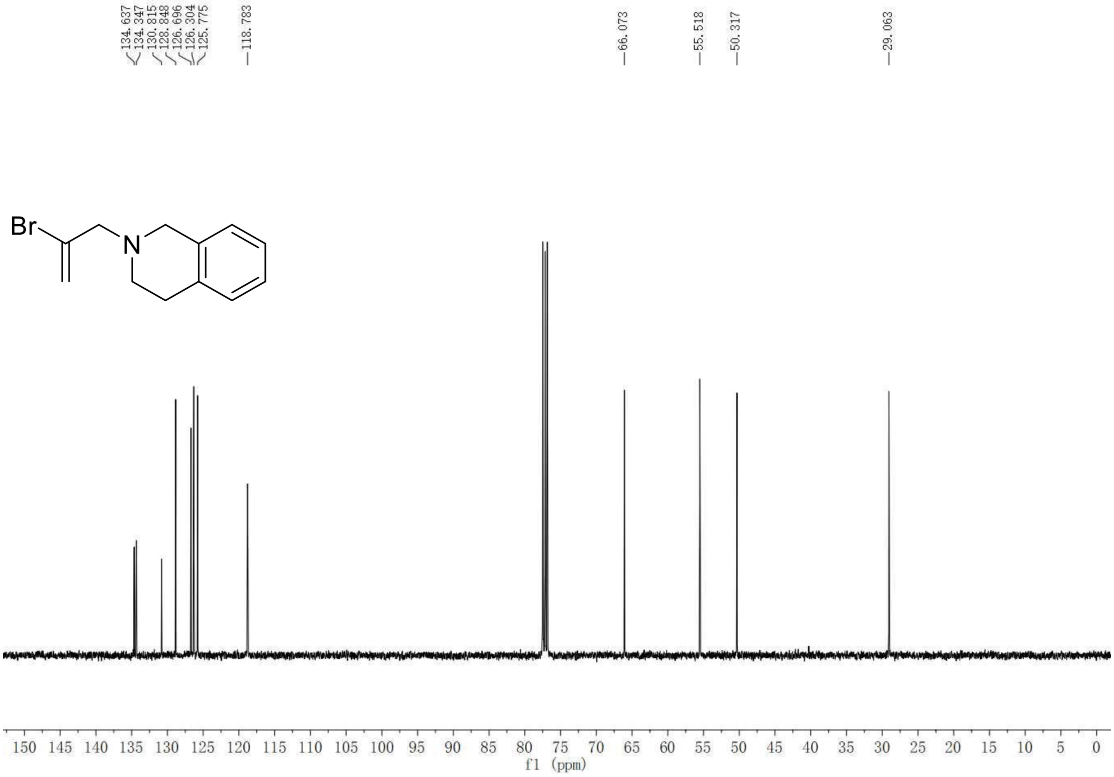

Figure S27. ^{1}H NMR spectra (400 MHz, Chloroform-*d*) of (*R*)-*N*-(2-Methyl-1-phenylallyl)-9*H*-fluoren-9-imine (3aa).

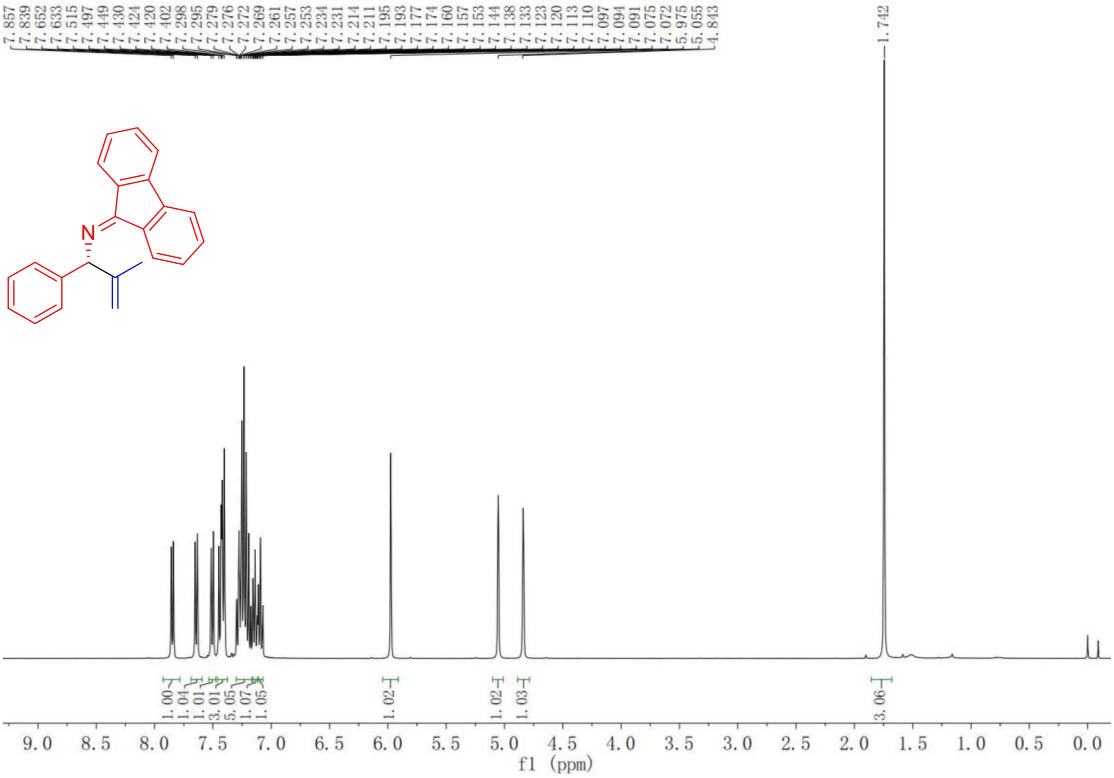

Figure S28. $^{13}C\{^{1}H\}$ NMR spectra (100 MHz, Chloroform-*d*) of (*R*)-*N*-(2-Methyl-1-phenylallyl)-9*H*-fluoren-9-imine (3aa).

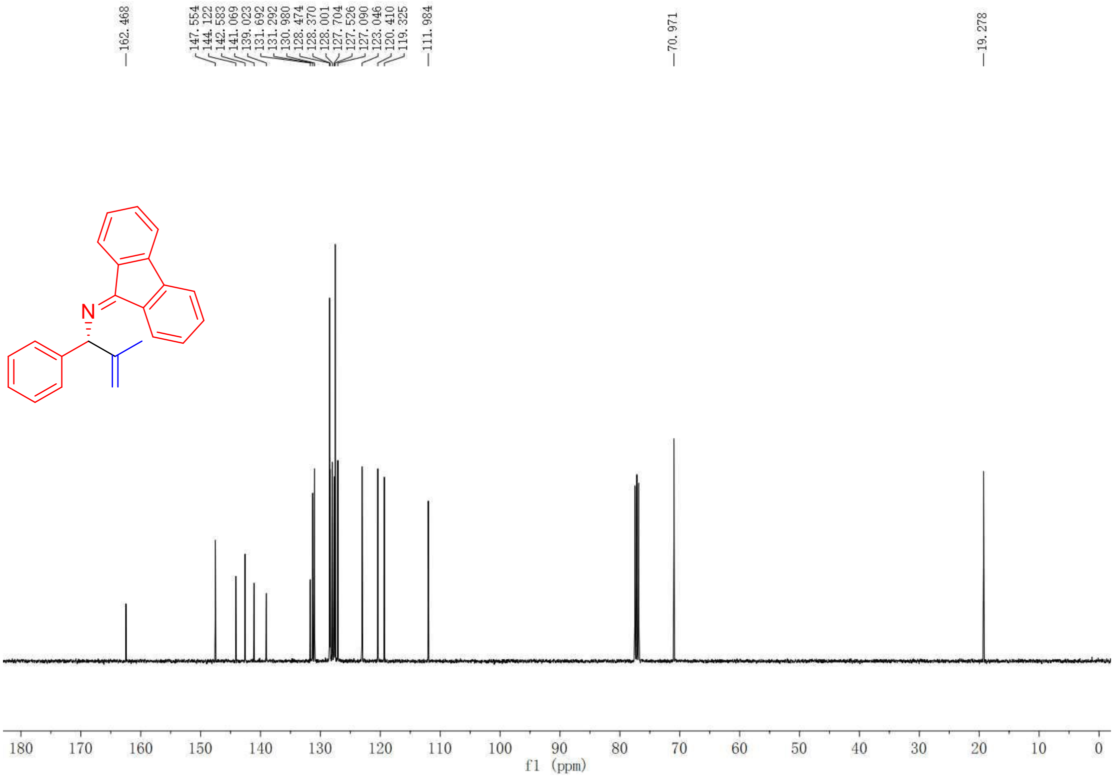

Figure S29. HPLC Chromatography of the Racemic *N*-(2-Methyl-1-phenylallyl)-9*H*-fluoren-9-imine (3aa).

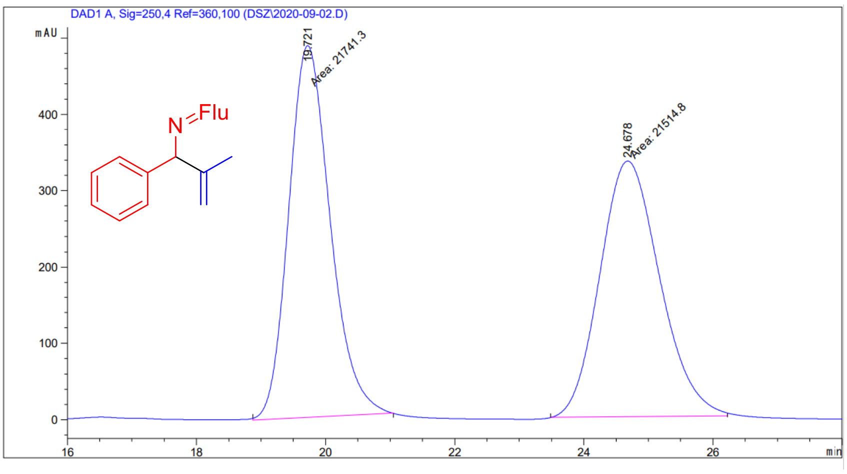

Signal 1: DAD1 A, Sig=250,4 Ref=360,100

Peak #	RetTime [min]	Type	Width [min]	Area [mAU*s]	Height [mAU]	Area %
1	19.721	MM	0.7448	2.17413e4	486.49799	50.2618
2	24.678	MM	1.0724	2.15148e4	334.36270	49.7382
Totals :				4.32562e4	820.86069	

Figure S30. HPLC Chromatography of (*R*)-*N*-(2-Methyl-1-phenylallyl)-9*H*-fluoren-9-imine (3aa).

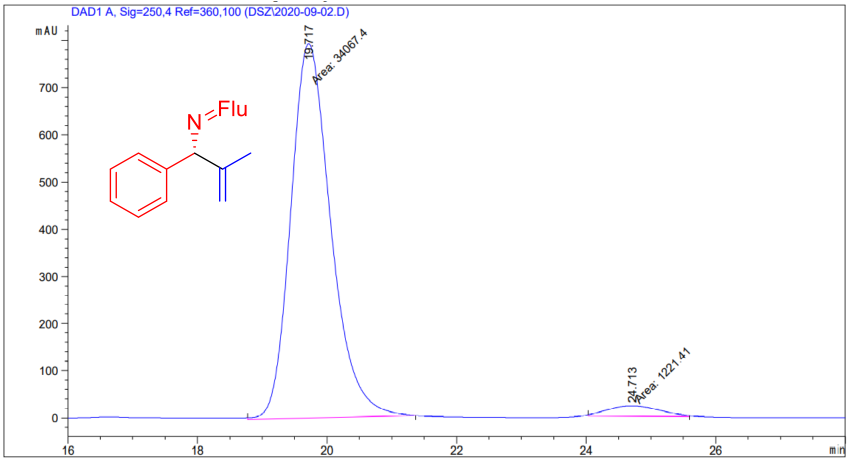

Signal 1: DAD1 A, Sig=250,4 Ref=360,100

Peak #	RetTime [min]	Type	Width [min]	Area [mAU*s]	Height [mAU]	Area %
1	19.717	MM	0.7166	3.40674e4	792.30365	96.5388
2	24.713	MM	0.9218	1221.40576	22.08405	3.4612
Totals :				3.52888e4	814.38770	

Figure S31. ^{1}H **NMR spectra (400 MHz, Chloroform-*d*) of (*R*)-4-(1-((9*H*-Fluoren-9-ylidene)amino)-2-methylallyl)-*N*,*N*-dimethylaniline (3ba).**

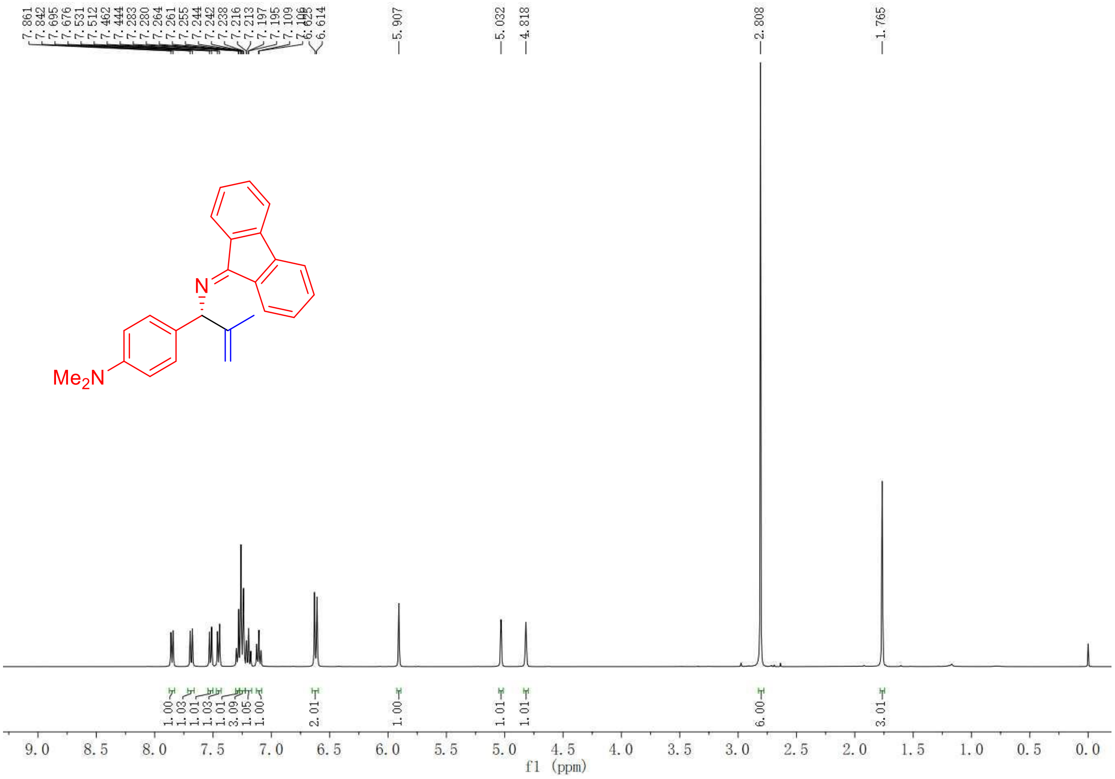

Figure S32. $^{13}C\{^{1}H\}$ **NMR spectra (100 MHz, Chloroform-*d*) of (*R*)-4-(1-((9*H*-Fluoren-9-ylidene)amino)-2-methylallyl)-*N*,*N*-dimethylaniline (3ba).**

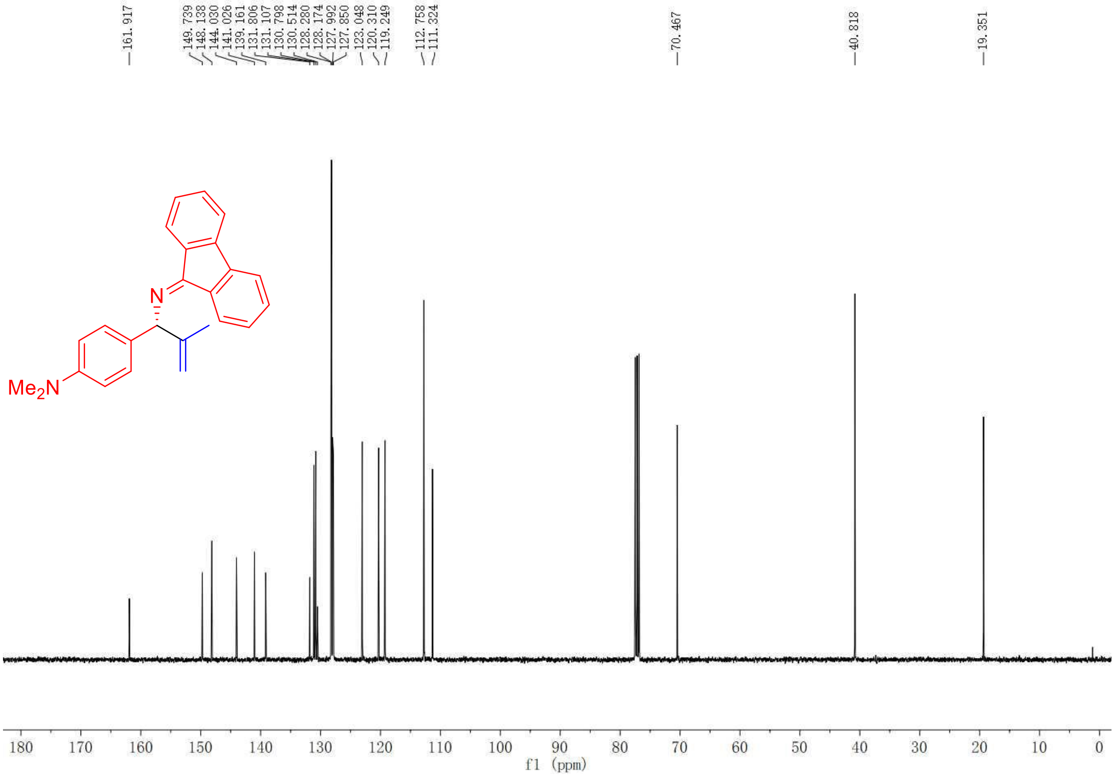

Figure S33. HPLC Chromatography of the Racemic 4-(1-((9*H*-Fluoren-9-ylidene)amino)-2-methylallyl)-*N*,*N*-dimethylaniline (3ba).

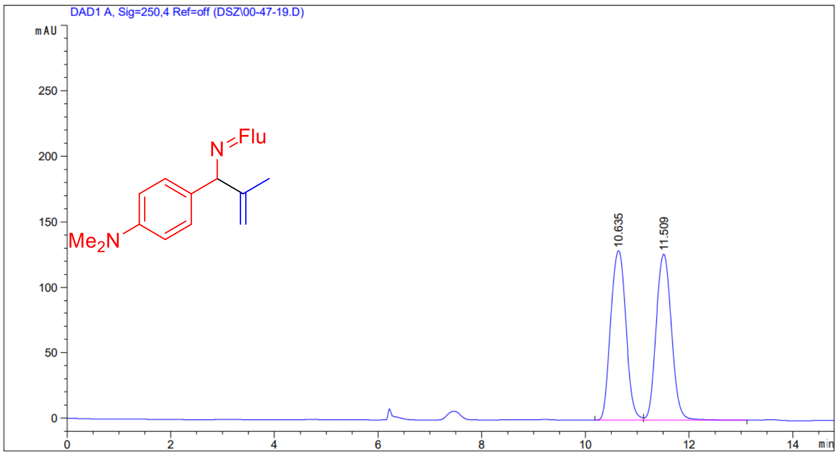

Signal 1: DAD1 A, Sig=250,4 Ref=off

Peak #	RetTime [min]	Type	Width [min]	Area [mAU*s]	Height [mAU]	Area %
1	10.635	BV	0.3294	2640.80225	129.80450	50.2210
2	11.509	VB	0.3284	2617.56006	127.04807	49.7790
Totals :				5258.36230	256.85258	

Figure S34. HPLC Chromatography of (*R*)-4-(1-((9*H*-Fluoren-9-ylidene)amino)-2-methylallyl)-*N*,*N*-dimethylaniline (3ba).

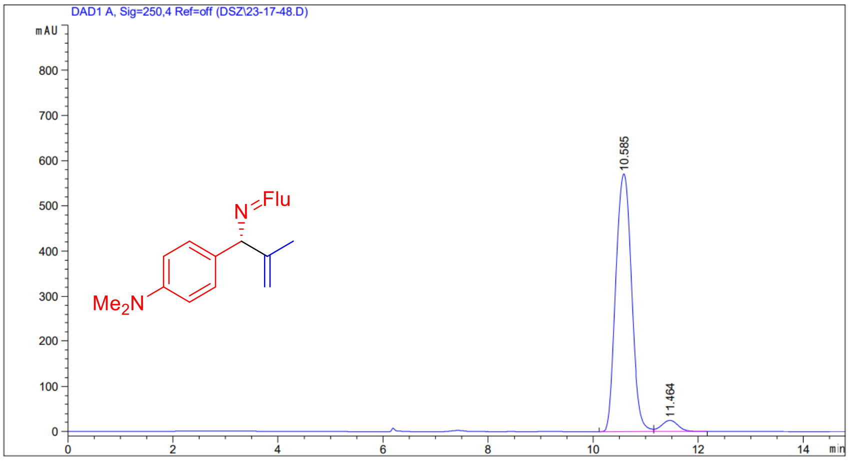

Signal 1: DAD1 A, Sig=250,4 Ref=off

Peak #	RetTime [min]	Type	Width [min]	Area [mAU*s]	Height [mAU]	Area %
1	10.585	BF	0.3298	1.18321e4	570.89478	95.5828
2	11.464	VBA	0.3480	546.79565	24.53609	4.4172
Totals :				1.23789e4	595.43086	

Figure S35. ^{1}H NMR spectra (400 MHz, Chloroform-*d*) of (*R*)-*N*-(1-(4-Fluorophenyl)-2-methylallyl)-9*H*-fluoren-9-imine (3ca).

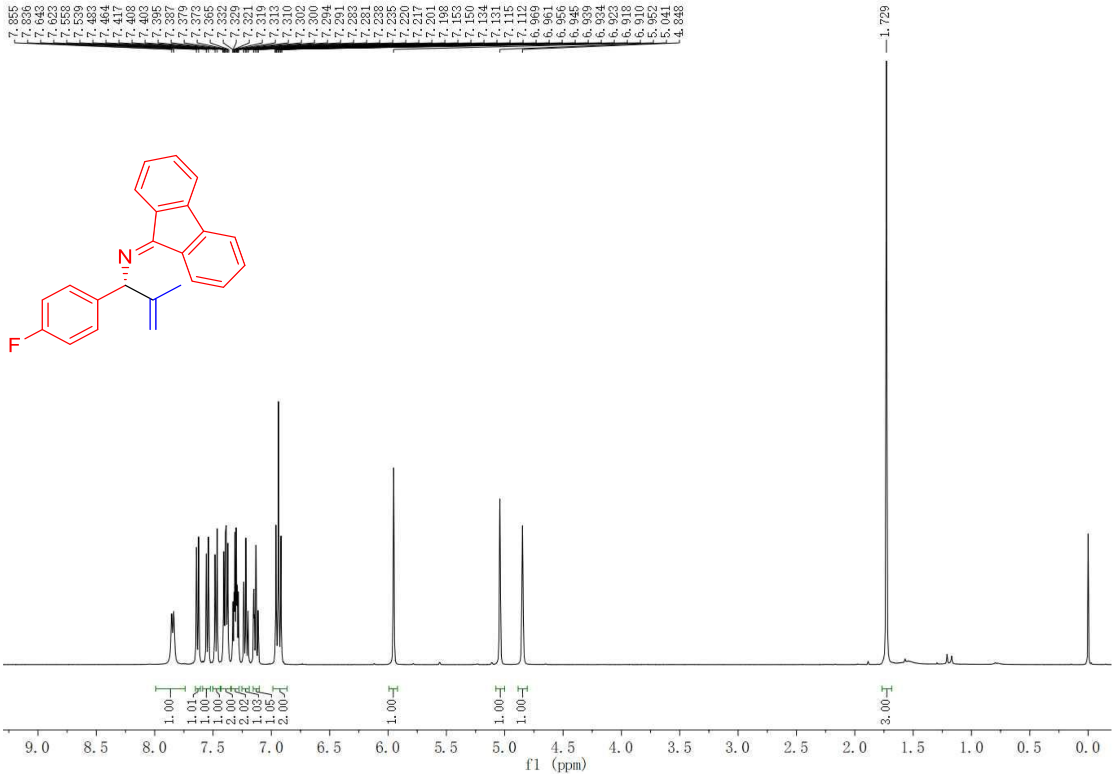

Figure S36. ^{13}C{^{1}H} NMR spectra (100 MHz, Chloroform-*d*) of (*R*)-*N*-(1-(4-Fluorophenyl)-2-methylallyl)-9*H*-fluoren-9-imine (3ca).

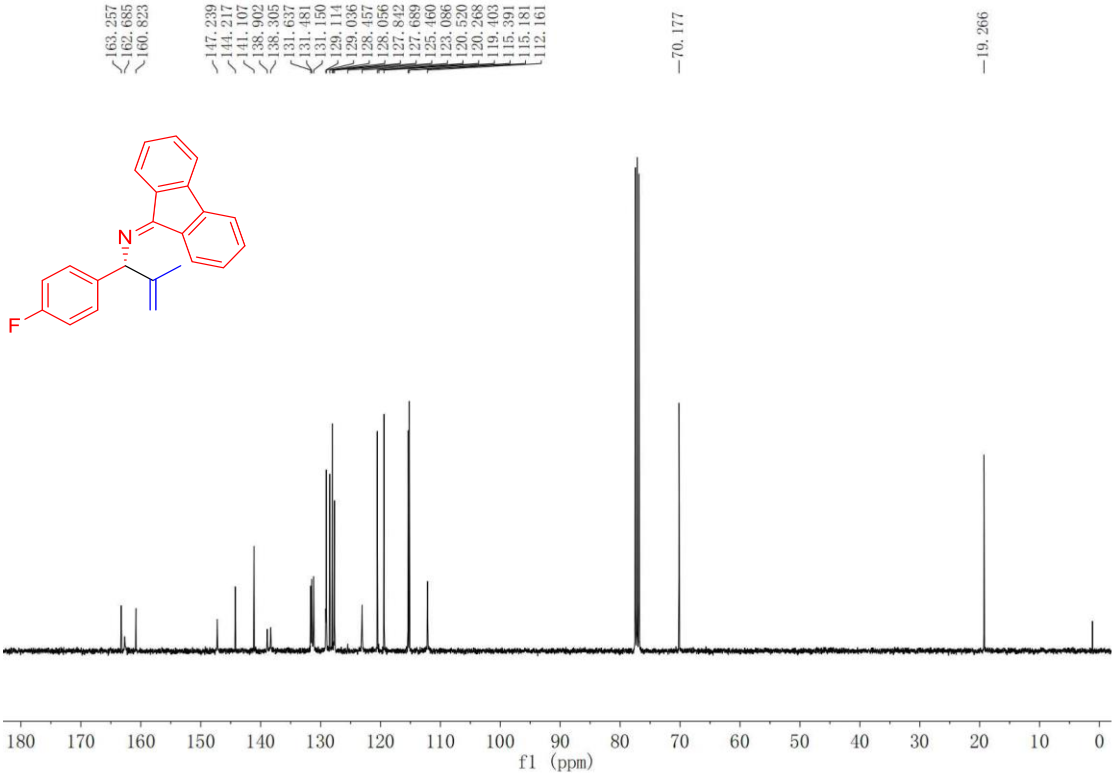

Figure S37. **^{19}F NMR spectra (376 MHz, Chloroform-*d*) of (*R*)-*N*-(1-(4-Fluorophenyl)-2-methylallyl)-9*H*-fluoren-9-imine (3ca).**

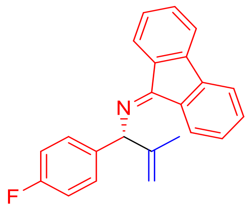

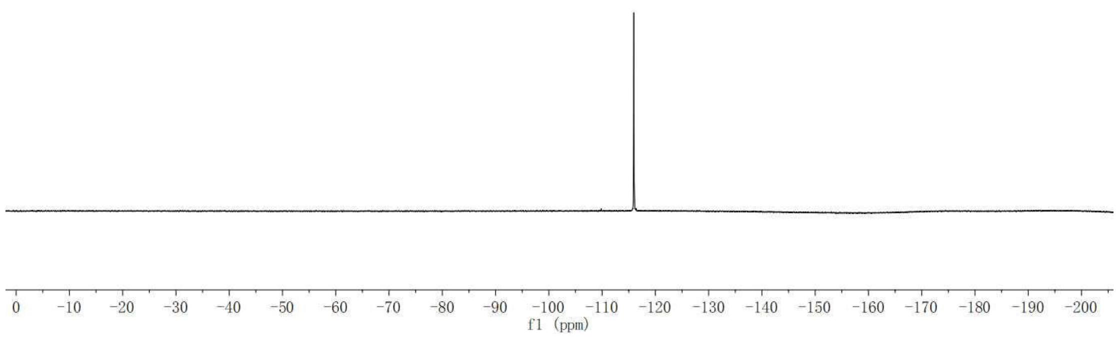

Figure S38. HPLC Chromatography of the Racemic *N*-(1-(4-Fluorophenyl)-2-methylallyl)-9*H*-fluoren-9-imine (3ca).

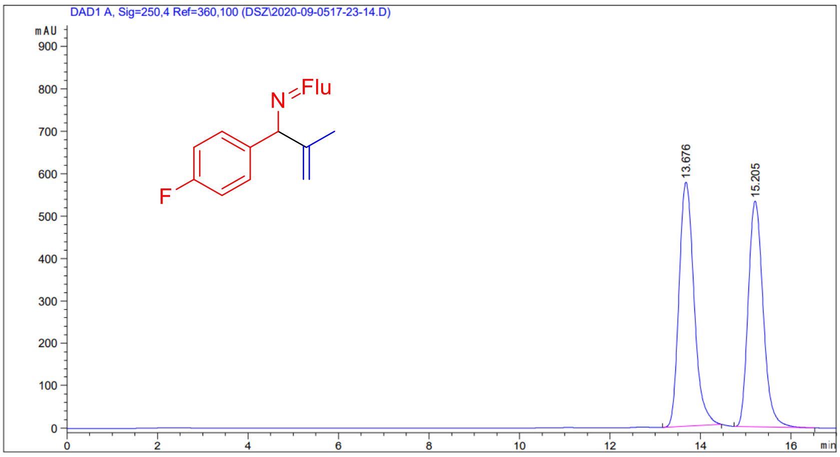

Signal 1: DAD1 A, Sig=250,4 Ref=360,100

Peak #	RetTime [min]	Type	Width [min]	Area [mAU*s]	Height [mAU]	Area %
1	13.676	BBA	0.3597	1.31941e4	575.11237	52.0442
2	15.205	BB	0.3605	1.21576e4	532.24512	47.9558

Totals : 2.53517e4 1107.35748

Figure S39. HPLC Chromatography of (*R*)-*N*-(1-(4-Fluorophenyl)-2-methylallyl)-9*H*-fluoren-9-imine (3ca).

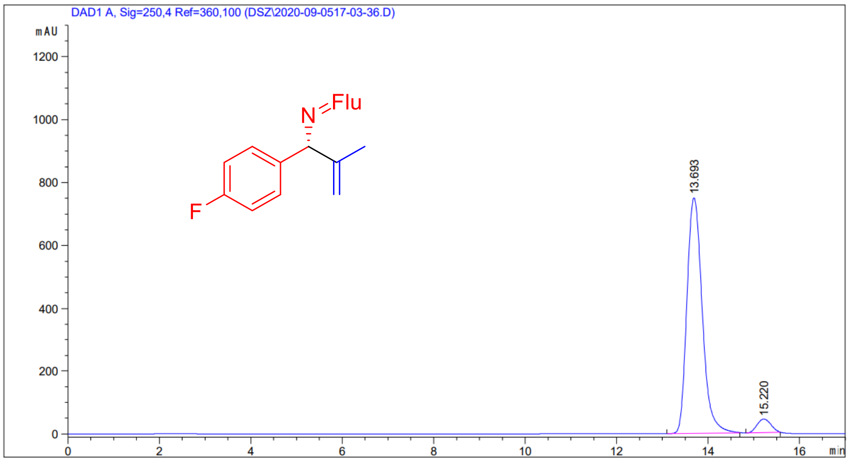

Signal 1: DAD1 A, Sig=250,4 Ref=360,100

Peak #	RetTime [min]	Type	Width [min]	Area [mAU*s]	Height [mAU]	Area %
1	13.693	BF	0.3670	1.74356e4	750.59973	95.1623
2	15.220	BBAS	0.3354	886.35358	42.48063	4.8377

Totals : 1.83219e4 793.08036

Figure S40. ^{1}**H NMR spectra (400 MHz, Chloroform-*d*) of (*R*)-*N*-(1-(4-Chlorophenyl)-2-methylallyl)-9*H*-fluoren-9-imine (3da).**

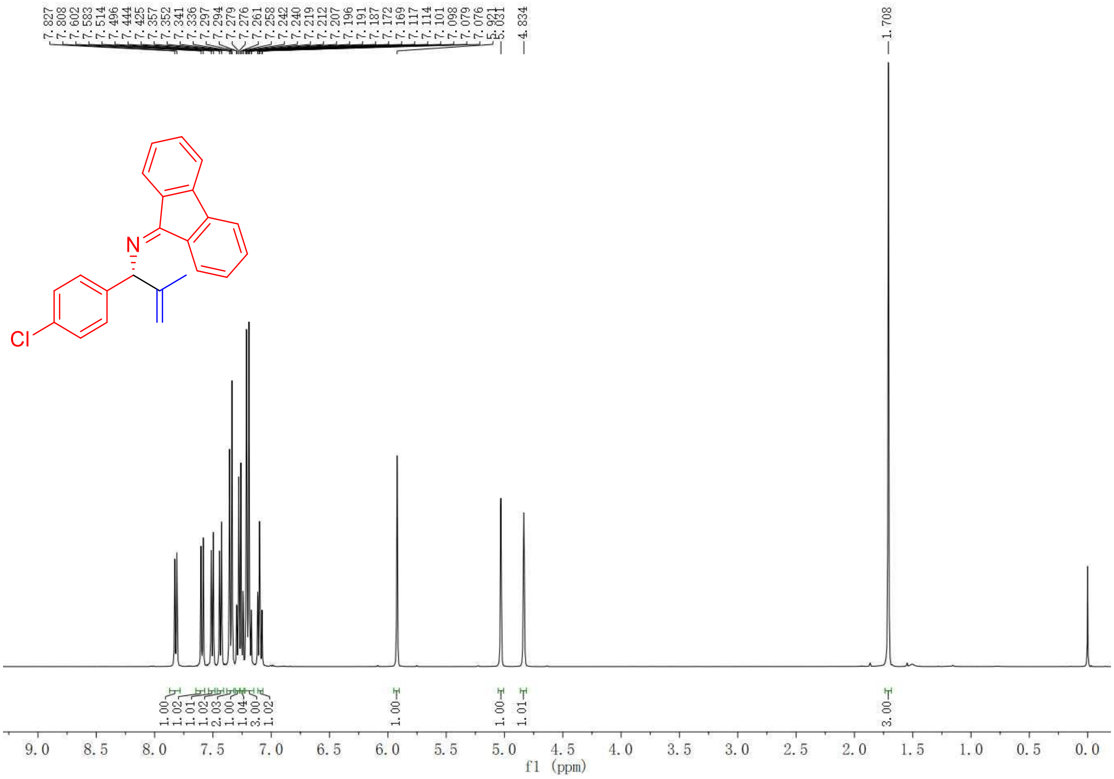

Figure S41. ^{13}**C{**^{1}**H} NMR spectra (100 MHz, Chloroform-*d*) of (*R*)-*N*-(1-(4-Chlorophenyl)-2-methylallyl)-9*H*-fluoren-9-imine (3da).**

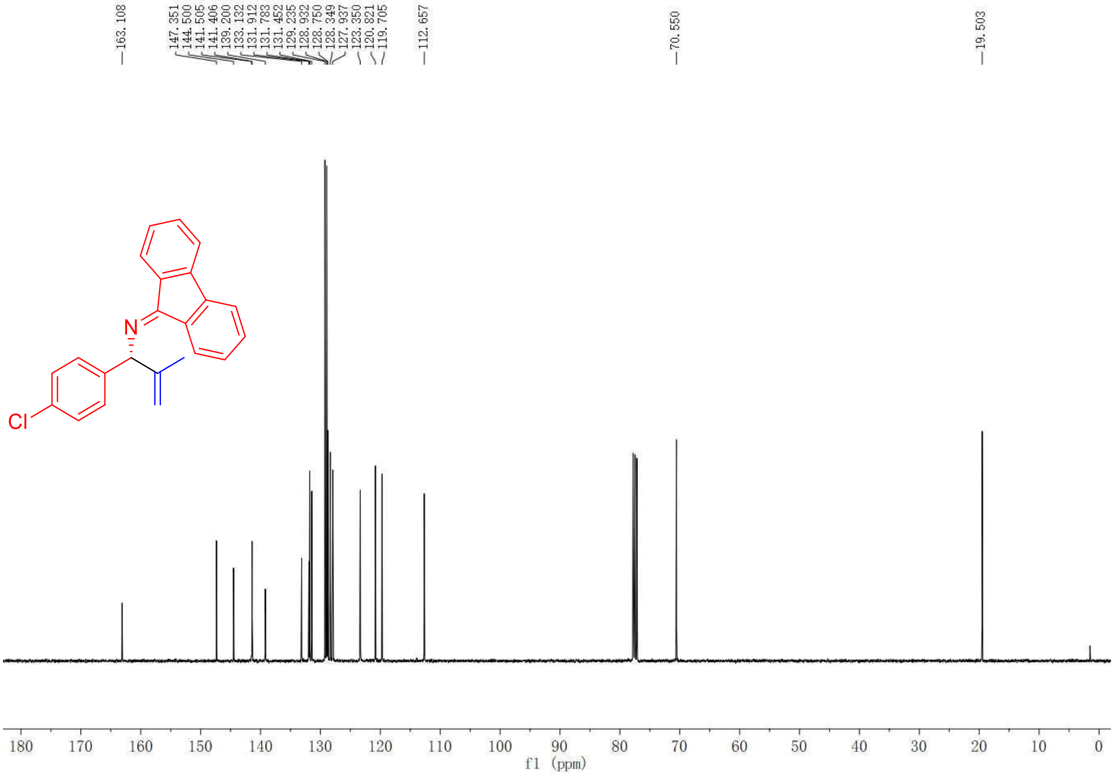

Figure S42. HPLC Chromatography of the Racemic *N*-(1-(4-Chlorophenyl)-2-methylallyl)-

9*H*-fluoren-9-imine (3da).

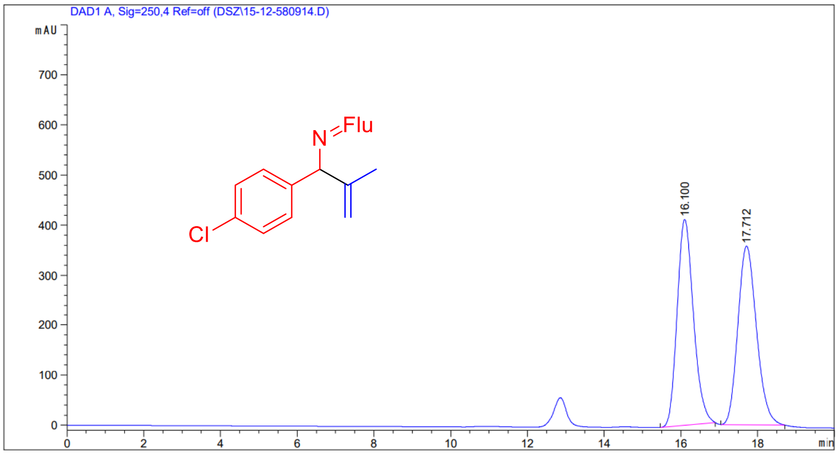

Signal 1: DAD1 A, Sig=250,4 Ref=off

Peak #	RetTime [min]	Type	Width [min]	Area [mAU*s]	Height [mAU]	Area %
1	16.100	BBA	0.4647	1.21650e4	411.72076	50.1813
2	17.712	BBA	0.5223	1.20771e4	357.37451	49.8187
Totals :				2.42422e4	769.09528	

Figure S43. HPLC Chromatography of (*R*)-*N*-(1-(4-Chlorophenyl)-2-methylallyl)-9*H*-fluoren-9-imine (3da).

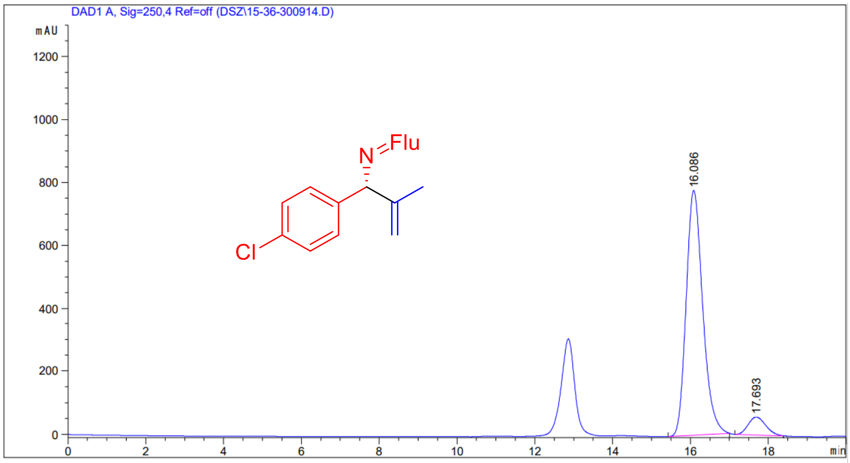

Signal 1: DAD1 A, Sig=250,4 Ref=off

Peak #	RetTime [min]	Type	Width [min]	Area [mAU*s]	Height [mAU]	Area %
1	16.086	BBA	0.4567	2.29554e4	777.01135	92.8253
2	17.693	BBA	0.4916	1774.29065	56.92944	7.1747
Totals :				2.47297e4	833.94080	

Figure S44. **^{1}H NMR spectra (400 MHz, Chloroform-*d*) of (*R*)-*N*-(1-(4-Bromophenyl)-2-methylallyl)-9*H*-fluoren-9-imine (3ea).**

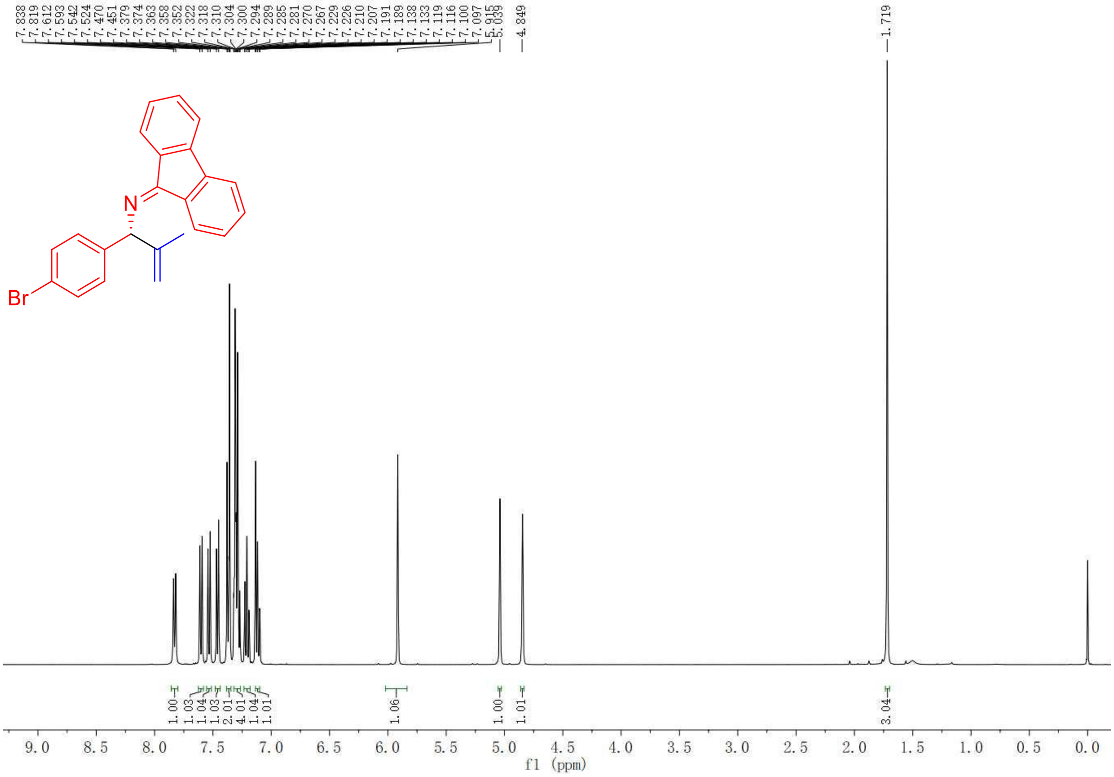

Figure S45. **^{13}C{^{1}H} NMR spectra (100 MHz, Chloroform-*d*) of (*R*)-*N*-(1-(4-Bromophenyl)-2-methylallyl)-9*H*-fluoren-9-imine (3ea).**

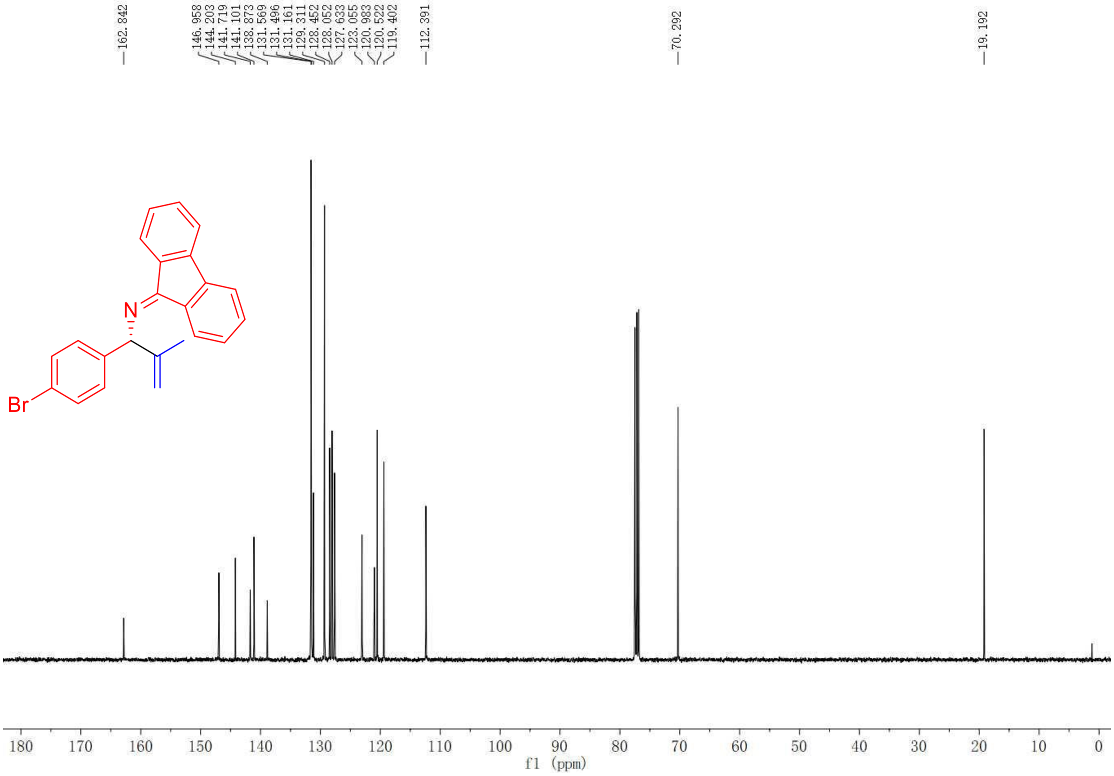

Figure S46. HPLC Chromatography of the Racemic *N*-(1-(4-Bromophenyl)-2- methylallyl)-9*H*- fluoren-9-imine (3ea).

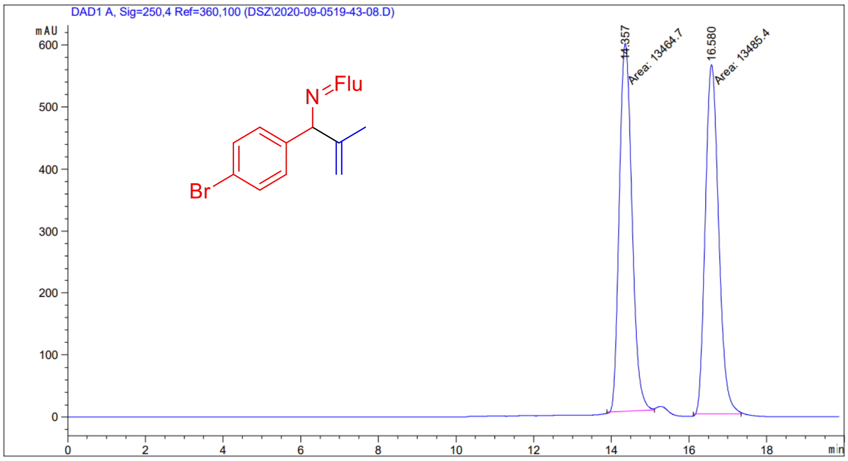

Signal 1: DAD1 A, Sig=250,4 Ref=360,100

Peak #	RetTime [min]	Type	Width [min]	Area [mAU*s]	Height [mAU]	Area %
1	14.357	MM	0.3784	1.34647e4	593.06653	49.9617
2	16.580	MM	0.3990	1.34854e4	563.26624	50.0383
Totals :				2.69501e4	1156.33276	

Figure S47. HPLC Chromatography of (*R*)-*N*-(1-(4-Bromophenyl)-2-methylallyl)-9*H*-fluoren-9-imine (3ea).

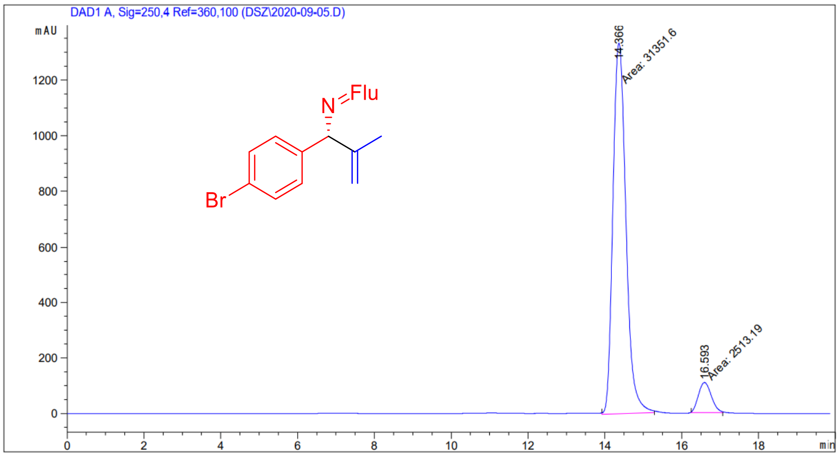

Signal 1: DAD1 A, Sig=250,4 Ref=360,100

Peak #	RetTime [min]	Type	Width [min]	Area [mAU*s]	Height [mAU]	Area %
1	14.366	MM	0.3918	3.13516e4	1333.76245	92.5788
2	16.593	MM	0.3882	2513.19092	107.89948	7.4212
Totals :				3.38648e4	1441.66193	

Figure S48. **^{1}H NMR spectra (400 MHz, Chloroform-*d*) of (*R*)-*N*-(1-(4-(*tert*-Butyl)phenyl)-2-methylallyl)-9*H*-fluoren-9-imine (3fa).**

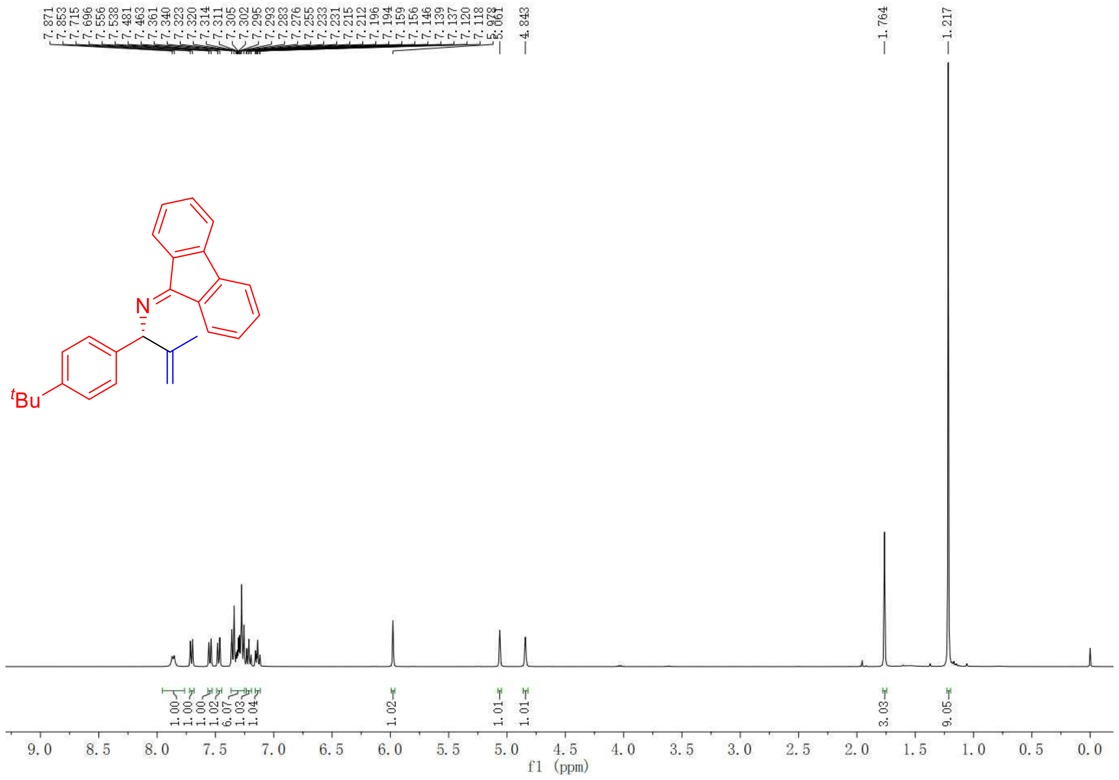

Figure S49. **^{13}C{^{1}H} NMR spectra (100 MHz, Chloroform-*d*) of (*R*)-*N*-(1-(4-(*tert*-Butyl)phenyl)-2-methylallyl)-9*H*-fluoren-9-imine (3fa).**

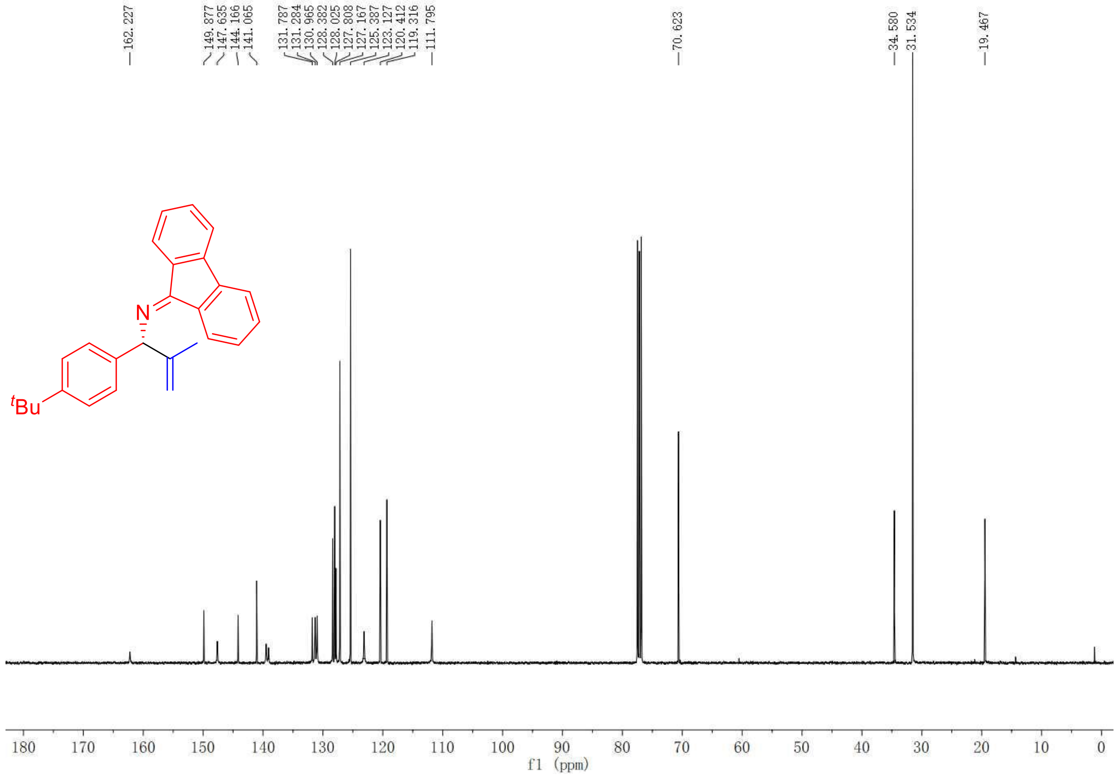

Figure S50. HPLC Chromatography of the Racemic *N*-(1-(4-(*tert*-Butyl)phenyl)-2-methylallyl)- 9*H*-fluoren-9-imine (3fa).

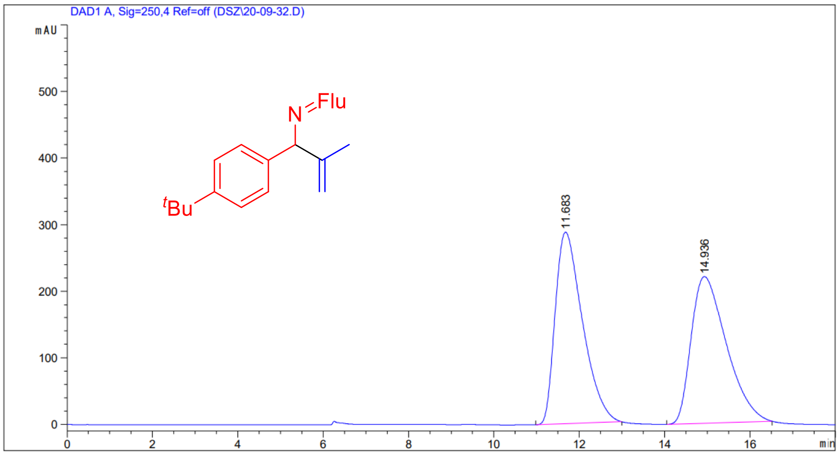

Signal 1: DAD1 A, Sig=250,4 Ref=off

Peak #	RetTime [min]	Type	Width [min]	Area [mAU*s]	Height [mAU]	Area %
1	11.683	BBA	0.6789	1.27881e4	287.50333	50.3565
2	14.936	BBA	0.8838	1.26070e4	220.23235	49.6435
Totals :				2.53951e4	507.73567	

Figure S51. HPLC Chromatography of (*R*)-*N*-(1-(4-(*tert*-Butyl)phenyl)-2-methylallyl)-9*H*-fluoren-9-imine (3fa).

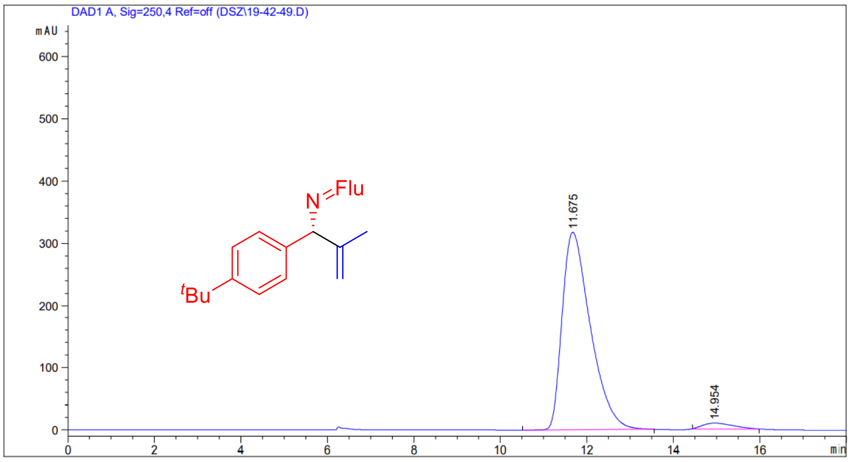

Signal 1: DAD1 A, Sig=250,4 Ref=off

Peak #	RetTime [min]	Type	Width [min]	Area [mAU*s]	Height [mAU]	Area %
1	11.675	BBA	0.6897	1.44282e4	317.80966	96.7296
2	14.954	HHA	0.7797	487.81732	9.68389	3.2704
Totals :				1.49160e4	327.49355	

Figure S52. **^{1}H NMR spectra (400 MHz, Chloroform-*d*) of (*R*)-*N*-(1-([1,1'-Biphenyl]-4-yl)-2-methylallyl)-9*H*-fluoren-9-imine (3ga).**

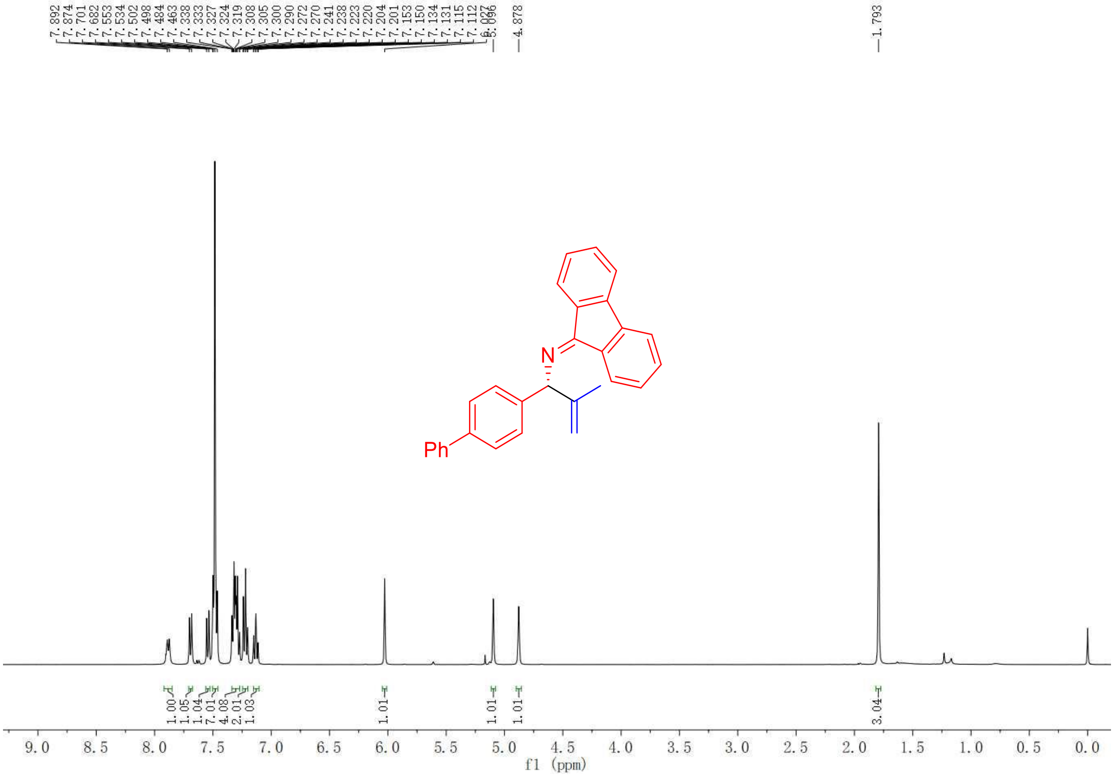

Figure S53. **^{13}C{^{1}H} NMR spectra (100 MHz, Chloroform-*d*) of (*R*)-*N*-(1-([1,1'-Biphenyl]-4-yl)-2-methylallyl)-9*H*-fluoren-9-imine (3ga).**

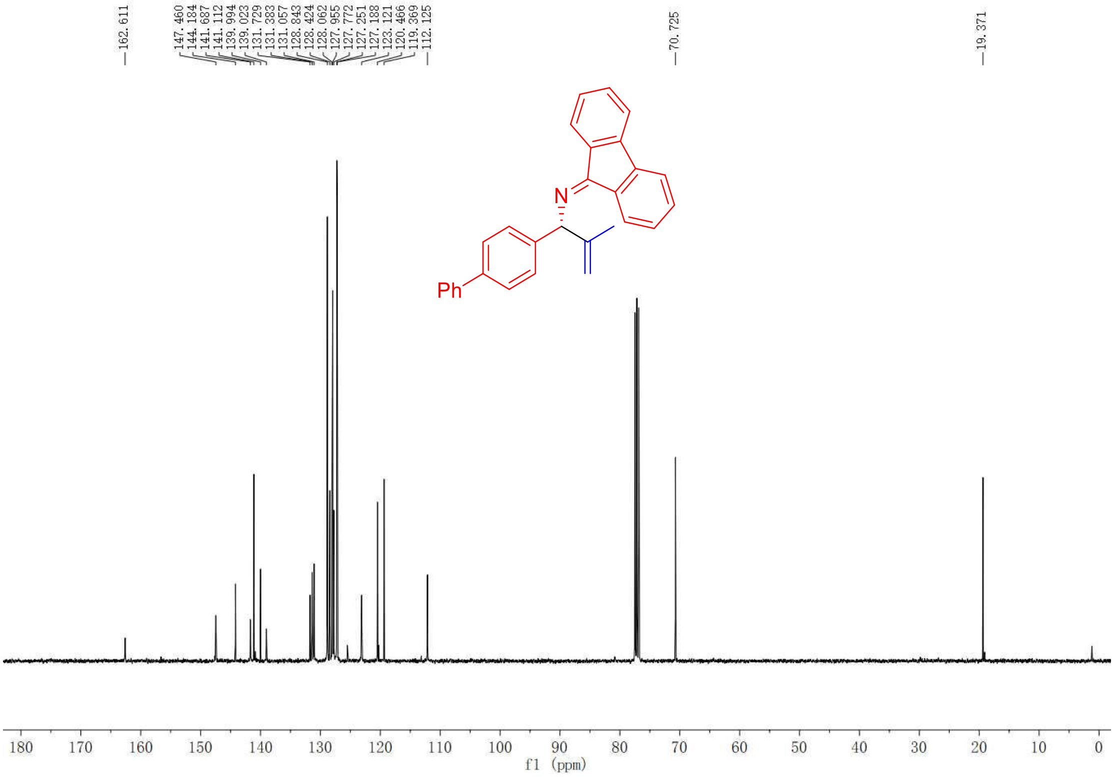

Figure S54. HPLC Chromatography of the Racemic (*N*-(1-([1,1'-Biphenyl]-4-yl)-2-methylallyl)-9*H*-fluoren-9-imine (3ga).

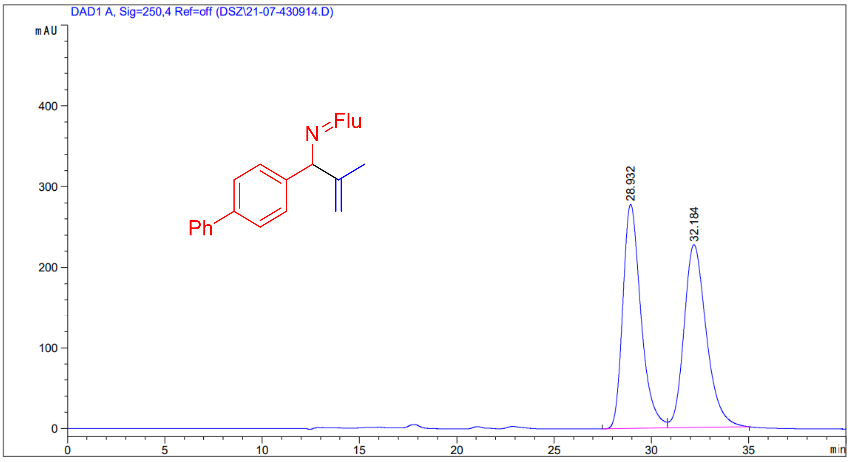

Signal 1: DAD1 A, Sig=250,4 Ref=off

Peak #	RetTime [min]	Type	Width [min]	Area [mAU*s]	Height [mAU]	Area %
1	28.932	BV	0.9883	1.80421e4	278.30872	49.8288
2	32.184	VBA	1.2206	1.81660e4	227.25209	50.1712

Totals : 3.62081e4 505.56081

Figure S55. HPLC Chromatography of (*R*)-*N*-(1-([1,1'-Biphenyl]-4-yl)-2-methylallyl)-9*H*-fluoren-9-imine (3ga).

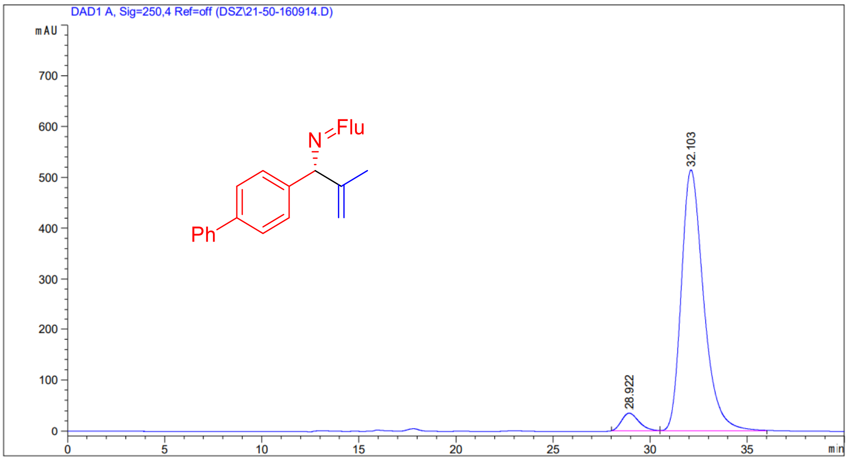

Signal 1: DAD1 A, Sig=250,4 Ref=off

Peak #	RetTime [min]	Type	Width [min]	Area [mAU*s]	Height [mAU]	Area %
1	28.922	HH S	0.9633	2194.71484	35.01414	5.0715
2	32.103	HHAS	1.2199	4.10805e4	514.30762	94.9285

Totals : 4.32752e4 549.32176

Figure S56. ^{1}H NMR spectra (400 MHz, Chloroform-*d*) of (*R*)-*N*-(2-Methyl-1-(3-(trifluoromethoxy)phenyl)allyl)-9*H*-fluoren-9-imine (3ha).

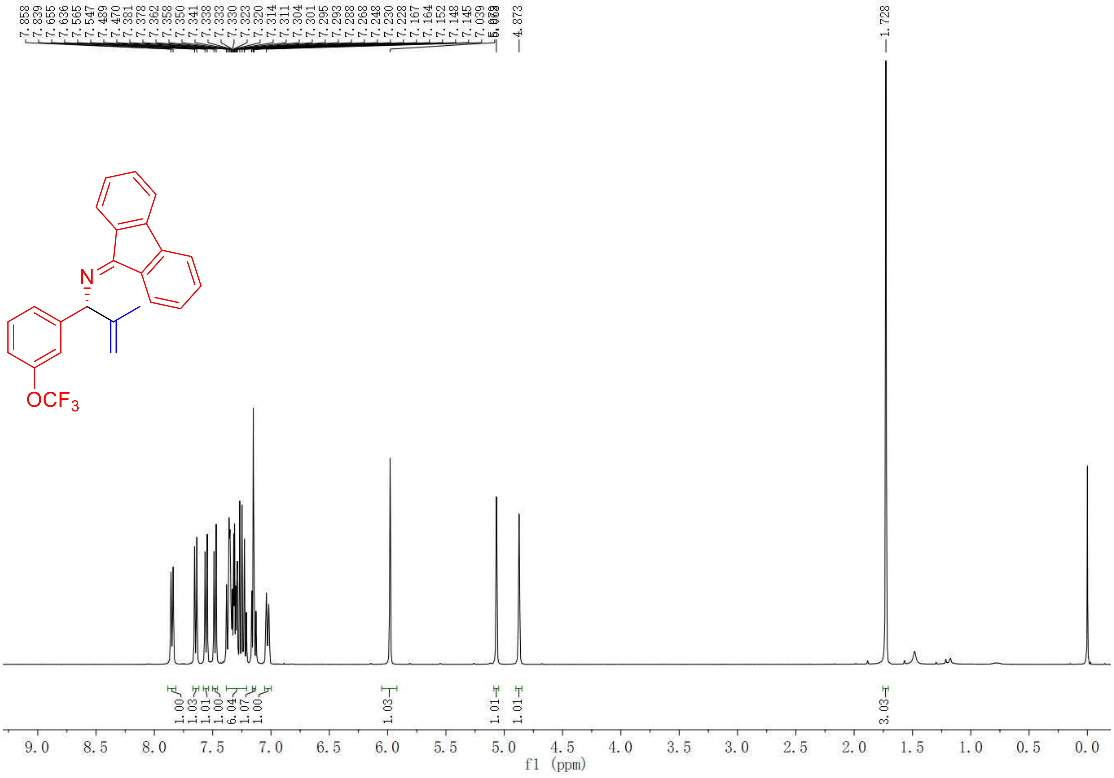

Figure S57. ^{13}C{^{1}H} NMR spectra (100 MHz, Chloroform-*d*) of (*R*)-*N*-(2-Methyl-1-(3-(trifluoromethoxy)phenyl)allyl)-9*H*-fluoren-9-imine (3ha).

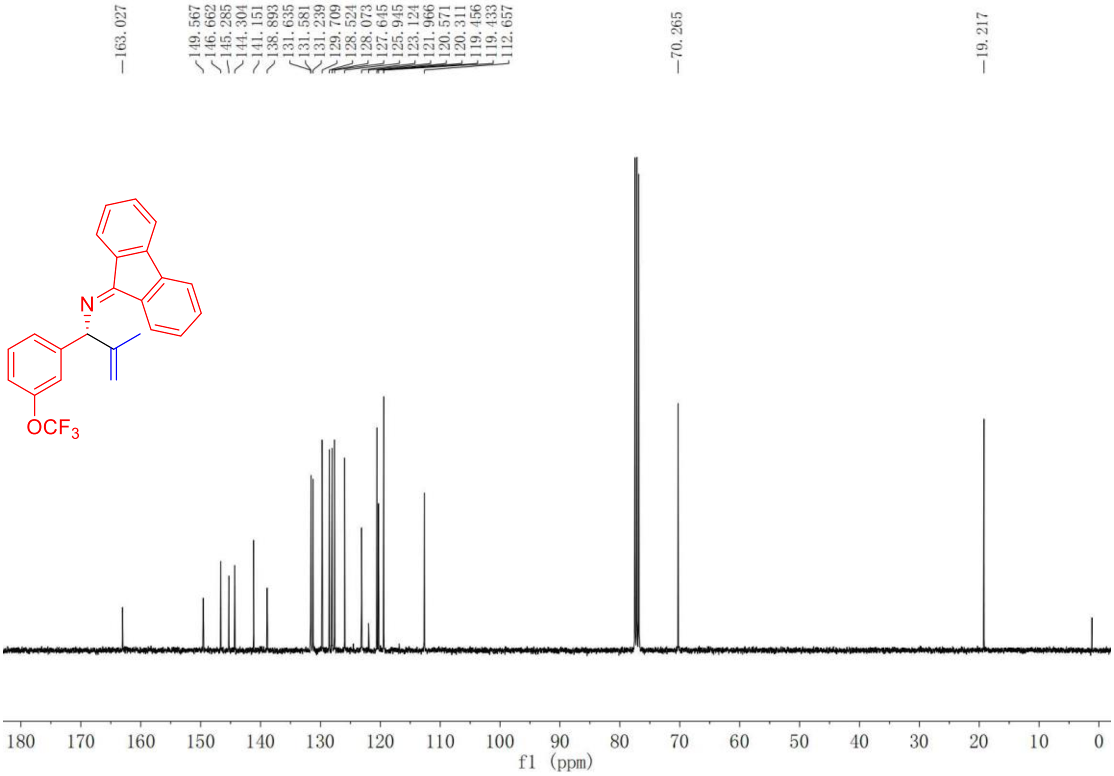

Figure S58. **^{19}F NMR spectra (376 MHz, Chloroform-*d*) of (*R*)-*N*-(2-Methyl-1-(3-(trifluoromethoxy)phenyl)allyl)-9*H*-fluoren-9-imine (3ha).**

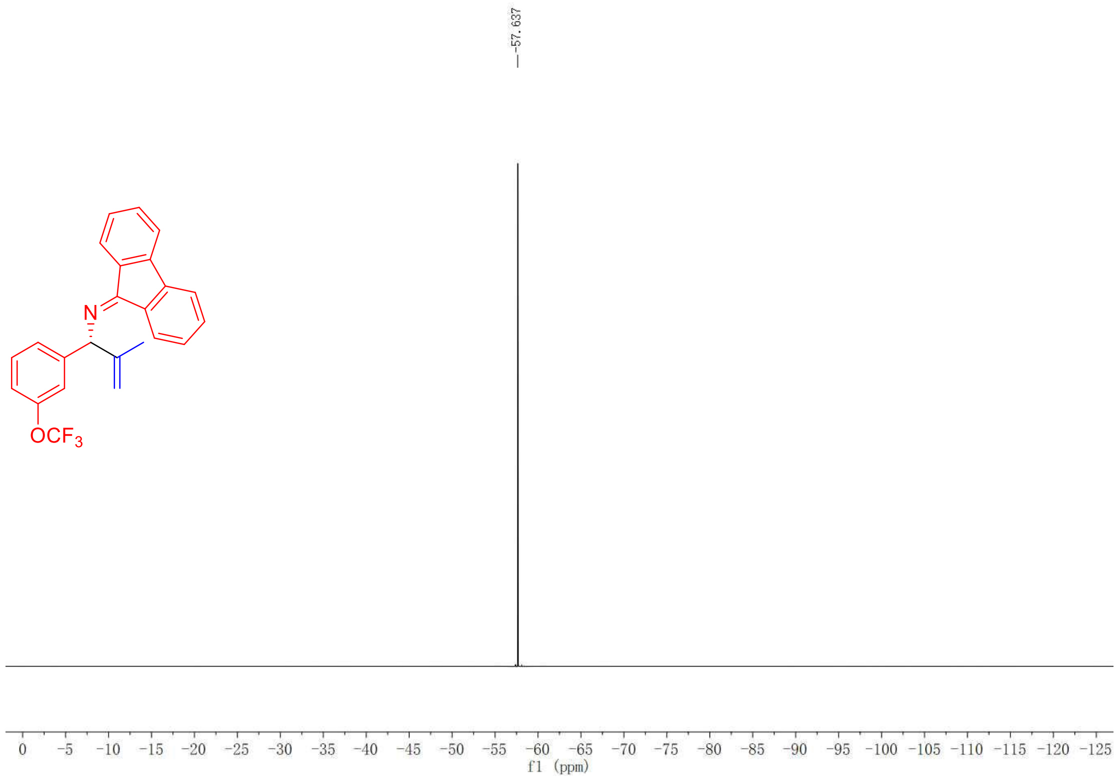

Figure S59. HPLC Chromatography of the Racemic *N*-(2-Methyl-1-(3-(trifluoromethoxy)phenyl)allyl)-9*H*-fluoren-9-imine (3ha).

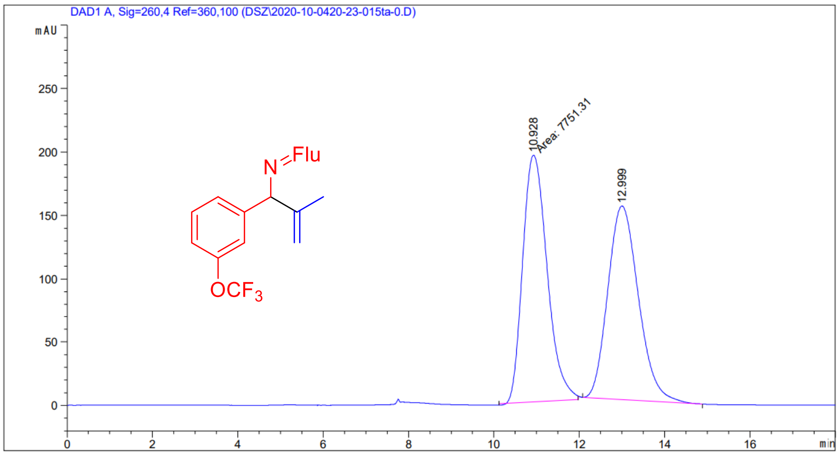

Signal 1: DAD1 A, Sig=260,4 Ref=360,100

Peak #	RetTime [min]	Type	Width [min]	Area [mAU*s]	Height [mAU]	Area %
1	10.928	MM	0.6633	7751.30957	194.76622	50.4327
2	12.999	BBA	0.7814	7618.31543	152.87025	49.5673

Totals : 1.53696e4 347.63647

Figure S60. HPLC Chromatography of (*R*)-*N*-(2-Methyl-1-(3-(trifluoromethoxy)phenyl)allyl)-9*H*-fluoren-9-imine (3ha).

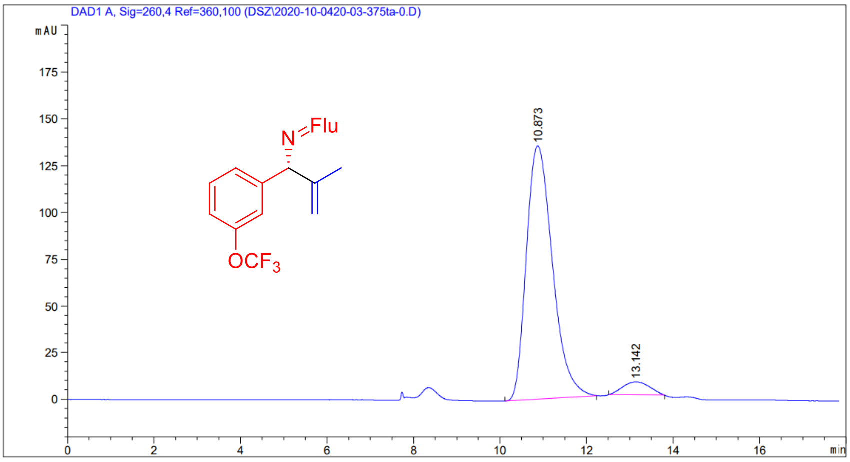

Signal 1: DAD1 A, Sig=260,4 Ref=360,100

Peak #	RetTime [min]	Type	Width [min]	Area [mAU*s]	Height [mAU]	Area %
1	10.873	BBA	0.6617	5686.99316	135.49939	94.9282
2	13.142	HHA	0.6903	303.84085	7.00077	5.0718

Totals : 5990.83401 142.50016

Figure S61. ^{1}**H NMR spectra (400 MHz, Chloroform-*d*) of (*R*)-*N*-(1-(3,4-Dimethoxyphenyl)- 2-methylallyl)-9*H*-fluoren-9-imine (3ia).**

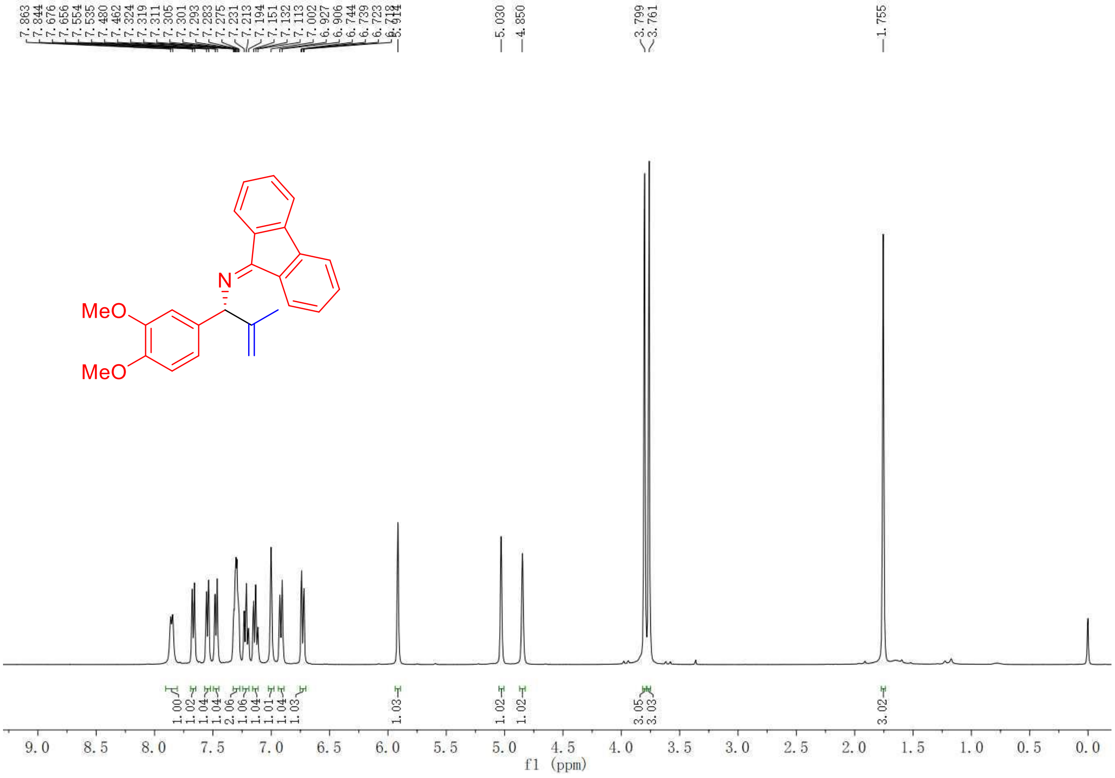

Figure S62. ^{13}**C{**^{1}**H} NMR spectra (100 MHz, Chloroform-*d*) of (*R*)-*N*-(1-(3,4- Dimethoxyphenyl)-2-methylallyl)-9*H*-fluoren-9-imine (3ia).**

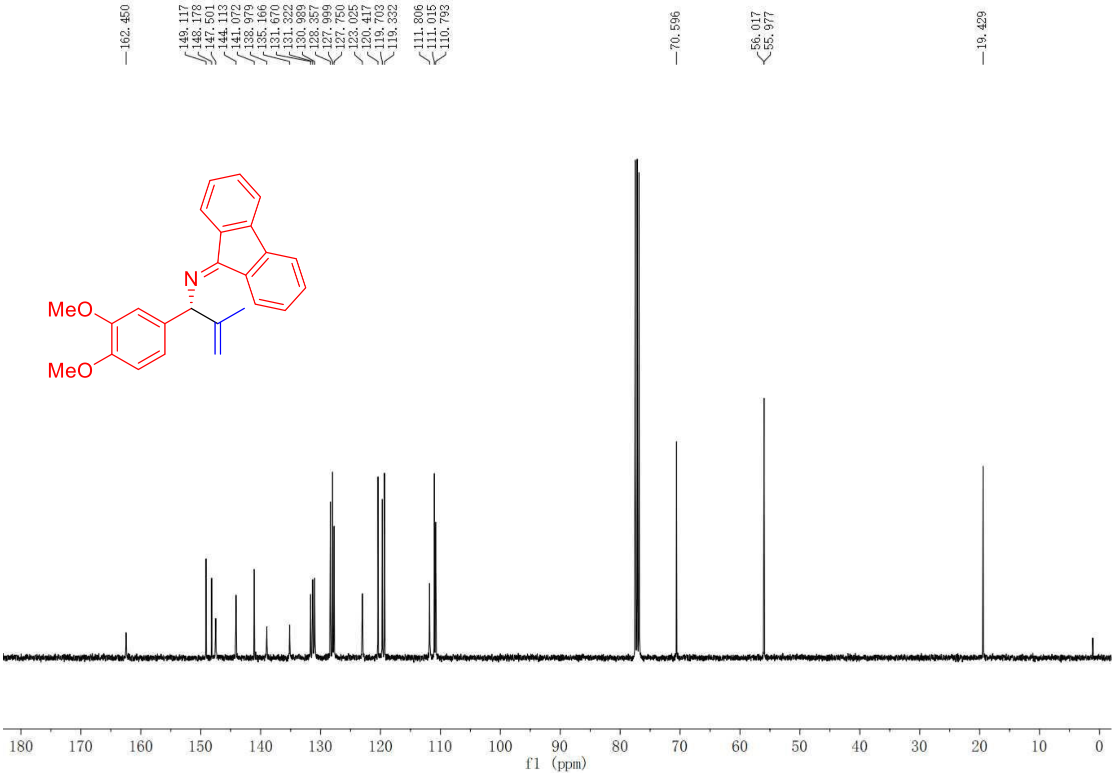

Figure S63. HPLC Chromatography of the Racemic *N*-(1-(3,4-Dimethoxyphenyl)-2-methylallyl)-9*H*-fluoren-9-imine (3ia).

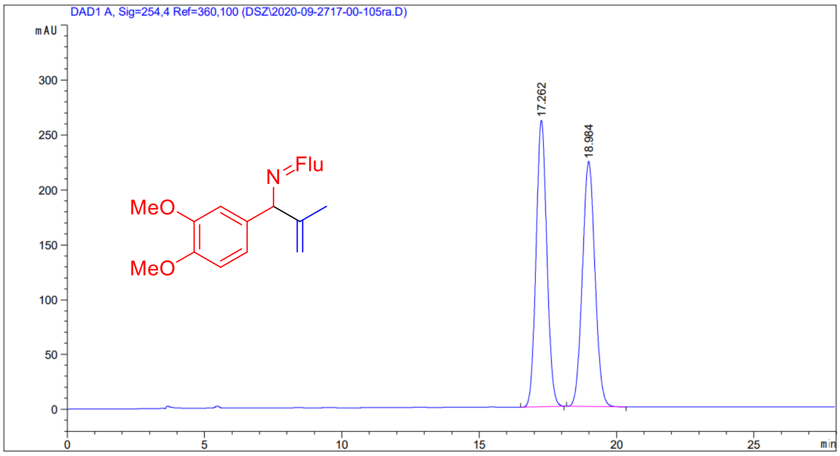

Signal 1: DAD1 A, Sig=254,4 Ref=360,100

Peak #	RetTime [min]	Type	Width [min]	Area [mAU*s]	Height [mAU]	Area %
1	17.262	BBA	0.4256	7105.77441	260.99222	49.9298
2	18.984	BBA	0.4923	7125.75049	223.27022	50.0702

Totals : 1.42315e4 484.26244

Figure S64 HPLC Chromatography of (*R*)-*N*-(1-(3,4-Dimethoxyphenyl)-2-methylallyl)- 9*H*-fluoren-9-imine (3ia).

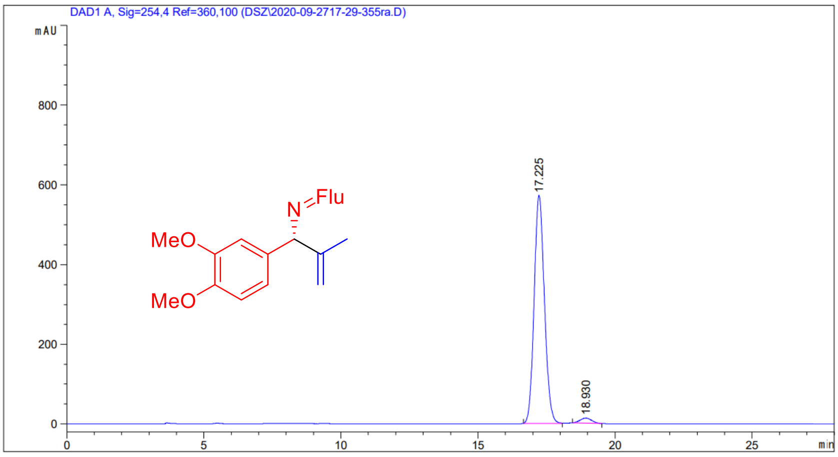

Signal 1: DAD1 A, Sig=254,4 Ref=360,100

Peak #	RetTime [min]	Type	Width [min]	Area [mAU*s]	Height [mAU]	Area %
1	17.225	HHA	0.4045	1.49467e4	572.70648	97.5722
2	18.930	HHA	0.4616	371.90195	12.70270	2.4278

Totals : 1.53186e4 585.40918

Figure S65. ^{1}**H NMR spectra (400 MHz, Chloroform-*d*) of (*R*)-*N*-(2-Methyl-1-(*o*-tolyl)allyl)-9*H*-fluoren-9-imine (3ja).**

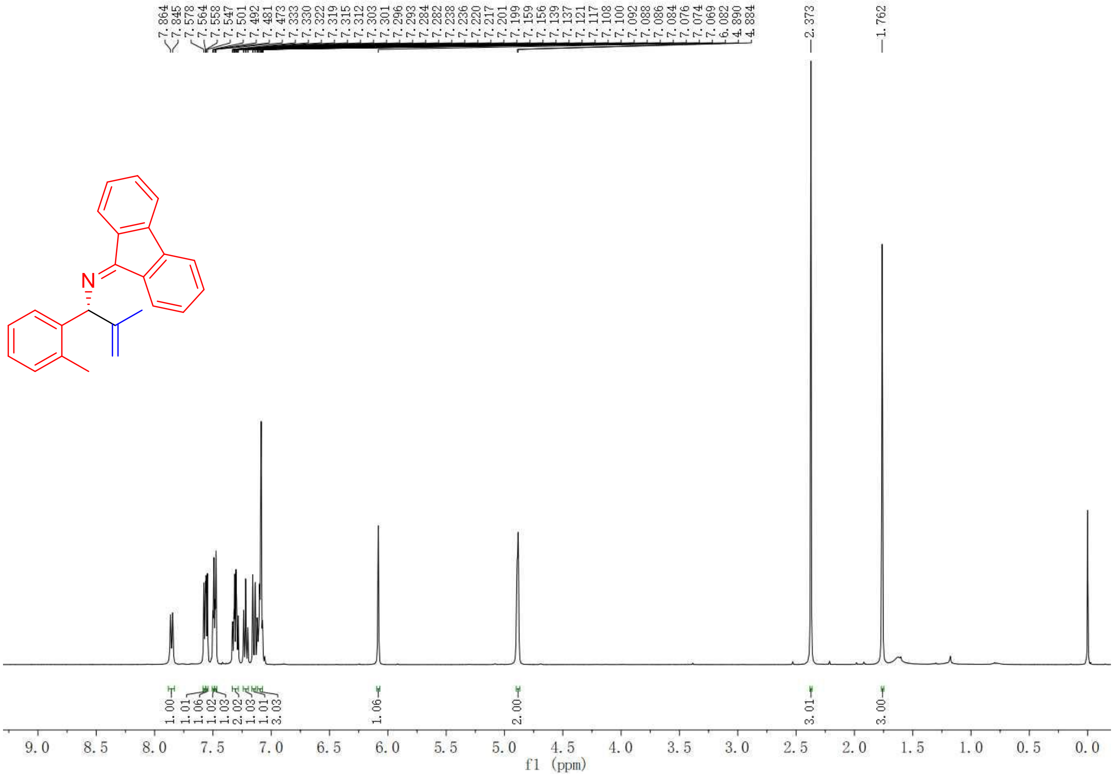

Figure S66. ^{13}**C{**^{1}**H} NMR spectra (100 MHz, Chloroform-*d*) of (*R*)-*N*-(2-Methyl-1-(*o*-tolyl) allyl)-9*H*-fluoren-9-imine (3ja).**

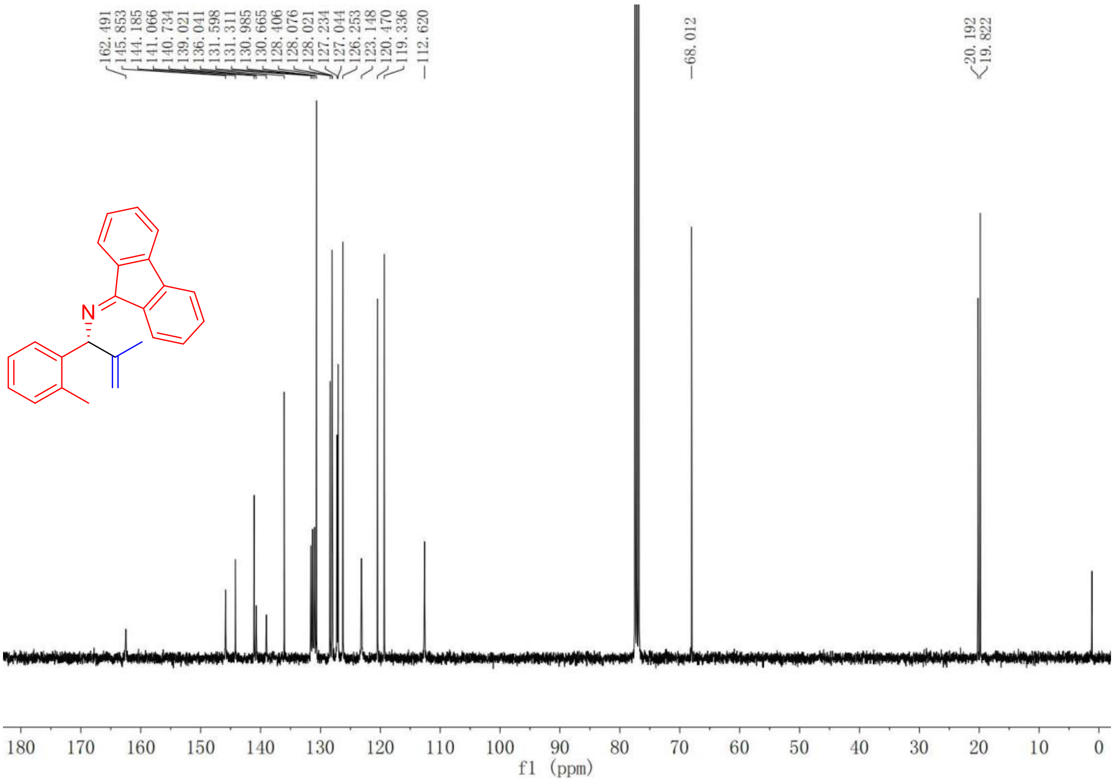

Figure S67. HPLC Chromatography of the Racemic *N*-(2-Methyl-1-(*o*-tolyl)allyl)-9*H*-fluoren-9-imine (3ja).

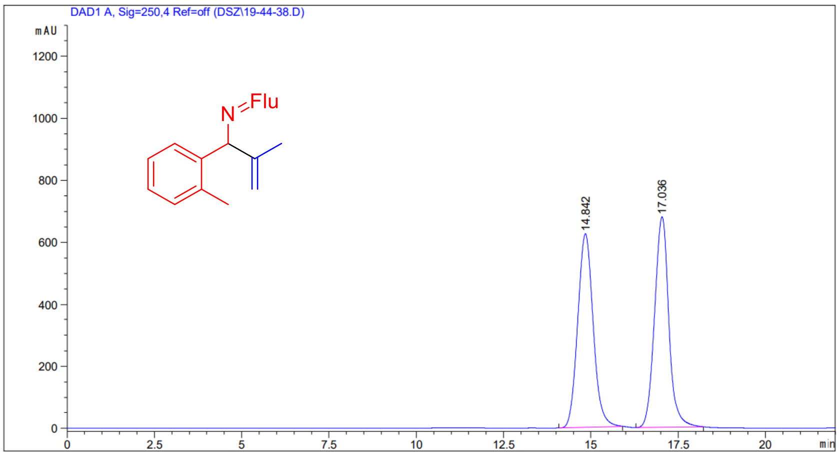

Signal 1: DAD1 A, Sig=250,4 Ref=off

Peak #	RetTime [min]	Type	Width [min]	Area [mAU*s]	Height [mAU]	Area %
1	14.842	BBA	0.4803	1.93124e4	625.35199	50.0527
2	17.036	BBA	0.4430	1.92717e4	679.32391	49.9473
Totals :				3.85841e4	1304.67590	

Figure S68. HPLC Chromatography of (*R*)-*N*-(2-Methyl-1-(*o*-tolyl)allyl)-9*H*-fluoren-9-imine (3ja).

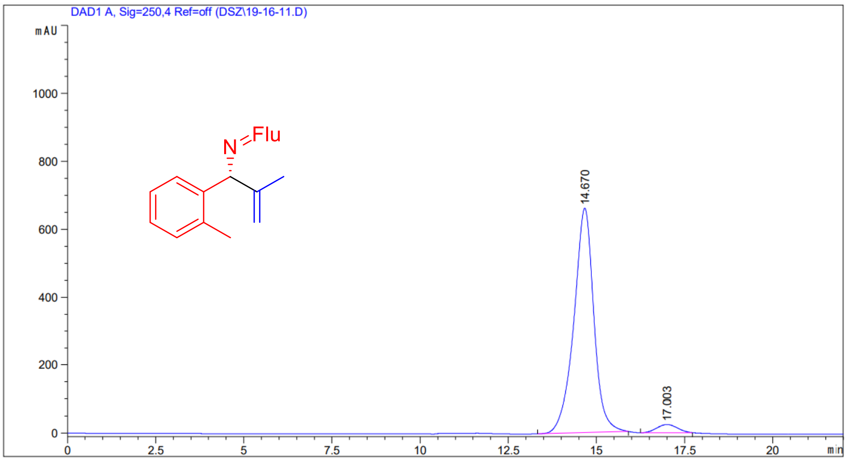

Signal 1: DAD1 A, Sig=250,4 Ref=off

Peak #	RetTime [min]	Type	Width [min]	Area [mAU*s]	Height [mAU]	Area %
1	14.670	BBA	0.5876	2.60296e4	660.78998	96.4106
2	17.003	BBA	0.6365	969.09601	24.13340	3.5894
Totals :				2.69987e4	684.92338	

Figure S69. ^{1}H **NMR spectra (400 MHz, Chloroform-*d*) of (*R*)-*N*-(2-Methyl-1-(naphthalen-1-yl)allyl)-9*H*-fluoren-9-imine (3ka).**

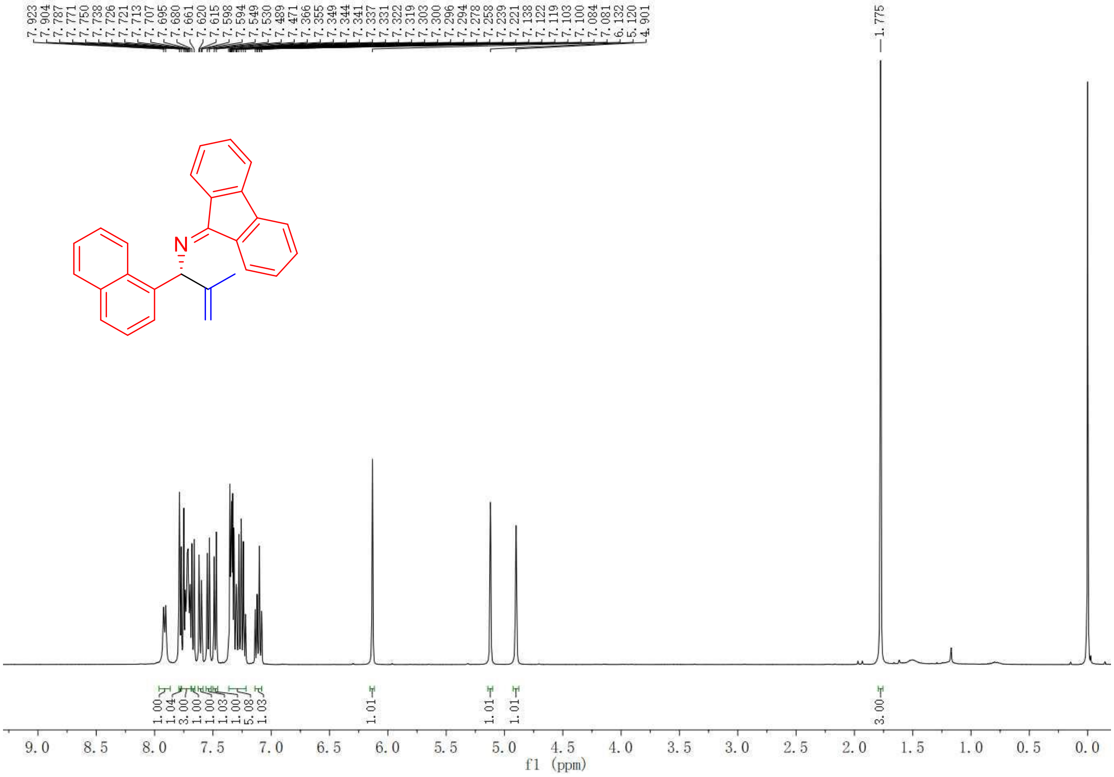

Figure S70. $^{13}C\{^{1}H\}$ **NMR spectra (100 MHz, Chloroform-*d*) of (*R*)-*N*-(2-Methyl-1-(naphthalen-1-yl)allyl)-9*H*-fluoren-9-imine (3ka).**

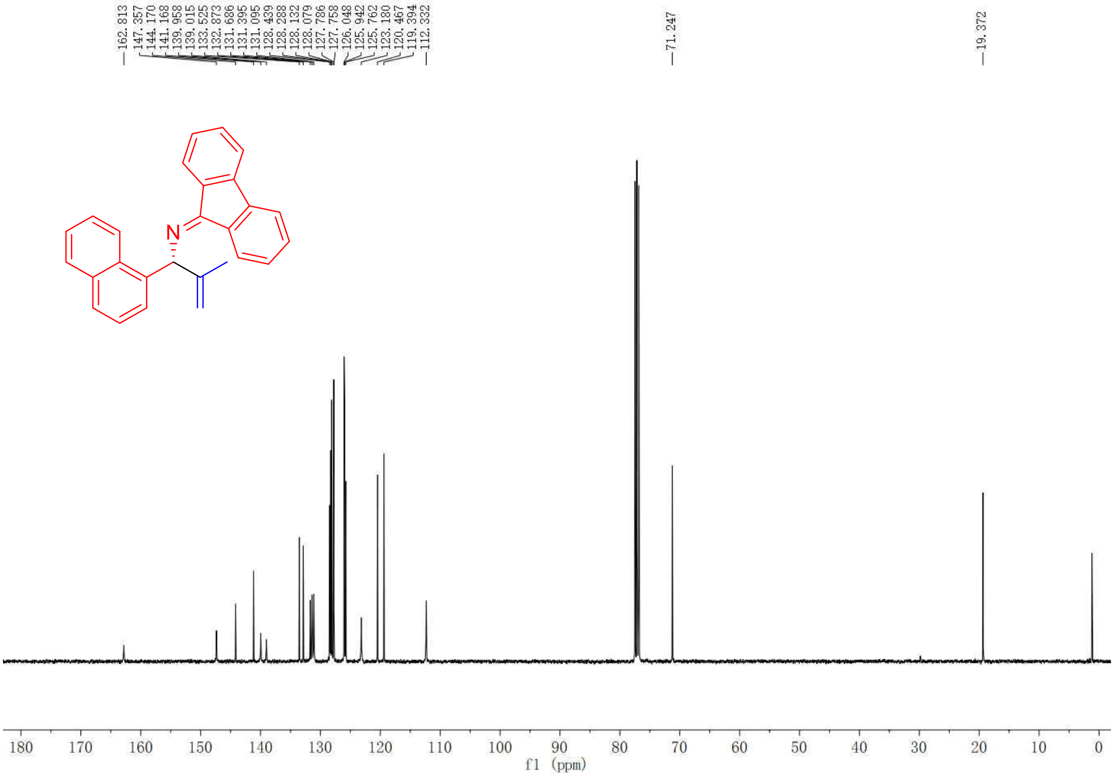

Figure S71. HPLC Chromatography of the Racemic *N*-(2-Methyl-1-(naphthalen-1-yl)allyl)-9*H*- fluoren-9-imine (3ka).

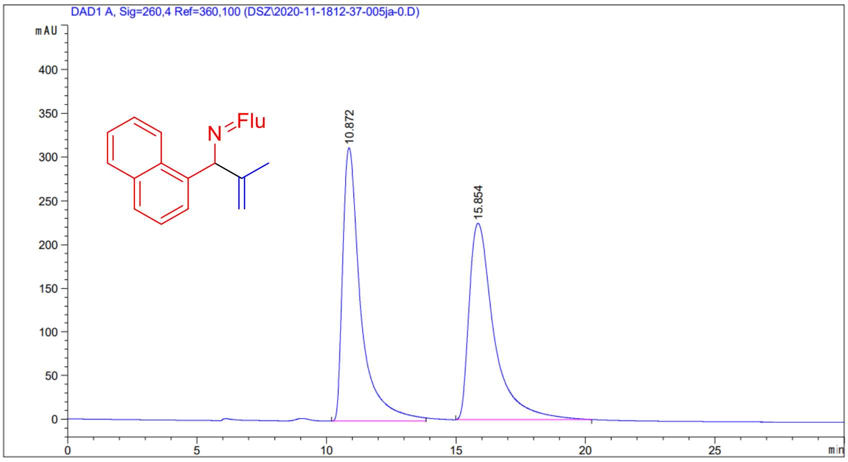

Signal 1: DAD1 A, Sig=260,4 Ref=360,100

Peak #	RetTime [min]	Type	Width [min]	Area [mAU*s]	Height [mAU]	Area %
1	10.872	HHA	0.7275	1.52150e4	312.79388	50.2907
2	15.854	HHA	1.0114	1.50391e4	225.11456	49.7093
Totals :				3.02542e4	537.90845	

Figure S72. HPLC Chromatography of (*R*)-*N*-(2-Methyl-1-(naphthalen-1-yl)allyl)-9*H*-fluoren-9-imine (3ka).

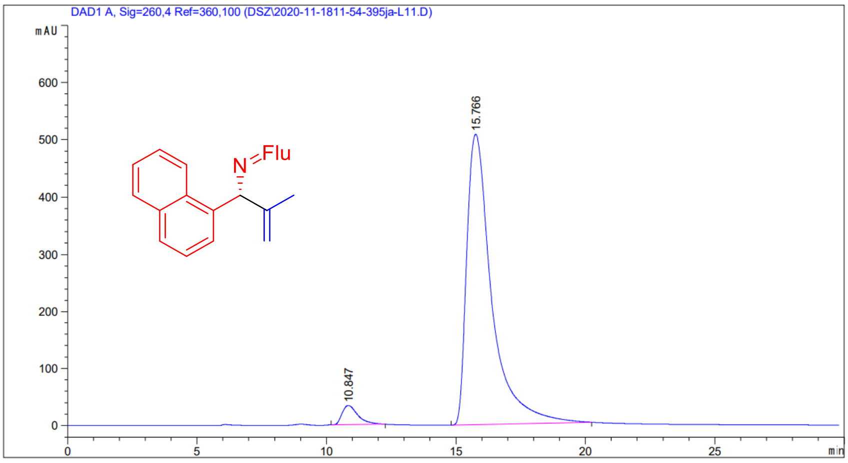

Signal 1: DAD1 A, Sig=260,4 Ref=360,100

Peak #	RetTime [min]	Type	Width [min]	Area [mAU*s]	Height [mAU]	Area %
1	10.847	HBA	0.6551	1420.48242	33.47132	4.0045
2	15.766	BBA	1.0145	3.40521e4	507.67627	95.9955
Totals :				3.54726e4	541.14759	

Figure S73. ^{1}H NMR spectra (400 MHz, Chloroform-*d*) of (*R*)-*N*-(1-(2,3-Dihydrobenzofuran-5-yl)-2-methylallyl)-9*H*-fluoren-9-imine (3la).

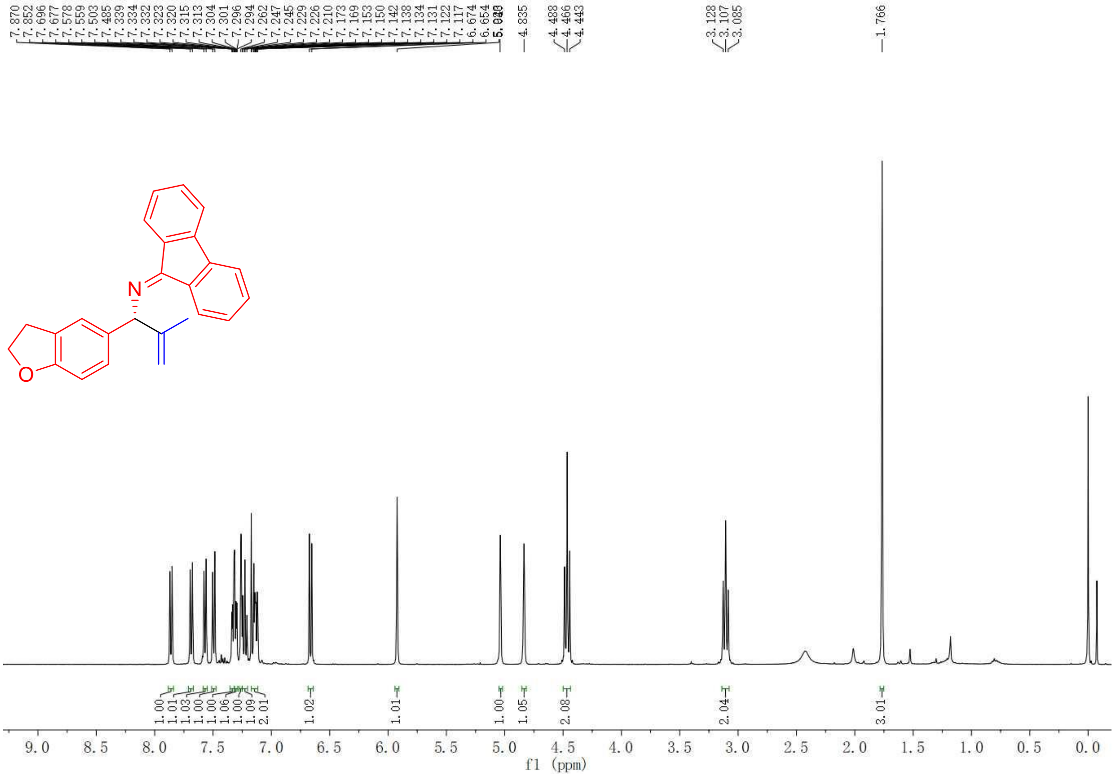

Figure S74. ^{13}C{^{1}H} NMR spectra (100 MHz, Chloroform-*d*) of (*R*)-*N*-(1-(2,3-Dihydrobenzofuran-5-yl)-2-methylallyl)-9*H*-fluoren-9-imine (3la).

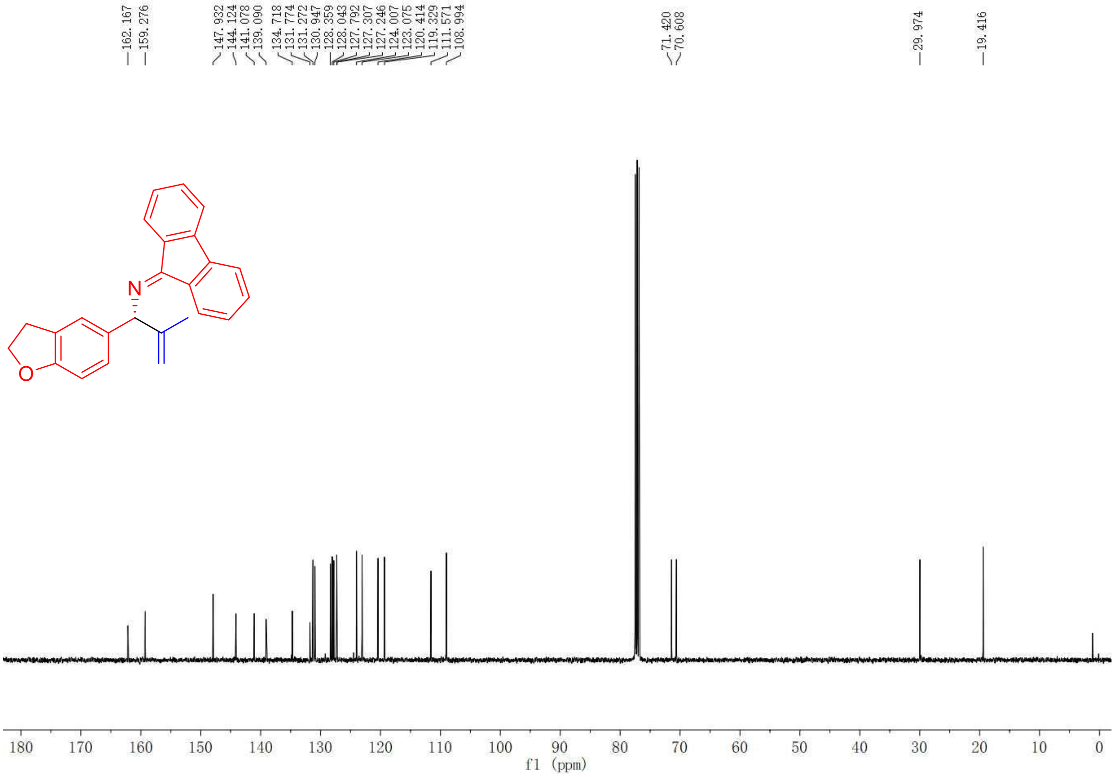

Figure S75. HPLC Chromatography of the Racemic *N*-(1-(2,3-Dihydrobenzofuran-5-yl)-2-methylallyl)-9*H*-fluoren-9-imine (3la).

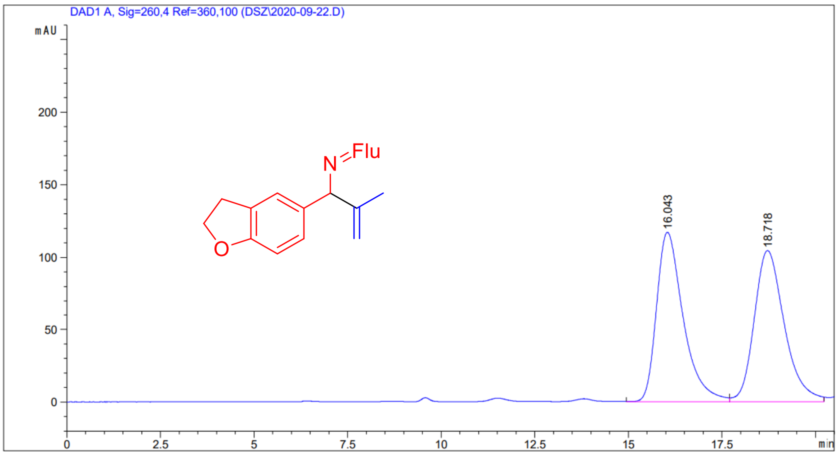

Signal 1: DAD1 A, Sig=260,4 Ref=360,100

Peak #	RetTime [min]	Type	Width [min]	Area [mAU*s]	Height [mAU]	Area %
1	16.043	HH	0.7548	5807.16650	117.05287	49.9021
2	18.718	HHA	0.8592	5829.95605	104.44483	50.0979

Totals : 1.16371e4 221.49770

Figure S76. HPLC Chromatography of (*R*)-*N*-(1-(2,3-Dihydrobenzofuran-5-yl)-2-methylallyl)-9*H*-fluoren-9-imine (3la).

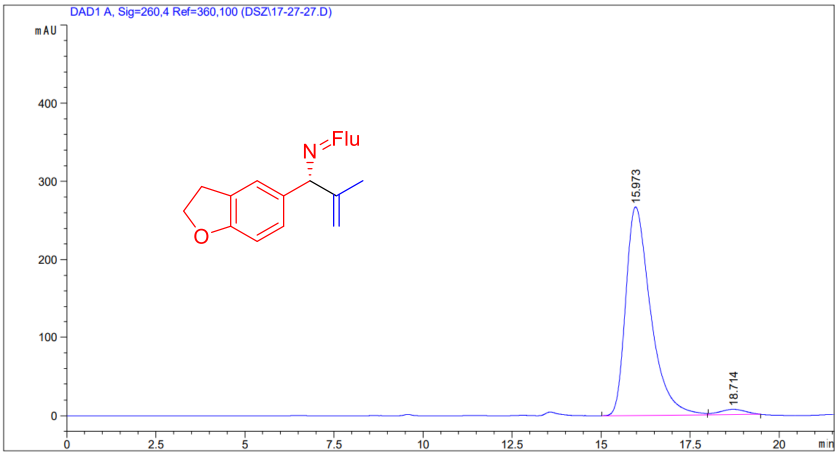

Signal 1: DAD1 A, Sig=260,4 Ref=360,100

Peak #	RetTime [min]	Type	Width [min]	Area [mAU*s]	Height [mAU]	Area %
1	15.973	BF	0.7344	1.29496e4	266.73911	97.5145
2	18.714	HBA	0.7565	330.07373	6.63313	2.4855

Totals : 1.32797e4 273.37224

Figure S77. ^{1}H NMR spectra (400 MHz, Chloroform-*d*) of (*R*)-*N*-(1-(2,6-Dimethoxypyridin- 3-yl)-2-methylallyl)-9*H*-fluoren-9-imine (3ma).

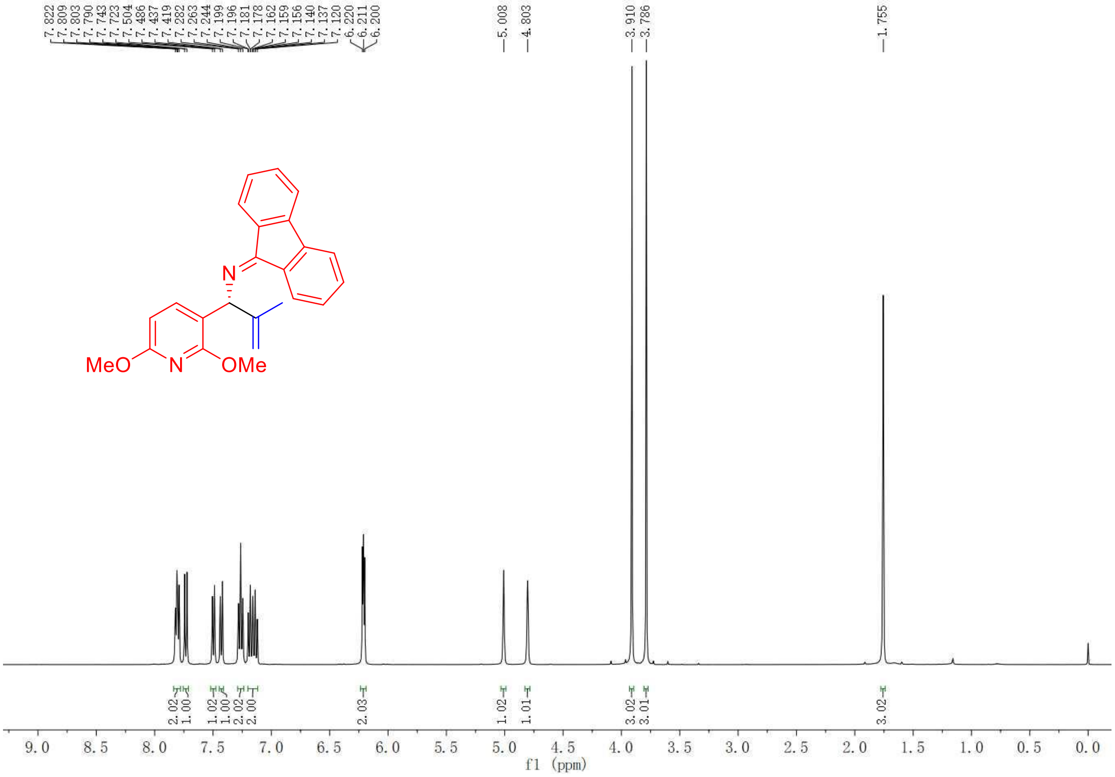

Figure S78. ^{13}C{^{1}H} NMR spectra (100 MHz, Chloroform-*d*) of (*R*)-*N*-(1-(2,6-Dimethoxypyridin-3-yl)-2-methylallyl)-9*H*-fluoren-9-imine (3ma).

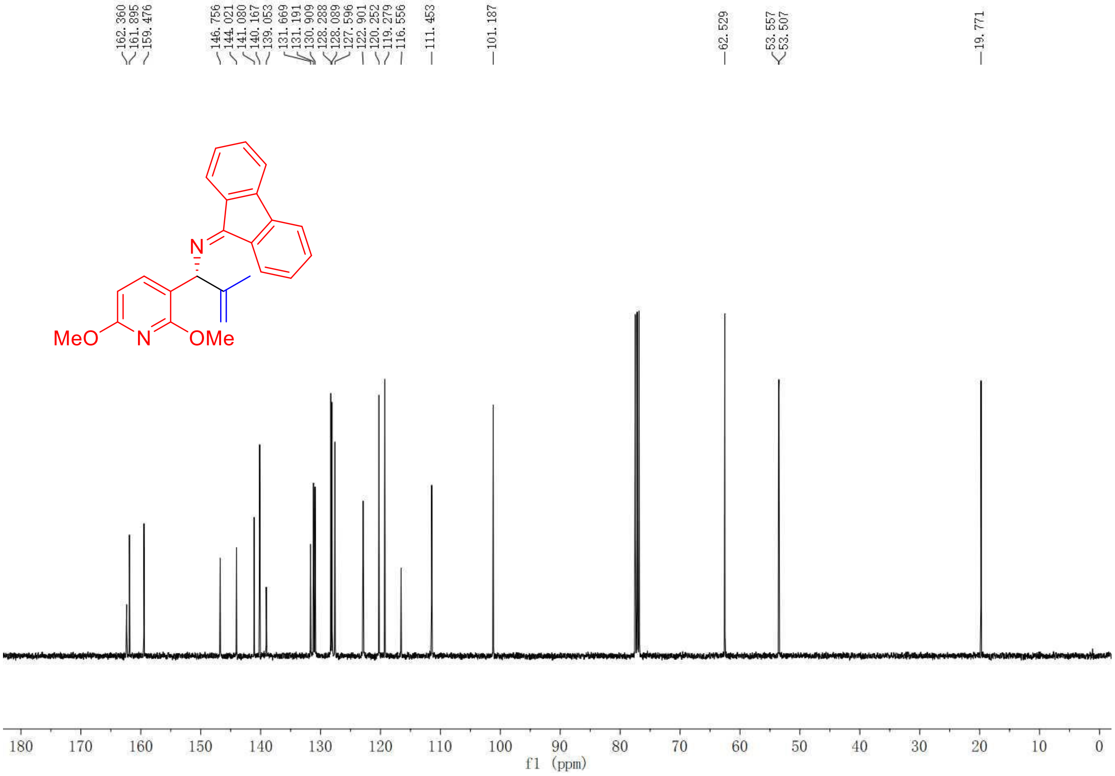

Figure S79. HPLC Chromatography of the Racemic *N*-(1-(2,6-Dimethoxypyridin-3-yl)-2-methylallyl)-9*H*-fluoren-9-imine (3ma).

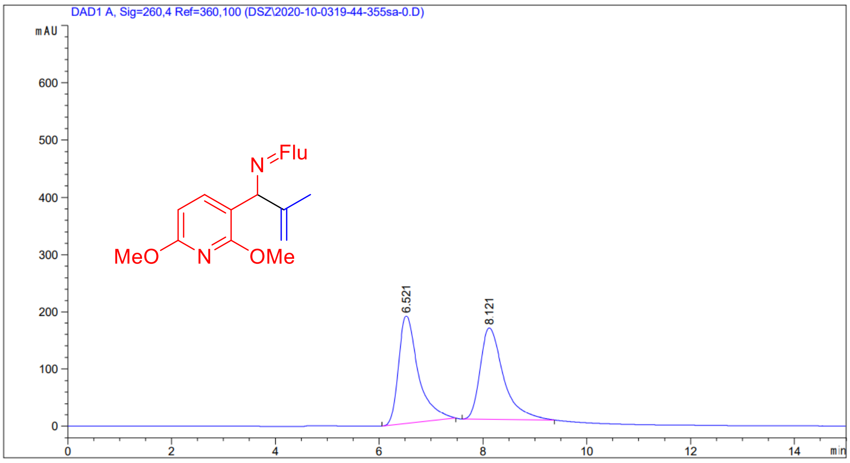

Signal 1: DAD1 A, Sig=260,4 Ref=360,100

Peak #	RetTime [min]	Type	Width [min]	Area [mAU*s]	Height [mAU]	Area %
1	6.521	BBA	0.3978	4994.41992	188.10025	49.9315
2	8.121	BBA	0.4749	5008.11377	159.23732	50.0685

Totals : 1.00025e4 347.33757

Figure S80. HPLC Chromatography of (*R*)-*N*-(1-(2,6-Dimethoxypyridin-3-yl)-2- methylallyl)-9*H*-fluoren-9-imine (3ma).

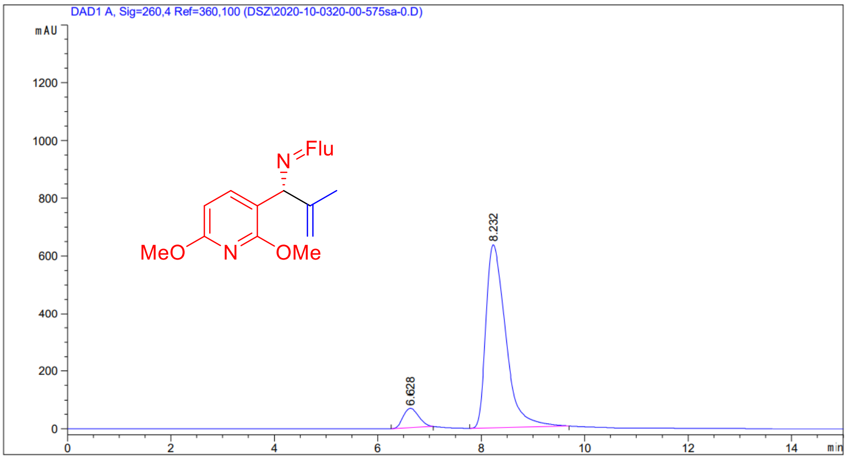

Signal 1: DAD1 A, Sig=260,4 Ref=360,100

Peak #	RetTime [min]	Type	Width [min]	Area [mAU*s]	Height [mAU]	Area %
1	6.628	BBA	0.3326	1401.51318	66.84074	7.6560
2	8.232	BBA	0.4225	1.69045e4	635.23511	92.3440

Totals : 1.83060e4 702.07584

Figure S81 ^{1}H NMR spectra (400 MHz, Chloroform-*d*) of (*R*)-*N*-(1-(Furan-3-yl)-2-methylallyl)-9*H*-fluoren-9-imine (3na).

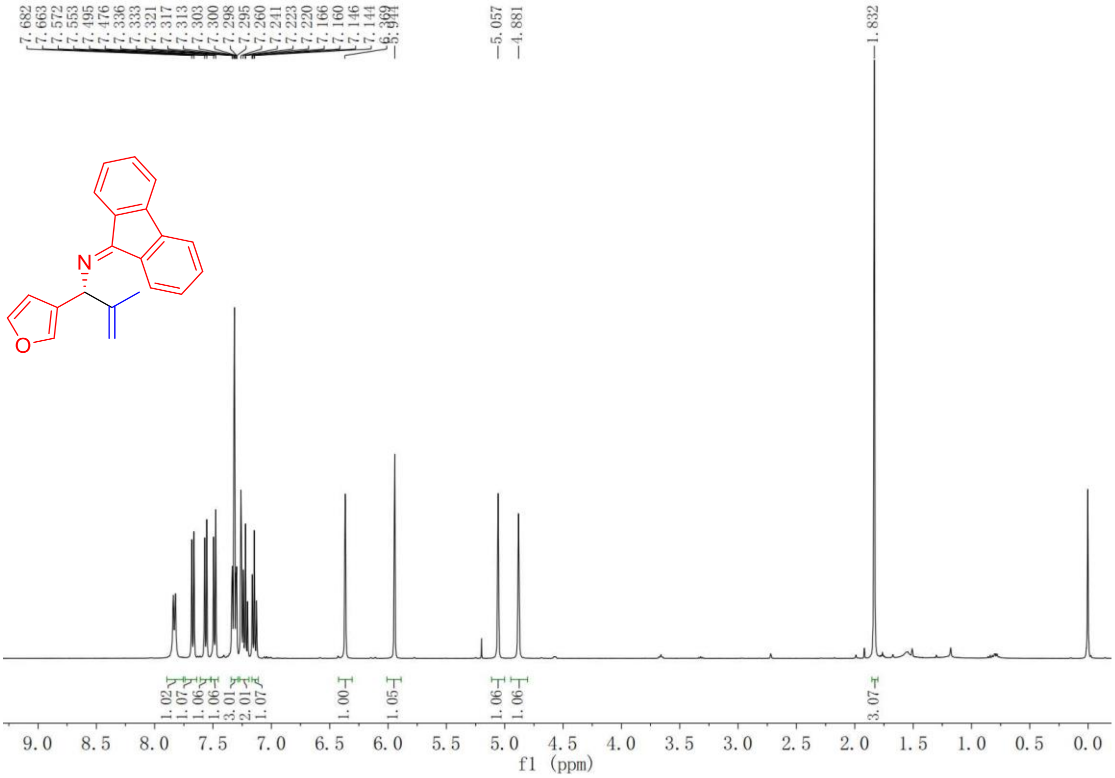

Figure S82. $^{13}C\{^{1}H\}$ NMR spectra (400 MHz, Chloroform-*d*) of (*R*)-*N*-(1-(Furan-3-yl)-2-methylallyl)-9*H*-fluoren-9-imine (3na).

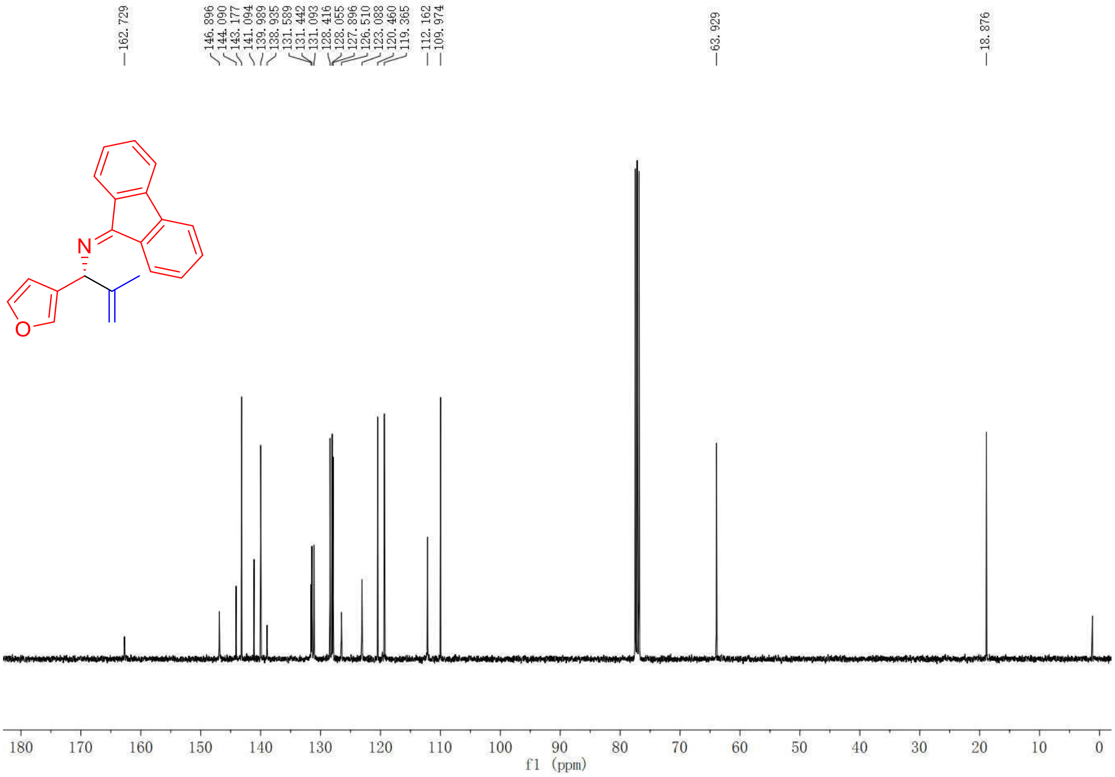

Figure S83. HPLC Chromatography of the Racemic *N*-(1-(Furan-3-yl)-2-methylallyl)-9*H*-fluoren-9-imine (3na).

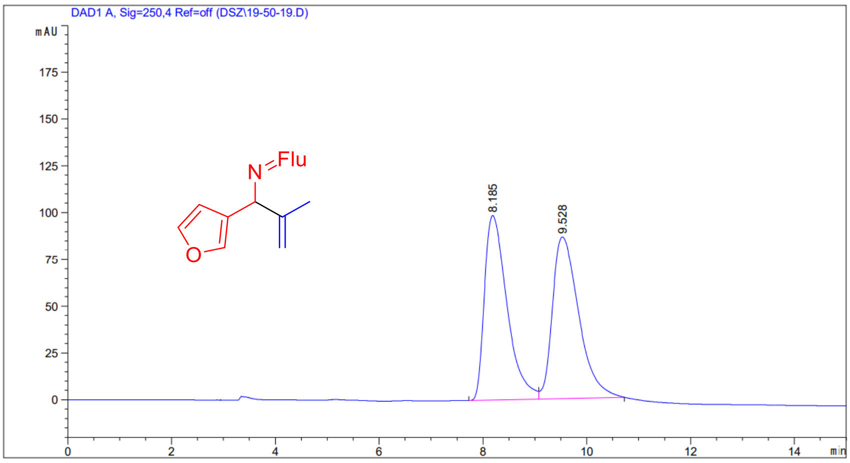

Signal 1: DAD1 A, Sig=250,4 Ref=off

Peak #	RetTime [min]	Type	Width [min]	Area [mAU*s]	Height [mAU]	Area %
1	8.185	BV	0.4718	2989.34326	98.56075	49.5632
2	9.528	VBA	0.5482	3042.03516	86.51473	50.4368

Totals : 6031.37842 185.07549

Figure S84. HPLC Chromatography of (*R*)-*N*-(1-(Furan-3-yl)-2-methylallyl)-9*H*-fluoren-9-imine (3na).

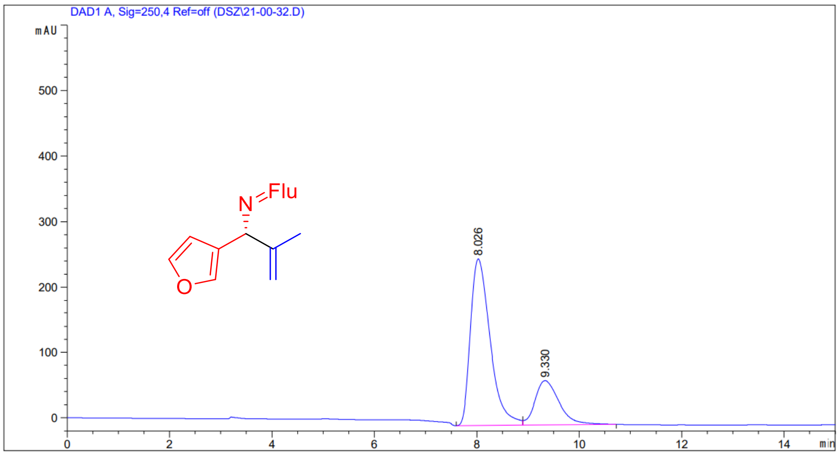

Signal 1: DAD1 A, Sig=250,4 Ref=off

Peak #	RetTime [min]	Type	Width [min]	Area [mAU*s]	Height [mAU]	Area %
1	8.026	BV	0.4163	6839.08350	255.49373	75.5315
2	9.330	VBA	0.5039	2215.52588	67.67581	24.4685

Totals : 9054.60938 323.16954

Figure S85. **^{1}H NMR spectra (400 MHz, Chloroform-*d*) of (*R*)-*N*-(1-Phenylallyl)-9*H*-fluoren-9-imine (3ab).**

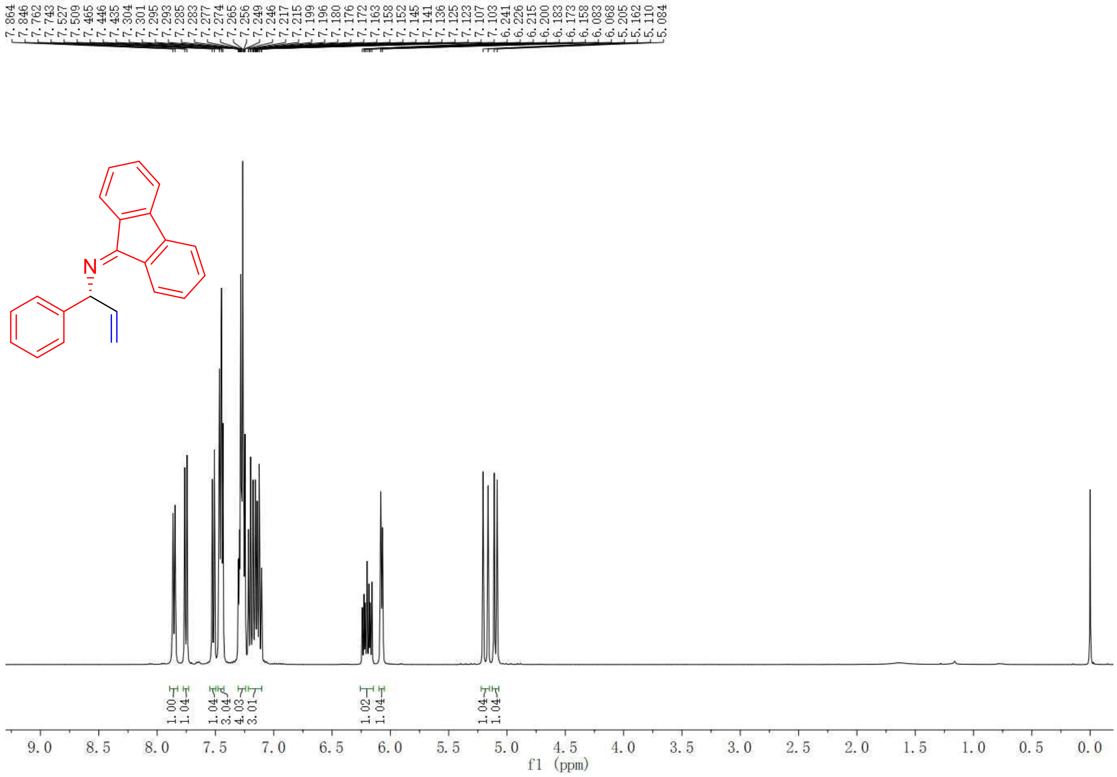

Figure S86. **^{13}C{^{1}H} NMR spectra (100 MHz, Chloroform-*d*) of (*R*)-*N*-(1-Phenylallyl)-9*H*-fluoren-9-imine (3ab).**

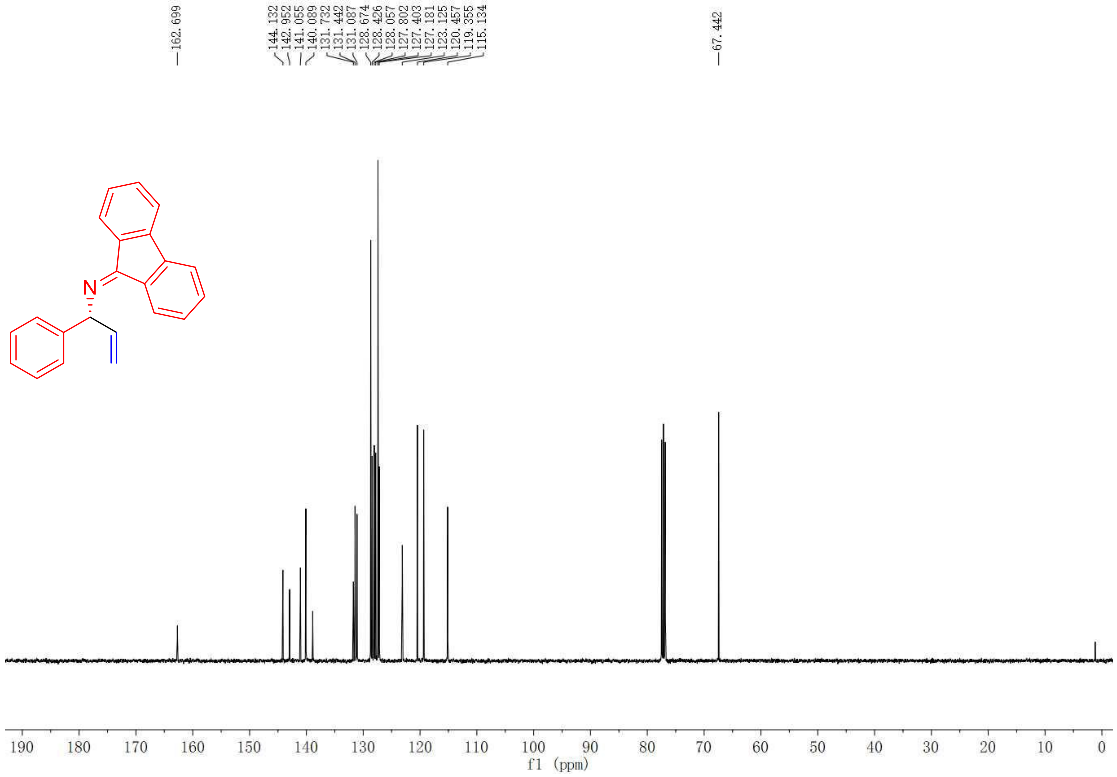

Figure S87. HPLC Chromatography of the Racemic *N*-(1-Phenylallyl)-9*H*-fluoren-9-imine (3ab).

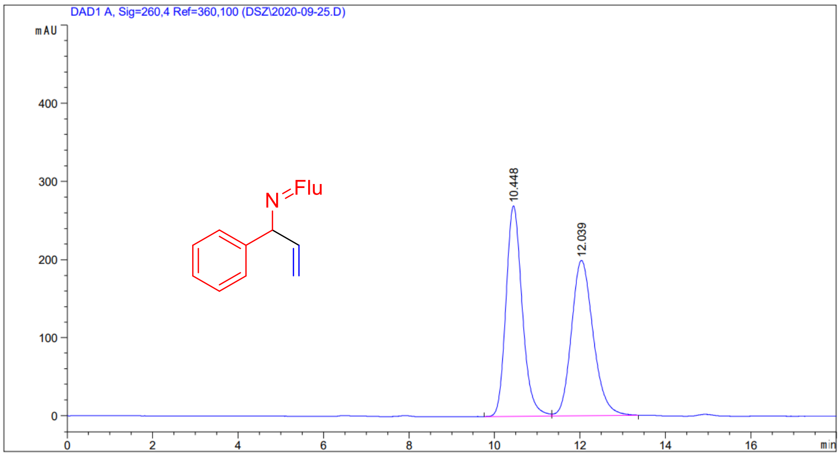

Signal 1: DAD1 A, Sig=260,4 Ref=360,100

Peak #	RetTime [min]	Type	Width [min]	Area [mAU*s]	Height [mAU]	Area %
1	10.448	BV	0.4081	7119.98877	269.60492	50.3271
2	12.039	VBA	0.5472	7027.43799	199.37094	49.6729

Totals : 1.41474e4 468.97586

Figure S88. HPLC Chromatography of (*R*)-*N*-(1-Phenylallyl)-9*H*-fluoren-9-imine (3ab).

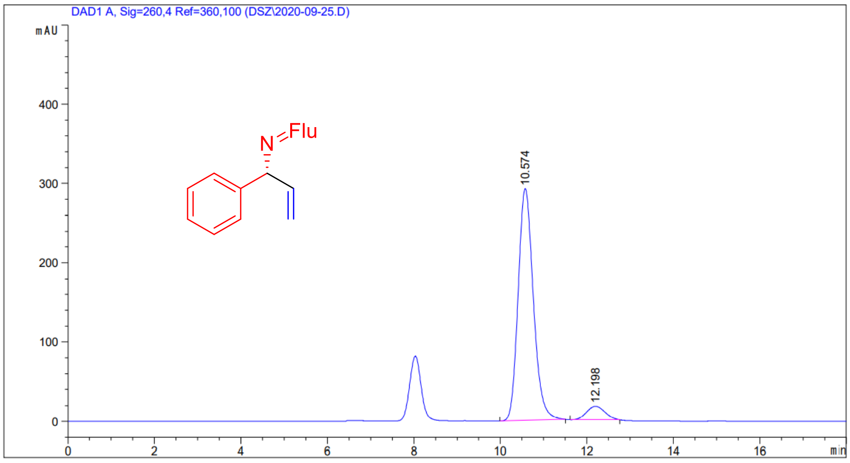

Signal 1: DAD1 A, Sig=260,4 Ref=360,100

Peak #	RetTime [min]	Type	Width [min]	Area [mAU*s]	Height [mAU]	Area %
1	10.574	BBA	0.3790	7144.65186	292.43661	93.3017
2	12.198	BHAS	0.4677	512.92401	17.40985	6.6983

Totals : 7657.57587 309.84647

Figure S89. ^{1}H NMR spectra (400 MHz, Chloroform-*d*) of (*R*)-*N*-(2-Methylene-1-phenylbutyl)-9*H*-fluoren-9-imine (3ac).

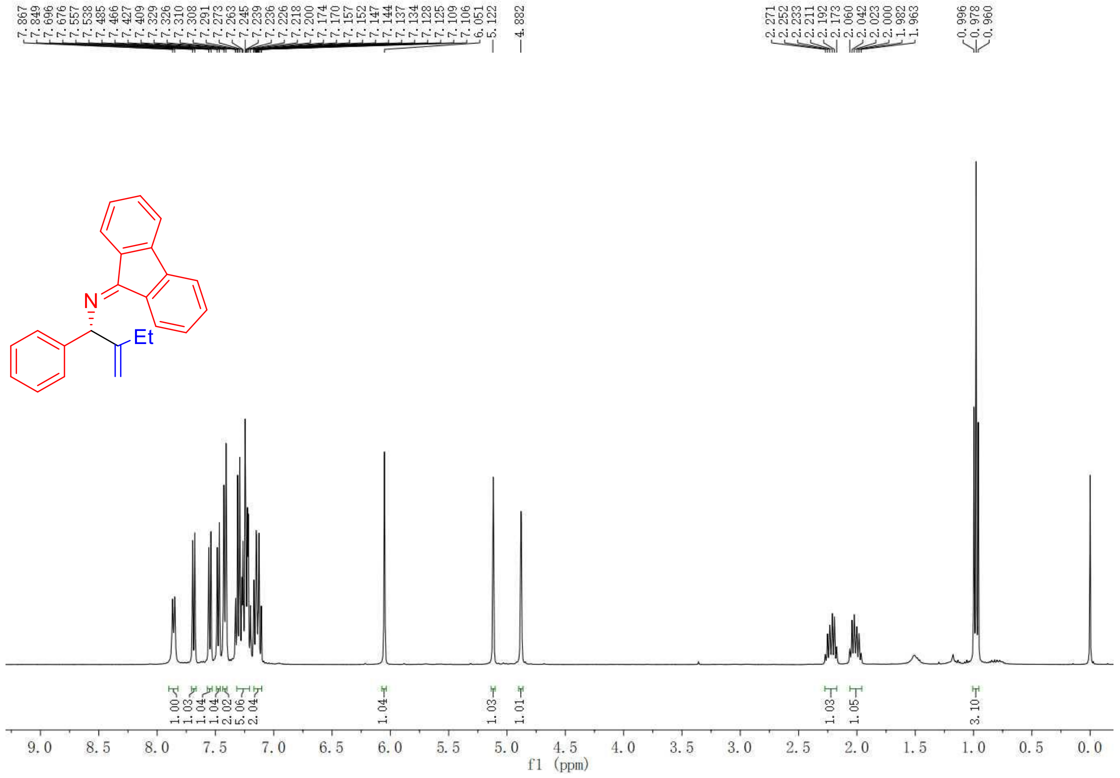

Figure S90. ^{13}C{^{1}H} NMR spectra (100 MHz, Chloroform-*d*) of (*R*)-*N*-(2-Methylene-1-phenylbutyl)-9*H*-fluoren-9-imine (3ac).

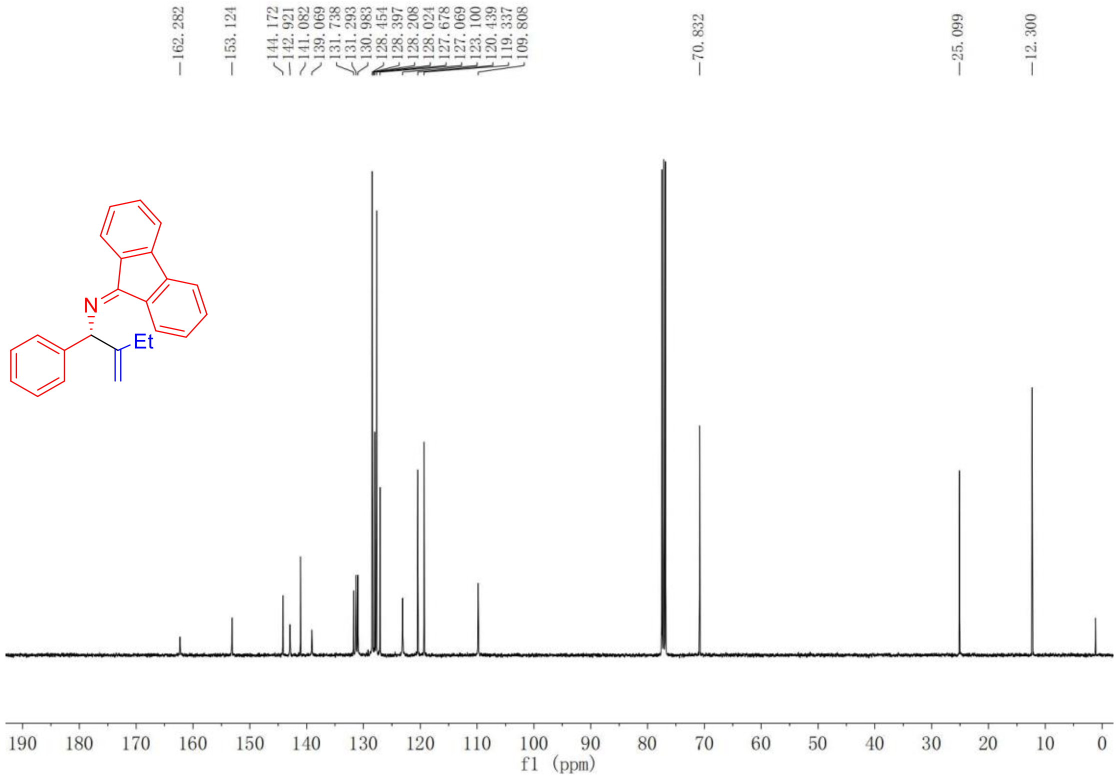

Figure S91. HPLC Chromatography of the Racemic *N*-(2-Methylene-1-phenylbutyl)-9*H*-fluoren-9-imine (3ac).

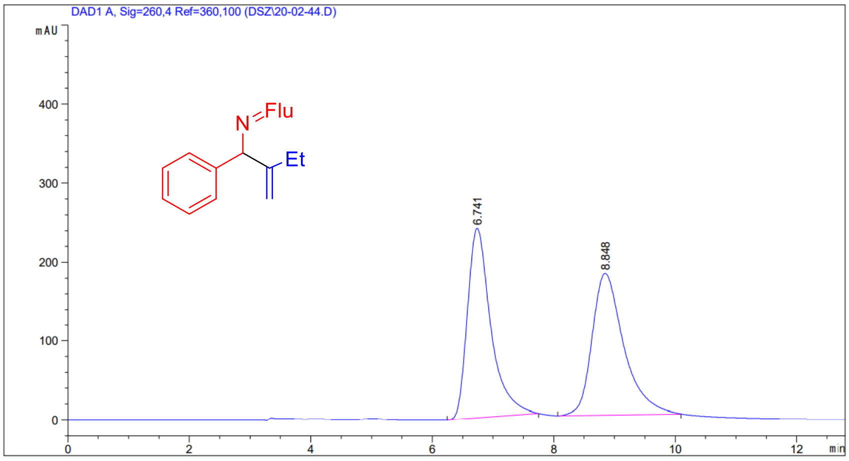

Signal 1: DAD1 A, Sig=260,4 Ref=360,100

Peak #	RetTime [min]	Type	Width [min]	Area [mAU*s]	Height [mAU]	Area %
1	6.741	BBA	0.4080	6423.00879	240.15022	49.6733
2	8.848	BBA	0.5462	6507.48730	179.80382	50.3267
Totals :				1.29305e4	419.95404	

Figure S92. HPLC Chromatography of (*R*)-*N*-(2-Methylene-1-phenylbutyl)-9*H*-fluoren-9-imine (3ac).

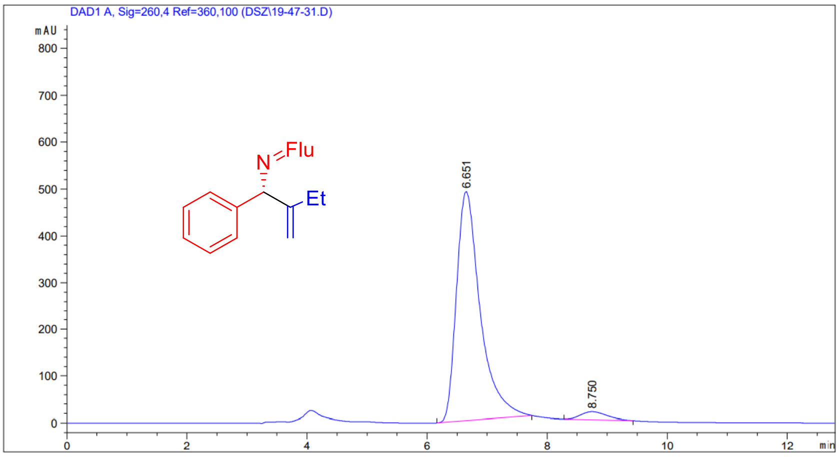

Signal 1: DAD1 A, Sig=260,4 Ref=360,100

Peak #	RetTime [min]	Type	Width [min]	Area [mAU*s]	Height [mAU]	Area %
1	6.651	BBA	0.4150	1.34653e4	489.41470	96.3257
2	8.750	BBA	0.4732	513.63373	16.77465	3.6743
Totals :				1.39790e4	506.18936	

Figure S93. ^{1}H NMR spectra (400 MHz, Chloroform-*d*) of (*R*, *E*)-*N*-(2-Methyl-1-phenylbut-2-en-1-yl)-9*H*-fluoren-9-imine (3ad).

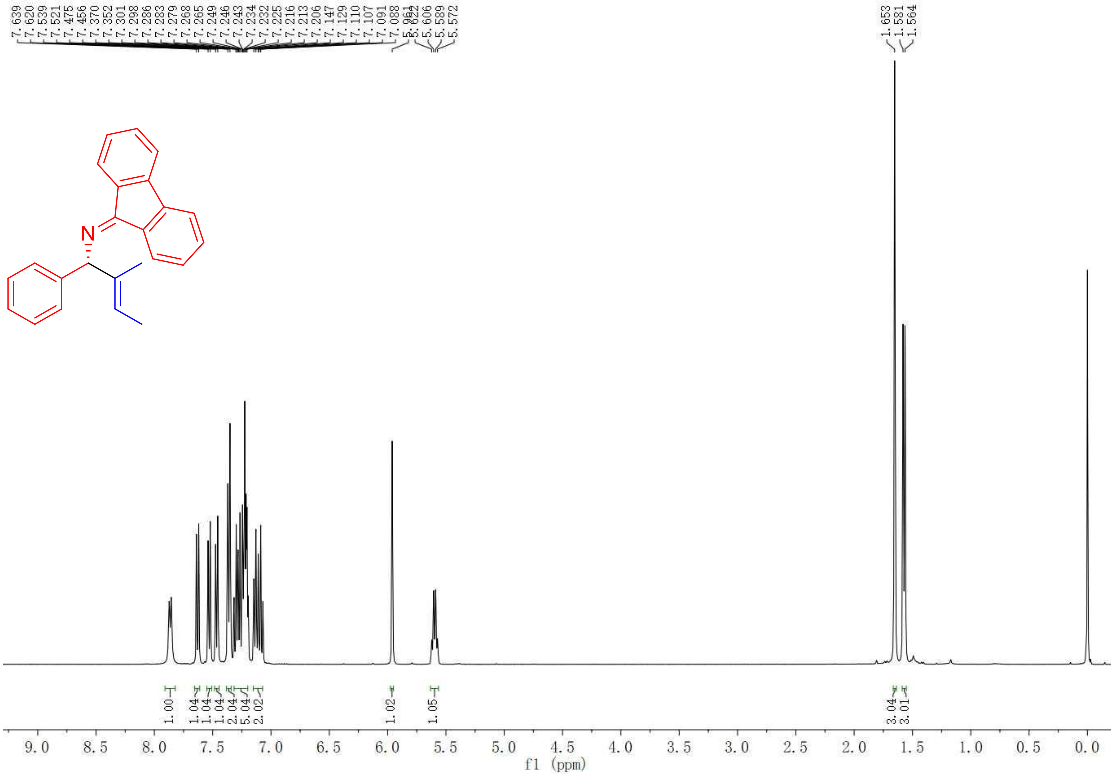

Figure S94. ^{13}C{^{1}H} NMR spectra (100 MHz, Chloroform-*d*) of (*R*, *E*)-*N*-(2-Methyl-1-phenylbut-2-en-1-yl)-9*H*-fluoren-9-imine (3ad).

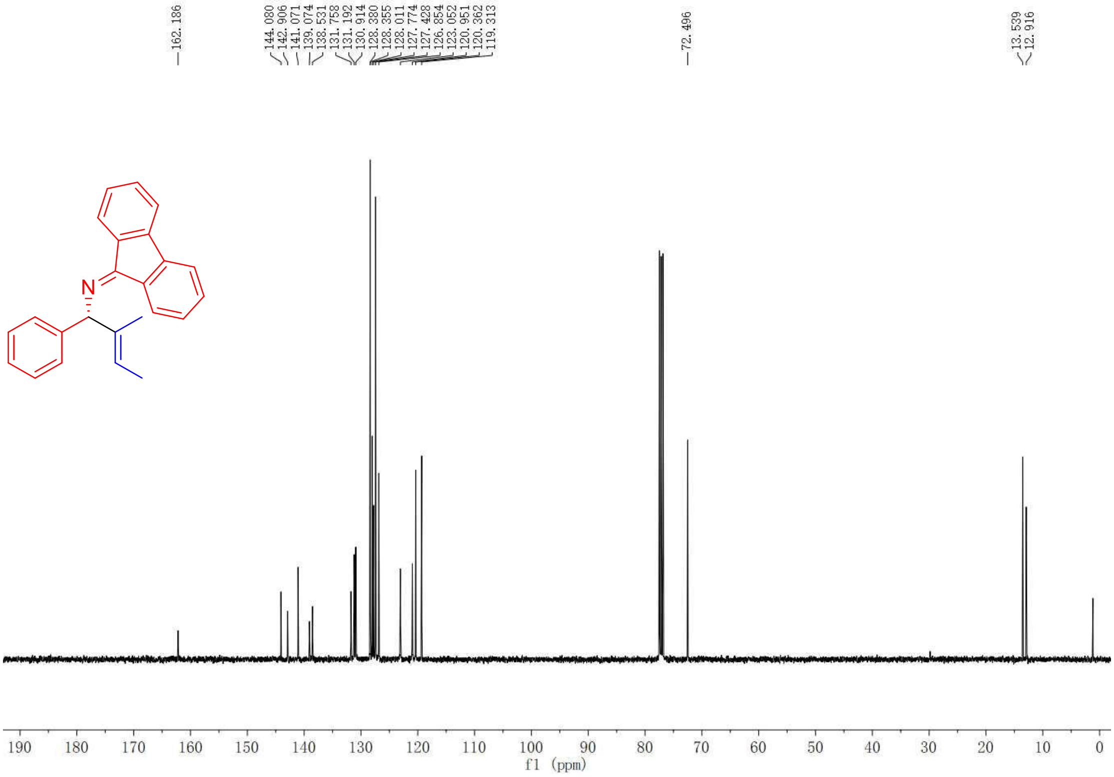

Figure S95. HPLC Chromatography of the Racemic (*E*)-*N*-(2-Methyl-1-phenylbut-2-en-1-yl)-9*H*-fluoren-9-imine (3ad).

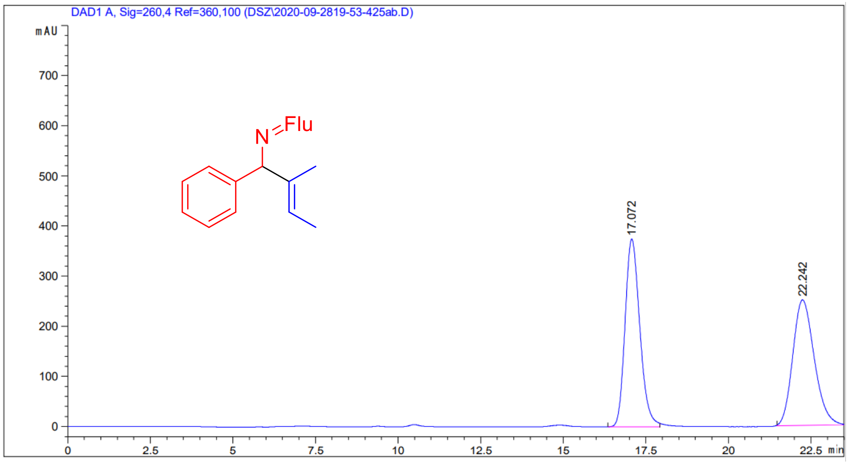

Signal 1: DAD1 A, Sig=260,4 Ref=360,100

Peak #	RetTime [min]	Type	Width [min]	Area [mAU*s]	Height [mAU]	Area %
1	17.072	HHA	0.4778	1.16101e4	374.43155	50.6501
2	22.242	HBA	0.6950	1.13120e4	250.40831	49.3499

Totals : 2.29221e4 624.83986

Figure S96. HPLC Chromatography of (*R*, *E*)-*N*-(2-Methyl-1-phenylbut-2-en-1-yl)-9*H*-fluoren-9-imine (3ad).

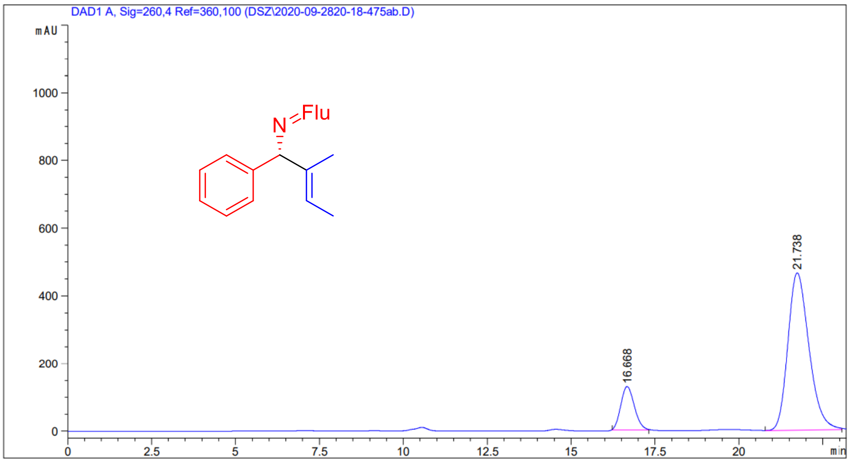

Signal 1: DAD1 A, Sig=260,4 Ref=360,100

Peak #	RetTime [min]	Type	Width [min]	Area [mAU*s]	Height [mAU]	Area %
1	16.668	HHA	0.4396	3588.31348	127.79852	15.0002
2	21.738	BHA	0.6790	2.03335e4	464.22116	84.9998

Totals : 2.39218e4 592.01968

Figure S97. ^{1}H **NMR spectra (400 MHz, Chloroform-*d*) of (*S*)-2-(((9*H*-Fluoren-9-ylidene)amino)(phenyl)methyl)-*N*-benzyl-*N*-methylprop-2-en-1-amine (3ae).**

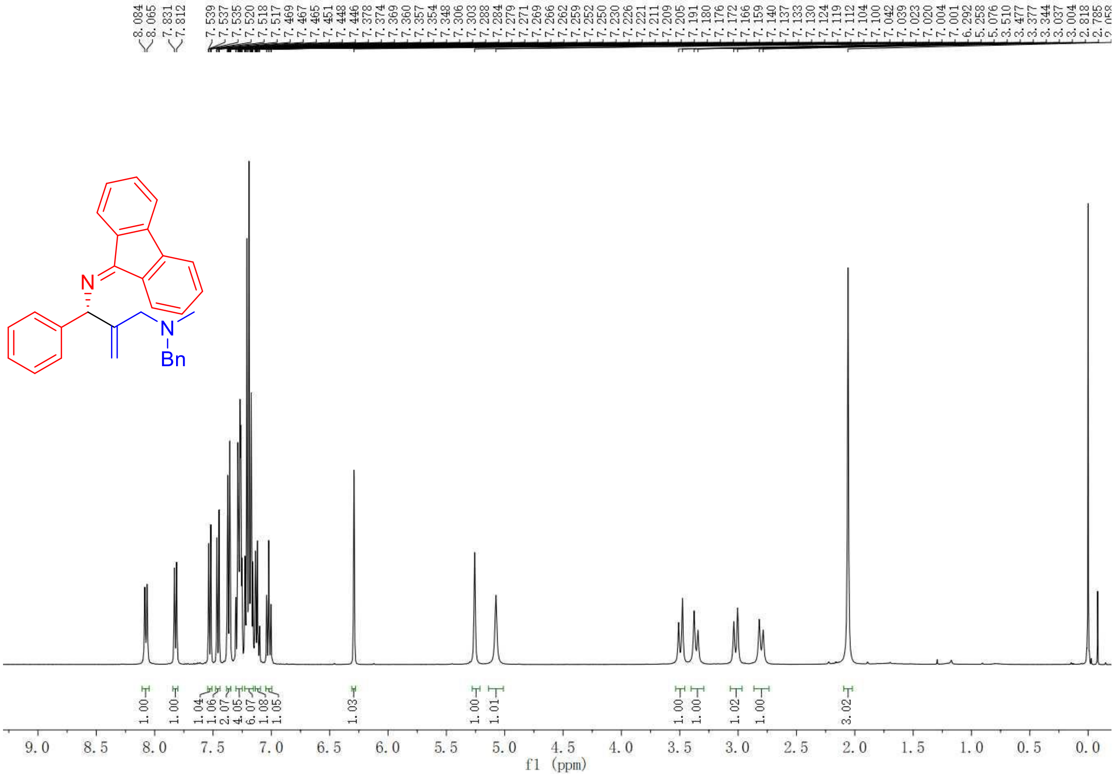

Figure S98. $^{13}C\{^{1}H\}$ **NMR spectra (100 MHz, Chloroform-*d*) of (*S*)-2-(((9*H*-Fluoren-9-ylidene) amino)(phenyl)methyl)-*N*-benzyl-*N*-methylprop-2-en-1-amine (3ae).**

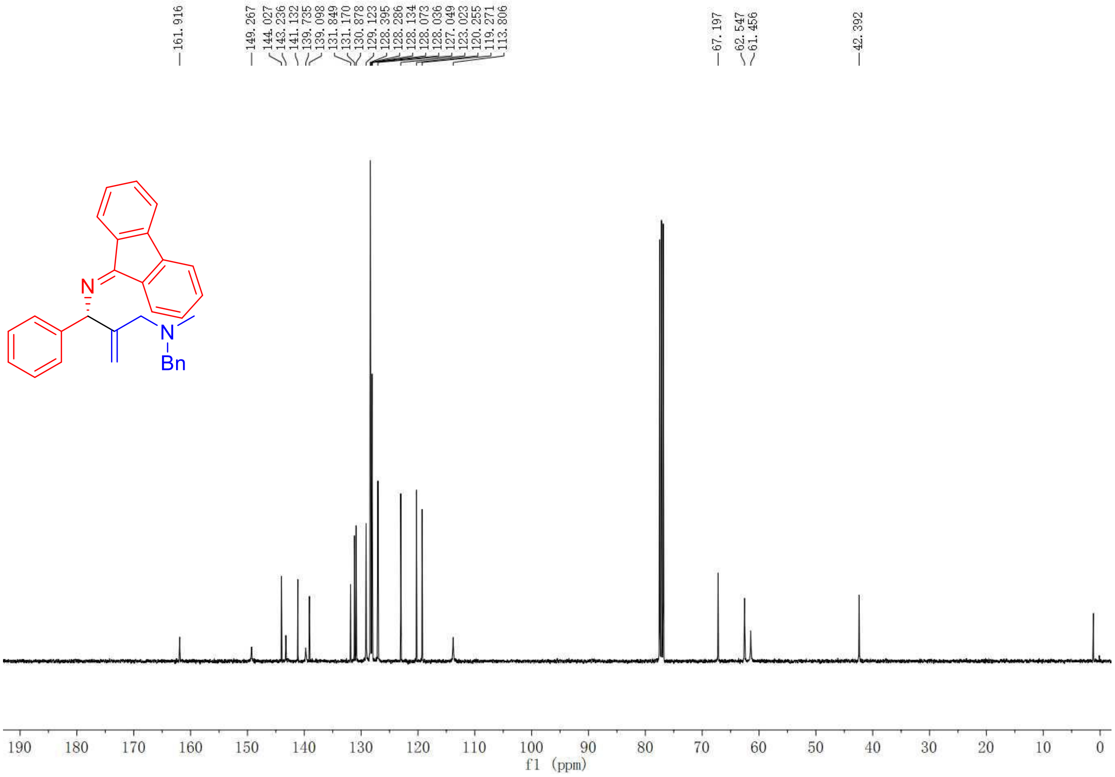

Figure S99. HPLC Chromatography of the Racemic 2-(((9*H*-Fluoren-9-ylidene)amino)(phenyl) methyl)-*N*-benzyl-*N*-methylprop-2-en-1-amine (3ae).

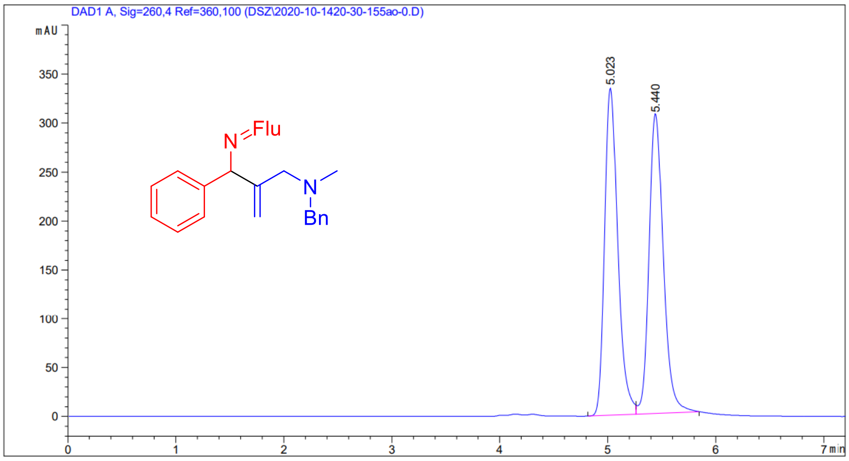

Signal 1: DAD1 A, Sig=260,4 Ref=360,100

Peak #	RetTime [min]	Type	Width [min]	Area [mAU*s]	Height [mAU]	Area %
1	5.023	BV	0.1357	2855.59351	334.28024	50.2406
2	5.440	VBA	0.1484	2828.24438	304.35300	49.7594

Totals : 5683.83789 638.63324

Figure S100. HPLC Chromatography of (*S*)-2-(((9*H*-Fluoren-9-ylidene)amino)(phenyl) methyl)-*N*-benzyl-*N*-methylprop-2-en-1-amine (3ae).

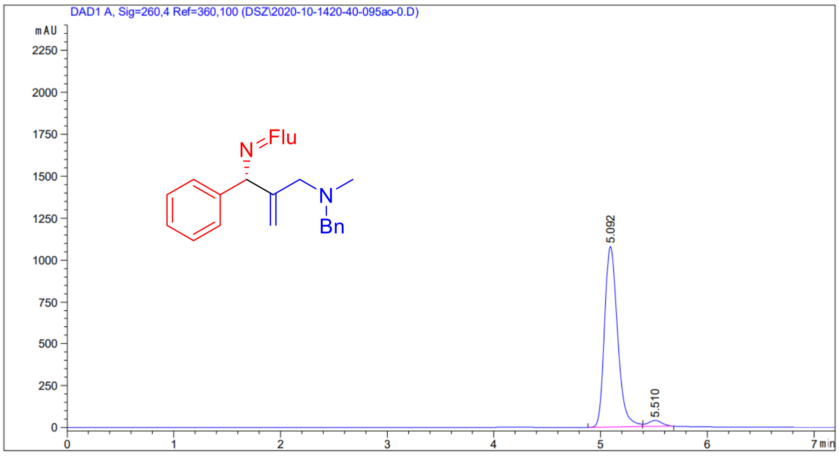

Signal 1: DAD1 A, Sig=260,4 Ref=360,100

Peak #	RetTime [min]	Type	Width [min]	Area [mAU*s]	Height [mAU]	Area %
1	5.092	BF	0.1325	9115.09961	1079.16333	96.4816
2	5.510	VBA	0.1413	332.39771	36.14714	3.5184

Totals : 9447.49731 1115.31047

Figure S101. **^{1}H NMR spectra (400 MHz, Chloroform-*d*) of (*S*)-*N*-(2-(Morpholinomethyl)-1-phenylallyl)-9*H*-fluoren-9-imine (3af).**

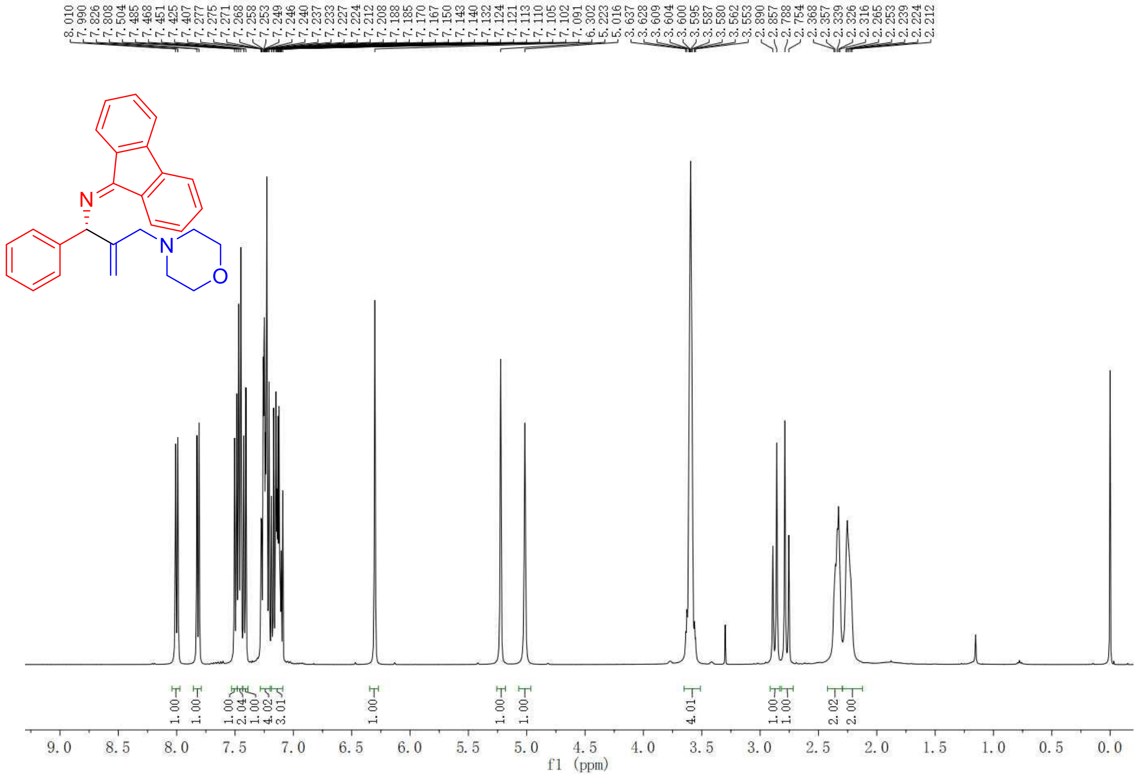

Figure S102. **^{13}C{^{1}H} NMR spectra (100 MHz, Chloroform-*d*) of (*S*)-*N*-(2-(Morpholinomethyl)-1-phenylallyl)-9*H*-fluoren-9-imine (3af).**

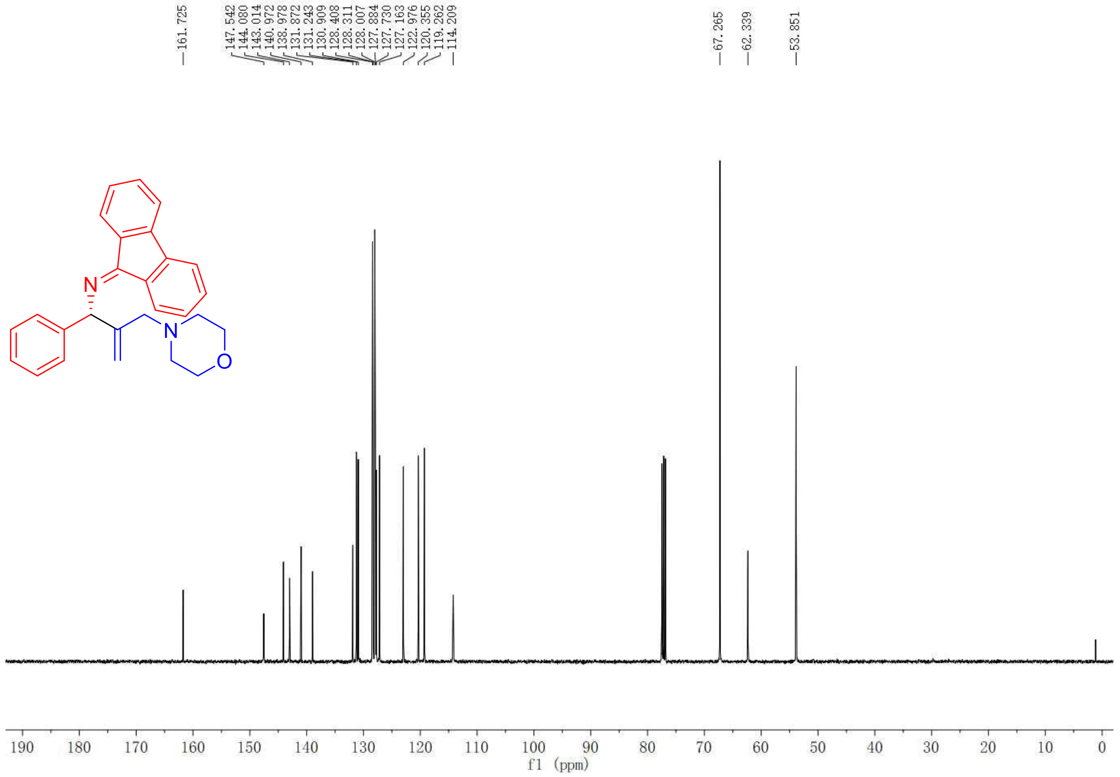

Figure S103. HPLC Chromatography of the Racemic *N*-(2-(Morpholinomethyl)-1-phenylallyl)- 9*H*-fluoren-9-imine (3af).

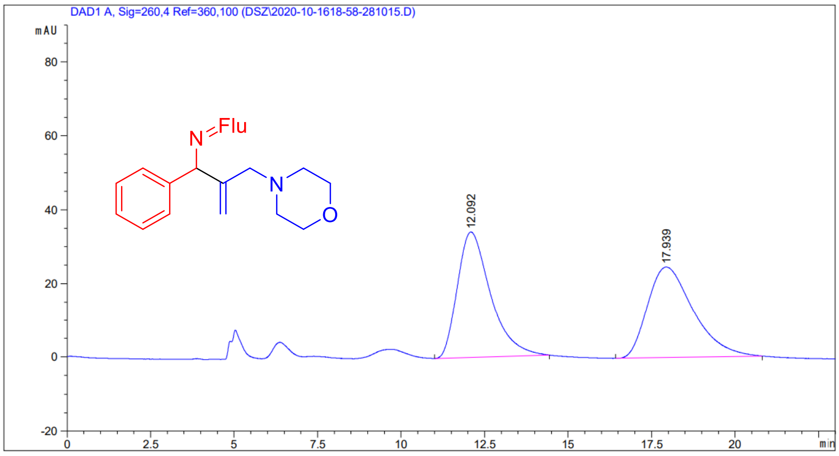

Signal 1: DAD1 A, Sig=260,4 Ref=360,100

Peak #	RetTime [min]	Type	Width [min]	Area [mAU*s]	Height [mAU]	Area %
1	12.092	BBA	1.0482	2362.90967	34.11057	50.0985
2	17.939	BBA	1.4555	2353.62109	24.70341	49.9015

Totals : 4716.53076 58.81398

Figure S104. HPLC Chromatography of (*S*)-*N*-(2-(Morpholinomethyl)-1-phenylallyl)-9*H*-fluoren-9-imine (3af).

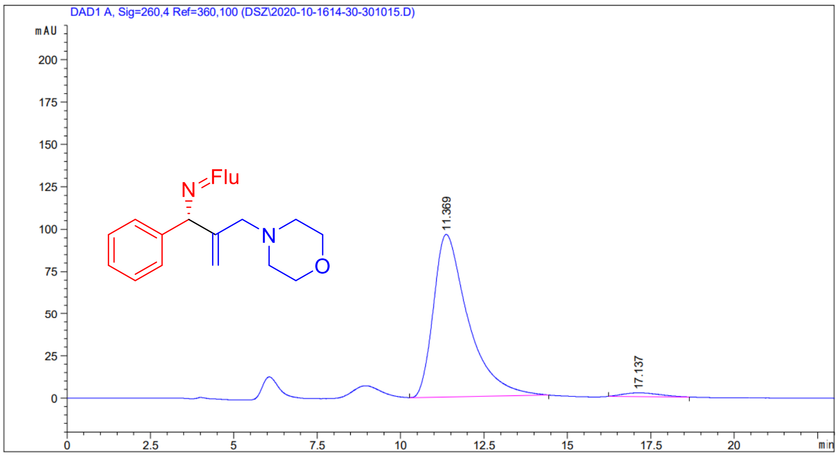

Signal 1: DAD1 A, Sig=260,4 Ref=360,100

Peak #	RetTime [min]	Type	Width [min]	Area [mAU*s]	Height [mAU]	Area %
1	11.369	BBA	1.0575	6949.00098	96.34267	97.5840
2	17.137	HHA	1.2087	172.04750	2.23856	2.4160

Totals : 7121.04848 98.58123

Figure S105. ^{1}H **NMR spectra (400 MHz, Chloroform-*d*) of *tert*-Butyl (*S*)-(1-phenyl-2-(pyrrolidin-1-ylmethyl)allyl)carbamate (3ag).**

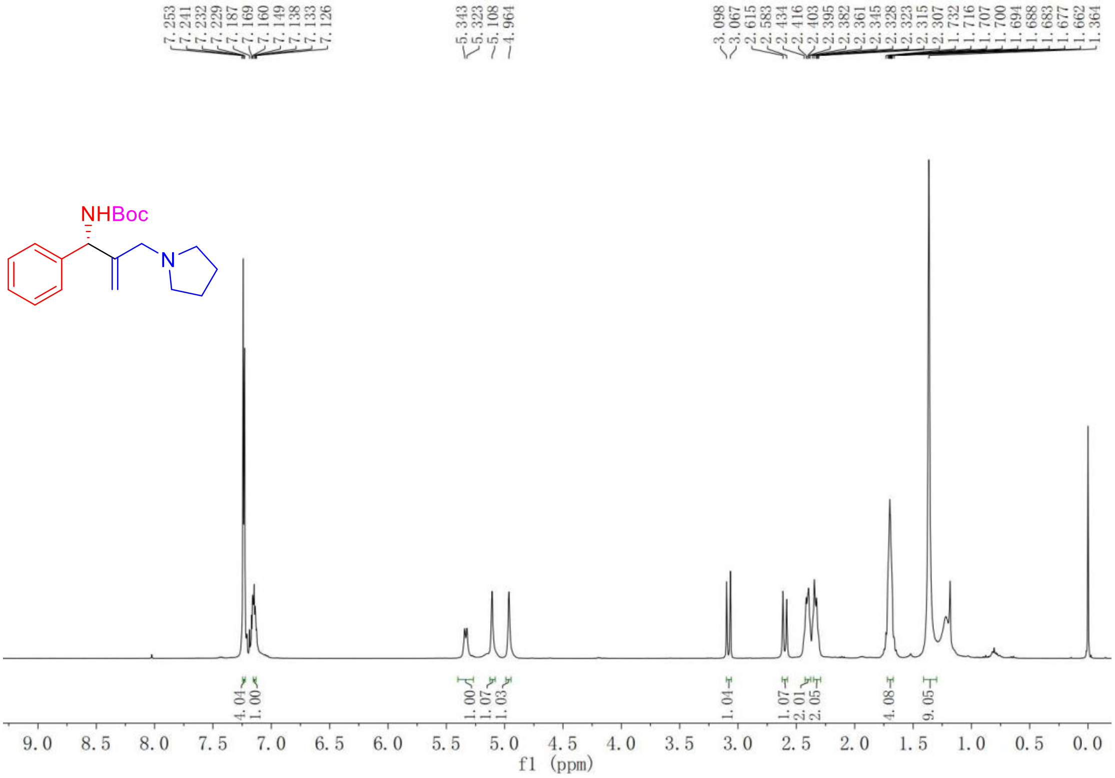

Figure S106. $^{13}C\{^{1}H\}$ **NMR spectra (100 MHz, Chloroform-*d*) of *tert*-Butyl (*S*)-(1-phenyl-2-(pyrrolidin-1-ylmethyl)allyl)carbamate (3ag).**

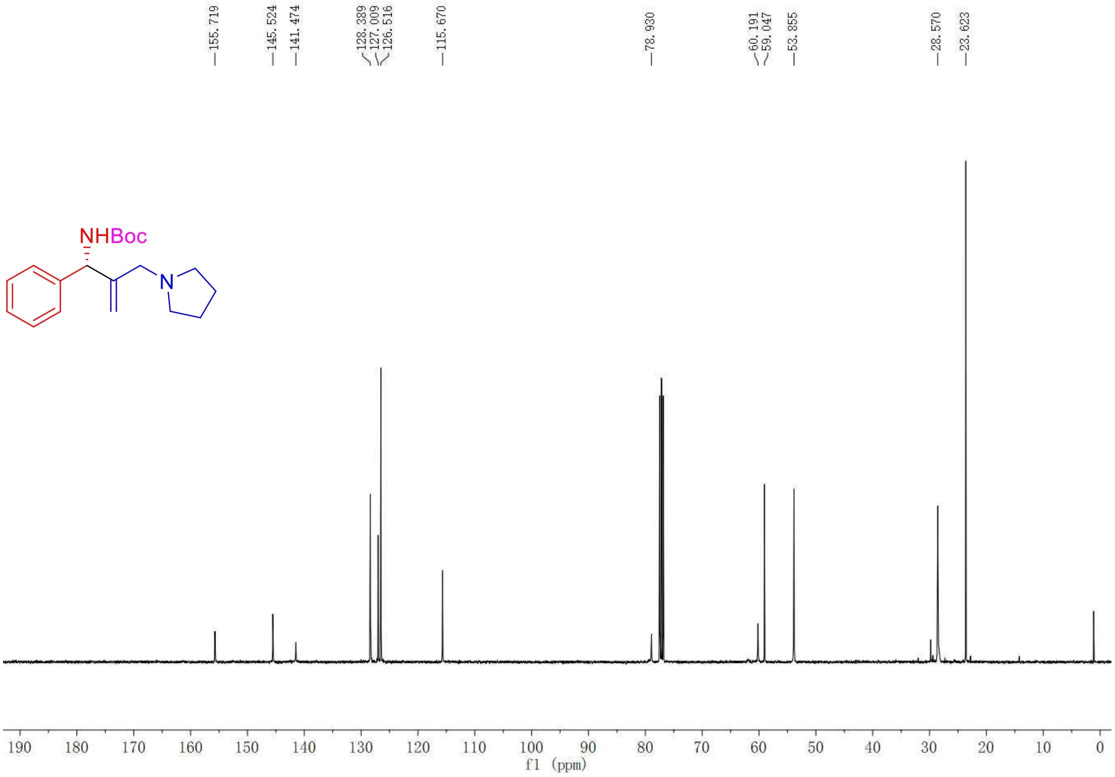

Figure S107. HPLC Chromatography of the Racemic *N*-(1-Phenyl-2-(pyrrolidin-1-ylmethyl) allyl)-9*H*-fluoren-9-imine (3ag).

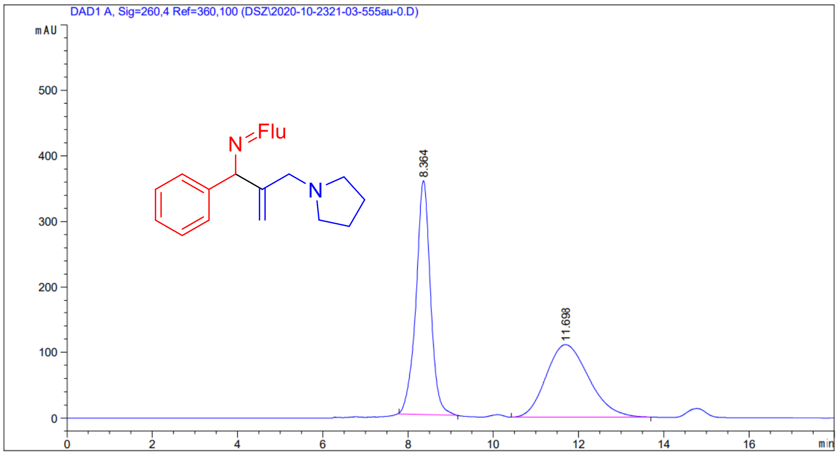

Signal 1: DAD1 A, Sig=260,4 Ref=360,100

Peak #	RetTime [min]	Type	Width [min]	Area [mAU*s]	Height [mAU]	Area %
1	8.364	HHA	0.3550	8414.98145	357.00095	52.4692
2	11.698	HHA	1.0862	7622.95947	109.77183	47.5308

Totals : 1.60379e4 466.77277

Figure S108. HPLC Chromatography of (*S*)-*N*-(1-Phenyl-2-(pyrrolidin-1-ylmethyl)allyl)-9*H*-fluoren-9-imine (3ag).

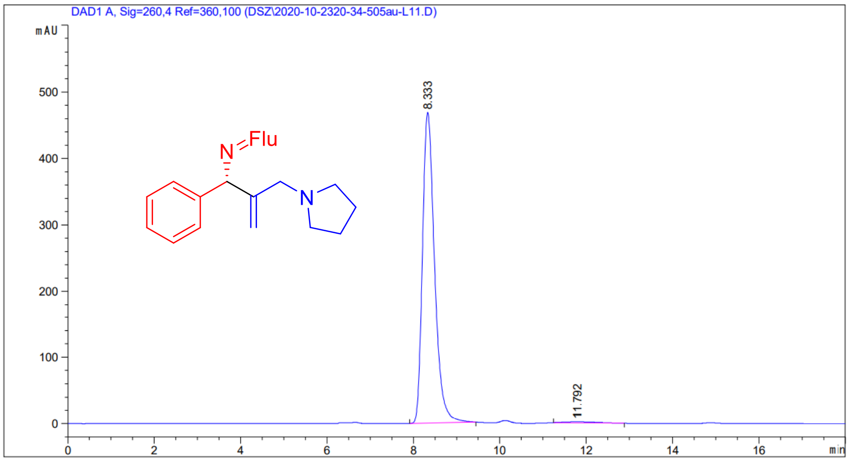

Signal 1: DAD1 A, Sig=260,4 Ref=360,100

Peak #	RetTime [min]	Type	Width [min]	Area [mAU*s]	Height [mAU]	Area %
1	8.333	BBA	0.2986	9098.54590	468.36200	98.9657
2	11.792	HHA	0.8871	95.09370	1.71457	1.0343

Totals : 9193.63960 470.07657

Figure S109. ^{1}H **NMR spectra (400 MHz, Chloroform-*d*) of *tert*-Butyl (*S*)-(1-phenyl-2-(piperidin-1-ylmethyl)allyl)carbamate (3ah).**

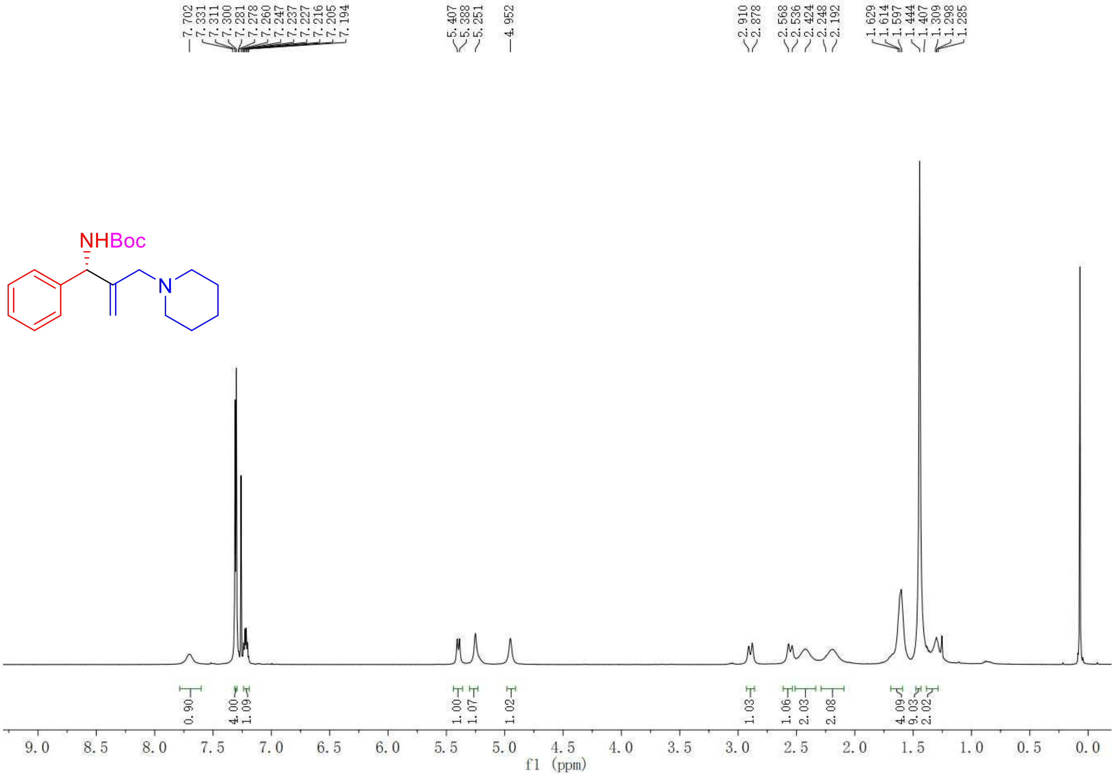

Figure S110. ^{13}C{^{1}H} **NMR spectra (100 MHz, Chloroform-*d*) of *tert*-Butyl (*S*)-(1-phenyl-2-(piperidin-1-ylmethyl)allyl)carbamate (3ah).**

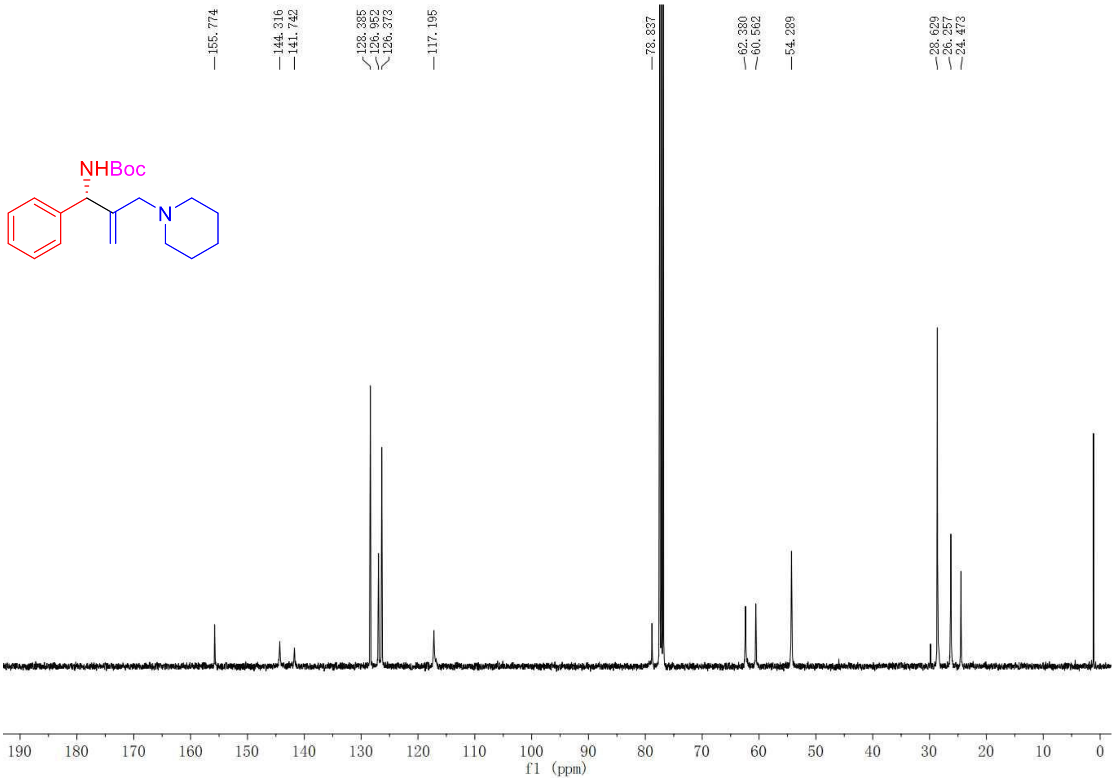

Figure S111. HPLC Chromatography of the Racemic *N*-(1-Phenyl-2-(piperidin-1-ylmethyl) allyl)-9*H*-fluoren-9-imine (3ah).

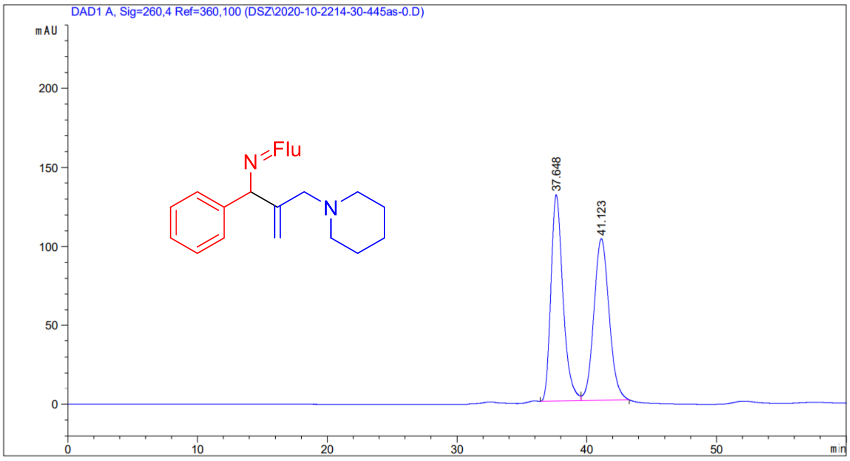

Signal 1: DAD1 A, Sig=260,4 Ref=360,100

Peak #	RetTime [min]	Type	Width [min]	Area [mAU*s]	Height [mAU]	Area %
1	37.648	BV	0.9919	8472.58594	130.76408	50.2899
2	41.123	VBA	1.2484	8374.89648	102.58543	49.7101
Totals :				1.68475e4	233.34951	

Figure S112. HPLC Chromatography of (*S*)-*N*-(1-Phenyl-2-(piperidin-1-ylmethyl)allyl)-9*H*-fluoren-9-imine (3ah).

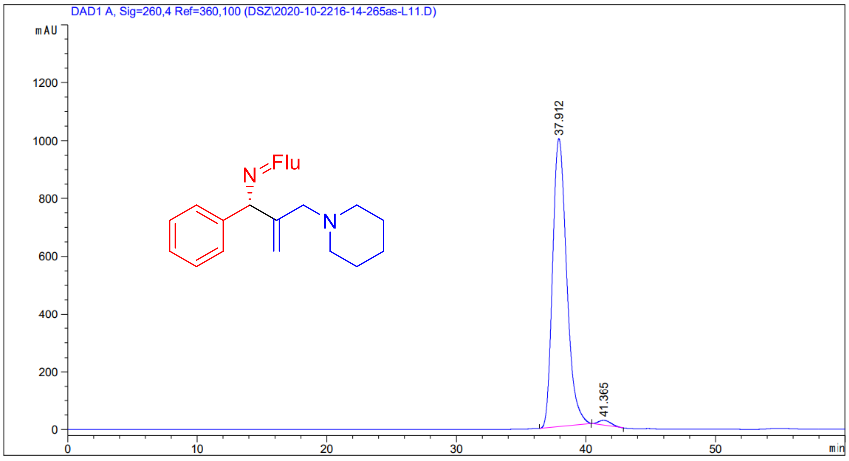

Signal 1: DAD1 A, Sig=260,4 Ref=360,100

Peak #	RetTime [min]	Type	Width [min]	Area [mAU*s]	Height [mAU]	Area %
1	37.912	BBA	1.1713	7.51240e4	996.71564	98.5255
2	41.365	BBA	1.0771	1124.25903	16.70677	1.4745
Totals :				7.62482e4	1013.42241	

Figure S113. ^{1}H NMR spectra (400 MHz, Chloroform-*d*) of (*S*)-*N*-(2-((3,4-Dihydroisoquinolin-2(1*H*)-yl)methyl)-1-phenylallyl)-9*H*-fluoren-9-imine (3ai).

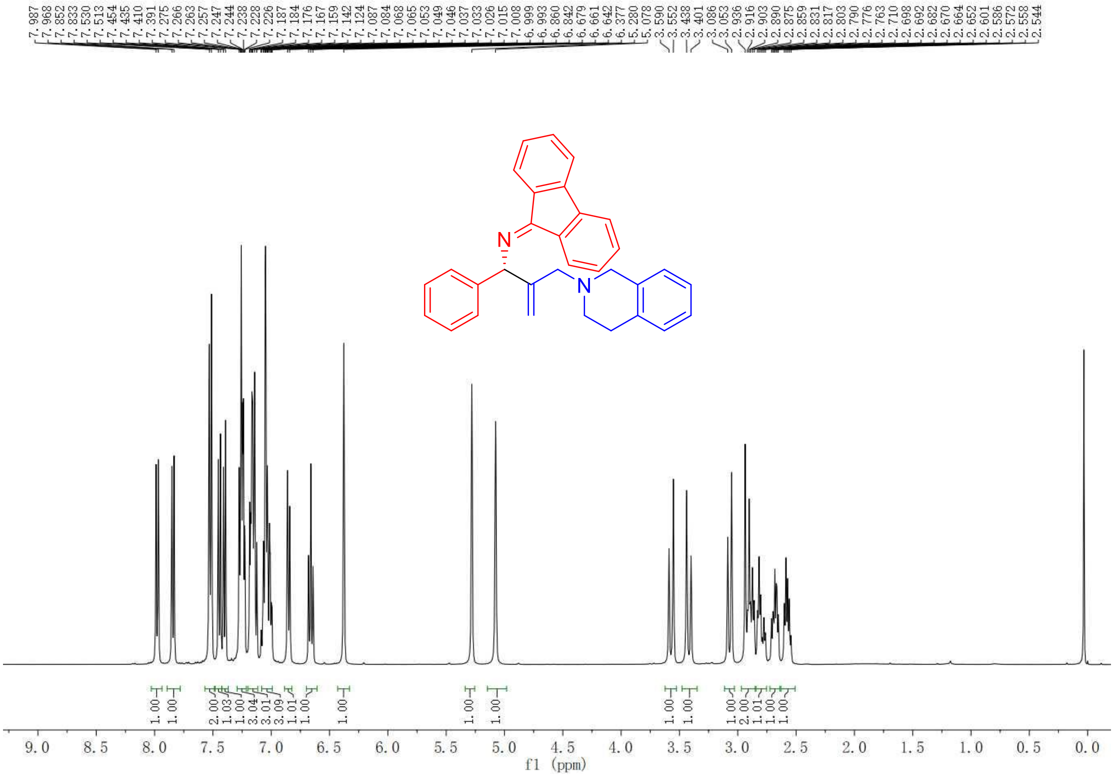

Figure S114. ^{13}C{^{1}H} NMR spectra (100 MHz, Chloroform-*d*) of (*S*)-*N*-(2-((3,4-Dihydroisoquinolin-2(1*H*)-yl)methyl)-1-phenylallyl)-9*H*-fluoren-9-imine (3ai).

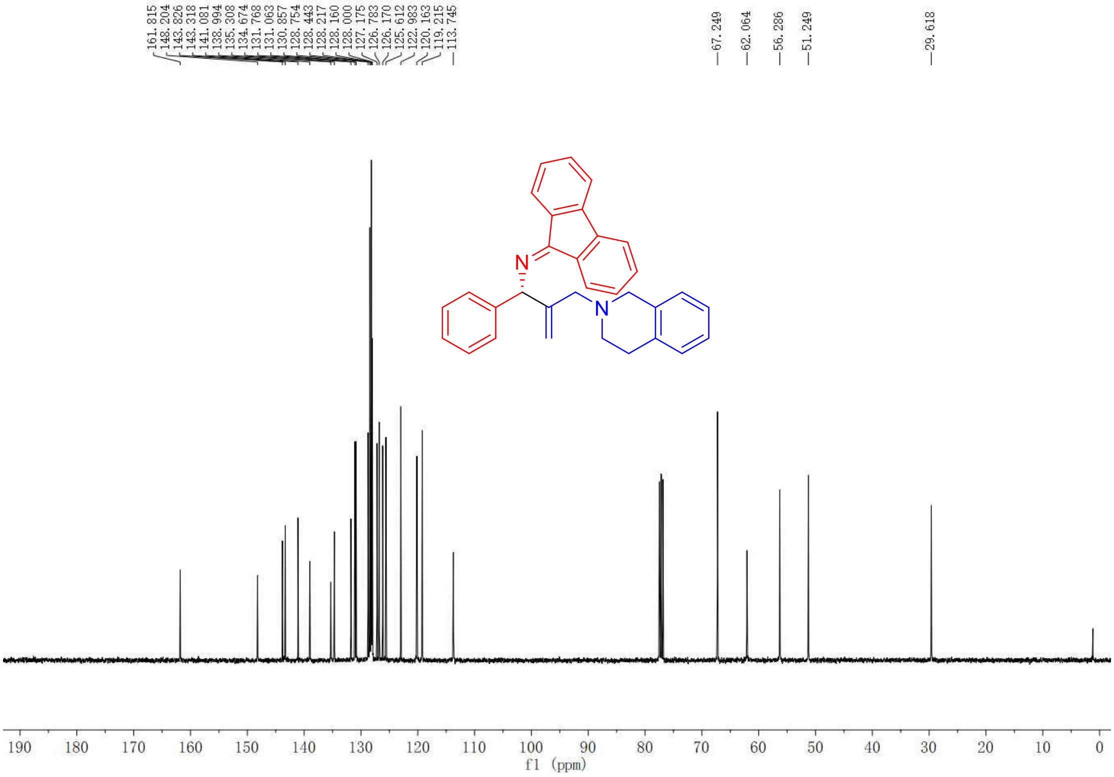

Figure S115. HPLC Chromatography of the Racemic *N*-(2-((3,4-Dihydroisoquinolin-2(1*H*)-yl)methyl)-1-phenylallyl)-9*H*-fluoren-9-imine (3ai).

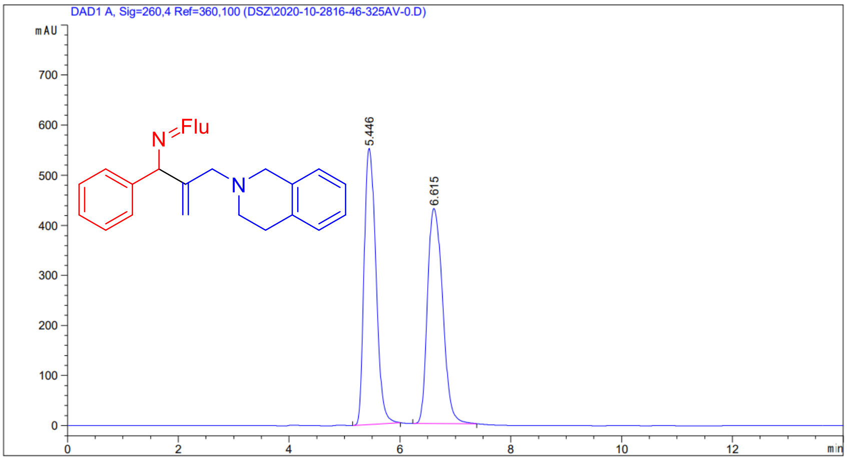

Signal 1: DAD1 A, Sig=260,4 Ref=360,100

Peak #	RetTime [min]	Type	Width [min]	Area [mAU*s]	Height [mAU]	Area %
1	5.446	BBA	0.2371	8165.89160	550.32367	49.9998
2	6.615	BBA	0.3096	8165.94385	430.00793	50.0002

Totals : 1.63318e4 980.33160

Figure S116. HPLC Chromatography of (*S*)-*N*-(2-((3,4-Dihydroisoquinolin-2(1*H*)-yl)methyl)-1-phenylallyl)-9*H*-fluoren-9-imine (3ai).

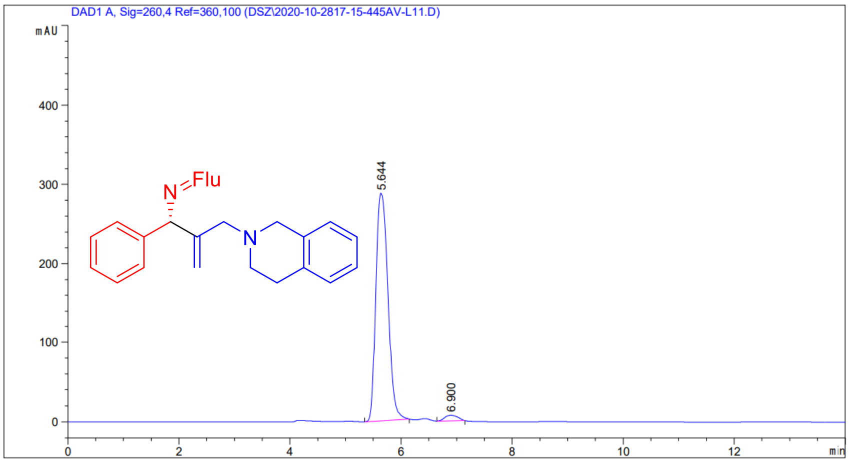

Signal 1: DAD1 A, Sig=260,4 Ref=360,100

Peak #	RetTime [min]	Type	Width [min]	Area [mAU*s]	Height [mAU]	Area %
1	5.644	BBA	0.2439	4336.20996	287.59045	97.4556
2	6.900	BBA	0.2696	113.21263	6.96725	2.5444

Totals : 4449.42259 294.55770

Figure S117. ^{1}**H NMR spectra (400 MHz, Chloroform-*d*) of (*S*)-2-(((9*H*-Fluoren-9-ylidene) amino)(2,3-dihydrobenzofuran-5-yl)methyl)-*N*-benzyl-*N*-methylprop-2-en-1-amine (3je).**

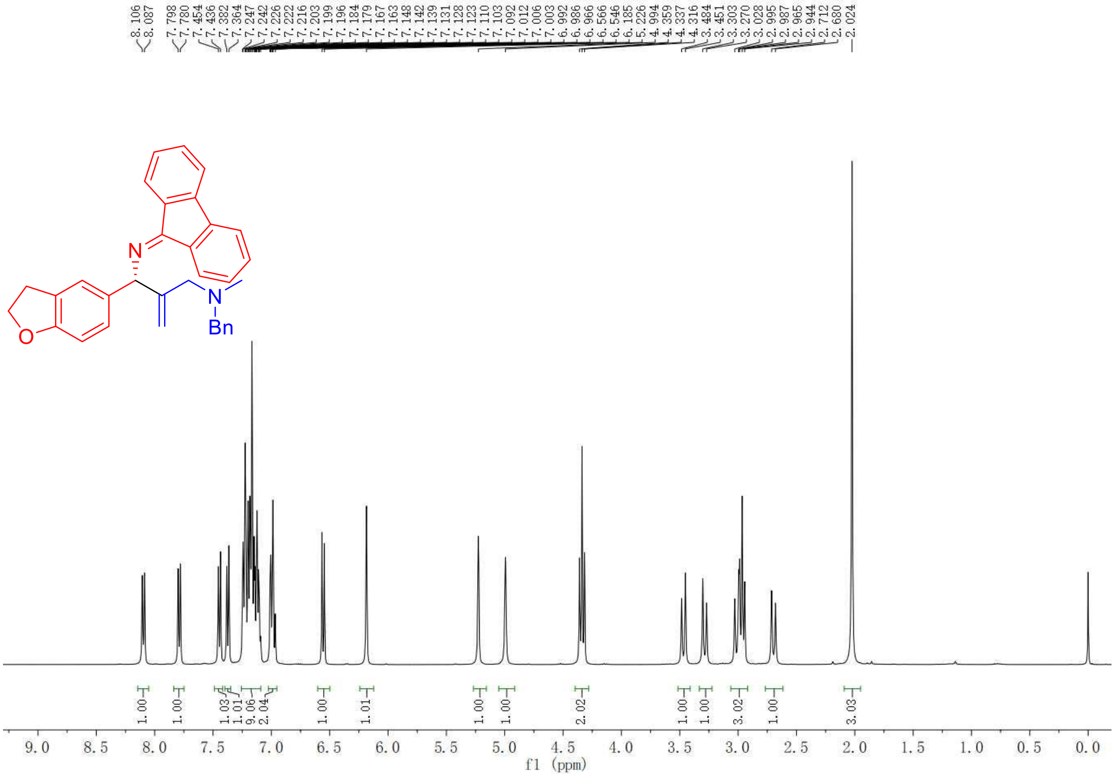

Figure S118. ^{13}**C{^{1}H} NMR spectra (100 MHz, Chloroform-*d*) of (*S*)-2-(((9*H*-Fluoren-9-ylidene) amino)(2,3-dihydrobenzofuran-5-yl)methyl)-*N*-benzyl-*N*-methylprop-2-en-1-amine (3je).**

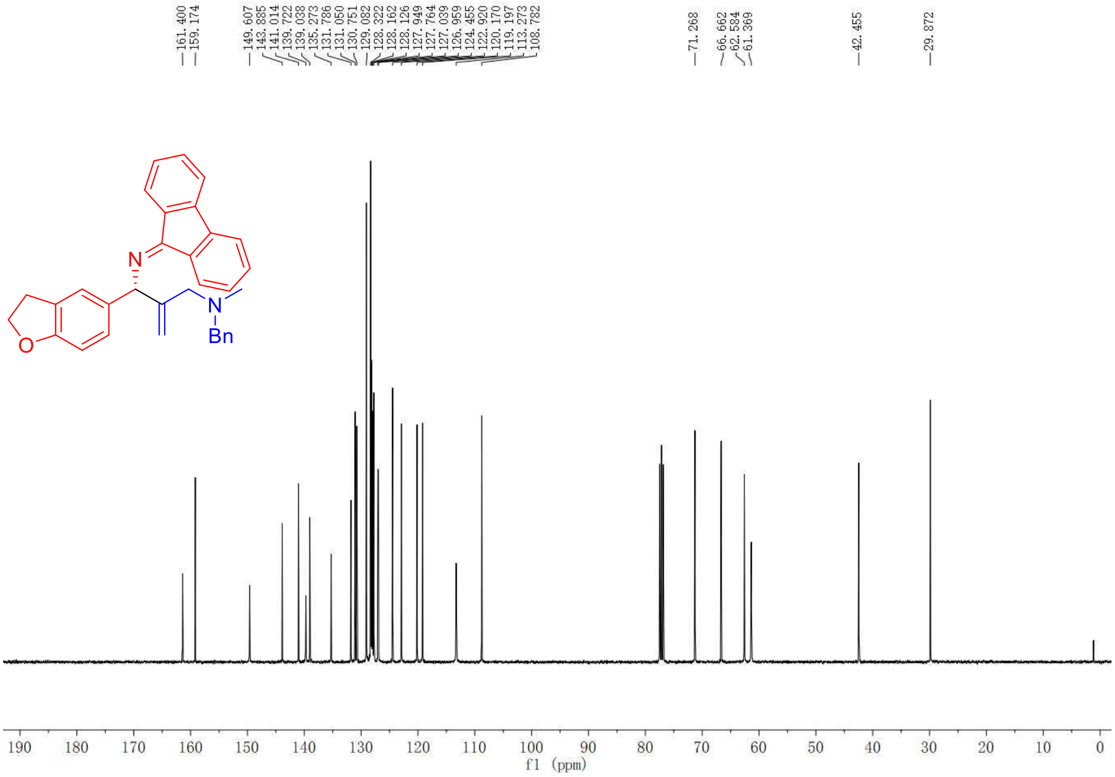

Figure S119. HPLC Chromatography of the Racemic 2-(((9*H*-Fluoren-9-ylidene)amino) (2,3-dihydrobenzofuran-5-yl)methyl)-*N*-benzyl-*N*-methylprop-2-en-1-amine (3je).

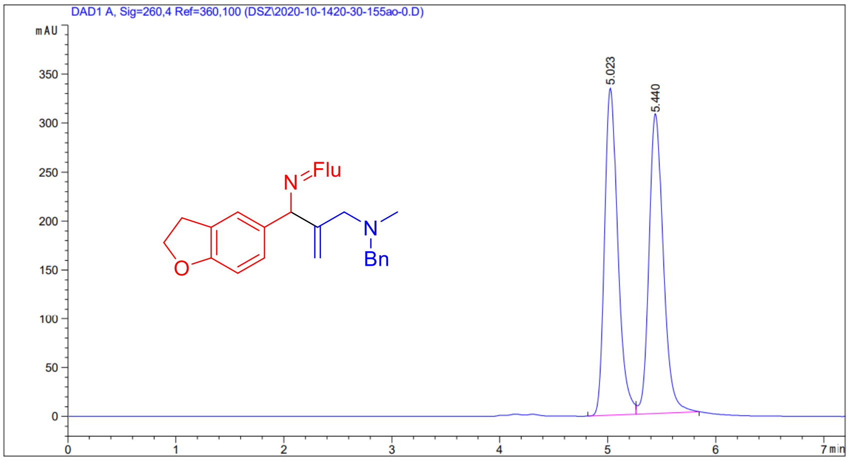

Signal 1: DAD1 A, Sig=260,4 Ref=360,100

Peak #	RetTime [min]	Type	Width [min]	Area [mAU*s]	Height [mAU]	Area %
1	5.023	BV	0.1357	2855.59351	334.28024	50.2406
2	5.440	VBA	0.1484	2828.24438	304.35300	49.7594

Totals : 5683.83789 638.63324

Figure S120. HPLC Chromatography of (*S*)-2-(((9*H*-Fluoren-9-ylidene)amino)(2,3-dihydrobenzofuran-5-yl)methyl)-*N*-benzyl-*N*-methylprop-2-en-1-amine (3je).

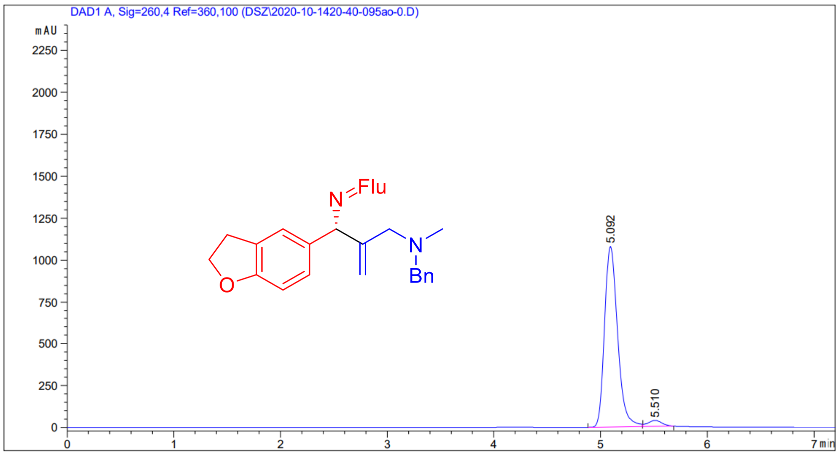

Signal 1: DAD1 A, Sig=260,4 Ref=360,100

Peak #	RetTime [min]	Type	Width [min]	Area [mAU*s]	Height [mAU]	Area %
1	5.092	BF	0.1325	9115.09961	1079.16333	96.4816
2	5.510	VBA	0.1413	332.39771	36.14714	3.5184

Totals : 9447.49731 1115.31047

Figure S121. **^{1}H NMR spectra (400 MHz, Chloroform-*d*) of (*S*)-*N*-(1-(2,3-Dihydrobenzofuran-5-yl)-2-(morpholinomethyl)allyl)-9*H*-fluoren-9-imine (3jf).**

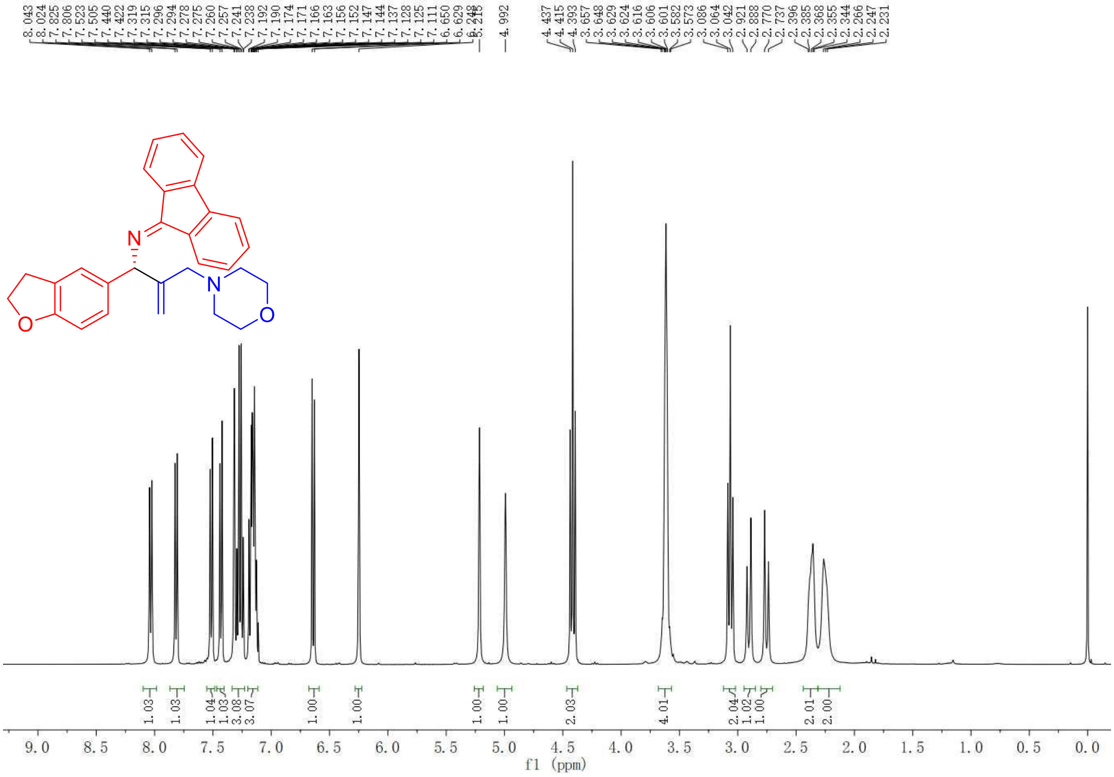

Figure S122. **^{13}C{^{1}H} NMR spectra (100 MHz, Chloroform-*d*) of (*S*)-*N*-(1-(2,3-Dihydrobenzofuran-5-yl)-2-(morpholinomethyl)allyl)-9*H*-fluoren-9-imine (3jf).**

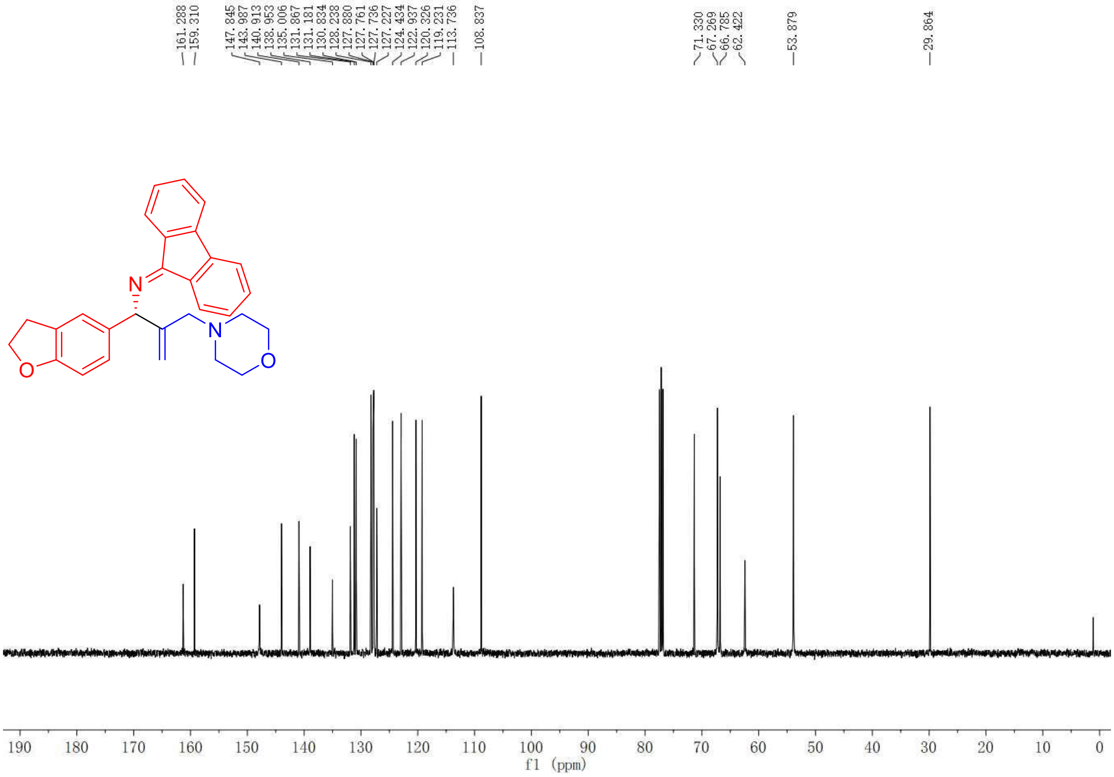

Figure S123. HPLC Chromatography of the Racemic *N*-(1-(2,3-Dihydrobenzofuran-5-yl)-2-(morpholinomethyl)allyl)-9*H*-fluoren-9-imine (3jf).

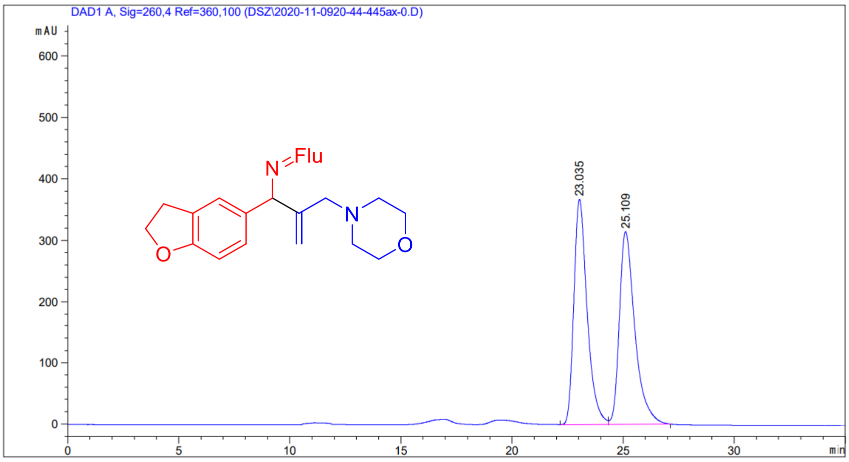

Signal 1: DAD1 A, Sig=260,4 Ref=360,100

Peak #	RetTime [min]	Type	Width [min]	Area [mAU*s]	Height [mAU]	Area %
1	23.035	BV	0.6290	1.52687e4	367.29022	50.0151
2	25.109	VBA	0.7336	1.52595e4	314.77200	49.9849

Totals : 3.05282e4 682.06223

Figure S124. HPLC Chromatography of (*S*)-*N*-(1-(2,3-Dihydrobenzofuran-5-yl)-2-(morpholinomethyl)allyl)-9*H*-fluoren-9-imine (3jf).

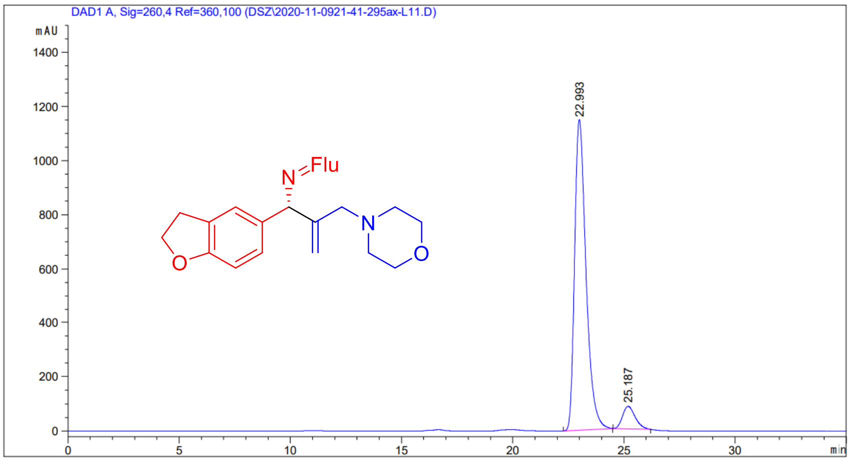

Signal 1: DAD1 A, Sig=260,4 Ref=360,100

Peak #	RetTime [min]	Type	Width [min]	Area [mAU*s]	Height [mAU]	Area %
1	22.993	BBA	0.5511	4.08007e4	1146.71045	92.6228
2	25.187	BBA	0.6095	3249.67700	82.86861	7.3772

Totals : 4.40504e4 1229.57906

Figure S125. ¹H NMR spectra (400 MHz, Chloroform-*d*) of (*S*)-*N*-(1-(2,3-Dihydrobenzofuran-5-yl)-2-((3,4-dihydroisoquinolin-2(1*H*)-yl)methyl)allyl)-9*H*-fluoren-9-imine (3ji).

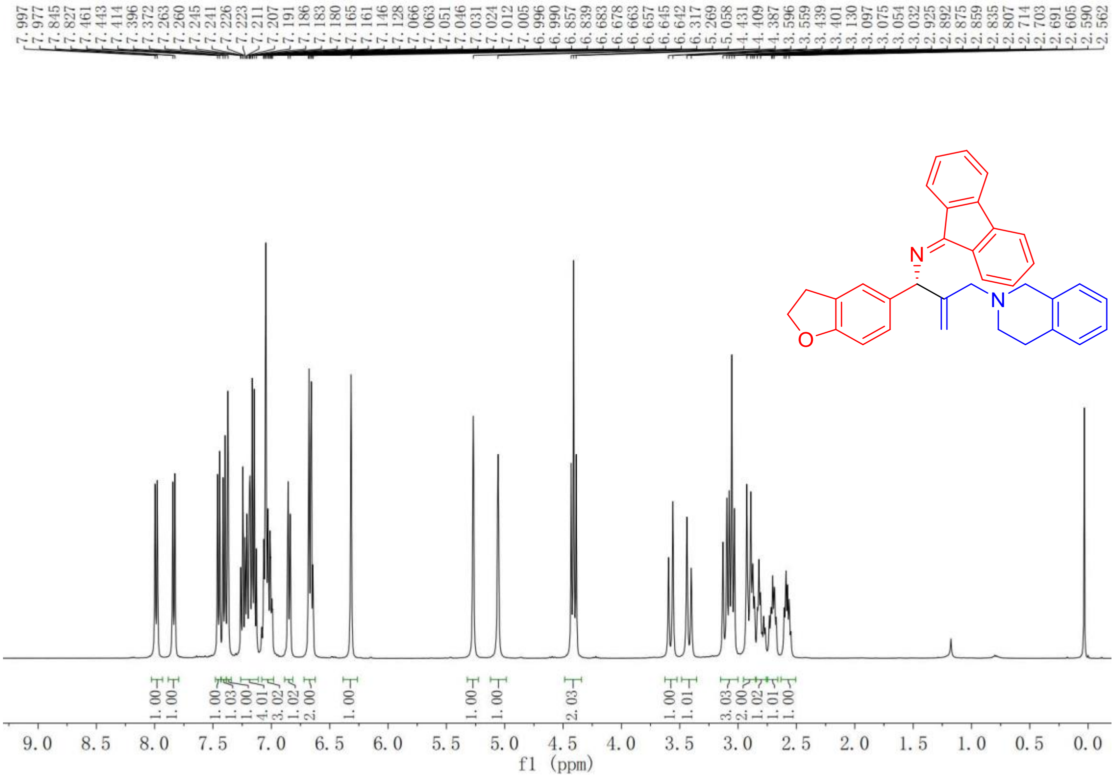

Figure S126. ¹³C{¹H} NMR spectra (100 MHz, Chloroform-*d*) of (*S*)-*N*-(1-(2,3-Dihydrobenzofuran-5-yl)-2-((3,4-dihydroisoquinolin-2(1*H*)-yl)methyl)allyl)-9*H*-fluoren-9-imine (3ji).

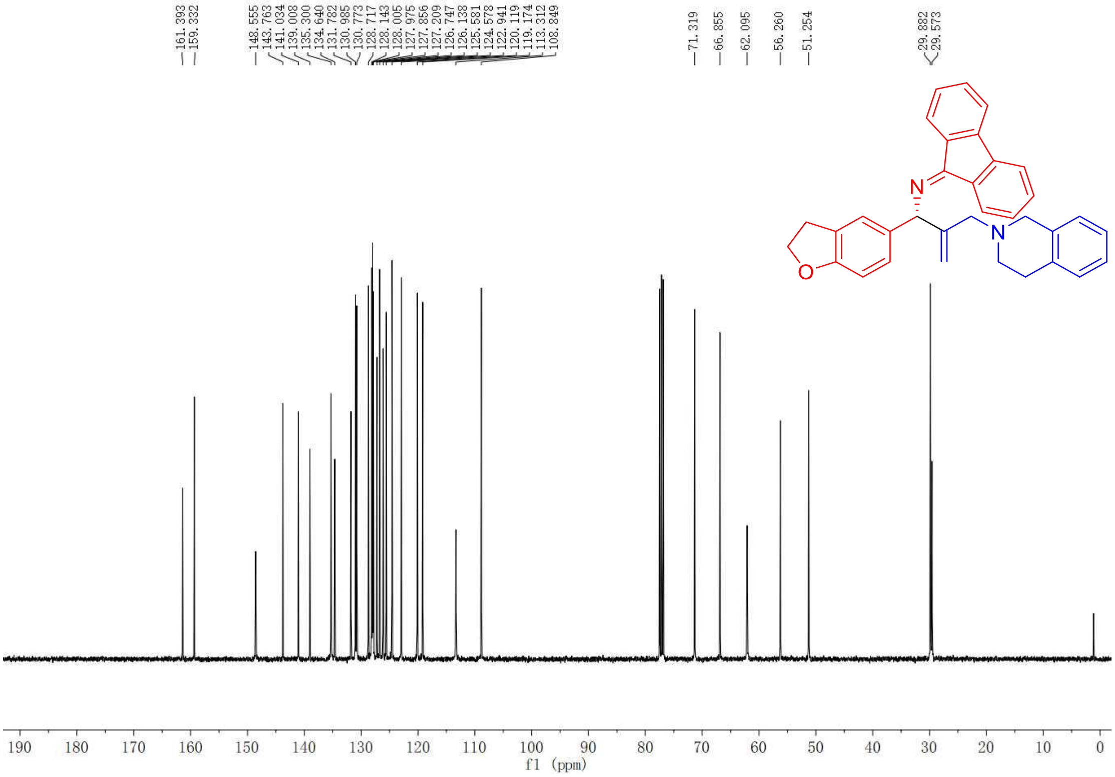

Figure S127. HPLC Chromatography of the Racemic *N*-(1-(2,3-Dihydrobenzofuran-5-yl)-2-((3,4- dihydroisoquinolin-2(1*H*)-yl)methyl)allyl)-9*H*-fluoren-9-imine (3ji).

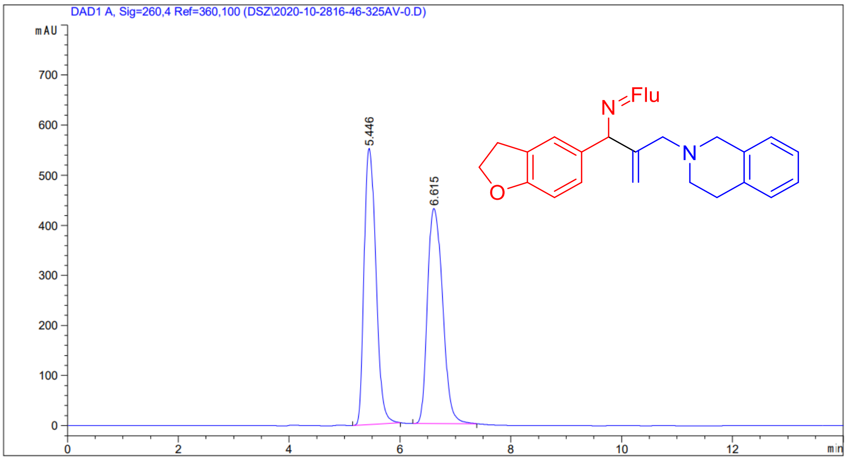

Signal 1: DAD1 A, Sig=260,4 Ref=360,100

Peak #	RetTime [min]	Type	Width [min]	Area [mAU*s]	Height [mAU]	Area %
1	5.446	BBA	0.2371	8165.89160	550.32367	49.9998
2	6.615	BBA	0.3096	8165.94385	430.00793	50.0002

Totals : 1.63318e4 980.33160

Figure S128. HPLC Chromatography of (*S*)-*N*-(1-(2,3-Dihydrobenzofuran-5-yl)-2-((3,4-dihydroisoquinolin-2(1*H*)-yl)methyl)allyl)-9*H*-fluoren-9-imine (3ji).

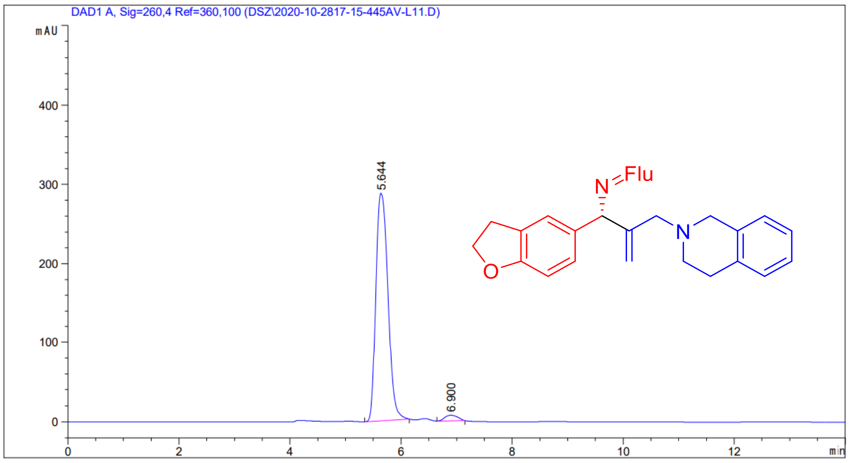

Signal 1: DAD1 A, Sig=260,4 Ref=360,100

Peak #	RetTime [min]	Type	Width [min]	Area [mAU*s]	Height [mAU]	Area %
1	5.644	BBA	0.2439	4336.20996	287.59045	97.4556
2	6.900	BBA	0.2696	113.21263	6.96725	2.5444

Totals : 4449.42259 294.55770

Figure S129. ^{1}H **NMR spectra (400 MHz, Chloroform-*d*) of (*R*)-4-Methyl-*N*-(2-methyl-1-(naphthalen-1-yl)allyl)benzenesulfonamide (4ka).**

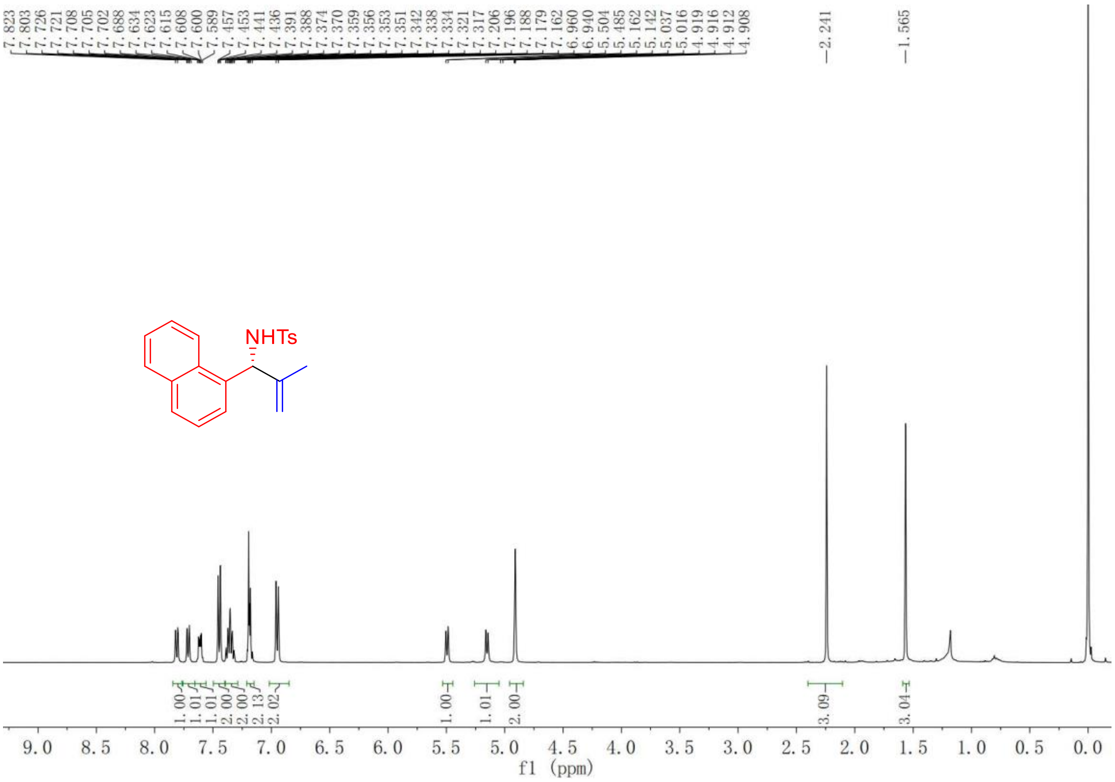

Figure S130. ^{13}C{^{1}H} **NMR spectra (100 MHz, Chloroform-*d*) of (*R*)-4-Methyl-*N*-(2-methyl-1-(naphthalen-1-yl)allyl)benzenesulfonamide (4ka).**

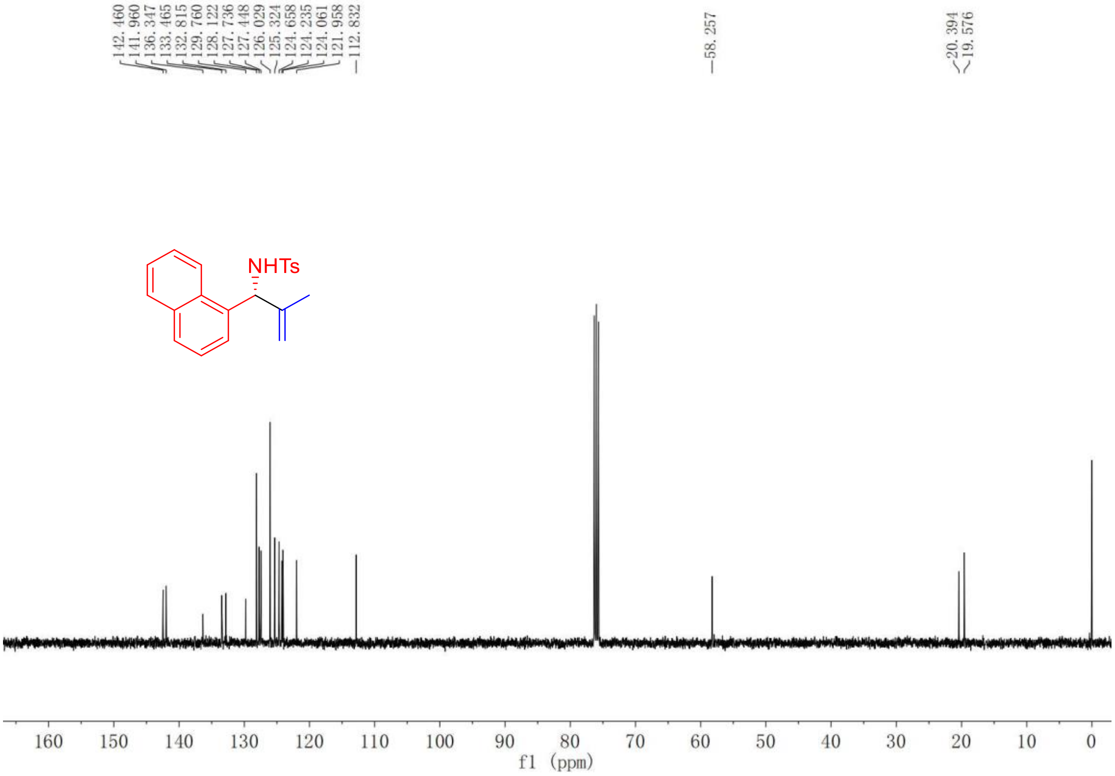

Figure S131. **^{1}H NMR spectra (400 MHz, Chloroform-*d*) of (*R*)-*N*-(1-(2,3-Dihydrobenzofuran-5-yl)-2-methylallyl)-4-methylbenzenesulfonamide (4la).**

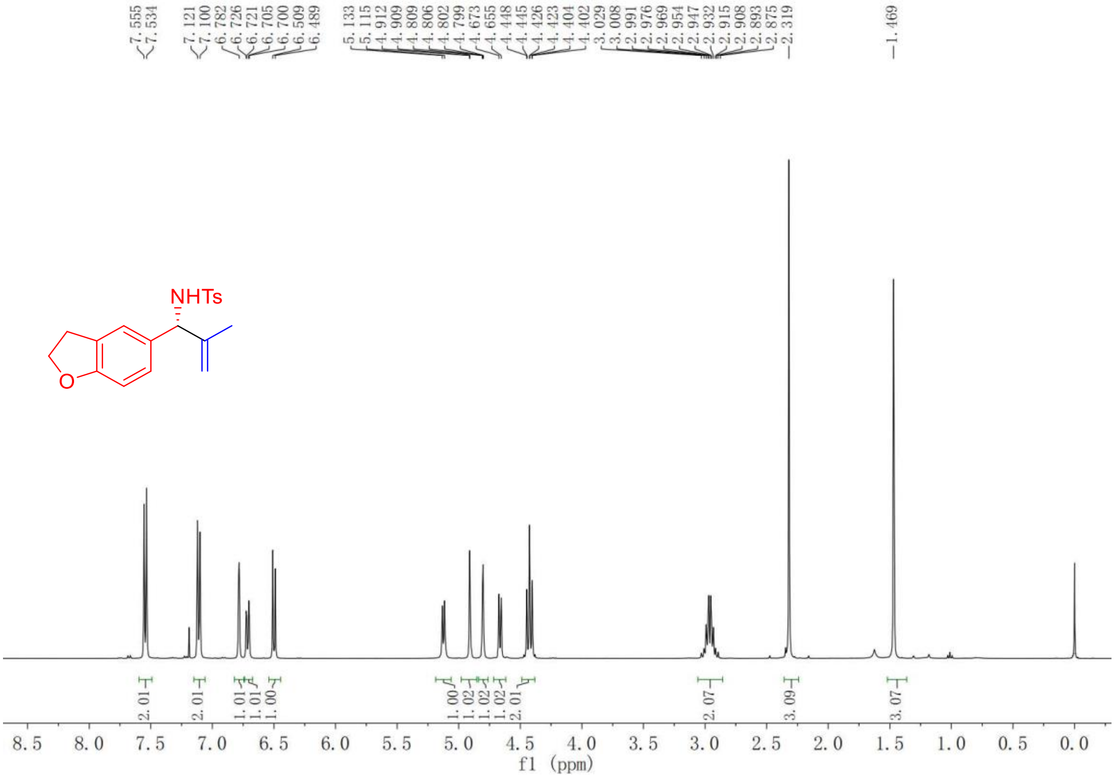

Figure S132. **$^{13}C\{^{1}H\}$ NMR spectra (100 MHz, Chloroform-*d*) of (*R*)-*N*-(1-(2,3-Dihydrobenzofuran-5-yl)-2-methylallyl)-4-methylbenzenesulfonamide (4la).**

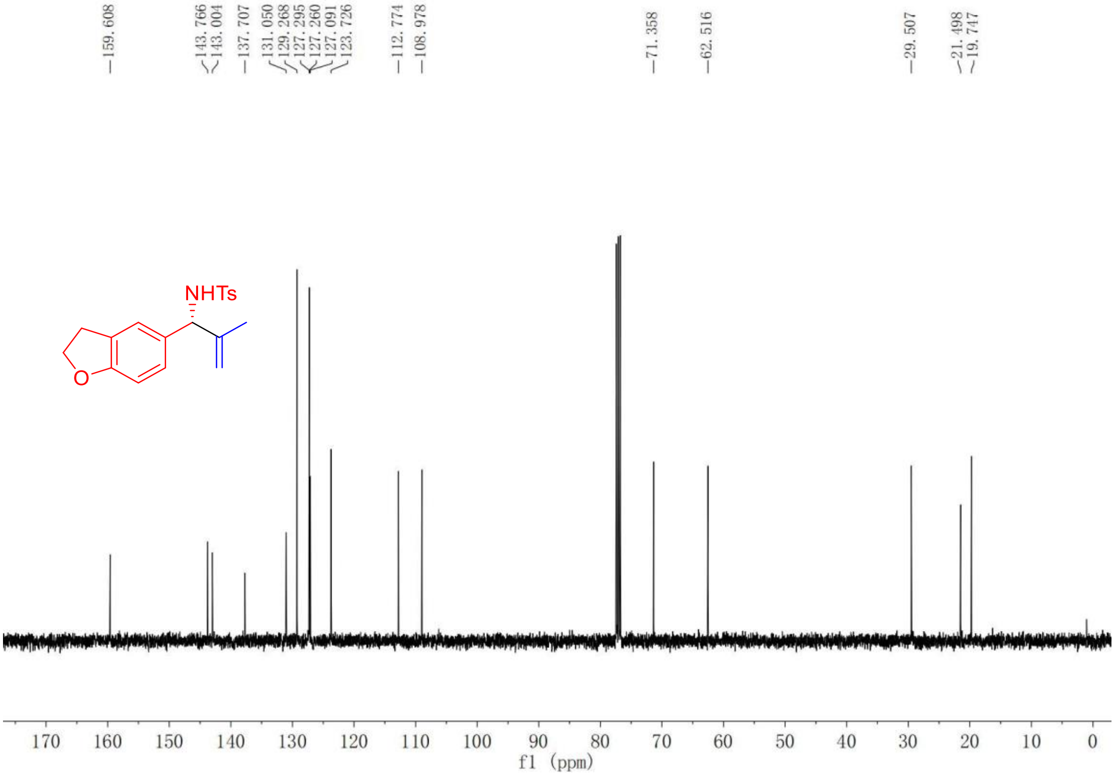

Figure S133. **^{1}H NMR spectra (400 MHz, Chloroform-*d*) of (*R*)-*N*-((2,3-Dihydrobenzofuran-5-yl)(1-methylcyclopropyl)methyl)-4-methylbenzenesulfonamide (5la).**

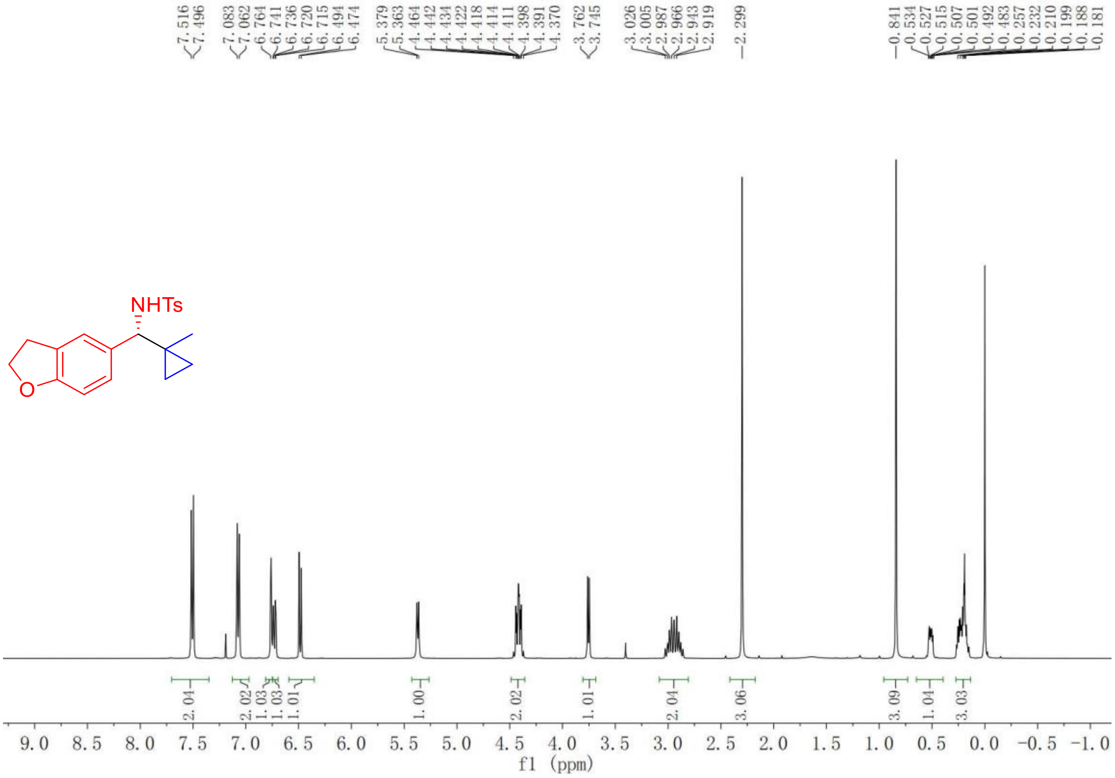

Figure S134. **$^{13}C\{^1H\}$ NMR spectra (100 MHz, Chloroform-*d*) of (*R*)-*N*-((2,3-Dihydrobenzofuran-5-yl)(1-methylcyclopropyl)methyl)-4-methylbenzenesulfonamide (5la).**

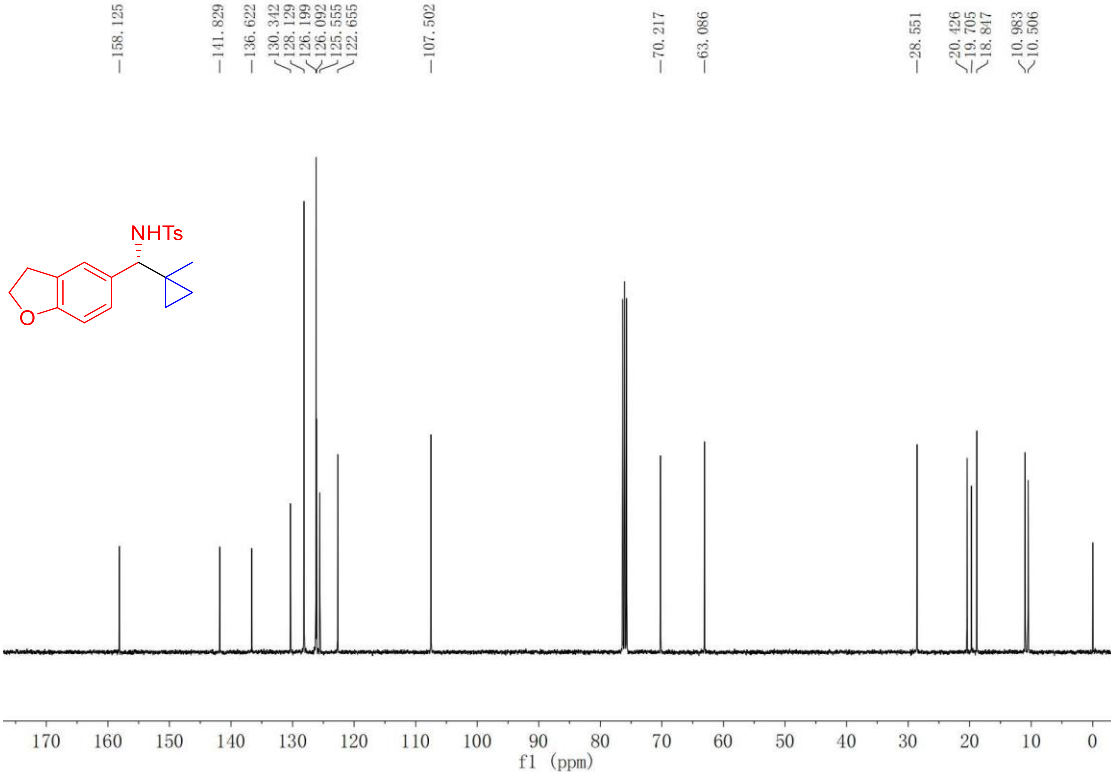

Figure S135. HPLC Chromatography of (*R*)-*N*-((2,3-Dihydrobenzofuran-5-yl)(1-methylcyclopropyl)methyl)-4-methylbenzenesulfonamide (5la).

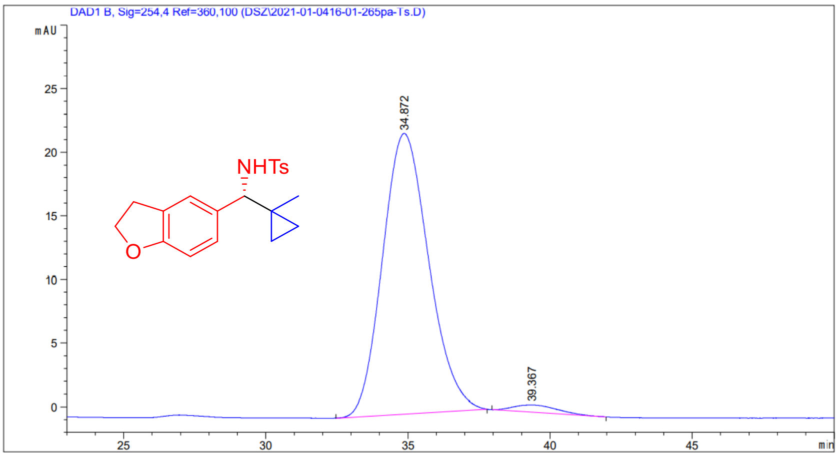

Signal 1: DAD1 B, Sig=254,4 Ref=360,100

Peak #	RetTime [min]	Type	Width [min]	Area [mAU*s]	Height [mAU]	Area %
1	34.872	BBA	1.7222	2484.42090	22.05529	97.5621
2	39.367	BB	1.2595	62.08019	5.79637e-1	2.4379

Totals : 2546.50109 22.63492

Figure S136. ^{1}**H NMR spectra (400 MHz, Chloroform-*d*) of (*R*)-1-(2,3-Dihydrobenzofuran-5-yl)-2-methylpropan-1-amine (6la).**

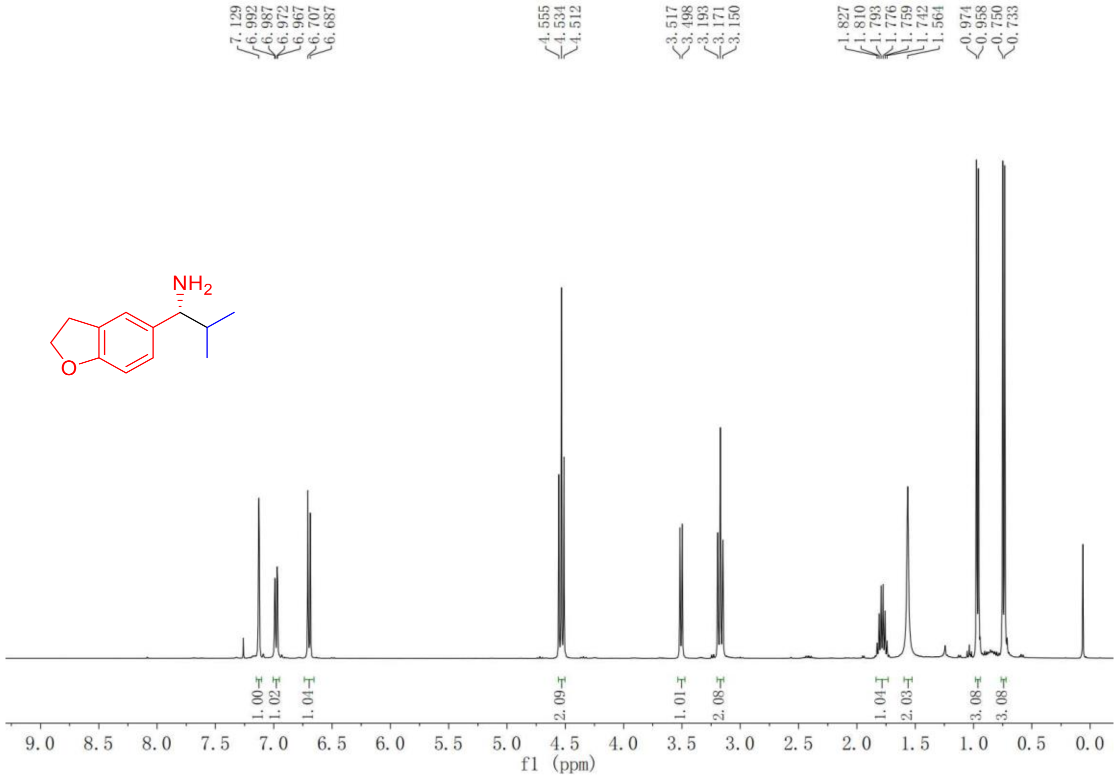

Figure S137. ^{13}**C{**^{1}**H} NMR spectra (100 MHz, Chloroform-*d*) of (*R*)-1-(2,3-Dihydrobenzofuran-5-yl)-2-methylpropan-1-amine (6la).**

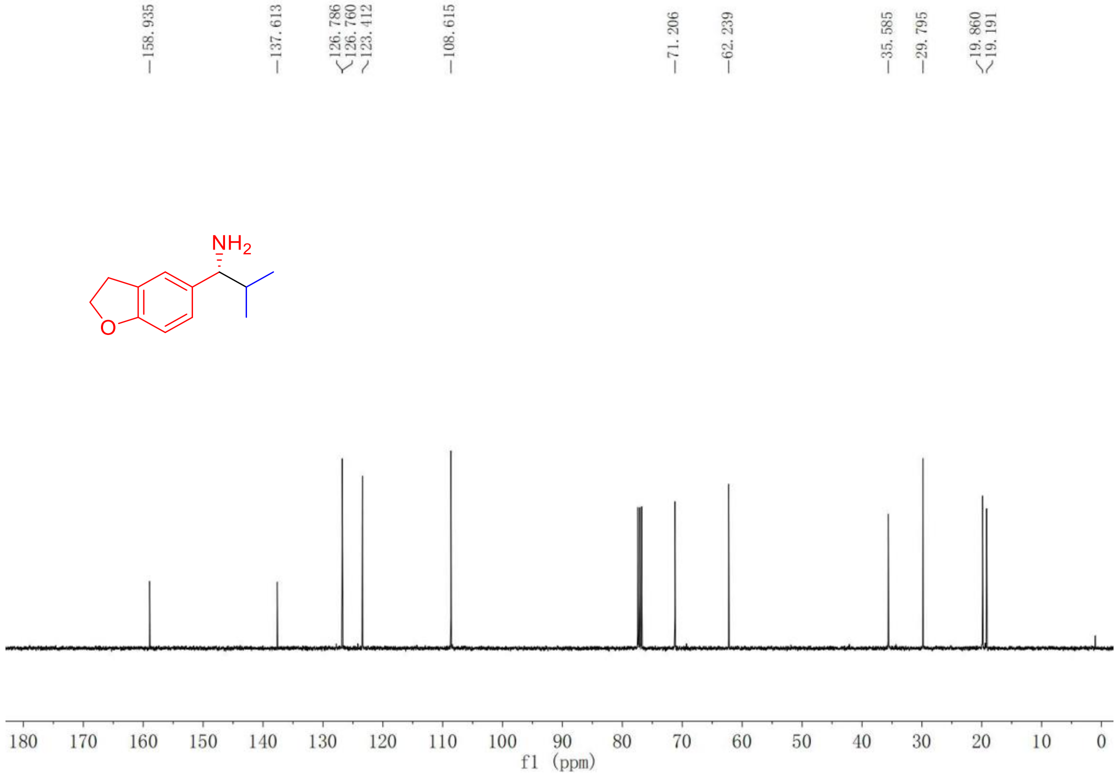

Figure S138. HPLC Chromatography of (*R*)-1-(2,3-Dihydrobenzofuran- 5-yl)-2-methylpropan-1-amine (6la).

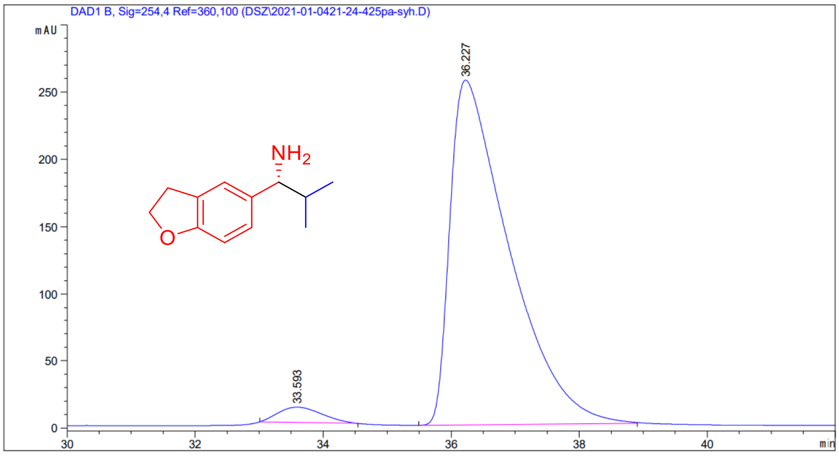

Signal 1: DAD1 B, Sig=254,4 Ref=360,100

Peak #	RetTime [min]	Type	Width [min]	Area [mAU*s]	Height [mAU]	Area %
1	33.593	HHA	0.7506	535.47443	11.34589	3.1494
2	36.227	HHA	0.9333	1.64672e4	256.41229	96.8506

Totals : 1.70026e4 267.75818
